# Molecular Characterization of Chimeric *Staphylococcus aureus* Strains from Waterfowl

**DOI:** 10.3390/microorganisms12010096

**Published:** 2024-01-03

**Authors:** Stefan Monecke, Sascha D. Braun, Maximillian Collatz, Celia Diezel, Elke Müller, Martin Reinicke, Adriana Cabal Rosel, Andrea T. Feßler, Dennis Hanke, Igor Loncaric, Stefan Schwarz, Sonia Cortez de Jäckel, Werner Ruppitsch, Dolores Gavier-Widén, Helmut Hotzel, Ralf Ehricht

**Affiliations:** 1 Leibniz Institute of Photonic Technology (IPHT), Leibniz Center for Photonics in Infection Research (LPI), 07745 Jena, Germany; 2 InfectoGnostics Research Campus, 07743 Jena, Germany; 3 Institute for Medical Microbiology and Virology, Dresden University Hospital, 01307 Dresden, Germany; 4 Austrian Agency for Health and Food Safety, Institute for Medical Microbiology and Hygiene, 1220 Vienna, Austria; 5 Institute of Microbiology and Epizootics, Centre for Infection, Medicine School of Veterinary Medicine, Freie Universität Berlin, 14163 Berlin, Germany; 6 Veterinary Centre for Resistance Research (TZR), School of Veterinary Medicine, Freie Universität Berlin, 14163 Berlin, Germany; 7 Institute of Microbiology, University of Veterinary Medicine, 1210 Vienna, Austria; igor.loncaric@vetmeduni.ac.at; 8 Poultry Clinics and Laboratory Pöppel, 33129 Delbrück, Germany; 9 Department of Pathology and Wildlife Disease, National Veterinary Institute (SVA), 75189 Uppsala, Sweden; 10 Department of Biomedical Sciences and Veterinary Public Health, Swedish University of Agricultural Sciences (SLU), 75007 Uppsala, Sweden; 11 Institute of Bacterial Infections and Zoonoses, Friedrich-Loeffler-Institut (Federal Research Institute for Animal Health), 07743 Jena, Germany; 12 Institute of Physical Chemistry, Friedrich-Schiller University, 07743 Jena, Germany

**Keywords:** *Staphylococcus aureus*, clonal complex 133, clonal complex 522, clonal complex 692, next generation sequencing, bacteriophages, chimerism, horizontal gene transfer, pathogens in waterfowl, One Health

## Abstract

*Staphylococcus aureus* is a versatile pathogen that does not only occur in humans but also in various wild and domestic animals, including several avian species. When characterizing *S. aureus* isolates from waterfowl, isolates were identified as atypical CC133 by DNA microarray analysis. They differed from previously sequenced CC133 strains in the presence of the collagen adhesin gene *cna*; some also showed a different capsule type and a deviant *spa* type. Thus, they were subjected to whole-genome sequencing. This revealed multiple insertions of large regions of DNA from other *S. aureus* lineages into a CC133-derived backbone genome. Three distinct strains were identified based on the size and extent of these inserts. One strain comprised two small inserts of foreign DNA up- and downstream of *oriC*; one of about 7000 nt or 0.25% originated from CC692 and the other, at ca. 38,000 nt or 1.3% slightly larger one was of CC522 provenance. The second strain carried a larger CC692 insert (nearly 257,000 nt or 10% of the strain’s genome), and its CC522-derived insert was also larger, at about 53,500 nt or 2% of the genome). The third strain carried an identical CC692-derived region (in which the same mutations were observed as in the second strain), but it had a considerably larger CC522-like insertion of about 167,000 nt or 5.9% of the genome. Both isolates of the first, and two out of four isolates of the second strain also harbored a hemolysin-beta-integrating prophage carrying “bird-specific” virulence factors, ornithine cyclodeaminase D0K6J8 and a putative protease D0K6J9. Furthermore, isolates had two different variants of SCC elements that lacked *mecA/mecC* genes. These findings highlight the role of horizontal gene transfer in the evolution of *S. aureus* facilitated by SCC elements, by phages, and by a yet undescribed mechanism for large-scale exchange of core genomic DNA.

## 1. Introduction

*Staphylococcus aureus* (*S. aureus*) is a versatile pathogen that does not only occur in humans but also in various wild and domestic animals. While a lot of typing data are available from medical settings and from livestock-associated methicillin-resistant *S. aureus* (MRSA), knowledge of methicillin-susceptible *S. aureus* (MSSA) from animals, especially from wildlife, is still poor. In birds, several lineages (clonal complexes or CCs) of *S. aureus* have been observed. As a simplified generalization, previous *S. aureus* observations in birds fall into four different phylogenetic and ecological categories.

First, predatory and scavenging birds can harbor *S. aureus* strains that originate from mammals and humans. These might be acquired by consumption of meat, carcasses or offal, or by transmission due to contact with contaminated offal. Examples include the presence of CC97- and CC425-MSSA in cinereous vultures (*Aegypius monachus*) in Spain, of a CC97 in a Swedish golden eagle (*Aquila chrysaetos*), of CC130 *mecC*-MRSA and CC398-MRSA in cinereous vultures, storks (*Ciconia ciconia*) and magpies (*Pica pica*) in Spain, of CC5, CC7, CC22, CC25, CC30, CC45, CC59, CC133, CC291 and CC398 (MSSA and MRSA) in Spanish storks, of (presumably rodent-associated) CC49- and CC1956-MSSA as well as of CC130 *mecC-*MRSA in Portuguese owls and of CC22-MRSA-IV (PVL+) and CC1-MRSA-IV in Austrian rooks (*Corvus frugilegus*) [1,2,3,4,5,6].

Second, there is at least one widespread lineage native to birds, CC692 (also known as CC385), that can be found in various bird species as diverse as domestic chickens, woodpeckers, raptors, owls and songbirds [3,6,7] but also in Australian wallaby [8]. Recently, MRSA from this lineage emerged in poultry in Korea [9,10].

Third, there is the curious case of a “human” lineage of *S. aureus*, CC5, that was transmitted from humans to poultry. It adapted to poultry by acquiring a phage carrying “bird-specific” virulence factors, a novel ornithine cyclodeaminase D0K6J8 and a putative novel protease D0K6J9, from CC692 and that spread henceforth in fowl and poultry around the world [7].

Finally, livestock-associated MRSA might be transmitted from other livestock or from farm personnel to chickens and, especially, to turkeys, being selected for by therapeutic use of antimicrobials or by illicit use of antibiotics as prophylactics or growth promotors and being consequentially found in poultry meat products. These strains are most commonly CC9- and CC398-MRSA, but other lineages also have been observed [9,11,12,13,14,15,16,17,18,19,20,21].

When characterizing animal strains from various wild, zoo and domestic animals using DNA microarrays as a molecular typing and screening tool, we identified two different strains with hybridization profiles that were broadly consistent with CC133, a well-known and previously sequenced ruminant lineage (ED133, GenBank CP001996.1) but that differed in particular features, such as carriage of the collagen adhesin gene *cna* as well as in capsule and *spa* types (for details see below). Thus, these strains were suspected to have emerged through large-scale genomic replacements or chimerism.

Mobile genetic elements play a considerable role in the evolution of *S. aureus*. They might also affect host specificity, as discussed above for the “bird-specific” phage in CC692, as well as in poultry-adapted CC5 and CC9 (see [7] and genomes from Bio Project PRJNA693396). However, this is not the only pathway to horizontal gene transfer as large-scale genomic replacements or chimerism might also contribute. Here, by a yet unknown mechanism, considerable parts of the genome can be transferred and integrated into *S. aureus* of other, frequently rather unrelated, lineages. Known examples of chimeric strains are Sequence Types (ST) 34/42 (a common MSSA lineage resulting from a gene transfer from CC10 into a CC30 genome, [22,23]), a CC1 × CC80 chimera from East Africa [24], ST71 (a chimeric MSSA observed in Irish sheep in which a fragment of unknown origin is integrated into a CC97 genome, [25]), ST239 (a pandemic MRSA clone in which CC30 DNA is integrated into a CC8 genome, [22]), a CC9 × CC398 chimera from European livestock [26], ST617 (a CC8 × CC45 chimera, [27]), ST1048 and ST1774 (chimeric MRSA from Hong Kong, [28]), ST2249 (a chimeric MRSA from Australia, involving three parental lineages, CC8, CC30 and CC45, [29]) and ST6610 (a CC8 × CC140 chimeric MRSA from East Africa, [30]).

The authors thus assumed that the “deviant CC133” strains from waterfowl mentioned above might have been such hybrid or chimeric strains, too. Therefore, these observations prompted further investigations, including whole-genome sequencing (WGS), analyses of single nucleotide polymorphisms (SNPs) and Core Genome Multilocus Sequence Typing (cgMLST). The results of these studies shall be discussed herein.

A presence of hybrid or chimeric *S. aureus* strains in waterfowl has implications for diverse fields, from microbiology and epidemiology to wildlife conservation and public health. This highlights the concept of One Health, which emphasizes the interconnectedness of human, animal, and environmental health [31,32]. Identifying chimeric strains in waterfowl could reveal pathways of transmission between wildlife, livestock, and humans, aiding in the prevention and management of infectious diseases. Furthermore, chimerism and hybridization can result in the emergence of novel bacterial variants with unique combinations of genetic traits. Such variants may possess altered virulence factors or antimicrobial resistance profiles, impacting disease severity and treatment strategies.

## 2. Materials and Methods

### 2.1. Strains

Seven study isolates were identified as atypical CC133 by DNA microarray hybridization during diagnostic work or work on other projects [3,14], and later subjected to whole-genome sequencing (WGS) to explain the irregular features observed by array analysis (see below). These included isolate 15V8707 from a wild mute swan (*Cygnus olor*) found dead in Sweden, isolates V315 and V482 from diagnostic samples (necropsy material from the heart) of domestic ducks (*Anas platyrhynchos domesticus*) submitted to a veterinary laboratory in North Rhine-Westphalia, Germany, IMT40427 collected from a white-faced whistling duck (*Dendrocygna viduata*) in a German zoo, as well as isolates 511509-22, 511510-22 and 511525-22, also obtained from domestic ducks, but in Austria.

In addition, a CC522 isolate from a domestic goat (*Capra aegagrus hircus*) from a German petting zoo was sequenced (17CS1042) to obtain an unfragmented, high-quality sequence of that clonal complex.

### 2.2. Characterization by DNA Microarray

Isolates were identified as *S. aureus* by microarray-based analysis, which facilitated the detection of resistance genes, virulence markers and other genes of interest, as well as an assignment to a clonal complex within *S. aureus*.

The microarrays, related protocols and methods, as well as probe and primer sequences, have previously been described in detail [28,33,34]. Briefly, DNA obtained by enzymatic lysis of *S. aureus* cells from overnight cultures was used as a template for a multiplex primer elongation reaction (i.e., with only one primer per target) that incorporated molecules of biotinylated dUTP into the single stranded amplicons. The resulting mixture of single-stranded, labeled amplicons was then hybridized into DNA microarrays on which the probes for the target genes were spotted following a predefined coordinate grid. Hybridizations were visualized by the addition of streptavidin-horseradish-peroxidase that, in the subsequent step, caused a dye to precipitate, resulting in the formation of visible spots at those probes that bound labeled amplicons. Digital images of the arrays were analyzed for the presence or absence of specific genes as well as for similarity to known reference profiles, allowing the identification of species, clonal complex and strain (reading device and software by Inter-Array GmbH, Bad Langensalza, Germany).

### 2.3. Nanopore Sequencing

The Oxford Nanopore Technologies (ONT) MinION platform was employed to perform WGS on four *S. aureus* isolates (swan isolate 15V8707, duck isolates V315 and V482, and goat isolate 17CS1042, for comparison).

Genomic DNA was extracted from an overnight culture cultivated at 37 °C on Columbia Blood Agar plates (Becton Dickinson GmbH, Heidelberg, Germany) using the Macherey and Nagel NucleoSpin Microbial DNA kit (MACHEREY-NAGEL GmbH & Co. KG, Dueren, Germany). The DNA library was generated employing the 1D genomic DNA ligation kit (SQK-LSK109 for Flongle, and SQK-NBD114.24 for Flow cell, ONT), following the manufacturer’s instructions with minor adjustments. Before library preparation, an Agencourt AMPure XP purification step was conducted in a ratio of 1/1 (*v*/*v*). We omitted the g-TUBE shearing and opted for a combined step of repairing potential DNA nicks and ends using the NEBNext FFPE DNA Repair Mix and NEBNext Ultra II End repair/dA-tailing Module from New England Biolabs, USA. To ensure comprehensive repair, the incubation time was doubled. Following these procedures, a second purification step was carried out using AMPure beads, followed by the ligation of sequencing adapters onto the prepared ends (Flongle). For DNA samples sequenced on a flow cell, a barcoding step was performed prior to adapter ligation following a third AMPure purification step. A final purification step utilizing AMPure beads along with the addition of ONT sequencing buffer and loading beads was performed. Before sequencing, an initial assessment of the Flongle flow cell (ID: ACG143) and the flow cells (IDs FAP37324, FAW75613, and FAW72995) revealed the presence between 25 (Flongle) and 1700 (flow cell) active pores. The libraries were quantified using a Qubit 4 Fluorometer (ThermoFisher Scientific, Waltham, MA, USA), and 500 ng per strain were loaded at a concentration totaling approximately 500 ng per strain onto the flow cells.

The duration of the sequencing process was 48 h for Flongles and 72 h for flow cells. Data from the sequencing device were analyzed by using the software MinKNOW version 21.02.1 up to 23.04.6, depending on the sequence run. Base calling was done by using the software guppy basecaller (version 4.5.4 + 66c1a7753 up to 6.5.7 + ca6d6af, ONT, Oxford, UK). The program translated the MinION raw reads (FAST5) into quality tagged sequence reads (4000 reads per FASTQ-file). For raw data processing, the barcode trimming option (model version: dna_r9.4.1_450bps_hac.cfg for the Flongle and dna_r9.4.1_450bps_sup.cfg and dna_r10.4.1_e8.2_260bps_sup.cfg for flow cell) was used. Contig assembling of each strain’s quality tagged sequence reads were done by using the software Flye (version 2.8.3-b1695 up to 2.9.1-b1780). The polishing of the assembled contigs was performed in two steps. Racoon (v1.4.21 or 1.5.0) was run iteratively for four rounds with the following parameters: match 8; mismatch 6; gap 8, and windows-lengths 500. Afterwards, medaka (version 1.4.3 up to 1.8.0) was used for the final polishing of the last racon-polished assembly. For medaka, the following models were used: r941_min_high_g360, r941_min_sup_g507 and r10.4.1_e82_400bps_sup_g615. The resulting corrected assemblies were used for further analysis.

Nanopore sequences of the study strains and of the comparator isolate from a goat have been published via GenBank and are also provided as Supplemental Files (swan isolate 15V8707, CP138363.1 = Supplemental File S1a; duck isolate V315, CP138362.1 = Supplemental File S1c; duck isolate V482 CP138361.1 = Supplemental File S1g; goat isolate 17CS1042, CP138360.1 = Supplemental File S1h).

### 2.4. Illumina Sequencing

WGS of the whistling duck isolate was performed using the Illumina MiSeq platform (Illumina, Inc., San Diego, CA, USA). The DNA was extracted using the QIAamp DNA Mini Kit (QIAGEN, Hilden, Germany) with adaptations for staphylococci, as described previously [35]). The library preparation for WGS was carried out using the Nextera XT DNA Library Preparation Kit (Illumina, Inc., San Diego, CA, USA) according to the manufacturer’s instructions. The 2 × 300 bp paired-end sequencing in 40-fold multiplexes was performed on the Illumina MiSeq platform (Illumina, Inc., San Diego, CA, USA). Genome sequences were de novo assembled using the Mimicking Intelligent Read Assembly (MIRA) v4.0 (Biomatters Ltd., Auckland, New Zealand) with default settings as a plugin within Geneious v10.1.3.

From the Austrian domestic duck isolates, high-quality genomic DNA (gDNA) was isolated from overnight cultures using the MagAttract HMW DNA Kit (Qiagen, Hilden, Germany) and quantified on a Qubit 2.0 Fluorometer (Thermo Fisher Scientific, Waltham, MA, USA) using the dsDNA BR Assay Kit (Thermo Fisher Scientific, Waltham, MA, USA). Nextera XT DNA Library Preparation Kit (Illumina, San Diego, CA, USA) was used for library preparation and paired-end sequenced with a read length of 2 × 300 base pairs on a NextSeq 2000 instrument according to the instructions of the manufacturer (Illumina, San Diego, CA, USA). Raw reads were trimmed with Trimmomatic v0.36 using default parameters. SPAdes version 3.11 and SeqSphere  +  version 5.1.0 (Ridom, Münster, Germany) were used for read assembly. Contigs were filtered for a minimum coverage of 5× and a minimum length of 200 bp.

Illumina sequences of the study strains are provided as Supplemental Files S1b (duck isolate 511525-22), S1d (duck isolate 511509-22), S1e (duck isolate 511510-22) and S1f (whistling duck isolate IMT40427).

### 2.5. Core Genome MLST

In addition to the array, *spa* and MLST data, cgMLST—as provided by the PubMLST database (https://pubmlst.org/bigsdb?db=pubmlst_saureus_seqdef, accessed on 12 December 2023; [36])—was used in order to screen genomes of various lineages of *S. aureus* in order to assess whether they belonged to lineages that could be considered as parental strains. In this approach, alleles of the individual target genes are assigned with code numbers that can be directly compared. For the study strains, as well as for published genomes used as comparators, these code numbers are listed in Supplemental File S2. The advantage of this approach is ease of use and speed. The disadvantage is that the allele numbers are assigned chronologically by the date of submission to the database, and thus, they do not allow inferring relatedness. Figuratively spoken, alleles “1” and “99” could differ in just one single SNP, while alleles “1” and “2” might differ in dozens of SNPs. Therefore, sequences were also compared directly.

### 2.6. Analysis of Similarities and Differences of Genes and Genome Sequences

For the purpose of the study, a set of 2257 genes was defined that either belonged to the core genome or to major genomic islands and that were present in at least one of the lineages relevant for this study. These genes are listed in Supplemental Files S3a and S3b. They always follow the same order within the genome (with only one exception being published, to the best of our knowledge, where a third of the genome was “flipped” or inverted [37]) and are usually, but not universally, of the same length. For every single gene, alignments were constructed (FAMSA, version 1.6.2; [38]), and single nucleotide polymorphisms were counted. Gaps, duplications and sequencing errors were counted likewise. The total number of mismatches was expressed as the percentage using 100% of the total length of the consensus sequence of the respective gene. If a gene was absent in one strain but present in another, the difference was set to 100%.

For comparing regions of several genes or entire genomes, the median of the differences, expressed in percent as described above, for all genes considered was used. Roughly, a median difference of 5% across the entire genome would be consistent with an affiliation to a different species (such as *S. aureus*, *S. argenteus*, *S. schweitzeri* and *S. roterodami* [39]). Two sequences with a median difference of around 0.5% would belong to different clonal complexes within *S. aureus*, and median values of around 0.1% or less could be considered due to random variation within a clonal complex.

Calculations and graphic visualizations for use in figures were performed using Excel 2019 (Microsoft, Redmond, WA, USA).

## 3. Results

### 3.1. Array Profiles, Spa and MLST of Waterfowl CC133 Strains

When characterizing poultry isolates, two different “deviant CC133” profiles could be discerned. They resembled CC133 with regard to the presence of *agr*-I alleles, to a presence of *lukD* accompanied by poor reactivity of the probe to *lukE*, to the allelic variants of *ssl/set* and MSCRAMM genes and the absence of the enterotoxin gene cluster *egc*, of the enterotoxin homolog ORF CM14 and of *sasG*.

One of these hybridization profiles was observed for an isolate from a Swedish Mute Swan (15V8707) and one from an Austrian duck (511525-22). It was characterized by affiliation to capsule type 8, which is the same capsule type as in regular CC133 strains. The isolates carried the collagen adhesin gene *cna*, which is normally absent from CC133 strains. Isolates belonged to ST3269, *arcC-*6, *aroE*-66, *glpF*-46, *gmk*-2, *pta*-372, *tpi-*50, *yqiL-*18. This is a single locus variant of ST133, and the difference (*pta-*372 rather than -7) can be explained by mutation rather than recombination. The *spa* type of two isolates was determined to be t1166, repeats 03-16-21-17-23-13-17-17-17-23-24, which can be considered as a variant of the *spa* type of typical CC133 (such as ED133, GenBank CP001996.1: t2678, 03-16-12-21-17-23-13-17-17-17-23-24).

A second hybridization profile was observed for two German and two Austrian isolates from domestic ducks (V315, V482, 511509-22 and 511510-22) and the isolate from a whistling duck (IMT40427). The most conspicuous differences to regular CC133 isolates were the presence of the collagen adhesin gene *cna,* the affiliation to capsule type 5 (rather than 8) and different *spa* types (t2379, repeats 26-17 or t15307, 26-23-12-21-17-34-25-17). MLST types were ST133 (6-66-46-2-7-50-18), ST2111 (06-66-46-2-7-50-7), ST3270 (6-66-46-2-373-50-18) and ST4432 (18-66-46-2-7-50-18).

### 3.2. Core Genome MLST

The dataset for cgMLST is provided in Supplemental File S2. It indicates that the region from SAUR0001 to SAUR0005 in isolates 15V8707 and 511525-22, as well as from SAUR0001 to SAUR0238 in the other isolates, match CC692 profiles. SAUR2900 to SAUR2937 in isolates 15V8707 and 511525-22 most closely resembled CC522 profiles, while CC522-like regions encompassed SAUR2795 to SAUR2937 in V482 or SAUR2884 to SAUR2937 in the other four study isolates. Other potential donor lineages (CC5, ST71, CC182, CC707, CC1956) for these regions were ruled out based on the dissimilarity of their cgMLST profiles. The remaining parts of the genomes of the seven study isolates were in accordance with CC133 profiles.

### 3.3. The Genome of the Swan Isolate 15V8707

The Swedish swan isolate 15V8707 was nanopore-sequenced in order to obtain a complete, unfragmented contig representing its entire genome. In addition, an Illumina sequence of a second isolate from an Austrian duck (511525-22) was available for comparison.

**Figure 1:**
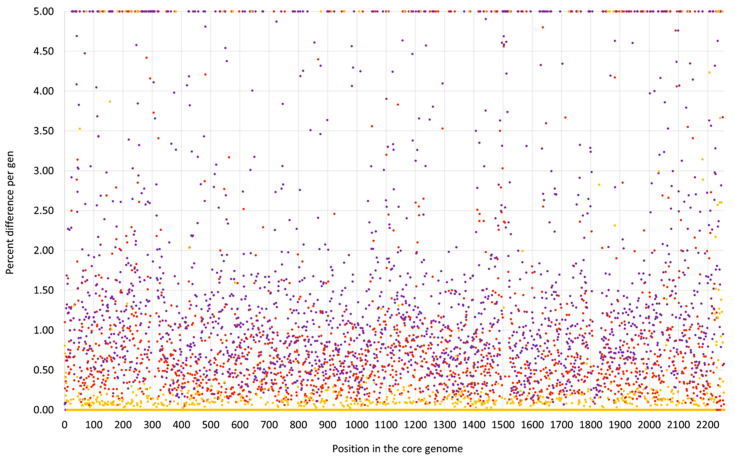
SNP analysis for the swan isolate 15V8707 compared to the CC133 reference sequence ED133, GenBank CP001996.1 (yellow dots), the CC692 reference sequence X22, GenBank CP042650.1 (purple dots) and to the CC522 goat isolate 17CS1042 (brown dots). The vertical axis shows the percentage of difference per gene, the horizontal one the position in the genome (see Supplemental File S3a/S3b for the gene names, positions and exact numerical values).

**Figure 2:**
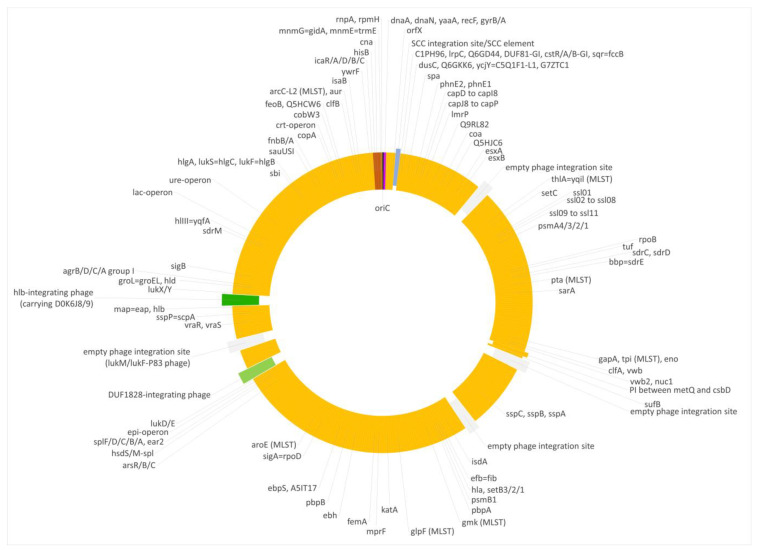
Diagram of the genome of the swan isolate 15V8707. Yellow, regions of CC133 origin; purple, regions of CC692 origin; brown, regions of CC522 origin. SCC elements are shown in blue, prophages in green and empty phage integration sites in grey. Approximately to scale, with one degree corresponding roughly to 8000 nt.

Most of the genome of this lineage matched the CC133 reference sequence of ED133, GenBank CP001996.1. This part included the MLST markers and the *spa* gene as well as the operon encoding capsule-related genes. However, two regions significantly differed from the CC133 sequence (Figure 1 and Figure 2).

First, a very short fragment downstream of *oriC,* including *dnaA*, *dnaN*, *yaaA*, *recF* and *gyrB* (i.e., from *oriC* to position 6968 of the genome), matched the CC692 reference sequence showing a median difference to X22, GenBank CP042650.1, of 0.07% and to ED133 of 0.47%. The size of this alien insert corresponds to ca. 0.25% of the strain´s genome.

Second, the region from *hisB* (cgMLST designation at https://pubmlst.org/, accessed on 12 December 2023: SAUR2899 (SAR_RS14495)) to *mnmE* = *trmE*, SAUR2937 (SAR_RS14685) was clearly deviant from ED133, at a median difference of 1.19%. The following two markers prior to *oriC* (*rnpA* and *rpmH*) are highly conserved among *S. aureus,* so their provenience could not be determined. This region approximately spanned from positions 2,790,364 to 2,828,252, i.e., about 38,000 nt or 1.33% of the strain´s genome. It also included the *cna* gene, which is normally absent from CC133 strains and whose detection by array prompted the investigation. This region was essentially identical to the CC522 isolate sequenced for comparison, showing only 0.03% median difference.

Both deviant regions were not flanked by transposons, phages or other mobile genetic elements.

### 3.4. The Genome of the Duck Isolate V315

The isolate V315 yielded the capsule type 5/*cna*-positive array profile. However, upon sequencing, it was observed that there were two distinct strains, one being represented by V315 and the other by isolate V482, which cannot be distinguished by the microarray used. The differences will be explained below.

**Figure 3:**
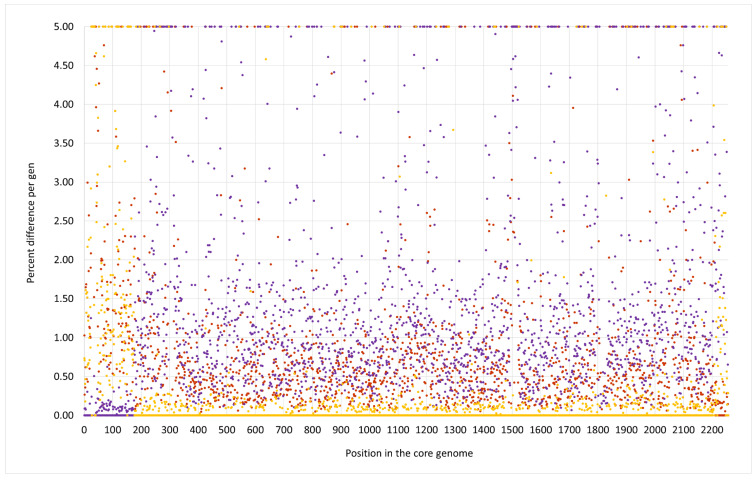
SNP analysis for the duck isolate V315 compared to the CC133 reference sequence ED133, GenBank CP001996.1 (yellow dots), the CC692 reference sequence X22, GenBank CP042650.1 (purple dots) and to the CC522 goat isolate 17CS1042 (brown dots). The vertical axis shows the percentage of difference per gene, the horizontal one the position in the genome (see Supplemental File S3a/3b for the gene names, positions and exact numerical values).

**Figure 4:**
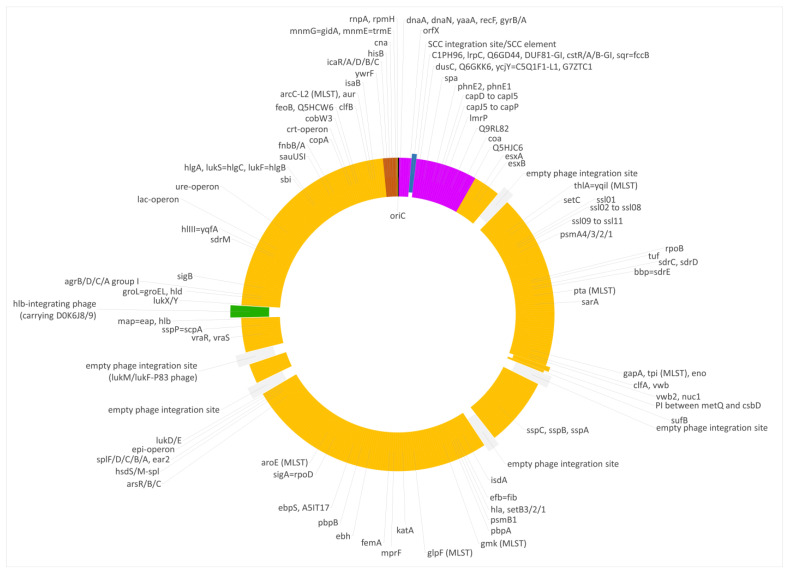
Diagram of the genome of the duck isolate V315. Yellow, regions of CC133 origin; purple, regions of CC692 origin; brown, regions of CC522 origin. SCC elements are shown in blue, prophages in green and empty phage integration sites in grey. Approximately to scale, with one degree corresponding roughly to 8000 nt.

As in the swan isolate 15V8707 described above, the region downstream *oriC* differed from regular CC133 but resembled CC692 (Figure 3 and Figure 4). However, the CC692-like region in V315 was considerably larger than in the swan isolate, ranging to Q5HJC6 (ending at position 256,955 of the strain´s genome or equalling ca. 9.2% of the strain´s genome). The recombination site might even be situated within the Q5HJC6 gene as the duck isolate shared nine SNPs out of the first ca. 700 nt of that gene (out of 1044 nt) with the X22 sequence, while in the remaining part of the gene, two SNPs matched the ED133 sequence.

The median difference per gene to ED133 was 1.38%, while genes of that region were identical to the CC692 reference sequence of X22 (median difference of 0.0%). The CC692-like region included the capsule locus and the *spa* gene. However, two differences were observed in comparison to X22. First, five genes (DUF81-GI, *cstR*-GI, *cstA*-GI, *cstB*-GI, *sqr = fccB*) of a genomic island downstream *oriX* und upstream of *dusC* that is typically found in CC692 (being present in X22, CP042650.1; CKMM-401M, CP129360.1; NCTC9546, UHAK01000002.1) were absent, while three other genes of that GI were still present (C1PH96, *lrpC*, Q6GD44). Second, a small deletion affected genes *phnE2* and *phnE1,* removing 5 nt at the overlap of their respective ends and, thus, truncating both of them.

V315 also carried an insert of CC522 provenance. It spanned the region from *ywrF* (cgMLST: SAUR2882 (SAR_RS14410) starting at position 2,738,034) to, at least, *mnmE = trmE* (ending at position 2,791,551), that is at least approximately, 53,500 nt or 1.9% of the genome. Again, the provenance of *rnpA* and *rpmH* remained unclarified because of their conserved sequence. The former gene differed in one SNP from ED133, X22 and the goat isolate; the latter gene was identical in all isolates. The median difference to ED133 was 1.06%, but the difference to the ST522 isolate 17CS1042 sequenced for this study was 0.07%. This region included the *cna* gene, thus explaining the difference to “normal” ED133-like CC133 isolates as observed by microarray hybridization.

Three additional isolates, 511509-22, 511510-22 and IMT40427 showed, according to Illumina data and cgMLST analyses, the same features as V315.

### 3.5. The Genome of the Duck Isolate V482

**Figure 5:**
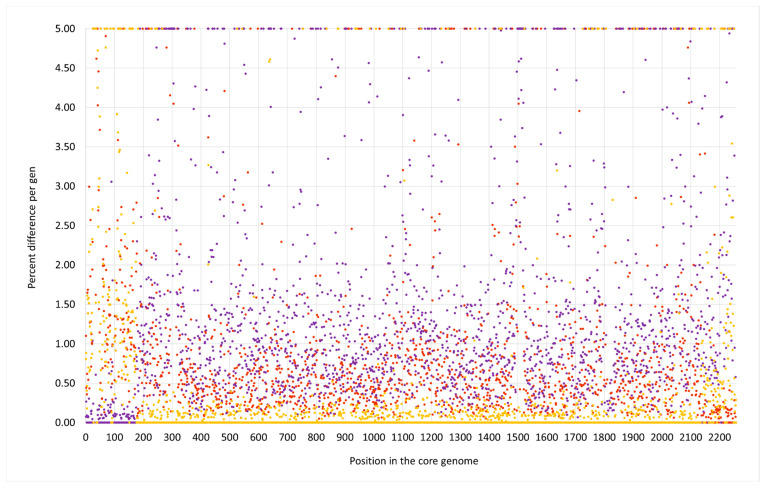
SNP analysis for the duck isolate V482 compared to the CC133 reference sequence ED133, GenBank CP001996.1 (yellow dots), the CC692 reference sequence X22, GenBank CP042650.1 (purple dots) and to the CC522 goat isolate 17CS1042 (brown dots). The vertical axis shows the percentage of difference per gene, the horizontal one the position in the genome (see Supplemental File S3a/3b for the gene names, positions and exact numerical values).

**Figure 6:**
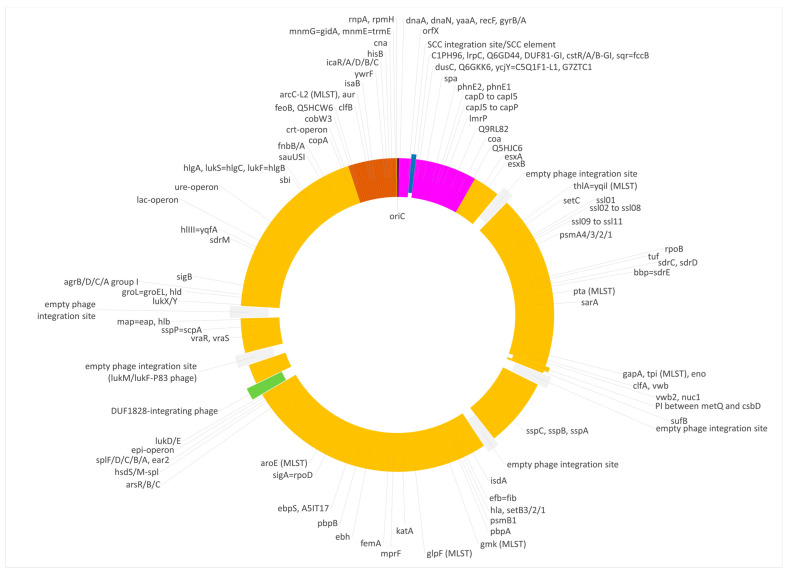
Diagram of the genome of the duck isolate V482. Yellow, regions of CC133 origin; purple, regions of CC692 origin; brown, regions of CC522 origin. SCC elements are shown in blue, prophages in green and empty phage integration sites in grey. Approximately to scale, with one degree corresponding roughly to 8000 nt.

The isolate V482 represented a second capsule type 5/*cna*-positive strain (Figure 5 and Figure 6). It carried the same CC692-like insert downstream of *oriC* as described for isolate V315. Here again, DUF81-GI, *cstR*-GI, *cstA-*GI, *cstB*-GI and *sqr = fccB* were absent, and the 5 nt deletion affecting *phnE2* and *phnE1* was also observed. Here, the median difference to ED133 was 1.33% and to X22, 0.00%. However, the CC522-like insert in that strain was much larger than in the others. It spanned from *cobW3* (cgMLST: SAUR2793 (SAR_RS13965)) to, at least, *mnmE* = *trmE*/*rnpA.* The *rnpA* differed in the same SNP from the comparator sequences as observed for V315, while the *rpmH* sequence was conserved among all study strains regardless of their affiliation. The difference to ED133 was 0.53%, and to the CC522 goat isolate, 0.06%. The insert size was about 167,000 nt or 5.9% of the strain´s genome.

### 3.6. SCC Elements in the Studied Isolates

The swan isolate 15V8707 shared with ED133 a SCC element without *mecA/C* that comprised a helicase gene (ED133 locus tag: SAOV_0027), a gene encoding a putative protein (C5N0V3, SAOV_0028), a putative noncoding RNA sequence and a gene coding a histidine triad domain protein (C5N0V4, SAOV_0029), followed by three different transposase gene copies (SAOV_0030 to SAOV_0032). The isolate 511525-22 harbored a similar SCC element (involving contigs 029, 155, 131, 115rc and 019).

The other isolates harbored a different SCC element. In V482, it was accompanied by integrated remnants of plasmids and/or transposons, and in 511509-22, 511510-22, as well as in IMT40427, it was split across several contigs. Therefore, the sequence from V315 shall be described here as a reference for this type of SCC element (Table 1). From the direct repeat of SCC at the end of *orfX* to the first region homologous to the CC692-MSSA sequence of X22, it encompassed 18,540 nt. It comprised first a gene for a “hypothetical protein” observed in *Mammaliicoccus lentus* (CP116807.1, locus tag PN942_09245), followed by another one for a “putative protein” (Q3T2M7, locus tag SAPIG0032 in the CC398-MRSA sequence AM990992.1).

This was followed by a variant of a putative *ccr* gene homolog previously named *ccrAA* and by a variant *ccrC* gene that showed about 96% sequence identity with *M. lentus* sequences (CP116807.1; position: 1,831,493 to 1,833,169 and CP059679.1; pos.: 1,831,493 to 1,833,169) and only about 77% with previously sequenced *S. aureus ccrC* genes (such as the second copy of the *ccrC* gene in the CC398 MRSA sequence AM990992.1, locus tag SAPIG0050). Thus, it might tentatively be named *ccrC3*. In addition, there was a gene encoding SAUGI family uracil-DNA glycosylase inhibitor again matching an *M. lentus* sequence (CP116807.1, locus tag PN942_09215), another putative protein gene (Q0P7G0, locus tag SAPIG0036 in the CC398-MRSA) and a gene encoding a DUF1643 putative protein (CP065795.1, locus tag I7830_05530). This was followed by a type I restrictionmodification system (*hsdR/S/M*) most similar to the one in *Mammaliicoccus stepanovicii,* GenBank LT906462, positions 40,917 to 46,940.

The CC522 goat isolate 17CS1042 harbored no SCC element, with *orfX* being directly followed by genes encoding G0LPR5 (putative adenylate/guanylate cyclase) and G0LPR6 (encoding a “putative protein”), known to be part of genomic islands at comparable positions in CC22 (locus tags ER16_RS00240 and ER16_RS00245 in EMRSA-15, GenBank CP007659.1) and CC425 (locus tags SARLGA251_00540 and SARLGA251_00540 in LGA251; GenBank FR821779.1). Of note, the CC692 sequence of X22 also did not contain a SCC element.

### 3.7. Prophages in the Studied Isolates

Six different prophages were observed in the study strains and in the reference sequences. They are summarized in Table 2.

The most conspicuous prophage was the one that carries “bird-specific” virulence factors D0K6J8 and D0K6J9. It was present in some of the isolates but not all (see Table 2), and its carriage does not fully correlate with strain affiliation. Interestingly, D0K6J8/D0K6J9-prophages are not identical (Supplemental File S4a). There are the following groups:

(1) The D0K6J8/D0K6J9-prophage which was first observed in the CC5 strain ED98, GenBank CP001781.1, and another one, also from a CC5 sequence, SA01, GenBank CP053075.1;
(2) The D0K6J8/D0K6J9-prophage from CC692 reference sequence X22, CP042650.1, and another one, also from CC692, CKMM-401M, GenBank CP129360.1;
(3) A related prophage from NCTC9546, cgMLST id-42524, GenBank UHAK01000002.1;
(4) The D0K6J8/D0K6J9-prophage from the swan isolate 15V8707 and the one from 511525-22, which were largely identical, although there were some gaps between Illumina contigs in the latter;
(5) The D0K6J8/D0K6J9-prophage from V315 and IMT40427, which were essentially identical to each other.

Although the presence and order of genes in these “bird-specific prophages” were found to be very similar, few significant differences were found (Supplemental File S4a–c).

Most conspicuously, the transcriptional activator *rinB* (locus tag SAAV_2059 in CP001781.1) was in the first group (the two prophages from CC5 strains) colocalized with an antirepressor (locus tag SAAV_2058 in CP001781.1). Contrastingly, *rinB* was in all other bird prophages present in another, shorter variant (such as locus tag SaO11_00291 in CP024649.1) and it was also located elsewhere. Prophages of the second group (in GenBank CP042650.1, CP129360.1) carried an integrated beta-lactamase operon (*blaZ/R/I*). However, the Group (3) prophage (in GenBank UHAK01000002.1) does not, and CC692 isolates, which the authors previously tested, also lacked the beta-lactamase operon [3]. Thus, the presence of *blaZ/R/I* cannot be considered a universal property of CC692 and its prophages. Group (4) prophages carried a large group of genes (ca. 15,000 nt) that interrupts the gene encoding the phage tail tape measure protein (phi-*tmpM*). This cluster included genes for “putative proteins”, transposases, a phage replication initiation factor, conjugal transfer protein and others that were absent from the other prophages. This region is, however, also present in ED98 and CKMM-401M, but there it is localized elsewhere, outside of the phage (CP001781.1: 1747933..1756582, locus tags SAAV_1692 to SAAV_1701, or, respectively, CP129360.1: 2201787..2210436; whereas the prophage sits at 2088250..2140329).

## 4. Discussion

We describe a group of isolates that were assigned to CC133 by MLST but that, by DNA microarray analysis and *spa* typing, displayed unusual features, including the presence of the collagen adhesion encoding gene *cna* in all isolates and of the unusual capsule type in some of these isolates. These features can be explained by multiple insertions of large regions of DNA from other *S. aureus* lineages into a CC133-derived backbone genome. Three distinct strains were identified based on the size and extent of these inserts.

One is represented by the mute swan isolate 15V8707 from Sweden and by an isolate from an Austrian domestic duck. These isolates comprised two small inserts of foreign DNA around *oriC*; one of about 0.25%, originated from CC692, and the other, at 1.3%, slightly larger, was of CC522 provenance. The second strain carried a larger CC692 insert (nearly 10% of the strain´s genome), and its CC522-derived insert was also larger, at nearly 2% of the genome). The third strain, represented by one isolate, carried an identical CC692-derived region (in which the same mutations were observed as in the second strain), but it had a considerably larger CC522-like insertion of about 5.9% of the genome.

Multiple isolates of these CC133 × CC692 × CC522 chimeras were identified that originated from different countries, isolated in different years and from different host species that all, however, belonged to *Anatidae*, i.e., waterfowl [40]. Another but similar CC133 × CC692 × CC522 chimeric strain was found in domestic ducks in China (see below). In addition, an ST2111 isolate was found in Canadian Goose (*Branta canadensis*) feces from Ohio [41], which also could be either CC133 or one of the chimeric lineages described herein. Thus, it can be ruled out that they represent isolated cases resulting accidentally, such as from a possible exposure to a mutagenic agent. It rather appears that these were geographically widespread chimeric strains in wild, domestic and captive waterfowl. If such strains also occur in other wild bird species that coinhabit similar biotopes, such as coots, divers, waders, cranes or gulls, and how common or widespread they are is currently unknown. Two of the parental lineages, CC133 and CC522, are known to occur in small ruminants, such as goats, sheep [42,43,44] or donkeys [45]. The third parental lineage, CC692, is well known to be a widespread lineage in birds (see Introduction). CC692 isolates usually host a beta-haemolysin-integrating lysogenic phage that carries two putative “bird-specific” virulence factors, an ornithine cyclodeaminase D0K6J8 and a putative protease D0K6J9 to which the cross-species spill-over of a human CC5 *S. aureus* lineage into poultry was attributed [7]. These phage-borne virulence factors were also found in some, but not all, deviant CC133 isolates described herein. One might speculate whether the insertion of large fragments of CC522 and CC692 DNA might contribute to a strain´s fitness in a bird/waterfowl host. It is not clear which factors could promote it. Bird strains differ in their capsule types and in the presence of *cna*, which was introduced from CC522 into all deviant CC133 lineages described herein but which, nevertheless, was absent from bird-adapted CC692.

In addition to the chimeric CC133 × CC692 × CC522 lineages described herein, there is a similar strain in Southern Chinese ducks [19]. Two genome sequences were identified (GenBank JAFEOM01 and JAOAKU01, BioProject PRJNA693396). According to them, it is also a CC133 strain with inserts of CC692 and CC522 provenance. These inserts are localized at approximately the same locations as in our strains, albeit they are different. The CC692 region in these sequences was similar to the one in 15V8707 but smaller than in V315/V482, while the CC522 insert was larger than in 15V8707 and V315 but smaller than in V482 (Supplemental File S2). Furthermore, there is a CC692, ST1347 sequence (PubMLST ID 39945, Isolate 61290; see also Supplemental File S2) that can be described as a CC692 genome with a CC522 insert (with a size comparable to the one in isolates 15V8707 and 511525-22). Unfortunately, neither metadata nor the complete sequence are available for this strain.

Foreign inserts of CC692 and CC522 provenance in all three waterfowl lineages described herein, as well as JAFEOM01 and JAOAKU01 were integrated into a CC133 backbone genome. The lineage comprising 15V8707 and 511525-22 clearly emerged independently from the other strains, as the sizes of both its inserts were different (see Figure 1, Figure 2, Figure 3, Figure 4 and Figure 5). It might have originated from two separate gene transfers of CC522- and CC692-derived regions into a CC133 backbone, or it might have been a result of *one* replacement event involving CC133 and a contiguous fragment from the CC692 × CC522 chimeric strain as represented by Isolate 61290, PubMLST ID 39945 (see above). This also applies to JAFEOM01 and JAOAKU01, but because of the different insert sizes, this Chinese strain must have evolved independently from 15V8707 and 511525-22.

For the lineages represented by V315 and V482, it can be assumed that an ancestor of both emerged from a single CC133 × CC692 hybridization event. In both strains, the CC692 insert shows not only the same size and extent but also the same deletions of a part of the *cstA/B/R* genomic island and of the same 5 nt at the overlap of *phnE2* and *phnE1*. This appears unlikely to be an accident, so we assume that first, a CC692 insertion gave rise to the common ancestor of both lineages. This ancestral strain was only later diversified by secondary insertions of different CC522 fragments. Whether the deletions occurred before the hybridization event, i.e., in the CC692 donor strain, or after, in the CC133 × CC692 chimera, cannot be determined. At least it can be said that there is indeed some variability affecting this region in CC692, as there is a CC692 sequence (DICM09_00289_9_HSA; SAMEA2298557) that lacks the entire *cstA/B/R* genomic island while still having normal, not truncated, *phnE2* and *phnE1* genes.

With various, apparently independent recombination events involving CC133, CC692 and CC522 in distinct lineages across four countries, two continents and several host species, one must assume that such chimeras must have a selective advantage in waterfowl hosts. This currently cannot be attributed to specific factors, either phage-borne or core genomic, but *S. aureus* isolates from waterfowl should be checked for features of these strains in order to obtain a more complete picture.

While antimicrobial resistance is a significant issue in *S. aureus*, the isolates in question did not harbor any relevant resistance genes. Even the penicillinase operon (*blaZ/blaR/blaI*) was absent, although it can most commonly be found in *S. aureus* from various sources, including CC692 where it is integrated into the beta-haemolysin-integrating “bird-specific” (D0K6J8 and D0K6J9) prophage. This might indicate that the strains in question emerged in wild waterfowl rather than from a farming environment, so they were not exposed to selective pressure by antimicrobial agents. Nevertheless, isolates carried SCC elements without *mecA/C.* In two isolates, 15V8707 and 511525-22, it was the same as in the C133 reference sequence. In the others, it carried genes, including *ccrAA/ccrC* homologs, that resemble genes found in *Mammaliicoccus* and in CC398 livestock-associated MRSA. While it should not be speculated here about the provenance and evolutionary history of these genes, it might be assumed that waterfowl also here serve as melting pots for the evolution of pathogens, and SCC/SCC*mec* elements, affecting birds, and various mammals, including pigs and humans. Anyway, JAFEOM01 and JAOAKU01 sequences show that chimeric waterfowl lineages also have the potential to evolve to MRSA by the acquisition of *mecA,* in this case, most likely as part of an SCC*mec* XIV element.

In summary, it can be stated that *S. aureus* is not a strictly “clonal” organism whose evolution is driven by gradual, time-dependent accumulation of single-point mutations plus occasional horizontal gene transfers by mobile genetic elements. There must be a further mechanism, albeit not yet understood, facilitating the horizontal exchange of large fragments of genomic DNA. Contrary to previous thought, large-scale genomic replacements in *S. aureus* cannot be considered exceedingly rare events but must be considered as a routine mechanism in this pathogen´s evolution (see also Introduction for additional examples), facilitating its adaptability and versatility. These replacements appear to be so common that even strains that originate from multiple transfer events can be identified. Undoubtedly, the rise of affordable sequencing technologies will lead to more such observations. The observed mechanism of large-scale genomic replacements is of significance for the evolutionary dynamics of *S. aureus,* as the examples listed in the introduction show. This mechanism represents a fundamental change from the conventional understanding to a more complex and dynamic evolutionary process. Such a mechanism serves as a robust strategy for rapidly incorporating diverse genetic material into the bacterial genome. Through the incorporation of sizable genetic fragments, *S. aureus* can acquire novel genes, virulence factors, and other adaptive elements from different lineages. This dynamic exchange is likely to be a potent driver of genetic diversity, allowing the bacterium to swiftly acquire traits that enhance its fitness within specific ecological niches. This includes its capacity to adapt to various hosts, as shown here, where a strain from small ruminants becomes an avian-specific strain by incorporation of DNA from another avian strain. The acquisition of genetic material from different lineages leads to the development of hybrid or chimeric strains with unique combinations of traits. In the context of *S. aureus* evolution, this mechanism allows the pathogen to potentially bridge gaps and jump between host species, enabling it to colonize and infect hosts that were previously less or not accessible. Consequently, such genetic exchanges could contribute to the emergence of novel strains with altered virulence, transmission patterns, and host range and need constant surveillance given the pathogenic potential of this species and the speed of bacterial evolution.

The issue of *S. aureus* in wild- and birdlife is still poorly understood. It is not even necessary to travel to distant countries sampling exotic wildlife to detect surprisingly interesting *S. aureus* lineages, as even common animal species, such as swans or domestic ducks, might harbor such strains. Here again, the new sequencing technologies combined with microarrays as a convenient tool for high-throughput screening might help to obtain more insight into the population structure and the evolution of that pathogen. The contribution of large-scale genomic replacements to host adaptation by *S. aureus* is particularly pertinent in the context of waterfowl. Due to their mobility and their long-range migratory habits, such birds could serve not only as a reservoir for a pathogen but also as vectors facilitating quick and distant dissemination. The relatedness of wild and domestic waterfowl and the close contact with humans and domestic animals of the latter might facilitate cross-species transmissions. The observation of chimeric strains comprising genomic material from different lineages associated with small ruminants and birds underscores the interconnectedness of bacterial populations across diverse host species and the dynamic nature of *S. aureus* evolution. Such strains might pose a risk for the emergence of novel livestock-associated strains (as the recent observation of chimeric CC133 × CC692 × CC522 MRSA in China shows, see above) as well as for possible zoonotic transmissions to humans. A better understanding of this mechanism could offer a perspective on how pathogens respond to selective pressures and adapt to changing environments and could provide insights into the emergence of virulent strains, antimicrobial resistance dynamics, and zoonotic events from a One Health perspective [31,32]. Further research into the genetic and functional consequences of large genomic DNA exchange will undoubtedly enhance our understanding of *S. aureus* as a versatile and adaptable pathogen.

## 5. Conclusions

In conclusion, we describe three distinct strains of *S. aureus* isolated from waterfowl that emerged from large genomic replacements involving CC133, CC522 and CC692. While the mechanism of horizontal transfers of large fragments of genomic DNA is not yet understood, it must be assumed that such events are rather common and that they play a role in the evolution and host adaption of this pathogen. As previously noted, D0K6J8/D0K6J9-prophages are associated with avian strains of *S. aureus*, being identified in four out of seven of these chimeric CC133 isolates. In conclusion, the vast and diverse avian population, coupled with, e.g., the sheer number of poultry, creates an environment where *S. aureus* can thrive and adapt, with the potential to bridge the gap between bird and human populations. The role of phages in facilitating genetic exchange further accentuates the significance of studying this phenomenon and its implications for One Health. The continuous coexistence and interaction of humans and domesticated poultry and waterfowl highlights the need for ongoing research to understand the dynamics of *S. aureus* transmission and adaptation.

## Figures and Tables

**Table 1:** Genes identified in the SCC element of the duck isolate V315 (and the related isolates).

Gene Symbol	Description	Most Similar Sequences in GenBank	Start Position in V315	End Position in V315	Orientation
*orfX*	23S rRNA methyltransferase		33,684	34,163	forward
N/A	hypothetical protein from *M*. *lentus* SCC	PN942_09245 from *M. lentus* PVZ.22 (CP116807.1: 1838230..1840569:RC)	34,220	36,580	forward
Q3T2M7	hypothetical protein	SAPIG0032 from *S. aureus* S0385 (AM990992: 38799..39170), SAT0131_02879 from *S. aureus* T0131 (CP002643: 2797995..2798366:RC)	34,220	38,189	forward
*ccrAA*	cassette chromosome recombinase homolog/”DUF5906 domain-containing protein”/DNA primase	PN942_09225 from *M*. *lentus* PVZ.22 (CP116807.1: 1833395..1834963:RC), HF021_12755 from *M. vitulinus* Tienloo1 (CP051882.1: 2616200..2617843:RC)	38,186	39,818	forward
*ccrC3*	hypothetical cassette chromosome recombinase C3/recombinase family protein	PN942_09220 from *M. lentus* PVZ.22 (CP116807.1: 1831493..1833169:RC), HF021_12750 from *M. vitulinus* Tienloo1 (CP051882.1: 2614298..2615974:RC)	40,048	41,724	forward
*sauGI*	SAUGI family uracil-DNA glycosylase inhibitor	PN942_09215 from *M. lentus* PVZ.22 (CP116807.1:1831038..1831388:RC)	41,829	42,179	forward
DUF1643	DUF1643 domain-containing putative membrane protein	I7830_05530 from *Mammaliicoccus sciuri* GDH8C110P (CP065795.1: 1064650..1065162)	42,595	43,107	forward
*hsdR*	type I restriction–modification system, endonuclease/restriction (R) subunit	SAMEA4384403_00041 from *Mammaliicoccus stepanovicii* NCTC13839 (LT906462.1:40905..44027:RC)	43,302	46,424	reverse
*hsdS*	type I restriction–modification system, site-specificity determinate (S subunit)	SAMEA4384403_00042 from *M. stepanovicii* NCTC13839 (LT906462.1:44011..45285:RC)	46,408	47,682	reverse
*hsdM*	type I restriction–modification system, DNA methylase (M) subunit	SAMEA4384403_00043 from *M. stepanovicii* NCTC13839 (LT906462.1:45275..46789:RC)	47,672	49,186	reverse

**Table 2:** Prophage carriage in the study strains.

Localization (Gene Name)	Localization (cg MLST)	Strain ED133, CP001996	Strain X22, CP042650	Swan Isolate 15V8707	Duck Isolate V315	Duck Isolate V482	Goat Isolate 17CS1042	Comments
Between *lip2* and A5IPQ2	SAUR0317 (SAR_RS01585), SAUR0318 (SAR_RS01590)	Positive (ca. 47,400 nt)	Positive (ca. 41,500 nt)	Negative	Negative	Negative	Negative	-
Between *sufB* and MW0800	SAUR0895 (SAR_RS04475), SAUR0896 (SAR_RS04480):	Negative	Positive (ca. 46,600 nt)	Negative	Negative	Negative	Negative	-
Between *rpmF* and *isdB*	SAUR1124 (SAR_RS05620), SAUR1125 (SAR_RS05625)	Positive (ca. 40,500 nt)	Negative	Negative	Negative	Negative	Positive (ca. 43,700 nt)	In ED133, the phage in this position carried a variant of the Enterotoxin A gene, *sea-*320E (GenBank: AY196686.1).
Between Q7A4X2 = DUF1828 and *ydeN*	N/A, and SAUR1973 (SAR_RS09865)	Incomplete (ca. 1600 nt)	Incomplete (ca. 1600 nt)	Positive (ca. 45,700 nt)	Incomplete (ca. 1600 nt)	Positive (ca. 42,800 nt)	Incomplete (ca. 1600 nt)	“Incomplete” means that there were only genes for Q99T51, a “putative DUF2871 domain-containing protein” and a phage integrase.
Between Q5HEP8 and A5IU43 = *yfkAB*	SAUR2053 (SAR_RS10265), SAUR2054 (SAR_RS10270)	Positive (ca. 44,700 nt)	Negative	Negative	Negative	Negative	Positive (ca. 40,600 nt)	The prophage in ED133 carried leukocidin genes *lukM/lukF-*P83, while the one in 17CS1042 harbored a variant thereof.
Within *hlb*	SAUR2190 (SAR_RS10950)	Negative	Positive (ca. 52,100 nt)	Positive * (ca. 62,800 nt)	Positive ** (ca. 45,700 nt)	Negative	Negative	Prophages in this position carried “bird-specific” virulence factors D0K6J8 and D0K6J9.

* The Austrian duck isolate 511525-22 was also positive, with the same prophage as in 15V8707 (see Supplemental Files S4a–c). ** IMT40427 was also positive, with the same prophage as in V315 (see Supplemental Files S4a–c), but 511509-22 and 511510-22 were negative.

## Data Availability

All relevant data are provided as Supplementary Files. The sequences of the ONT sequenced genomes discussed are submitted to GenBank. The BioProject ID is PRJNA1036839, the BioSample accession numbers are SAMN38153147, SAMN38153148, SAMN38153149 and SAMN38153150. The GenBank accession numbers are CP138360.1, CP138361.1, CP138362.1 and CP138363.1.

## Supplementary Materials

The following supporting information can be downloaded at: https://www.mdpi.com/article/10.3390/microorganisms12010096/s1, Supplemental File S1a: Nanopore sequence of the mute swan isolate 15V8707. Supplemental File S1b: Illumina sequence of the duck isolate 511525-22. Supplemental File S1c: Nanopore sequence of the duck isolate V315. Supplemental File S1d: Illumina sequence of the duck isolate 511509-22. Supplemental File S1e: Illumina sequence of the duck isolate 511510-22. Supplemental File S1f: Illumina sequence of the whistling duck isolate IMT40427. Supplemental File S1g: Nanopore sequence of the duck isolate_V482. Supplemental File S1h: Nanopore sequence of the goat isolate 17CS1042. Supplemental File S2: cgMLST data for the study strains and comparison to other lineages (Yellow, regions of CC133 origin; purple, regions of CC692 origin; brown, regions of CC522 origin. Positions of undeterminable origin in grey). Supplemental File S3a: Alignments of markers used for construction of Figure 1 and Figure 2. Supplemental File S3b: Numerical values used for construction of Figure 1, Figure 3 and Figure 5 (with differences between sequences color-coded as heatmap: pale yellow, identical sequences, to dark red, with differences ≥ 5%). Supplemental File S4a: Sequences of the avian prophages in study isolates and reference sequences, displayed with the integrase gene first. Supplemental File S4b: Alignment of the avian prophages in study isolates and reference sequences, displayed with the integrase gene first. Supplemental File S4c: Annotation of the avian prophages in study isolates and reference sequences.

## References

[1] Loncaric I., Stalder G.L., Mehinagic K., Rosengarten R., Hoelzl F., Knauer F., Walzer C. (2013). Comparison of ESBL—And AmpC producing *Enterobacteriaceae* and methicillin-resistant *Staphylococcus aureus* (MRSA) isolated from migratory and resident population of rooks (*Corvus frugilegus*) in Austria. PLoS ONE.

[2] Gómez P., Lozano C., Camacho M.C., Lima-Barbero J.F., Hernandez J.M., Zarazaga M., Hofle U., Torres C. (2016). Detection of MRSA ST3061-t843-*mecC* and ST398-t011-*mecA* in white stork nestlings exposed to human residues. J. Antimicrob. Chemother..

[3] Monecke S., Gavier-Widén D., Hotzel H., Peters M., Guenther S., Lazaris A., Loncaric I., Müller E., Reissig A., Ruppelt-Lorz A. (2016). Diversity of *Staphylococcus aureus* Isolates in European Wildlife. PLoS ONE.

[4] Ruiz-Ripa L., Gómez P., Alonso C.A., Camacho M.C., de la Puente J., Fernández-Fernández R., Ramiro Y., Quevedo M.A., Blanco J.M., Zarazaga M. (2019). Detection of MRSA of Lineages CC130-*mecC* and CC398-*mecA* and *Staphylococcus delphini*-*lnu*(A) in Magpies and Cinereous Vultures in Spain. Microb. Ecol..

[5] Abdullahi I.N., Juárez-Fernández G., Höfle U., Latorre-Fernández J., Cardona-Cabrera T., Mínguez-Romero D., Zarazaga M., Lozano C., Torres C. (2023). *Staphylococcus aureus* Carriage in the Nasotracheal Cavities of White Stork Nestlings (*Ciconia ciconia*) in Spain: Genetic Diversity, Resistomes and Virulence Factors. Microb. Ecol..

[6] Silva V., Lopes A.F., Soeiro V., Caniça M., Manageiro V., Pereira J.E., Maltez L., Capelo J.L., Igrejas G., Poeta P. (2022). Nocturnal Birds of Prey as Carriers of *Staphylococcus aureus* and Other Staphylococci: Diversity, Antimicrobial Resistance and Clonal Lineages. Antibiotics.

[7] Lowder B.V., Guinane C.M., Ben Zakour N.L., Weinert L.A., Conway-Morris A., Cartwright R.A., Simpson A.J., Rambaut A., Nübel U., Fitzgerald J.R. (2009). Recent human-to-poultry host jump, adaptation, and pandemic spread of *Staphylococcus aureus*. Proc. Natl. Acad. Sci. USA.

[8] Chen M.M.S., Monecke S., Brown M.H. (2016). Clonal diversity of methicillin-sensitive *Staphylococcus aureus* from South Australian wallabies. One Health.

[9] Lee G.Y., Lee S.I., Kim S.D., Park J.H., Kim G.B., Yang S.J. (2022). Clonal distribution and antimicrobial resistance of methicillin-susceptible and -resistant *Staphylococcus aureus* strains isolated from broiler farms, slaughterhouses, and retail chicken meat. Poult. Sci..

[10] Moon D.C., Kim B.Y., Tamang M.D., Nam H.M., Jang G.C., Jung S.C., Lee H.S., Park Y.H., Lim S.K. (2016). Genome Sequence of a Unique t2247-ST692-III Livestock-Associated Methicillin-Resistant *Staphylococcus aureus* Strain from Chicken Carcass. Genome Announc..

[11] Vanderhaeghen W., Hermans K., Haesebrouck F., Butaye P. (2010). Methicillin-resistant *Staphylococcus aureus* (MRSA) in food production animals. Epidemiol. Infect..

[12] Feßler A.T., Kadlec K., Hassel M., Hauschild T., Eidam C., Ehricht R., Monecke S., Schwarz S. (2011). Characterization of methicillin-resistant *Staphylococcus aureus* isolates from food and food products of poultry origin in Germany. Appl. Environ. Microbiol..

[13] Price L.B., Stegger M., Hasman H., Aziz M., Larsen J., Andersen P.S., Pearson T., Waters A.E., Foster J.T., Schupp J. (2012). *Staphylococcus aureus* CC398: Host adaptation and emergence of methicillin resistance in livestock. mBio.

[14] Monecke S., Ruppelt A., Wendlandt S., Schwarz S., Slickers P., Ehricht R., Jackel S.C. (2013). Genotyping of *Staphylococcus aureus* isolates from diseased poultry. Vet. Microbiol..

[15] Ye X., Wang X., Fan Y., Peng Y., Li L., Li S., Huang J., Yao Z., Chen S. (2016). Genotypic and Phenotypic Markers of Livestock-Associated Methicillin-Resistant *Staphylococcus aureus* CC9 in Humans. Appl. Environ. Microbiol..

[16] Gonçalves da Silva A., Baines S.L., Carter G.P., Heffernan H., French N.P., Ren X., Seemann T., Bulach D., Kwong J., Stinear T.P. (2017). A phylogenomic framework for assessing the global emergence and evolution of clonal complex 398 methicillin-resistant *Staphylococcus aureus*. Microb. Genom..

[17] Wang W., Liu F., Baloch Z., Zhang C.S., Ma K., Peng Z.X., Yan S.F., Hu Y.J., Gan X., Dong Y.P. (2017). Genotypic Characterization of Methicillin-resistant *Staphylococcus aureus* Isolated from Pigs and Retail Foods in China. Biomed. Environ. Sci..

[18] Abd El-Ghany W.A. (2021). *Staphylococcus aureus* in poultry, with special emphasis on methicillin-resistant strain infection: A comprehensive review from one health perspective. Int. J. One Health.

[19] Li X., Li G., Huang H., Wan P., Lu Y., Li Z., Xie L., Xiong W., Zeng Z. (2023). The occurrence and contamination of *optrA-*positive methicillin-resistant *Staphylococcus aureus* from duck farms in Guangdong, China. J. Glob. Antimicrob. Resist..

[20] Li Y., Li W., Pan Y., Liu C., Liang S., Zeng Z. (2022). The emergence and molecular study of methicillin-resistant *Staphylococcus aureus* ST239, ST59, ST9, and ST630 in food animals, Chongqing, China. Vet. Microbiol..

[21] Zhang T., Jia M., Cheng Y., Zhang W., Lu Q., Guo Y., Wen G., Shao H., Luo Q. (2021). First report of ST9-MRSA-XII from a chicken farm in China. J. Glob. Antimicrob. Resist..

[22] Robinson D.A., Enright M.C. (2004). Evolution of *Staphylococcus aureus* by large chromosomal replacements. J. Bacteriol..

[23] Thomas J.C., Godfrey P.A., Feldgarden M., Robinson D.A. (2012). Draft genome sequences of *Staphylococcus aureus* sequence type 34 (ST34) and ST42 hybrids. J. Bacteriol..

[24] Gawlik D., Ruppelt-Lorz A., Muller E., Reissig A., Hotzel H., Braun S.D., Soderquist B., Ziegler-Cordts A., Stein C., Pletz M.W. (2020). Molecular investigations on a chimeric strain of *Staphylococcus aureus* sequence type 80. PLoS ONE.

[25] Spoor L.E., Richardson E., Richards A.C., Wilson G.J., Mendonca C., Gupta R.K., McAdam P.R., Nutbeam-Tuffs S., Black N.S., O’Gara J.P. (2015). Recombination-mediated remodelling of host–pathogen interactions during *Staphylococcus aureus* niche adaptation. Microb. Genom..

[26] Fetsch A., Kraushaar B., Kasbohrer A., Hammerl J.A. (2017). Turkey Meat as Source of CC9/CC398 Methicillin-Resistant *Staphylococcus aureus* in Humans?. Clin. Infect. Dis..

[27] Strommenger B., Braulke C., Heuck D., Schmidt C., Pasemann B., Nübel U., Witte W. (2008). *Spa* Typing of *Staphylococcus aureus* as a Frontline Tool in Epidemiological Typing. J. Clin. Microbiol..

[28] Monecke S., Coombs G., Shore A.C., Coleman D.C., Akpaka P., Borg M., Chow H., Ip M., Jatzwauk L., Jonas D. (2011). A field guide to pandemic, epidemic and sporadic clones of methicillin-resistant *Staphylococcus aureus*. PLoS ONE.

[29] Nimmo G.R., Steen J.A., Monecke S., Ehricht R., Slickers P., Thomas J.C., Appleton S., Goering R.V., Robinson D.A., Coombs G.W. (2015). ST2249-MRSA-III: A second major recombinant methicillin-resistant *Staphylococcus aureus* clone causing healthcare infection in the 1970s. Clin. Microbiol. Infect..

[30] Burgold-Voigt S., Monecke S., Simbeck A., Holzmann T., Kieninger B., Liebler-Tenorio E.M., Braun S.D., Collatz M., Diezel C., Müller E. (2021). Characterisation and molecular analysis of an unusual chimeric methicillin resistant *Staphylococcus aureus* strain and its bacteriophages. Front. Genet..

[31] Adisasmito W.B., Almuhairi S., Behravesh C.B., Bilivogui P., Bukachi S.A., Casas N., Cediel Becerra N., Charron D.F., Chaudhary A., Ciacci Zanella J.R. (2022). One Health: A new definition for a sustainable and healthy future. PLoS Pathog..

[32] Mackenzie J.S., Jeggo M. (2019). The One Health Approach—Why Is It So Important?. Trop. Med. Infect. Dis..

[33] Monecke S., Slickers P., Ehricht R. (2008). Assignment of *Staphylococcus aureus* isolates to clonal complexes based on microarray analysis and pattern recognition. FEMS Immunol. Med. Microbiol..

[34] Monecke S., Jatzwauk L., Müller E., Nitschke H., Pfohl K., Slickers P., Reissig A., Ruppelt-Lorz A., Ehricht R. (2016). Diversity of SCC*mec* elements in *Staphylococcus aureus* as observed in South-Eastern Germany. PLoS ONE.

[35] Scholtzek A.D., Hanke D., Walther B., Eichhorn I., Stöckle S.D., Klein K.S., Gehlen H., Lübke-Becker A., Schwarz S., Feßler A.T. (2019). Molecular Characterization of Equine *Staphylococcus aureus* Isolates Exhibiting Reduced Oxacillin Susceptibility. Toxins.

[36] Jolley K.A., Bray J.E., Maiden M.C.J. (2018). Open-access bacterial population genomics: BIGSdb software, the PubMLST.org website and their applications. Wellcome Open Res..

[37] Wan T.W., Khokhlova O.E., Iwao Y., Higuchi W., Hung W.C., Reva I.V., Singur O.A., Gostev V.V., Sidorenko S.V., Peryanova O.V. (2016). Complete Circular Genome Sequence of Successful ST8/SCC*mecI*V Community-Associated Methicillin-Resistant *Staphylococcus aureus* (OC8) in Russia: One-Megabase Genomic Inversion, IS256’s Spread, and Evolution of Russia ST8-IV. PLoS ONE.

[38] Deorowicz S., Debudaj-Grabysz A., Gudyś A. (2016). FAMSA: Fast and accurate multiple sequence alignment of huge protein families. Sci. Rep..

[39] Monecke S., Schaumburg F., Shittu A.O., Schwarz S., Mühldorfer K., Brandt C., Braun S.D., Collatz M., Diezel C., Gawlik D. (2022). Description of Staphylococcal Strains from Straw-Coloured Fruit Bat (*Eidolon helvum*) and Diamond Firetail (*Stagonopleura guttata*) and a Review of their Phylogenetic Relationships to Other Staphylococci. Front. Cell. Infect. Microbiol..

[40] Sargata-Vicens J., del Hoyo J., Elliot A., Imboden C. (1992). Handbook of the Birds of the World: Ostrich to Ducks.

[41] Thapaliya D., Dalman M., Kadariya J., Little K., Mansell V., Taha M.Y., Grenier D., Smith T.C. (2017). Characterization of *Staphylococcus aureus* in Goose Feces from State Parks in Northeast Ohio. Ecohealth.

[42] Achek R., El-Adawy H., Hotzel H., Tomaso H., Ehricht R., Hamdi T.M., Azzi O., Monecke S. (2020). Short communication: Diversity of staphylococci isolated from sheep mastitis in northern Algeria. J. Dairy Sci..

[43] Smith E.M., Needs P.F., Manley G., Green L.E. (2014). Global distribution and diversity of ovine-associated *Staphylococcus aureus*. Infect. Genet. Evol..

[44] Bar-Gal G.K., Blum S.E., Hadas L., Ehricht R., Monecke S., Leitner G. (2015). Host-specificity of *Staphylococcus aureus* causing intramammary infections in dairy animals assessed by genotyping and virulence genes. Vet. Microbiol..

[45] Gharsa H., Ben Sallem R., Ben Slama K., Gomez-Sanz E., Lozano C., Jouini A., Klibi N., Zarazaga M., Boudabous A., Torres C. (2012). High diversity of genetic lineages and virulence genes in nasal *Staphylococcus aureus* isolates from donkeys destined to food consumption in Tunisia with predominance of the ruminant associated CC133 lineage. BMC Vet. Res..

