# Supplementary material for: Molecular Characterization of Chimeric Staphylococcus aureus Strains from Waterfowl
Source: Microorganisms. 2024 Jan 3;12(1):96. doi: 10.3390/microorganisms12010096 (PMC10821479; doi:10.3390/microorganisms12010096)
Supplement: Supplementary file 1 [file microorganisms-12-00096-s001.zip › Supplemental File 2_cgMLST data for the study strains and comparison to other lineages_2023-12-12.pdf]

Locus (cg MLST)	Alternative designation	Position (in ED133)	Study Isolates							CC133			CC692				CC522					ST1347	Chinese CC133 MRSA isolates		CC5	ST71	CC182	CC707	CC1956
			Swan isolate 15V8707	Duck isolate 511525-22	Duck isolate V315	Duck isolate 511509-22	Duck isolate 511510-22	Whistling duck isolate [illegible]	Duck isolate V482	CP001996.1, ED133	CP065353.1, AR39 [illegible]	LR134271.1, NCTC7988	CP042650.1, X22	UHAK01000002.1, NCTC9546	PubMLST ID 39948, 973N0056	CP129360, CKMM-401M	Goat isolate 17CS1042	PubMLST ID 24977	PubMLST ID 24358	PubMLST ID 11704	SAMEA1904171	PubMLST ID 39945, 61290	JAOAKU01, GDQ20D75A	JAFEOM01, GDQ20D75A	CP001781.1, ED98	SAMN03247575, MOK-099	CP070991, CBS2016-05	CP029751, SMITH	CP084892, WT19
SAUR0001 (SAR_RS00005)	dnaA	517	389	389	12	12	12	12	N/A	76	76	76	12	12	12	12	92	92	92	92	92	372	12	12	7	77	121	152	13
SAUR0002 (SAR_RS00010)	dnaN	2158	9	N/A	9	9	9	9	N/A	106	2	2	N/A	9	9	N/A	119	272	119	119	119	119	9	9	8	93	143	178	N/A
SAUR0003 (SAR_RS00015)	N/A	3681	1	1	1	1	1	1	1	3	3	3	1	1	1	1	3	3	3	3	3	3	1	1	1	3	3	2	3
SAUR0004 (SAR_RS00020)	recF	3914	7	7	7	7	7	7	7	71	71	71	N/A	7	7	N/A	88	88	88	88	88	88	7	7	6	76	2	2	89
SAUR0005 (SAR_RS00025)	gyrB	5036	170	170	170	170	170	170	170	111	111	111	170	170	170	170	137	137	137	137	137	137	170	170	10	118	172	225	139
SAUR0006 (SAR_RS00030)	N/A	7007	608	N/A	N/A	N/A	N/A	N/A	N/A	116	116	116	N/A	N/A	N/A	N/A	138	336	138	138	138	N/A	N/A	N/A	15	115	178	225	N/A
SAUR0007 (SAR_RS00035)	nnrD	9755	255	255	9	9	9	9	9	20	20	20	9	9	9	9	N/A	20	20	20	20	N/A	20	20	8	54	54	104	N/A
SAUR0008 (SAR_RS00040)	hutH	10893	100	100	173	N/A	N/A	173	N/A	100	100	100	173	173	173	173	139	139	139	139	139	N/A	173	173	11	107	20	217	140
SAUR0009 (SAR_RS00045)	serS	12786	81	81	41	41	41	41	N/A	81	81	81	41	41	41	41	36	36	36	36	36	41	81	81	9	86	108	157	44
SAUR0010 (SAR_RS00050)	azlC	14726	82	82	119	119	119	119	119	82	82	82	119	119	348	119	200	2	2	2	2	347	82	82	4	83	2	147	104
SAUR0011 (SAR_RS00055)	azlD	15418	52	52	N/A	N/A	N/A	N/A	N/A	52	52	52	4	4	N/A	4	65	65	N/A	65	65	4	52	52	9	24	24	91	24
SAUR0012 (SAR_RS00060)	metX	16110	423	423	151	151	151	151	151	40	100	40	151	151	151	151	N/A	52	52	52	52	151	N/A	N/A	142	43	67	78	53
SAUR0013 (SAR_RS00065)	N/A	17369	N/A	41	11	11	11	11	11	41	41	41	11	11	377	11	88	88	153	88	88	11	N/A	N/A	8	76	118	155	N/A
SAUR0014 (SAR_RS00070)	gdpP	18322	172	172	176	176	176	176	176	172	327	172	176	176	176	N/A	148	148	357	148	148	176	172	172	643	159	148	252	149
SAUR0015 (SAR_RS00075)	rplI	20286	45	45	4	4	4	4	4	45	45	45	4	4	4	4	3	3	3	3	3	4	45	45	8	3	11	6	3
SAUR0016 (SAR_RS00080)	dnaC	20764	100	100	166	N/A	N/A	166	166	100	100	109	166	166	166	166	129	129	129	129	207	166	N/A	N/A	11	107	135	211	130
SAUR0017 (SAR_RS00085)	purA	22443	30	30	111	111	111	111	111	30	30	30	111	111	111	111	N/A	92	92	92	92	111	30	30	9	77	96	137	93
SAUR0020 (walR)	N/A	24921	47	47	67	67	67	67	N/A	47	47	47	67	67	67	67	56	56	56	56	56	67	47	47	7	2	58	58	20
SAUR0021 (walK)	N/A	25635	147	147	519	519	519	519	N/A	147	147	147	519	519	519	519	182	182	182	182	182	519	147	147	75	151	647	221	N/A
SAUR0022 (SAR_RS00110)	walR=vycH	27454	591	591	N/A	N/A	N/A	N/A	N/A	104	303	N/A	N/A	175	175	175	132	132	132	132	132	N/A	N/A	N/A	11	109	176	241	133
SAUR0023 (SAR_RS00115)	walI=vycI	28789	51	51	86	86	86	86	86	247	51	51	86	86	86	86	69	69	69	69	69	86	51	51	26	N/A	55	109	71
SAUR0024 (SAR_RS00120)	walJ	29965	13	13	86	86	86	86	86	13	13	13	86	86	86	86	27	27	27	27	27	86	13	13	8	27	94	108	72
SAUR0025 (SAR_RS00125)	sasH=adsA	30992	61	61	313	N/A	N/A	313	N/A	61	61	61	313	313	N/A	N/A	N/A	115	115	78	115	313	61	61	235	N/A	563	117	N/A
SAUR0026 (SAR_RS00130)	orfX	33678	140	140	N/A	N/A	N/A	N/A	N/A	140	140	140	N/A	458	189	N/A	172	172	172	172	172	189	N/A	N/A	33	144	268	225	173
SAUR0106 (SAR_RS00530)	DUF1648	69297	210	210	63	63	63	63	63	2	2	2	N/A	63	63	N/A	2	2	2	2	2	63	2	2	6	40	40	2	15
SAUR0107 (SAR_RS00535)	lctP-locus1	70032	72	72	N/A	164	164	164	164	494	72	72	164	164	164	164	127	N/A	127	127	127	497	72	72	412	100	40	72	N/A
SAUR0109 (SAR_RS00545)	N/A	73948	23	23	199	199	199	199	199	101	23	23	199	199	199	199	105	105	105	61	105	199	23	23	11	23	78	108	63
SAUR0110 (SAR_RS00550)	sirC	75069	66	66	N/A	N/A	N/A	N/A	2	66	66	66	N/A	2	2	N/A	N/A	2	2	2	2	2	66	66	334	71	122	164	N/A
SAUR0111 (SAR_RS00555)	sirB	76064	9	9	122	122	122	122	122	9	196	9	N/A	396	122	122	92	92	92	92	92	122	N/A	N/A	21	74	14	159	93
SAUR0112 (SAR_RS00560)	sirA	77075	63	63	106	106	106	106	106	63	63	63	106	106	106	106	84	84	84	84	84	312	63	63	9	68	89	89	25
SAUR0113 (SAR_RS00565)	sbnA=cysK/L1	78298	53	53	92	92	92	92	92	53	53	53	92	92	92	92	3	3	171	3	3	92	53	53	7	3	93	127	72
SAUR0114 (SAR_RS00570)	sbnB	79275	31	31	93	93	93	93	93	31	31	31	93	93	93	93	80	80	80	80	80	93	N/A	N/A	8	64	94	130	N/A
SAUR0115 (SAR_RS00575)	sbnC	80306	112	112	N/A	N/A	N/A	N/A	N/A	517	112	112	307	307	307	307	117	117	117	117	117	307	344	344	11	117	188	236	146
SAUR0116 (SAR_RS00580)	sbnD	82053	101	101	154	154	154	154	154	101	101	101	154	154	154	154	197	267	267	128	197	N/A	101	101	12	105	2	201	129
SAUR0117 (SAR_RS00585)	sbnE	83299	367	367	180	180	180	180	N/A	105	105	105	180	180	180	180	142	142	142	142	142	N/A	105	105	30	N/A	50	237	144
SAUR0118 (SAR_RS00590)	N/A	85016	109	109	N/A	N/A	N/A	N/A	N/A	548	315	N/A	189	189	189	189	149	149	149	149	149	189	109	109	10	116	2	253	N/A
SAUR0119 (SAR_RS00595)	sbnG	86769	2	2	N/A	87	87	87	87	2	2	2	87	87	87	87	57	57	57	57	57	N/A	2	2	298	57	2	119	2
SAUR0120 (SAR_RS00600)	sbnH	87545	88	88	N/A	142	142	142	N/A	88	88	88	N/A	442	142	N/A	117	117	117	117	117	142	N/A	N/A	8	92	145	187	118
SAUR0121 (SAR_RS00605)	sbnI	88751	40	40	16	N/A	N/A	16	16	274	40	40	16	207	207	16	77	77	77	77	77	279	40	40	8	16	37	119	16
SAUR0122 (SAR_RS00610)	N/A	N/A	N/A	N/A	8	8	8	8	8	N/A	N/A	N/A	8	8	8	8	21	21	21	21	21	N/A	N/A	N/A	2	2	N/A	N/A	2
SAUR0124 (SAR_RS00620)	butA	91193	68	68	8	8	8	8	8	68	68	68	8	8	8	8	89	89	89	89	89	8	68	68	8	74	117	145	90
SAUR0125 (SAR_RS00625)	N/A	92113	3	3	4	4	4	4	4	3	N/A	3	4	4	4	8	N/A	N/A	N/A	N/A	N/A	N/A	3	3	4	3	N/A	N/A	3
SAUR0126 (SAR_RS00630)	galE	92314	2	2	100	100	100	100	100	2	2	2	N/A	100	100	N/A	80	80	80	80	80	100	N/A	N/A	7	N/A	101	N/A	N/A
SAUR0127 (SAR_RS00635)	wcaJ=tuaA	93248	2	2	N/A	N/A	N/A	N/A	N/A	2	2	2	94	292	94	94	33	33	33	33	33	N/A	2	2	90	2	36	52	2
SAUR0128 (SAR_RS00640)	epsF	94150	2	2	147	147	147	147	147	2	236	N/A	N/A	512	147	147	123	123	123	123	123	N/A	2	2	8	2	156	112	N/A
SAUR0129 (SAR_RS00645)	wzy	95297	471	471	19	19	19	19	19	103	103	103	N/A	489	19	N/A	N/A	82	275	82	82	N/A	103	103	39	N/A	29	208	139
SAUR0130 (SAR_RS00650)	wzx	96525	157	157	374	374	374	374	374	110	279	110	374	374	374	N/A	216	216	216	133	216	374	N/A	N/A	7	108	159	217	N/A
SAUR0131 (SAR_RS00655)	sodA-L1	N/A	N/A	N/A	46	N/A	46	46	46	N/A	35	35	46	46	144	46	N/A	34	34	34	34	46	35	35	6	34	2	34	183
SAUR0133 (SAR_RS00665)	OSHIN3	99256	51	51	88	88	88	88	88	51	51	51	88	88	88	88	149	14	14	14	14	88	51	51	11	14	14	14	23
SAUR0134 (SAR_RS00670)	N/A	100114	12	12	56	56	56	56	56	12	12	12	56	56	56	56	60	60	60	60	60	N/A	12	12	5	2	79	12	12
SAUR0135 (SAR_RS00675)	deoD-L1	100247	13	13	6	6	6	6	6	13	13	13	6	76	76	6	78	78	78	78	78	6	13	13	6	N/A	97	63	49
SAUR0136 (SAR_RS00680)	tet38	100961	447	447	306	306	306	306	N/A	88	88	88	N/A	306	306	N/A	174	254	174	107	174	306	88	88	7	94	111	177	2
SAUR0138 (SAR_RS00690)	deoB	103084	395	395	112	112	112	112	112	64	64	64	112	112	112	112	89	89	89	89	89	112	N/A	N/A	7	72	92	27	27
SAUR0139 (SAR_RS00695)	phnE2	104392	76	76	N/A	N/A	N/A	N/A	N/A	76	76	145	123	123	123	123	96	96	96	96	96	123	76	76	10	29	127	2	2
SAUR0140 (SAR_RS00700)	phnE1	105204	2	2	N/A	N/A	N/A	N/A	N/A	71	2	71	22	22	22	22	2	2	188	2	2	22	2	2	8	57	113	2	2
SAUR0141 (SAR_RS00705)	phnC	106006	378	378	120	120	120	120	120	37	37	37	120	120	120	120	N/A	219	155	95	155	120	37	37	298	60	100	157	96
SAUR0142 (SAR_RS00710)	phnB	106993	74	74	12	12	12	12	12	74	74	74	12	12	12	12	102	102	102	102	102	12	74	74	6	55	25	161	N/A
SAUR0143 (SAR_RS00715)	OSHJM4	108178	116	116	24	24	24	N/A	N/A	116	116	116	N/A	708	24	N/A	367	116	116	116	116	N/A	N/A	N/A	9	122	197	262	161
SAUR0144 (SAR_RS00720)	codB	109773	144	144	N/A	N/A	N/A	N/A	N/A	108	144	108	N/A	184	184	N/A	144	144	144	144	144	605	144	144	499	114	186	231	146
SAUR0147 (SAR_RS00735)	adhE	113799	895	895	N/A	N/A	N/A	N/A	N/A	144	144	144	N/A	926	251	N/A	198	518	198	198	198	251	144	144	10	148	778	324	N/A
SAUR0148 (SAR_RS00740)	capA-L1	116753	2	2	83	83	83	83	2	2	2	2	83	N/A	262	83	69	69	69	69	69	83	2	2	7	58	2	2	2
SAUR0149 (SAR_RS00745)	capB-L1	117437	2	2	N/A	115	115	115	2	2	2	2	115	115	115	115	2	2	2	2	2	115	2	2	15	49	49	2	49
SAUR0150 (SAR_RS00750)	capC-L1	118126	28	28	154	154	154	154	N/A	28	28	28	N/A	154	154	154	28	28	28	28	28	274	28	28	229	32	97	121	N/A
SAUR0151 (SAR_RS00755)	capD	118910	N/A	N/A	N/A	N/A	N/A	N/A	N/A	147	147	147	N/A	222	740	222	N/A	188	188	188	188	N/A	N/A	N/A	16	N/A	225	289	2
SAUR0152 (SAR_RS00760)	capE	120723	84	84	11	N/A	N/A	11	84	84	84	84	11	11	11	11	105	105	105	105	105	N/A	84	84	11	86	2	164	N/A
SAUR0153 (SAR_RS00765)	capF	121764	95	95	154	154	154	154	95	95	95	95	154	154	154	N/A	116	116	116	116	116	154	95	95	11	98	157	193	2
SAUR0154 (SAR_RS00770)	capG	122877	88	88	N/A	N/A	N/A	N/A	N/A	88	88	88	390	129	129	390	102	242	102	102	102	391	88	88	9	43	43	155	2
SAUR0159 (SAR_RS00795)	capL	128305	543	543	155	N/A	N/A	155	155	89	89	89	155	155	155	155	125	125	125	125	125	155	N/A	N/A	12	97	158	205	126
SAUR0160 (SAR_RS00800)	capM	129521	217	217	6	6	6	6	6	44	44	44	6	6	6	6	58	58	58	58	58	6	44	44	6	39	N/A	59	59
SAUR0161 (SAR_RS00805)	capN	130078	72	72	120	120	120	120	120	72	72	72	N/A	120	120	120	92	92	92	92	92	120	72	72	9	77	122	N/A	N/A
SAUR0162 (SAR_RS00810)	capO	131019	102	102	162	162	162	162	162	102	102	102	N/A	162	162	162	2	2	2	2	2	162	102	102	8	109	163	213	2
SAUR0163 (SAR_RS00815)	capP	132358	105	105	160	160	160	160	N/A	105	105	105	160	160	160	160	134	134	134	134	201	N/A	105	105	6	109	138	203	2
SAUR0164 (SAR_RS00820)	isdI	133568	17	17	3	3	3	3	3	17	17	17	138	3	3	138	17	82	17	17	17	3	17	17	19	1	46	17	1
SAUR0165 (SAR_RS00825)	vbaN	133901	1	1	7	7	7	7	7	1	1	1	7	7	7	7	1	1	1	1	1	7	N/A	N/A	4	43	1	1	1
SAUR0166 (SAR_RS00830)	aldA1	134711	34	34	168	168	168	168	168	34	34	34	168	168	168	168	139	139	139	139	139	168	34	34	9	148	170	218	N/A
SAUR0167 (SAR_RS00835)	czcD	136846	16	16	110	110	110	110	110	16	16	16	362	110	110	362	88	88	88	88	88	110	16	16	8	74	91	136	N/A
SAUR0168 (SAR_RS00840)	N/A	137866	15	15	6	6	6	6	6	15	15	15	6	6	6	6	23	23	23	23	23	6	N/A	N/A	5	19	15	15	15
SAUR0169 (SAR_RS00845)	OSHJK0=sroF	138248	10	10	4	4	4	4	4	10	10	10	4	4	4	4	10	10	10	10	10	4	10	10	5	65	72	60	N/A
SAUR0170 (SAR_RS00850)	tauB	139101	354	354	191	191	191	191	191	25	25	25	191	191	191	191	25	25	25	25	25	N/A	N/A	N/A	30	25	305	148	25
SAUR0171 (SAR_RS00855)	tauA	139855	N/A	N/A	276	276	276	276	276	86	265	86	276	276	276	276	131	131	131	131	206	N/A	N/A	N/A	33	105	158	210	72
SAUR0172 (SAR_RS00860)	tauC	140826	69	69	7	7	7	7	7	69	196	69	7	7	7	7	215	150	150	96	150	374	69	69	38	74	120	153	97
SAUR0173 (SAR_RS00865)	N/A	N/A	N/A	N/A	305	305	305	305	305	N/A	N/A	N/A	305	305	305	305	93	42	N/A	42	42	N/A	N/A	N/A	121	37	44	68	N/A
SAUR0176 (SAR_RS00880)	lmrP	143083	509	509	N/A	112	112	N/A	112	331	326	331	112	112	112	112	N/A	377	375	340	340	N/A	326	326	4	N/A	450	359	N/A
SAUR0177 (SAR_RS00885)	ausA	144780	2251	2251	N/A	N/A	N/A	N/A	2208	2008	336	336	N/A	N/A	2207	N/A	835	1327	1218	490	835	N	N/A	N/A	23	634	2003	843	N/A
SAUR0178 (SAR_RS00890)	ausB	151968	17	17	144	144	144	144	144	17	17	17	144	297	144	144	76	159	76	76	76	144	17	17	8	17	90	17	N/A
SAUR0179 (SAR_RS00895)	OSHJJ2	152940	20	20	10	10	10	10	10	20	20	20	10	10	10	10	N/A	48	48	48	48	10	20	20	9	48	74	20	48
SAUR0181 (SAR_RS00905)	argJ	154467	45	45	160	160	160	160	160	45	45	45	N/A	160	160	N/A	N/A	64	64	64	64	N	45	45	154	49	2	87	2
SAUR0182 (SAR_RS00910)	argC	155720	72	72	N/A	N/A	N/A	N/A	N/A	67	72	67	115	115	115	115	94	94	94	94	94	115	228	228	5	75	116	150	95
SAUR0183 (SAR_RS00915)	rocD1	156790	89	89	154	154	154	154	154	89	260	89	154	154	473	154	205	205	280	122	205	471	N/A	N/A	5	93	126	209	N/A
SAUR0184 (SAR_RS00920)	brnQ1	158227	79	79	118	118	118	118	118	409	79	79	118	118	118	118	58	58	58	58	167	N/A	79	79	N/A	80	96	169	58
SAUR0185 (SAR_RS00925)	N/A	159859	244	244	N/A	N/A	N/A	N/A	84	23	23	23	N/A	84	84	N/A	32	32	65	32	32	N/A	23	23	84	25	13	49	19
SAUR0186 (SAR_RS00930)	iodC	160483	90	90	180	N/A	N/A	180	N/A	90	319	90	N/A	180	180	N/A	146	373	146	146	146	180	N/A	N/A	32	115	11	250	147
SAUR0187 (SAR_RS00935)	glcA=ptsG	162395	95	95	180	N/A	N/A	180	180	95	95	95	N/A	180	180	N/A	N/A	147	147	147	240	615	95	95	11	40	66	243	148
SAUR0188 (SAR_RS00940)	DUF871	165026	88	88	86	N/A	86	86	86	417	88	93	86	86	86	86	121	121	121	121	121	86	88	88	8	81	159	81	81
SAUR0189 (SAR_RS00945)	murQ	166081	89	89	156	156	156	156	N/A	89	89	89	N/A	156	156	N/A	N/A	N/A	N/A	N/A	N/A	156	380	380	9	89	15	224	N/A
SAUR0190 (SAR_RS00950)	OSHJI0	166989	92	92	171	171	171	171	171	92	92	92	171	171	171	171	134	357	134	134	134	171	N/A	N/A	8	105	142	231	N/A
SAUR0191 (SAR_RS00955)	rpiRB	168446	N/A	N/A	11	11	11	11	11	51	51	51	11	11	11	11	66	66	66	66	66	11	51	51	10	58	23	124	67
SAUR0193 (SAR_RS00965)	hsdR	170578	997	997	N/A	N/A	N/A	N/A	N/A	211	178	178	297	297	975	297	N/A	N/A	N/A	N/A	N/A	N	N/A	N/A	887	N/A	300	376	N/A
SAUR0195 (SAR_RS00975)	oppF2	176704	747	747	N/A	205	205	N/A	205	129	129	129	205	205	205	205	173	428	173	173	274	205	129	129	8	137	182	2	N/A
SAUR0196 (SAR_RS00980)	N/A	178298	39	39	213	213	213	213	213	39	39	39	213	213	213	213	N/A	163	163	163	163	213	39	39	9	N/A	215	268	N/A
SAUR0197 (SAR_RS00985)	oppC2	179737	83	83	N/A	N/A	N/A	N/A	N/A	83	83	83	139	139	139	139	N/A	189	189	110	189	139	83	83	489	85	140	190	111
SAUR0198 (SAR_RS00990)	oppA2	180917	744	744	N/A	N/A	N/A	N/A	N/A	125	125	125	N/A	213	720	213	N/A	171	379	171	171	719	125	125	637	135	218	270	N/A
SAUR0199 (SAR_RS00995)	ggt	182730	41	41	693	693	693	693	693	41	41	41	693	694	694	693	N/A	744	744	744	744	693	41	41	576	49	65	683	N/A
SAUR0200 (SAR_RS01000)	acpD	185274	21	21	97	97	97	97	97	21	21	21	97	97	97	97	2	2	2	2	2	97	N/A	N/A	8	N/A	21	125	122
SAUR0201 (SAR_RS01005)	OSHJG7	186109	296	296	93	93	93	93	93	60	60	60	93	93	93	93	120	120	120	79	120	93	60	60	8	63	95	122	60
SAUR0202 (SAR_RS01010)	malK=ugpC	187072	69	69	109	109	109	109	109	412	69	69	109	109	109	109	93	93	93	93	93	109	N/A	N/A	8	72	25	152	69
SAUR0203 (SAR_RS01015)	malF	188182	95	95	149	149	149	149	149	101	95	95	149	149	149	149	118	118	118	118	118	149	N/A	N/A	7	43	150	2	N/A
SAUR0204 (SAR_RS01020)	malC	189456	29	29	163	163	163	163	N/A	29	29	29	163	163	163	163	29	29	29	29	29	163	29	29	8	95	95	29	N/A
SAUR0205 (SAR_RS01025)	malD	190726	68	68	108	108	108	108	108	68	68	68	108	108	108	108	N/A	92	92	92	92	108	N/A	N/A	8	72	68	140	N/A
SAUR0206 (SAR_RS01030)	vrbE	191836	100	100	93	93	93	93	93	100	100	100	93	93	93	93	129	129	129	129	129	93	100	100	394	110	421	195	130
SAUR0207 (SAR_RS01035)	mviM	192937	N/A	N/A	N/A	N/A	N/A	N/A	33	97	92	97	33	33	33	33	172	172	172	119	172	33	N/A	N/A	35	94	355	176	N/A
SAUR0208 (SAR_RS01040)	iolE	194029	29	29	103	103	103	103	103	29	29	29	N/A	103	103	103	83	83	83	83	83	103	29	29	8	69	69	62	N/A
SAUR0209 (SAR_RS01045)	O7A1X1	195356	170	170	91	91	91	91	N/A	34	34	34	91	91	91	91	34	34	95	34	34	91	34	34	1	37	1	24	45
SAUR0210 (SAR_RS01050)	uhpT	196083	81	81	N/A	133	133	N/A	133	170	229	N/A	N/A	133	133	N/A	106	106	106	106	106	133	81	81	8	114	110	2	107
SAUR0211 (hotR)	N/A	197821	57	57	33	33	33	33	57	57	57	57	33	33	33	33	74	74	74	74	74	33	N/A	N/A	9	60	90	32	N/A
SAUR0212 (hotS)	N/A	198572	116	116	N/A	171	171	171	N/A	518	116	116	171	428	428	171	149	149	149	149	149	171	116	116	8	121	516	239	N/A
SAUR0213 (hotA)	N/A	200125	92	N/A	N/A	N/A	N/A	N/A	50	407	92	92	413	50	50	413	125	125	125	125	125	50	92	92	9	77	41	186	41
SAUR0214 (SAR_RS01070)	pflB	201681	93	93	N/A	N/A	N/A	N/A	N/A	93	93	93	143	143	143	143	122	122	122	122	122	143	93	93	11	99	128	2	124
SAUR0215 (SAR_RS01075)	N/A	203953	3	3	3	3	3	3	3	3	3	3	3	3	3	3	98	98	98	59	98	3	3	3	10	N/A	75	12	48
SAUR0217 (SAR_RS01085)	glpQ1	205030	106	106	163	163	163	163	163	106	106	106	N/A	641	163	N/A	137	137	322	137	137	163	106	106	595	110	171	195	139
SAUR0218 (SAR_RS01090)	O2YV49	206957	43	43	14	14	14	14	14	43	43	43	14	14	14	14	56	56	56	56	56	14	43	43	7	N/A	43	43	57
SAUR0220 (SAR_RS01100)	N/A	N/A	N/A	N/A	29	29	29	29	29	N/A	N/A	N/A	29	29	29	29	3	3	3	3	3	N/A	N/A	N/A	6	1	N/A	1	N/A
SAUR0221 (SAR_RS01105)	N/A	209941	2	2	4	4	4	4	4	2	2	2	4	4	4	4	2	2	2	2	2	N/A	2	2	3	2	N/A	2	N/A
[illegible]	[illegible]	[illegible]	[illegible]	[illegible]	[illegible]	[illegible]	[illegible]	[illegible]	[illegible]	[illegible]	[illegible]	[illegible]	[illegible]	[illegible]	[illegible]	[illegible]	[illegible]	[illegible]	[illegible]	[illegible]	[illegible]	[illegible]	[illegible]	[illegible]	[illegible]	[illegible]	[illegible]	[illegible]	[illegible]

Locus (cg MLST)	Alternative designation	Position (in ED133)	Study Isolates: Swan isolate 15V8707	Duck isolate 511525-22	Duck isolate V315	Duck isolate 511509-22	Duck isolate 511510-22	Whistling duck isolate [illegible]	Duck isolate V482	CC133: CP001996.1, ED133	CP065353.1, AR39 [illegible]	LR134271.1, NCTC7988	CC692: CP042650.1, X22	UHAK01000002.1, NCTC9546	PubMLST ID 39948, 973N0056	CP129360, CKMM-401M	CC522: Goat isolate 17CS1042	PubMLST ID 24977	PubMLST ID 24358	PubMLST ID 11704	SAMEA1904171	ST1347: PubMLST ID 39945, 61290	Chinese CC133 MRSA isolates: JAOAKU01, GDQ20D75A	JAFEOM01, GDQ20D75A	CC5: CP001781.1, ED98	ST71: SAMN03247575, MOK-099	CC182: CP070991, CBS2016-05	CC707: CP029751, SMITH	CC1956: CP084892, WT19
SAUR0463 (SAR_RS02315)	nudG	498303	2	2	2	2	2	2	2	2	N/A	2	46	46	46	46	18	18	18	18	18	N/A	2	2	33	7	72	93	16
SAUR0464 (SAR_RS02320)	bltD	498688	54	54	54	54	54	54	54	54	54	54	6	6	291	N/A	N/A	71	71	71	71	6	N/A	N/A	238	20	87	44	67
SAUR0465 (SAR_RS02325)	vibF	499342	55	55	55	N/A	N/A	55	55	55	55	55	14	14	14	14	67	67	144	67	67	14	N/A	N/A	7	28	85	100	N/A
SAUR0466 (SAR_RS02330)	vibE	500121	422	422	82	82	82	82	82	82	82	82	135	135	135	135	114	114	243	114	114	135	N/A	N/A	13	31	141	170	115
SAUR0467 (SAR_RS02335)	gltC	501354	66	66	66	66	66	66	66	66	187	66	196	196	196	196	96	96	96	96	96	196	66	66	4	8	116	149	246
SAUR0468 (SAR_RS02340)	gltB	502419	1870	1870	N/A	291	291	N/A	N/A	1786	915	274	1817	N/A	1818	1817	N/A	668	1007	384	668	N/A	291	291	18	84	N/A	673	N/A
SAUR0469 (SAR_RS02345)	gltD	506936	113	113	113	113	113	113	113	113	113	113	N/A	677	95	N/A	149	149	337	149	241	95	113	113	10	35	187	245	N/A
SAUR0471 (SAR_RS02355)	treP	509231	115	115	115	115	115	115	115	265	115	115	12	12	12	N/A	274	274	274	151	274	12	115	115	7	24	N/A	277	N/A
SAUR0472 (SAR_RS02360)	treC	510722	130	130	130	130	130	130	130	122	130	122	20	20	20	20	168	168	372	168	168	20	130	130	9	45	219	262	170
SAUR0473 (SAR_RS02365)	treR	512387	25	25	25	25	25	25	25	25	25	25	14	N/A	14	14	31	177	31	31	31	14	25	25	7	21	31	115	80
SAUR0474 (SAR_RS02370)	N/A	513516	13	13	13	13	13	13	13	13	13	13	9	9	53	9	13	13	13	13	13	N/A	13	13	9	9	25	13	N/A
SAUR0475 (SAR_RS02375)	O5HIK0	513756	3	3	3	3	3	N/A	3	84	3	3	6	6	6	6	3	3	3	3	3	6	3	3	6	6	73	34	34
SAUR0476 (SAR_RS02380)	dnaX	514349	2	2	N/A	N/A	N/A	N/A	N/A	542	2	2	197	197	197	197	N/A	167	342	167	167	197	2	2	596	50	207	255	2
SAUR0477 (SAR_RS02385)	yaaK	516136	1	1	1	1	1	1	1	1	1	1	1	1	1	1	1	1	1	1	1	1	1	1	2	35	1	1	1
SAUR0478 (SAR_RS02390)	recR	516460	2	2	2	2	2	2	2	2	2	2	9	9	9	9	2	2	2	2	2	9	2	2	11	19	71	2	2
SAUR0484 (SAR_RS02420)	yaaO	523901	103	103	N/A	N/A	N/A	N/A	N/A	103	103	103	18	18	18	18	203	203	203	130	203	18	N/A	N/A	11	10	162	206	N/A
SAUR0485 (SAR_RS02425)	tmk	525240	51	51	51	51	51	51	51	51	51	51	7	7	7	7	20	20	20	20	20	7	51	51	7	7	16	89	20
SAUR0486 (SAR_RS02430)	darA	525885	1	1	1	1	1	1	1	1	1	1	1	1	143	1	1	1	1	1	1	142	1	1	4	4	1	73	10
SAUR0487 (SAR_RS02435)	N/A	526333	1	1	N/A	1	1	1	1	1	1	1	4	4	4	4	1	1	1	1	1	N/A	1	1	4	4	1	4	1
SAUR0488 (SAR_RS02440)	holB	526428	81	81	81	81	81	81	81	81	81	81	9	9	9	9	7	7	7	7	7	9	81	81	9	5	121	72	93
SAUR0489 (SAR_RS02445)	yaaT=tpl	527355	16	16	16	16	16	16	16	78	16	78	7	7	7	7	49	49	49	49	49	7	16	16	7	N/A	138	179	103
SAUR0490 (SAR_RS02450)	yabA	528175	1	1	1	1	1	1	1	1	1	1	1	1	1	1	1	1	1	1	1	144	1	1	1	1	1	1	1
SAUR0491 (SAR_RS02455)	yabB	528796	79	79	79	79	79	79	79	79	189	79	299	9	9	299	N/A	126	126	98	126	377	N/A	N/A	7	6	126	148	100
SAUR0492 (SAR_RS02460)	N/A	529514	1	1	1	1	1	1	1	1	1	1	80	1	1	80	19	19	19	19	19	N/A	1	1	1	1	1	1	1
SAUR0493 (SAR_RS02465)	rsmI	529764	44	44	44	44	44	44	44	44	44	44	76	76	76	76	95	95	95	95	95	76	N/A	N/A	9	9	116	96	96
SAUR0494 (SAR_RS02470)	N/A	530628	1	1	1	1	1	1	1	1	1	1	1	1	1	1	5	5	5	5	5	N/A	1	1	1	1	N/A	1	5
SAUR0495 (SAR_RS02475)	metS=metG	530888	392	392	155	155	155	155	155	155	155	155	N/A	27	27	27	189	437	189	189	189	27	N/A	N/A	10	208	236	306	N/A
SAUR0496 (SAR_RS02480)	tatP	532892	3	3	3	3	3	3	3	3	3	3	13	13	13	13	81	81	81	81	81	332	3	3	35	N/A	298	139	N/A
SAUR0497 (SAR_RS02485)	rnmV	533832	48	48	48	48	48	48	48	48	48	48	N/A	N/A	N/A	307	2	2	2	2	2	N/A	48	48	1	11	80	105	2
SAUR0498 (SAR_RS02490)	N/A	534379	69	69	N/A	N/A	N/A	N/A	N/A	69	69	69	N/A	463	N/A	N/A	150	150	150	90	150	463	69	69	9	19	120	156	N/A
SAUR0499 (SAR_RS02495)	N/A	535372	1	1	1	1	1	1	1	1	1	1	1	1	1	1	1	1	1	1	1	1	1	1	1	3	1	1	1
SAUR0500 (SAR_RS02500)	ispE	535945	65	65	65	65	65	65	65	65	65	65	11	11	11	11	83	83	83	83	83	11	65	65	12	N/A	48	126	85
SAUR0501 (SAR_RS02505)	purR	536807	2	2	N/A	N/A	N/A	N/A	N/A	2	2	2	5	5	5	5	2	2	2	2	2	72	2	2	6	21	99	2	2
SAUR0502 (SAR_RS02510)	yabJ	537648	25	25	25	25	25	25	25	25	25	25	6	6	6	6	2	142	2	2	2	6	25	25	7	19	25	25	66
SAUR0503 (SAR_RS02515)	N/A	538100	2	2	2	2	2	2	2	2	2	2	2	2	2	2	2	2	2	2	2	2	2	2	2	2	182	83	2
SAUR0504 (SAR_RS02520)	N/A	538590	2	N/A	2	2	2	2	2	2	2	2	3	3	3	3	2	2	2	2	2	3	N/A	N/A	3	3	2	2	2
SAUR0505 (SAR_RS02525)	glmU	538743	573	573	108	108	108	108	108	108	108	108	33	33	33	N/A	139	139	139	139	139	33	108	108	38	35	79	204	129
SAUR0506 (SAR_RS02530)	prs	540242	21	21	21	21	21	21	21	21	21	21	1	1	80	N/A	36	36	36	36	36	1	21	21	1	1	36	95	21
SAUR0507 (SAR_RS02535)	N/A	541265	2	2	2	2	2	2	2	2	2	2	1	1	1	1	2	2	2	2	2	N/A	2	2	1	1	2	2	2
SAUR0508 (SAR_RS02540)	rplY	541357	2	2	2	2	2	2	2	2	2	2	6	6	6	6	49	49	49	49	49	6	2	2	7	5	5	5	5
SAUR0509 (SAR_RS02545)	pth	542321	34	34	34	34	34	34	34	34	34	34	68	68	68	68	55	55	55	55	55	68	34	34	7	9	35	2	56
SAUR0510 (SAR_RS02550)	mfd	542893	219	219	N/A	N/A	N/A	N/A	N/A	219	219	219	N/A	369	369	N/A	290	766	708	290	290	1288	N/A	N/A	114	N/A	626	485	N/A
SAUR0511 (SAR_RS02555)	yabM	546389	116	116	116	116	116	116	116	116	N/A	116	346	346	346	346	N/A	161	161	161	161	N/A	116	116	7	13	197	253	N/A
SAUR0512 (SAR_RS02560)	mazG	547915	96	96	N/A	N/A	N/A	N/A	N/A	96	96	96	176	176	176	176	N/A	134	134	134	134	573	96	96	7	18	177	237	135
SAUR0513 (SAR_RS02565)	hslR	549105	1	1	1	1	1	1	1	127	1	1	3	3	3	3	2	2	2	2	2	3	1	1	3	14	1	1	N/A
SAUR0514 (SAR_RS02570)	divIC	549386	7	7	7	7	7	N/A	7	7	7	7	3	3	3	3	2	2	2	2	2	3	7	7	7	6	3	7	3
SAUR0515 (SAR_RS02575)	yabR	549883	2	2	2	2	2	2	2	2	2	2	5	5	5	5	34	34	34	34	34	5	2	2	6	5	2	2	2
SAUR0516 (SAR_RS02580)	tilS	550464	30	30	30	N/A	N/A	30	30	30	30	30	157	157	495	157	126	126	126	126	126	157	30	30	7	26	160	206	2
SAUR0517 (SAR_RS02585)	N/A	551764	11	11	11	11	11	11	11	164	11	11	4	4	4	4	2	2	2	2	2	4	11	11	1	1	58	76	105
SAUR0518 (SAR_RS02590)	ftsH	552561	96	96	96	96	96	N/A	96	96	96	96	9	9	9	9	134	134	134	134	134	9	N/A	N/A	9	N/A	178	96	135
SAUR0519 (SAR_RS02595)	hslO	555300	2	2	2	2	2	2	2	2	2	2	123	123	123	123	2	2	2	2	2	123	2	2	12	8	125	2	91
SAUR0520 (SAR_RS02600)	cysK	556360	265	85	85	85	85	85	N/A	85	85	85	9	9	9	9	101	101	101	101	101	9	85	85	8	33	126	161	14
SAUR0521 (SAR_RS02605)	folP	557508	21	21	N/A	N/A	N/A	N/A	N/A	21	21	21	N/A	88	88	N/A	58	2	2	2	2	88	21	21	82	84	118	182	N/A
SAUR0522 (SAR_RS02610)	folB	558289	15	15	15	15	15	15	15	15	15	15	5	5	5	5	2	2	15	2	2	N/A	15	15	5	5	23	2	N/A
SAUR0523 (SAR_RS02615)	folK	558651	42	42	42	42	42	42	42	42	42	42	180	180	180	180	52	52	52	52	52	180	42	42	5	19	10	2	10
SAUR0524 (SAR_RS02620)	N/A	559521	5	5	5	5	5	5	5	5	5	5	2	2	2	2	2	2	2	2	2	2	5	5	2	2	2	2	2
SAUR0525 (SAR_RS02625)	lysS	559666	83	83	83	83	83	83	83	83	83	N/A	N/A	367	486	142	104	104	104	104	104	142	83	83	10	20	143	186	66
SAUR0541 (SAR_RS02705)	pdxR	568316	N/A	N/A	78	78	78	78	N/A	78	78	78	131	131	131	131	105	105	105	105	105	469	78	78	457	11	132	34	182
SAUR0542 (SAR_RS02710)	pdxS	569802	2	2	2	2	2	2	2	2	2	2	283	68	68	N/A	177	185	128	38	128	68	2	2	7	16	N/A	130	11
SAUR0543 (SAR_RS02715)	pdxT	570693	1	1	1	1	1	1	1	1	1	1	63	63	63	63	1	1	113	1	1	63	N/A	N/A	1	N/A	1	87	56
SAUR0545 (SAR_RS02725)	nupC1	571461	29	29	29	29	29	29	29	29	29	29	81	356	81	81	94	94	94	94	94	81	29	29	6	32	29	91	N/A
SAUR0546 (SAR_RS02730)	ctsR	572833	15	15	15	15	15	15	15	15	15	15	5	5	5	5	25	25	25	25	25	5	15	15	5	5	36	15	26
SAUR0547 (SAR_RS02735)	mcsA	573313	19	19	19	19	19	19	19	19	19	19	2	2	2	2	46	46	46	65	46	N/A	19	19	217	6	81	81	66
SAUR0548 (SAR_RS02740)	mcsB	573869	24	24	N/A	N/A	N/A	N/A	N/A	24	24	24	11	11	11	11	11	11	11	11	11	11	N/A	N/A	10	11	11	11	94
SAUR0549 (SAR_RS02745)	clpC	574890	814	814	108	108	108	108	108	108	108	108	253	253	253	253	443	207	207	207	207	789	108	108	15	44	268	282	208
SAUR0550 (SAR_RS02750)	N/A	577274	2	2	2	2	2	2	2	13	2	13	4	4	4	4	4	4	4	4	4	N/A	2	2	4	4	17	4	4
SAUR0551 (SAR_RS02755)	radA	577831	535	535	89	89	89	89	89	89	262	89	9	9	9	9	112	112	112	112	112	9	262	262	9	9	82	82	N/A
SAUR0552 (SAR_RS02760)	pilT	579220	96	96	N/A	96	96	N/A	313	96	96	96	76	76	76	76	121	121	121	121	121	76	96	96	449	30	153	153	60
SAUR0553 (SAR_RS02765)	gltX	580850	104	104	N/A	N/A	N/A	N/A	N/A	104	104	104	173	173	173	N/A	221	221	221	136	221	173	104	104	9	35	174	224	N/A
SAUR0554 (SAR_RS02770)	cysE	582733	1	1	1	1	1	1	1	1	1	1	9	9	237	N/A	134	2	9	9	9	N/A	1	1	5	9	39	39	9
SAUR0555 (SAR_RS02775)	cysS	583358	98	98	98	98	98	98	98	98	98	98	94	94	94	94	136	136	315	136	136	94	98	98	7	17	279	75	137
SAUR0556 (SAR_RS02780)	mrnC	584808	2	2	2	2	2	2	2	2	2	2	6	6	6	6	2	2	2	2	2	N/A	2	2	6	6	125	2	22
SAUR0557 (SAR_RS02785)	vacO=rlmB	585163	80	80	80	80	80	80	80	80	80	80	6	6	6	6	99	99	206	99	99	6	80	80	9	22	2	2	66
SAUR0558 (SAR_RS02790)	vacP	585909	11	11	11	11	11	11	N/A	11	11	11	N/A	7	7	N/A	66	66	119	66	66	7	11	11	6	7	78	94	11
SAUR0559 (SAR_RS02795)	sigH	586514	64	64	64	64	64	64	64	64	64	64	N/A	21	21	N/A	116	178	116	86	116	302	64	64	8	N/A	105	101	N/A
SAUR0560 (SAR_RS02800)	N/A	587397	1	1	1	1	1	1	1	1	1	1	1	1	1	1	1	1	1	1	1	1	8	8	1	1	1	1	1
SAUR0561 (SAR_RS02805)	N/A	587592	49	49	49	49	49	49	49	49	49	49	4	4	4	4	63	63	63	63	63	4	49	49	4	8	63	63	2
SAUR0562 (SAR_RS02810)	rplK	588321	166	166	1	1	1	1	1	1	1	1	2	2	2	2	1	1	1	1	1	2	1	1	2	2	1	31	1
SAUR0564 (SAR_RS02820)	rplJ	589915	1	1	1	1	1	1	1	1	1	1	4	4	4	4	1	1	1	1	111	4	1	1	4	1	1	1	1
SAUR0565 (SAR_RS02825)	rplL	590458	3	3	3	3	3	3	3	3	3	3	5	5	5	5	3	3	3	3	3	5	3	3	5	5	3	3	2
SAUR0566 (SAR_RS02830)	ybxB=rsmC	591001	65	65	65	65	65	65	65	65	65	65	8	8	8	8	85	85	85	85	85	8	65	65	8	25	28	143	86
SAUR0567 (SAR_RS02835)	rpoB	591824	2	2	2	2	2	2	2	223	2	223	N/A	1283	1221	N/A	296	296	696	296	296	1220	N/A	N/A	818	69	635	498	297
SAUR0568 (SAR_RS02840)	rpoC	595512	1021	1021	43	N/A	43	43	43	43	43	43	N/A	519	519	N/A	N/A	223	223	223	223	1004	N/A	N/A	831	9	309	402	N/A
SAUR0569 (SAR_RS02845)	N/A	599271	1	1	1	1	1	1	1	1	1	1	1	1	1	1	7	7	7	7	7	1	1	1	1	1	34	34	1
SAUR0570 (SAR_RS02850)	rpsL	599623	1	1	1	1	1	1	1	1	1	1	1	1	1	1	1	1	1	1	1	1	3	3	1	5	1	1	1
SAUR0571 (SAR_RS02855)	rpsG	600102	3	3	3	3	3	3	3	3	3	3	1	1	1	1	3	3	3	3	3	1	3	3	1	1	3	3	3
SAUR0572 (SAR_RS02860)	efe=fusA	600695	36	36	36	N/A	N/A	36	N/A	36	36	36	237	237	237	237	200	471	200	200	200	237	36	36	8	7	412	325	36
SAUR0573 (SAR_RS02865)	tuf	602993	2	2	2	2	2	2	2	2	2	2	8	8	8	8	56	56	56	56	56	8	2	2	9	8	122	39	11
SAUR0574 (SAR_RS02870)	yhaA=amaA	604459	93	93	93	93	93	93	93	93	283	93	169	169	169	169	119	119	119	119	119	169	333	333	12	N/A	179	N/A	134
SAUR0575 (SAR_RS02875)	kbl	605805	415	415	25	25	25	25	25	25	25	25	129	129	129	129	25	25	25	25	25	395	25	25	7	6	131	152	N/A
SAUR0576 (SAR_RS02880)	hchA	607259	83	83	83	83	83	83	83	83	83	83	25	255	255	25	193	105	105	105	105	25	83	83	11	22	121	149	36
SAUR0577 (SAR_RS02885)	araB	608295	111	111	N/A	N/A	N/A	N/A	N/A	609	111	111	314	314	314	N/A	151	151	151	151	151	314	111	111	16	29	194	83	2
SAUR0578 (SAR_RS02890)	O5HIC2	610146	10	10	10	10	10	10	N/A	10	10	10	20	20	315	20	136	136	136	93	136	20	N/A	N/A	6	20	117	163	N/A
SAUR0579 (SAR_RS02895)	ilvE	611447	378	378	12	12	12	12	12	12	12	12	202	202	202	202	86	86	86	86	86	N/A	12	12	7	20	105	105	12
SAUR0580 (SAR_RS02900)	ppaX1	612771	2	2	2	2	2	2	N/A	2	2	2	58	58	58	58	2	2	167	2	2	58	2	2	5	7	2	2	N/A
SAUR0581 (SAR_RS02905)	dck	613570	2	2	2	2	2	2	N/A	2	2	2	14	14	14	14	2	2	2	2	2	14	2	2	6	21	2	2	53
SAUR0582 (SAR_RS02910)	dgk	614225	31	N/A	31	31	31	31	31	31	31	31	4	4	4	4	55	55	55	55	55	N/A	31	31	5	14	60	60	55
SAUR0583 (SAR_RS02915)	tadA	614909	2	2	2	2	2	2	2	143	2	2	6	6	6	6	2	2	2	2	2	N/A	2	2	6	12	50	70	2
SAUR0584 (SAR_RS02920)	A5IOB3	615526	79	79	79	79	79	79	79	79	79	79	15	15	15	15	2	2	2	2	2	15	79	79	7	15	2	2	2
SAUR0585 (SAR_RS02925)	azo1	616415	8	N/A	8	8	8	8	8	8	8	8	7	7	7	7	42	42	95	42	69	7	8	8	4	7	43	43	N/A
SAUR0586 (SAR_RS02930)	N/A	617412	N/A	N/A	276	N/A	N/A	276	N/A	276	N/A	276	N/A	1162	N/A	N/A	N/A	N/A	N/A	N/A	N/A	N/A	N/A	N/A	N/A	N/A	N/A	N/A	N/A
SAUR0588 (SAR_RS02940)	sdeA	628499	38	38	38	38	38	38	38	541	38	38	302	302	302	302	131	131	131	131	131	602	38	38	42	57	179	218	N/A
SAUR0589 (SAR_RS02945)	sdeB	630094	455	455	455	455	455	455	N/A	539	455	454	N/A	586	550	414	456	456	456	456	456	549	455	455	7	27	455	240	143
SAUR0590 (SAR_RS02950)	folE2	631866	42	42	42	42	42	42	42	42	42	42	9	9	9	9	2	2	2	2	2	9	42	42	8	14	38	104	12
SAUR0591 (SAR_RS02955)	bshB2	632757	20	20	20	N/A	N/A	20	20	20	20	20	5	5	5	5	3	3	3	3	3	5	20	20	5	13	96	111	77
SAUR0592 (SAR_RS02960)	yoiF	633436	28	28	28	28	28	28	28	28	28	28	6	6	6	6	41	41	41	41	41	6	28	28	6	8	2	12	42
SAUR0593 (SAR_RS02965)	naeB	634074	65	65	65	65	65	65	65	65	65	65	N/A	96	96	96	81	81	81	81	81	96	65	65	6	6	97	138	82
SAUR0594 (SAR_RS02970)	hxlA	634909	44	44	44	44	44	44	44	44	44	44	72	72	72	72	44	44	44	44	44	72	44	44	243	6	12	61	61
SAUR0595 (SAR_RS02975)	sis=hxlB	635543	53	53	53	53	53	53	53	53	53	53	86	86	86	86	138	53	137	53	53	86	53	53	8	8	94	91	70
SAUR0596 (SAR_RS02980)	O2FJ68	636204	49	49	49	49	49	49	49	39	49	39	61	302	61	61	N/A	49	49	49	49	N/A	49	49	268	158	N/A	16	50
SAUR0597 (SAR_RS02985)	proP	637653	100	100	100	N/A	N/A	100	100	516	100	100	N/A	378	378	N/A	2	2	2	2	2	378	100	100	8	N/A	159	203	16
SAUR0598 (SAR_RS02990)	vraA	639597	N/A	109	109	109	109	109	109	109	109	109	179	179	179	179	314	145	145	145	145	179	109	109	11	11	180	228	N/A
SAUR0599 (SAR_RS02995)	vraB	640975	622	622	116	N/A	116	116	116	116	116	116	N/A	439	439	439	162	162	345	162	162	194	N/A	N/A	10	35	197	253	N/A
SAUR0600 (SAR_RS03000)	N/A	642089	37	37	37	37	37	37	37	37	37	37	95	95	95	95	47	47	47	47	47	N/A	37	37	7	7	60	75	48
SAUR0601 (SAR_RS03005)	O5HI98	N/A	N/A	N/A	N/A	N/A	N/A	N/A	N/A	N/A	N/A	N/A	11	11	11	11	N/A	N/A	N/A	N/A	N/A	N/A	N/A	N/A	11	11	N/A	N/A	N/A
SAUR0602 (SAR_RS03010)	N/A	642926	2	2	2	2	2	2	2	2	2	2	2	2	2	2	2	2	2	2	2	2	2	2	2	2	2	26	17
SAUR0603 (SAR_RS03015)	thiD1	643628	68	68	N/A	N/A	N/A	N/A	N/A	68	68	68	119	119	119	119	90	90	90	90	90	119	68	68	8	23	121	159	91
SAUR0604 (SAR_RS03020)	ung	644642	44	44	44	44	44	44	44	44	44	44	N/A	71	71	N/A	2	2	2	2	2	71	44	44	9	13	72	95	61
SAUR0605 (SAR_RS03025)	ywdI	645299	2	2	N/A	N/A	N/A	N/A	N/A	2	2	2	73	73	73	73	91	125	2	2	91	73	2	2	8	8	75	93	N/A
SAUR0606 (SAR_RS03030)	N/A	645812	29	N/A	N/A	N/A	N/A	N/A	N/A	29	29	29	2	2	2	2	2	85	2	2	2	2	29	29	6	7	2	2	2
SAUR0607 (SAR_RS03035)	yfnA2	646301	497	497	94	94	94	94	94	94	94	94	150	150	150	150	N/A	120	120	119	120	150	94	94	12	31	438	203	120
SAUR0608 (SAR_RS03040)	DUF3815	647926	11	11	11	11	11	11	11	11	11	11	98	114	114	98	21	21	21	21	21	156	11	11	67	70	10	11	22
SAUR0609 (SAR_RS03045)	DUF1212_L1	648391	65	65	65	65	65	65	65	65	65	65	N/A	173	173	N/A	77	77	77	77	77	173	65	65	7	18	45	134	77
SAUR0610 (SAR_RS03050)	hemQ	649702	74	74	74	74	74	74	74	74	74	74	N/A	311	104	104	32	32	32	32	32	104	74	74	8	9	2	119	32
SAUR0611 (SAR_RS03055)	pta	650622	467	467	70	N/A	N/A	70	N/A	70	70	70	114	114	114	114	70	70	70	70	70	114	70	70	8	13	115	155	28
pta	N/A	650718	372	372	7	373	373	7	7	7	7	7	4	4	4	4	7	7	7	7	7	N/A	7	7	12	1	13	7	7
SAUR0612 (SAR_RS03060)	lipL	651611	72	72	72	72	72	72	72	282	72	72	107	107	107	107	90	90	90	90	90	107	72	72	7	8	110	61	338
SAUR0613 (SAR_RS03065)	mvaK1	653035	55	55	55	55	55	55	55	55	55	55	53	53	53	53	77	77	77	77	77	53	55	55	8	9	32	55	55
SAUR0614 (SAR_RS03070)	mvaD	653960	401	401	75	N/A	N/A	75	N/A	345	75	75	117	117	117	117	151	2	151	98	151	117	75	75	7	8	119	154	2
SAUR0615 (SAR_RS03075)	mvaK2	654956	23	23	23	N/A	N/A	23	23	23	167	23	172	172	172	172	44	44	44	44	44	172	23	23	7	9	281	44	76
SAUR0616 (SAR_RS03080)	DUF1450	656209	144	144	2	2	2	2	2	2	2	2	2	2	2	2	32	32	32	32	32	2	2	2	3	3	39	3	2
SAUR0617 (SAR_RS03085)	ykeC	656602	549	549	81	N/A	N/A	81	81	81	81	81	338	338	338	N/A	216	373	216	153	216	N/A	81	81	N/A	44	188	253	155
SAUR0618 (SAR_RS03090)	O5HIR1	657911	31	31	31	31	31	31	31	31	31	31	34	34	34	34	63	63	134	63	63	N/A	31	31	33	34	64	97	64
SAUR0619 (SAR_RS03095)	N/A	658409	45	45	45	45	45	45	45	45	45	45	5	5	5	5	45	45	45	45	45	5	45	45	5	5	10	10	10
SAUR0620 (SAR_RS03100)	O5HI79	658976	453	453	18	N/A	N/A	18	18	18	48	18	N/A	N/A	N/A	N/A	N/A	N/A	N/A	N/A	N/A	N/A	N/A	N/A	87	108	N/A	N/A	174
SAUR0623 (SAR_RS03115)	DUF0443	661247	301	N/A	301	301	301	N/A	301	301	301	301	182	182	182	182	N/A	N/A	N/A	N/A	N/A	N/A	301	301	6	31	177	36	N/A
SAUR0625 (SAR_RS03125)	O5HI67	663090	79	N/A	79	79	79	79	79	79	228	79	43	43	43	43	105	105	105	105	105	43	228	228	5	8	130	175	64
SAUR0626 (SAR_RS03130)	N/A	664238	8	8	8	8	8	8	8	8	8	8	58	2	2	58	57	57	57	57	57	N/A	8	8	2	2	71	7	8
SAUR0627 (SAR_RS03135)	O8NXU4	664943	1	1	1	1	1	1	1	38	1	38	6	6	6	6	N/A	46	46	46	46	N/A	1	1	6	1	6	76	6
SAUR0628 (SAR_RS03140)	O5HI65	665459	452	452	85	85	85	85	85	85	85	85	12	12	12	N/A	109	109	233	109	109	12	85	85	6	34	131	171	110
SAUR0629 (SAR_RS03145)	ywhD	666797	5	5	5	5	5	5	5	5	5	5	5	5	205	5	68	68	68	68	68	5	5	5	6	8	79	5	5
SAUR0630 (SAR_RS03150)	adhA=adhP	667802	15	15	15	15	15	15	15	15	15	15	126	126	126	126	N/A	102	102	102	102	126	15	15	8	10	2	167	103
SAUR0631 (SAR_RS03155)	N/A	668887	5	5	5	5	5	5	5	5	5	5	N/A	N/A	N/A	N/A	3	3	3	3	3	N/A	5	5	N/A	3	5	5	N/A
SAUR0632 (SAR_RS03160)	ywiB	669087	29	29	29	29	29	29	29	29	29	29	4	4	4	4	35	35	35	35	35	4	29	29	4	4	1	1	1
SAUR0633 (SAR_RS03165)	argS	669500	105	105	105	105	105	105	105	105	105	105	157	157	157	157	137	137	268	137	137	157	105	105	11	13	158	204	N/A
SAUR0634 (SAR_RS03170)	nth2	671640	18	18	18	18	18	18	18	18	N/A	18	43	43	43	43	18	18	18	18	18	43	18	18	11	21	233	29	29
SAUR0635 (SAR_RS03175)	A5IOG3	672654	73	73	73	73	73	73	73	73	73	73	8	8	8	8	100	100	N/A	100	100	8	73	73	8	10	121	159	2
SAUR0637 (SAR_RS03185)	ppaX2	674680	245	245	54	54	54	54	54	54	54	54	5	5	28	5	50	50	50	50	50	N/A	54	54	5	5	81	104	251
SAUR0638 (SAR_RS03190)	ydiP	675383	66	66	N/A	N/A	N/A	N/A	N/A	66	66	66	6	6	6	6	82	82	82	82	133	6	66	66	6	10	101	136	84
SAUR0639 (SAR_RS03195)	O5HI54	676319	37	37	37	37	37	37	37	37	37	37	6	156	156	6	2	2	2	2	2	6	37	37	6	5	56	2	18
SAUR0640 (SAR_RS03200)	O5HI53	676987	55	55	55	N/A	N/A	55	55	55	55	55	7	180	180	7	N/A	2	2	2	2	7	55	55	6	8	2	2	35
SAUR0641 (SAR_RS03205)	O5HI52	N/A	N/A	N/A	N/A	N/A	N/A	N/A	N/A	N/A	N/A	N/A	7	7	7	7	2	2	2	2	2	N/A	N/A	N/A	7	N/A	N/A	26	N/A
SAUR0642 (SAR_RS03210)	N/A	679128	1	1	1	1	1	1	1	1	1	1	1	1	1	1	1	1	1	1	1	1	1	1	1	1	1	1	1
SAUR0643 (SAR_RS03215)	O2YSV8	680450	7	7	7	N/A	7	7	7	7	7	7	91	91	N/A	91	25	25	25	25	25	N/A	N/A	N/A	83	85	34	41	N/A
SAUR0644 (SAR_RS03220)	N/A	681574	2	2	2	2	2	2	2	2	2	2	7	7	7	7	2	2	2	2	2	7	N/A	N/A	7	10	40	2	2
SAUR0645 (SAR_RS03225)	O7A1N3	681815	2	2	2	2	2	2	2	2	2	2	39	39	39	39	2	2	2	2	2	N/A	2	2	39	40	15	2	2
SAUR0646 (SAR_RS03230)	N/A	682177	50	50	50	50	50	N/A	50	50	50	50	52	52	52	52	67	67	67	67	67	N/A	50	50	52	52	86	67	50
SAUR0647 (SAR_RS03235)	mrpA	682756	736	736	188	188	188	188	N/A	188	188	188	260	260	260	260	426	226	226	226	309	N/A	188	188	100	135	650	312	N/A
SAUR0648 (SAR_RS03240)	mrpB	685145	1	1	1	1	1	1	1	1	1	N/A	40	40	40	40	50	50	50	50	50	N/A	1	1	39	40	73	48	50
SAUR0649 (SAR_RS03245)	mrpC	685567	43	43	N/A	43	43	43	43	43	43	43	26	26	26	26	N/A	24	24	24	24	N/A	43	43	26	28	25	49	24
SAUR0650 (SAR_RS03250)	mrpD	685901	414	414	414	414	414	414	414	414	414	414	175	175	175	175	10	10	10	10	10	513	414	414	5	19	177	10	148
SAUR0651 (SAR_RS03255)	mrpE	687398	32	32	32	32	32	32	32	32	32	32	55	55	55	55	43	43	43	43	43	55	32	32	7	7	56	2	2
SAUR0652 (SAR_RS03260)	N/A	687877	20	20	20	20	20	20	20	20	20	20	7	7	7	7	20	20	20	20	20	N/A	20	20	2	2	30	2	2
SAUR0653 (SAR_RS03265)	N/A	688154	2	2	2	2	2	2	2	2	2	2	N/A	N/A	N/A	N/A	2	2	2	N/A	2	N/A	2	2	N/A	N/A	2	N/A	N/A
SAUR0654 (SAR_RS03270)	nhaK1	688931	119	119	119	119	119	119	119	119	119	119	N/A	177	592	N/A	N/A	154	154	154	154	591	119	119	51	18	178	241	N/A
SAUR0658 (SAR_RS03290)	mntC	691817	290	290	59	59	59	59	59	59	59	59	85	85	85	85	24	24	24	24	24	85	59	59	7	20	86	40	74
SAUR0659 (SAR_RS03295)	mntB	692743	30	30	30	30	30	30	30	30	30	30	7	7	7	7	3	2	2	2	2	7	30	30	7	7	65	30	30
SAUR0660 (SAR_RS03300)	N/A	693573	3	3	3	3	3	3	3	3	3	3	3	3	3	3	20	20	20	20	109	N/A	3	3	3	3	2	3	2
SAUR0661 (SAR_RS03305)	mntR	694438	2	2	2	2	2	2	N/A	2	2	2	80	80	80	80	N/A	69	69	69	69	80	2	2	7	7	81	2	2
SAUR0662 (SAR_RS03310)	O5HI33	695162	9	9	9	N/A	9	9	9	9	9	9	9	9	9	9	3	193	3	3	3	9	9	9	285	25	45	129	45
SAUR0663 (SAR_RS03315)	tarA	696150	303	303	14	14	14	14	14	14	14	14	53	53	53	53	77	77	77	77	77	53	14	14	6	9	14	130	14
SAUR0664 (SAR_RS03320)	tarH=tagH	696975	2	2	2	2	2	2	2	2	2	2	49	49	49	49	2	2	2	72	2	49	2	2	7	30	86	2	2
SAUR0665 (SAR_RS03325)	tarG=tagG	698116	2	2	2	2	2	2	2	2	2	2	N/A	19	19	19	2	2	2	2	2	19	2	2	9	19	68	2	2
SAUR0666 (SAR_RS03330)	tarB	699027	35	35	35	N/A	N/A	35	35	35	35	35	78	78	78	78	N/A	119	119	119	119	78	35	35	371	33	148	2	120
SAUR0667 (SAR_RS03335)	tarX	700127	2	2	2	N/A	N/A	2	2	225	2	2	77	77	77	77	N/A	114	114	114	114	77	2	2	11	32	138	44	115
SAUR0668 (SAR_RS03340)	tarD	701251	3	3	3	3	3	3	3	3	3	3	3	59	59	3	2	2	2	2	2	3	3	3	3	3	3	2	2
SAUR0669 (SAR_RS03345)	[illegible]	701766	38	38	38	38	38	38	38	38	38	N/A	145	540	145	145	120	289	266	120	120	145	38	38	13	130	146	197	121
SAUR0670 (SAR_RS03350)	msbA1	703482	86	86	86	86	86	86	86	86	86	86	185	185	185	185	86	86	86	86	86	185	N/A	N/A	9	N/A	188	243	86
SAUR0671 (SAR_RS03355)	nupG	705568	80	80	80	80	80	80	80	80	80	80	18	306	306	18	108	108	234	108	108	421	80	80	9	31	212	173	2
SAUR0672 (SAR_RS03360)	N/A	707052	1	1	1	N/A	1	1	1	1	1	1	1	1	1	1	1	1	1	1	1	1	1	1	1	1	1	20	1
SAUR0673 (SAR_RS03365)	yxkD	707322	72	72	72	72	72	72	72	72	72	72	112	112	112	112	72	72	72	72	72	112	72	72	9	15	2	143	90
SAUR0674 (SAR_RS03370)	fhuC	708437	44	44	44	44	44	44	N/A	44	44	44	38	38	38	38	144	2	2	2	2	38	44	44	30	19	77	106	62
SAUR0675 (SAR_RS03375)	fhuB	709270	77	77	77	77	77	77	N/A	77	77	77	7	7	7	7	77	77	77	77	77	7	77	77	7	28	124	77	N/A
SAUR0676 (SAR_RS03380)	fhuG	710271	87	87	N/A	N/A	N/A	N/A	N/A	453	87	87	N/A	68	68	68	N/A	87	87	87	188	N	87	87	7	15	407	194	117
SAUR0677 (SAR_RS03385)	dakK=dhaK	711521	2	2	2	2	2	2	2	74	2	74	9	9	9	9	2	2	2	98	2	N/A	2	2	41	52	120	161	70
SAUR0678 (SAR_RS03390)	dakL=dhaL	712531	2	2	2	2	2	2	2	2	2	2	6	6	6	6	2	2	2	2	2	6	2	2	5	9	95	21	2
SAUR0679 (SAR_RS03395)	dakP=dhaM	713108	2	2	N/A	N/A	N/A	N/A	N/A	2	2	2	N/A	6	6	6	2	2	2	2	2	N/A	2	2	N/A	5	2	2	39
SAUR0680 (SAR_RS03400)	O5HI15	713586	12	12	12	12	12	12	12	12	12	12	2	2	2	2	37	37	37	37	37	2	12	12	2	2	10	12	N/A
SAUR0681 (SAR_RS03405)	O5HI14	714540	2	2	2	2	2	N/A	2	2	2	2	8	8	8	8	34	N/A	34	34	132	8	2	2	8	10	156	34	34
SAUR0682 (SAR_RS03410)	lip4	715758	427	427	2	2	2	2	2	2	2	2	122	122	122	122	N/A	108	108	108	108	122	2	2	6	25	123	123	N/A
SAUR0683 (SAR_RS03415)	N/A	716966	2	2	2	2	2	2	2	2	2	2	4	4	4	4	1	1	1	1	1	N/A	2	2	4	5	N/A	N/A	N/A
SAUR0684 (SAR_RS03420)	O5HI12	717131	2	2	2	N/A	2	N/A	2	2	2	2	48	48	48	48	19	19	19	19	19	48	2	2	48	49	23	5	5
SAUR0685 (SAR_RS03425)	O5HI11	717766	29	29	29	29	29	29	29	29	151	29	16	16	16	16	71	167	71	71	71	16	158	158	10	16	28	29	28
SAUR0686 (eraX)	N/A	718385	82	82	82	82	82	82	82	82	82	82	87	87	87	87	82	82	82	82	82	416	82	82	6	6	123	30	N/A
SAUR0687 (eraR)	N/A	719324	N/A	11	11	11	11	11	N/A	11	11	11	20	20	20	20	63	63	63	63	63	N/A	11	11	21	36	16	16	16
SAUR0688 (eraS)	N/A	719991	123	123	123	123	123	123	123	316	123	123	73	73	73	73	144	144	144	144	144	N/A	N/A	N/A	73	75	162	100	100
SAUR0689 (SAR_RS03445)	N/A	721175	107	107	107	107	107	107	107	107	107	107	N/A	150	150	150	N/A	135	135	135	135	N/A	107	107	94	96	133	170	136
SAUR0690 (SAR_RS03450)	vraG	721926	254	254	N/A	N/A	N/A	N/A	N/A	254	254	254	N/A	371	371	793	410	341	341	341	410	N/A	N/A	N/A	714	N/A	373	414	N/A
SAUR0691 (SAR_RS03455)	ykaA=pitR	724540	2	2	2	N/A	2	2	2	2	2	2	6	6	6	6	2	2	2	2	2	6	2	2	5	6	2	2	2
SAUR0692 (SAR_RS03460)	pitA	725173	2	2	2	2	2	N/A	2	2	2	2	81	81	81	81	2	2	2	2	2	81	2	2	7	20	2	N/A	23
SAUR0693 (SAR_RS03465)	ssaA5	726766	65	65	65	N/A	65	65	N/A	65	65	65	52	52	52	52	81	81	81	81	81	366	65	65	6	17	108	45	20
SAUR0694 (SAR_RS03470)	yetI	727924	45	45	45	N/A	45	45	45	45	45	45	11	11	11	11	72	72	72	72	72	257	45	45	8	12	45	72	73
SAUR0695 (SAR_RS03475)	N/A	729265	2	2	2	N/A	2	N/A	2	2	2	2	2	2	2	2	N/A	N/A	N/A	N/A	N/A	N/A	2	2	2	2	N/A	2	N/A
SAUR0696 (SAR_RS03480)	N/A	729472	123	N/A	123	N/A	N/A	N/A	123	123	123	132	21	670	21	21	160	160	365	160	160	N/A	N/A	N/A	9	N/A	34	254	161
SAUR0697 (SAR_RS03485)	sarX	731561	1	1	1	1	1	1	1	60	1	1	3	3	3	3	1	1	1	1	1	3	1	1	3	42	1	1	1
SAUR0698 (SAR_RS03490)	yeeI	732110	24	24	24	24	24	24	24	24	24	24	7	7	7	7	60	60	60	60	60	7	24	24	236	7	2	24	38
SAUR0699 (SAR_RS03495)	DUF985	732826	23	23	23	23	23	23	23	23	23	23	8	8	8	8	N/A	23	23	64	23	8	23	23	6	6	75	23	23
SAUR0701 (SAR_RS03505)	A5IOM5	733652	2	2	2	2	2	2	N/A	2	2	2	6	6	6	6	68	2	2	2	68	N/A	2	2	4	6	2	2	N/A
SAUR0702 (SAR_RS03510)	ccpE	734412	51	51	51	51	51	51	51	51	51	51	78	78	78	78	65	65	65	65	65	78	51	51	7	15	79	43	66
SAUR0703 (SAR_RS03515)	A8Z196	735482	79	79	79	N/A	N/A	79	79	79	79	79	136	136	136	136	109	109	109	109	109	466	79	79	7	N/A	137	182	N/A
SAUR0710 (SAR_RS03550)	N/A	1858497	353	353	353	353	353	353	353	353	353	353	190	190	190	190	N/A	358	358	358	358	N/A	353	353	92	98	N/A	373	359
SAUR0740 (SAR_RS03700)	A5ION0	738102	25	25	25	N/A	25	25	25	25	25	25	17	17	17	17	25	25	25	25	25	17	25	25	6	12	N/A	85	25
SAUR0741 (SAR_RS03705)	A8YZU8	738613	116	116	116	116	116	N/A	116	116	116	116	48	48	48	48	146	N/A	146	146	146	N/A	116	116	12	6	38	144	N/A
SAUR0742 (SAR_RS03710)	O5HHZ0	739122	29	29	29	29	29	29	29	29	29	29	8	8	8	8	N/A	37	56	37	56	8	29	29	8	12	46	29	1
SAUR0743 (SAR_RS03715)	ykkB	739501	2	2	2	2	2	2	2	60	2	60	86	86	86	86	52	52	52	52	52	86	2	2	8	19	95	2	24
SAUR0744 (SAR_RS03720)	yydD	740138	54	54	54	54	54	54	54	54	54	54	80	80	80	80	71	71	71	71	71	80	54	54	6	10	81	107	N/A
SAUR0745 (SAR_RS03725)	yqxD	740706	47	47	47	N/A	N/A	47	N/A	47	47	47	70	70	204	70	23	23	23	23	23	70	47	47	7	7	76	2	2
SAUR0746 (SAR_RS03730)	O5HHY6	741167	61	61	61	61	61	61	61	61	61	61	99	99	99	99	80	80	80	80	80	297	N/A	N/A	9	9	100	25	2
SAUR0747 (SAR_RS03735)	uppP	742021	65	65	N/A	N/A	N/A	N/A	N/A	297	65	65	158	158	158	158	76	176	76	76	76	158	65	65	6	11	26	20	26
SAUR0748 (SAR_RS03740)	cydD	743115	678	678	128	N/A	128	128	128	128	325	128	191	705	191	191	160	160	160	160	160	191	128	128	10	34	192	251	80
SAUR0749 (SAR_RS03745)	cydC	744743	133	133	133	133	133	133	N/A	133	133	133	209	742	209	209	172	172	172	172	172	209	438	438	10	11	213	286	173
SAUR0750 (SAR_RS03750)	N/A	746543	1	1	1	1	1	1	1	1	1	1	N/A	5	5	N/A	1	1	1	1	1	5	1	1	5	5	47	1	1
SAUR0751 (SAR_RS03755)	cobW2	747213	77	77	77	N/A	N/A	77	77	77	77	77	12	12	12	12	32	32	32	32	32	12	77	77	9	N/A	32	159	N/A
SAUR0752 (SAR_RS03760)	ycsN	748242	85	N/A	85	85	85	85	85	80	85	80	N/A	225	225	N/A	N/A	105	171	105	171	225	85	85	8	17	135	173	106
SAUR0753 (SAR_RS03765)	O5HHX9	749185	1	1	1	1	1	1	1	1	1	1	8	8	8	8	1	1	1	1	1	8	1	1	5	13	1	1	14
SAUR0754 (SAR_RS03770)	yflS	749755	124	124	124	124	124	124	124	124	N/A	124	N/A	59	59	N/A	375	264	264	169	264	59	N/A	N/A	8	177	597	269	170
SAUR0755 (SAR_RS03775)	nhrB	751394	531	531	531	531	531	531	531	531	572	531	N/A	280	280	N/A	621	621	621	621	621	N/A	531	531	5	N/A	N/A	617	624
SAUR0756 (SAR_RS03780)	O2G0A5	752849	144	144	89	89	89	89	89	89	89	89	11	11	11	11	89	89	89	89	89	N/A	89	89	5	11	89	91	89
SAUR0757 (SAR_RS03785)	O5HHX6	753130	167	167	167	167	167	N/A	N/A	174	167	174	55	55	55	55	N/A	166	166	166	166	N/A	167	167	5	15	166	169	166
SAUR0758 (SAR_RS03790)	O5HHX5	N/A	N/A	N/A	222	N/A	N/A	222	N/A	N/A	N/A	N/A	9	9	9	9	2	2	2	2	2	181	222	222	9	9	N/A	N/A	N/A
SAUR0759 (SAR_RS03795)	norA	754566	220	220	220	220	220	220	220	220	220	220	175	175	175	175	191	191	191	191	191	175	220	220	171	171	253	282	N/A
SAUR0760 (SAR_RS03800)	yedL	756028	42	42	42	42	42	42	42	42	42	42	62	62	62	62	20	20	20	20	20	62	42	42	6	8	63	20	20
SAUR0761 (SAR_RS03805)	ybaK	756654	8	8	8	8	8	8	8	8	8	8	69	69	69	69	8	8	8	8	8	N/A	8	8	36	19	71	61	61
SAUR0762 (SAR_RS03810)	fruR	757389	2	2	2	2	2	2	2	2	2	2	10	10	308	10	16	16	16	16	16	10	2	2	7	10	98	78	78
SAUR0763 (SAR_RS03815)	fruB=pfkB	758147	84	84	N/A	N/A	N/A	N/A	84	84	84	84	122	122	122	122	109	109	109	109	109	122	84	84	5	6	23	159	110
SAUR0764 (SAR_RS03820)	fruA	759073	41	41	41	41	41	41	N/A	41	N/A	41	304	683	304	304	N/A	59	59	59	59	N/A	41	41	198	224	559	104	102
SAUR0766 (SAR_RS03830)	nagA	761345	30	30	30	30	30	30	30	30	30	30	N/A	123	123	123	100	100	100	100	100	123	N/A	N/A	33	55	130	161	101
SAUR0767 (SAR_RS03835)	corC=mpfA	762746	75	75	75	75	75	75	75	75	75	75	106	106	106	N/A	88	88	88	88	88	106	N/A	N/A	5	19	69	133	89
SAUR0768 (SAR_RS03840)	yveN1	764317	64	64	64	64	64	64	64	64	64	64	103	103	103	103	87	87	183	87	87	103	64	64	6	22	104	136	56
SAUR0769 (SAR_RS03845)	csbB	765288	77	77	77	77	77	77	77	77	77	77	N/A	46	46	N/A	103	216	103	103	153	46	77	77	291	28	121	60	N/A
SAUR0770 (saeS)	N/A	766343	N/A	70	70	N/A	70	70	70	70	70	70	15	457	15	15	96	96	96	96	96	430	70	70	15	20	125	2	125
SAUR0771 (saeR)	N/A	767398	2	2	2	2	2	2	2	2	2	2	N/A	2	2	N/A	2	2	2	2	2	2	2	2	2	2	2	2	2
SAUR0772 (saeQ)	N/A	768059	37	37	N/A	N/A	N/A	N/A	N/A	37	37	37	7	7	7	7	2	2	2	2	2	N/A	37	37	7	7	2	2	44
SAUR0773 (saeP)	N/A	768875	43	43	43	43	43	43	43	43	97	43	10	10	10	10	N/A	5	5	5	5	10	43	43	9	15	69	14	2
SAUR0775 (SAR_RS03875)	queE	770278	66	N/A	66	N/A	66	66	66	66	66	66	88	88	88	88	27	27	27	27	27	88	66	66	8	15	27	113	79
SAUR0776 (SAR_RS03880)	queD	770995	21	21	21	21	21	21	21	21	21	21	158	5	5	158	21	21	21	21	21	5	N/A	N/A	5	15	21	1	52
SAUR0777 (SAR_RS03885)	queC	771416	48	48	48	48	48	48	48	48	48	48	5	5	5	5	69	69	69	69	69	5	48	48	5	12	22	117	70
SAUR0778 (SAR_RS03890)	pabA	772435	25	25	25	25	25	25	25	25	25	25	9	9	9	9	2	2	2	2	2	9	N/A	N/A	9	15	83	25	50
SAUR0779 (SAR_RS03895)	pabB	773012	84	84	84	84	84	84	84	84	84	84	127	127	127	127	107	107	107	107	107	N/A	84	84	7	13	129	174	439
SAUR0780 (SAR_RS03900)	pabC	774163	57	57	57	57	57	57	57	57	57	57	N/A	87	87	N/A	69	69	141	69	69	N/A	57	57	16	22	235	111	70
SAUR0781 (SAR_RS03905)	O6GIS6	774837	31	31	31	31	31	31	31	31	63	31	41	41	41	41	38	38	38	38	38	N/A	31	31	6	7	31	2	N/A
SAUR0782 (SAR_RS03910)	ahs1	775131	65	65	65	65	65	65	65	65	65	65	N/A	324	102	N/A	55	55	55	55	55	N/A	65	65	245	7	105	55	55
SAUR0783 (SAR_RS03915)	ahs2	775825	82	82	82	82	82	82	N/A	82	82	82	141	141	443	141	110	110	110	110	110	141	82	82	10	30	33	180	N/A
SAUR0784 (SAR_RS03920)	ltaS	777303	96	96	96	96	96	96	N/A	96	96	96	N/A	159	159	N/A	303	328	130	130	130	159	N/A	N/A	8	33	170	212	N/A
SAUR0785 (SAR_RS03925)	N/A	779385	3	3	3	3	3	3	3	3	3	3	4	4	4	4	3	3	3	3	3	N/A	3	3	3	4	3	3	3
SAUR0786 (SAR_RS03930)	uup	779520	N/A	N/A	126	126	126	126	126	625	126	126	198	198	198	198	394	394	394	156	272	198	126	126	12	20	211	276	N/A
SAUR0787 (SAR_RS03935)	N/A	781412	94	94	94	94	94	94	N/A	94	94	94	149	499	149	149	115	115	115	115	115	N/A	N/A	N/A	7	12	2	194	116
SAUR0788 (SAR_RS03940)	opuBA	783412	72	72	72	72	72	72	72	72	72	72	176	176	312	176	N/A	88	88	88	88	N/A	72	72	8	11	167	36	89
SAUR0789 (SAR_RS03945)	opuBB	784382	82	82	82	82	82	82	82	82	82	82	124	502	124	124	239	262	99	99	166	124	82	82	10	108	410	169	N/A
SAUR0790 (SAR_RS03950)	hisC2	786136	77	77	77	77	77	77	77	77	77	77	9	397	397	9	97	97	97	97	97	N/A	77	77	7	31	125	155	98
SAUR0791 (SAR_RS03955)	yorS	787592	2	2	2	2	2	2	2	2	2	2	8	8	8	8	N/A	70	70	47	70	8	2	2	7	10	157	17	48
SAUR0792 (SAR_RS03960)	bmrU=dgkA1	788686	69	69	69	69	69	69	69	69	69	69	N/A	109	109	N/A	89	89	89	89	89	109	69	69	6	23	111	146	90
SAUR0794 (SAR_RS03970)	N/A	789885	76	76	76	76	76	76	76	76	76	76	124	453	124	124	103	103	103	103	170	124	76	76	9	26	29	172	N/A
SAUR0795 (SAR_RS03975)	queF	N/A	18	18	18	18	18	18	18	N/A	18	18	5	5	5	5	39	39	39	39	39	5	18	18	92	5	31	73	39
SAUR0796 (SAR_RS03980)	yxxF	792251	30	30	N/A	N/A	N/A	N/A	N/A	30	30	30	N/A	345	88	N/A	N/A	178	57	57	57	88	N/A	N/A	4	5	26	N/A	57
SAUR0797 (SAR_RS03985)	N/A	793918	10	10	10	10	10	10	10	10	10	10	1	1	1	1	2	2	2	2	2	N/A	10	10	2	2	2	10	10
SAUR0798 (SAR_RS03990)	nrdE	794279	45	45	45	45	45	45	45	45	45	45	N/A	252	446	252	187	187	258	121	187	252	45	45	432	41	33	189	122
SAUR0799 (SAR_RS03995)	nrdF	796502	18	18	18	18	18	18	18	18	18	18	6	6	6	6	42	42	90	42	42	146	18	18	6	6	18	18	18
SAUR0801 (SAR_RS04005)	sstA	797843	57	57	57	57	57	N/A	57	57	57	57	7	7	326	7	80	80	80	80	80	N/A	57	57	5	20	103	50	2
SAUR0802 (SAR_RS04010)	sstB	798801	66	66	66	N/A	66	66	N/A	66	166	66	7	7	7	7	86	86	86	86	86	N/A	66	66	7	9	108	135	88
SAUR0803 (SAR_RS04015)	sstC	799754	53	53	53	53	53	53	53	53	53	53	90	90	90	90	N/A	68	68	68	68	90	53	53	6	7	91	116	20
SAUR0804 (SAR_RS04020)	sstD	800634	391	391	71	71	71	71	71	71	71	71	10	378	378	10	91	91	91	91	91	N/A	71	71	10	73	111	138	92
SAUR0805 (SAR_RS04025)	O5HHT3	801923	16	16	16	16	16	16	16	16	16	16	7	7	7	7	9	80	9	9	9	7	16	16	24	7	9	9	37
SAUR0806 (SAR_RS04030)	murB	802255	23	23	23	23	23	23	23	23	23	23	32	32	32	32	90	90	90	90	90	32	23	23	8	17	50	91	91
SAUR0807 (SAR_RS04035)	grpB	803305	36	36	36	N/A	36	36	36	36	36	36	N/A	57	57	N/A	39	39	39	39	39	57	36	36	5	33	2	77	2
SAUR0808 (SAR_RS04040)	O5HHT0	803943	56	56	56	N/A	56	56	N/A	56	56	56	98	98	98	98	75	75	75	75	75	98	56	56	5	6	99	134	76
SAUR0809 (SAR_RS04045)	ytxJ	804975	1	1	1	1	1	1	1	1	1	1	5	5	5	5	1	1	1	1	1	5	1	1	5	5	1	1	1
SAUR0810 (SAR_RS04050)	glxK2	805720	95	95	95	95	95	95	95	95	95	95	N/A	145	145	145	118	118	118	118	118	145	95	95	7	32	147	198	119
SAUR0811 (SAR_RS04055)	pepT	807032	120	120	120	120	120	120	120	120	302	120	N/A	182	182	N/A	152	152	323	152	152	182	120	120	457	41	183	236	153

Locus (cg MLST)	Alternative designation	Position (in ED133)	Study Isolates: Swan isolate 15V8707	Study Isolates: Duck isolate 511525-22	Study Isolates: Duck isolate V315	Study Isolates: Duck isolate 511509-22	Study Isolates: Duck isolate 511510-22	Study Isolates: Whistling duck isolate [illegible]	Study Isolates: Duck isolate V482	CC133: CP001996.1, ED133	CC133: CP065353.1, AR39 [illegible]	CC133: LR134271.1, NCTC7988	CC692: CP042650.1, X22	CC692: UHAK01000002.1, NCTC9546	CC692: PubMLST ID 39948, 973N0056	CC692: CP129360, CKMM-401M	CC522: Goat isolate 17CS1042	CC522: PubMLST ID 24977	CC522: PubMLST ID 24358	CC522: PubMLST ID 11704	CC522: SAMEA1904171	ST1347: PubMLST ID 39945, 61290	Chinese CC133 MRSA isolates: JAOAKU01, GDQ20D75A	Chinese CC133 MRSA isolates: JAFEOM01, GDQ20D75A	CC5: CP001781.1, ED98	ST71: SAMN03247575, MOK-099	CC182: CP070991, CBS2016-05	CC707: CP029751, SMITH	CC1956: CP084892, WT19
SAUR0812 (SAR_RS04060)	Q5HHS6	808272	29	29	29	29	29	29	29	29	29	29	7	7	7	7	2	2	2	2	2	7	29	29	7	20	29	65	2
SAUR0813 (SAR_RS04065)	DUF1212_L2	808784	50	50	50	50	50	50	N/A	50	50	50	13	13	13	13	14	14	14	14	14	13	50	50	6	27	48	50	N/A
SAUR0814 (SAR_RS04070)	gdpS	809741	72	72	N/A	N/A	N/A	N/A	N/A	72	72	72	109	109	109	109	100	100	100	100	100	109	72	72	10	19	2	138	2
SAUR0815 (SAR_RS04075)	tarO	811129	66	66	N/A	66	66	66	66	66	161	66	7	7	7	7	83	83	83	83	83	7	161	161	7	8	272	116	79
SAUR0816 (SAR_RS04080)	Q5HHS2	812356	299	299	52	52	52	52	52	52	52	52	87	87	87	87	73	73	73	73	73	87	52	52	8	23	241	110	70
SAUR0818 (SAR_RS04090)	comEA	814371	89	89	89	89	89	89	89	89	89	89	150	150	150	150	125	125	125	125	125	496	89	89	13	69	153	208	66
SAUR0819 (SAR_RS04095)	comEC	815446	19	19	19	19	19	19	19	19	19	19	18	18	18	18	N/A	92	92	92	168	N/A	19	19	9	9	125	169	24
SAUR0820 (SAR_RS04100)	vfiA=raiA	816181	200	200	13	13	13	13	13	13	13	13	1	1	1	1	1	1	1	1	1	1	13	13	5	1	1	77	1
SAUR0821 (SAR_RS04105)	secA1	817167	128	128	128	128	128	N/A	128	128	413	128	240	240	240	240	186	186	186	186	186	240	N/A	N/A	9	N/A	242	242	N/A
SAUR0822 (SAR_RS04110)	N/A	819740	2	2	N/A	N/A	N/A	N/A	N/A	2	2	2	3	3	3	3	2	2	2	2	2	N/A	2	2	N/A	27	1	1	2
SAUR0823 (SAR_RS04115)	prfB	820126	74	74	74	74	74	74	74	74	74	74	122	122	122	122	99	99	99	99	99	N/A	74	74	11	N/A	18	156	64
SAUR0825 (SAR_RS04125)	lysM	821527	84	84	84	84	84	84	84	84	84	84	139	139	139	139	64	64	64	64	64	N/A	84	84	366	26	140	182	N/A
SAUR0826 (SAR_RS04130)	vfbR	822540	51	51	51	N/A	51	51	51	51	51	51	N/A	9	9	N/A	19	19	19	19	19	9	51	51	6	7	19	19	65
SAUR0827 (SAR_RS04135)	DUF2198	823187	3	3	3	3	3	3	3	3	3	3	N/A	5	5	5	N/A	30	30	30	30	5	3	3	5	5	15	15	15
SAUR0828 (SAR_RS04140)	uvrB	823692	128	128	128	128	128	128	N/A	128	128	N/A	671	204	204	671	164	N/A	164	164	164	N/A	128	128	10	40	217	275	165
SAUR0829 (SAR_RS04145)	uvrA	825685	151	151	N/A	N/A	N/A	N/A	N/A	151	151	151	N/A	509	509	509	211	211	211	211	211	N/A	151	151	13	38	852	368	N/A
SAUR0831 (SAR_RS04155)	hpr=hprK	829127	57	57	N/A	N/A	N/A	N/A	N/A	57	57	57	N/A	6	6	6	75	75	75	75	75	6	N/A	N/A	6	20	2	51	76
SAUR0832 (SAR_RS04160)	lgt	830065	197	197	197	N/A	197	197	197	197	197	197	357	376	92	357	215	215	215	215	215	92	197	197	36	35	218	197	215
SAUR0833 (SAR_RS04165)	yvoF	830912	47	47	47	N/A	47	N/A	47	47	47	47	222	14	14	222	47	47	47	66	47	14	47	47	6	7	193	32	47
SAUR0834 (SAR_RS04170)	yycD	831405	128	128	N/A	N/A	N/A	N/A	N/A	122	128	122	317	317	579	317	150	150	150	150	150	578	N/A	N/A	8	32	188	244	152
SAUR0835 (SAR_RS04175)	trxB	832911	61	61	61	61	61	61	61	61	178	61	N/A	7	7	N/A	84	84	84	84	84	7	61	61	7	20	61	32	N/A
SAUR0836 (SAR_RS04180)	yycJ=rapZ	834846	85	85	N/A	85	85	85	85	85	85	85	N/A	114	114	N/A	199	199	199	101	139	114	85	85	309	N/A	38	2	2
SAUR0837 (SAR_RS04185)	mgfK=yycK	835754	68	68	68	68	68	68	68	68	68	68	7	7	7	7	85	85	85	85	130	7	68	68	6	18	20	133	57
SAUR0838 (SAR_RS04190)	whiA	836860	42	42	42	42	42	42	42	42	42	42	77	77	268	77	42	42	42	42	42	77	42	42	6	9	78	105	70
SAUR0840 (SAR_RS04200)	clpP	838376	28	28	28	28	28	28	28	28	28	28	8	178	8	8	44	101	44	44	44	8	28	28	8	8	25	23	55
SAUR0842 (SAR_RS04210)	vfcH	840986	72	72	N/A	72	72	N/A	72	72	72	72	108	108	108	N/A	90	90	90	90	90	108	72	72	8	15	33	145	236
SAUR0843 (SAR_RS04215)	Q5HHP8	842591	24	24	24	24	24	24	24	24	24	24	94	94	94	94	79	79	79	79	79	94	24	24	9	9	72	122	72
SAUR0844 (SAR_RS04220)	gapR	843978	13	13	N/A	N/A	N/A	N/A	N/A	13	13	N/A	N/A	118	118	N/A	N/A	3	3	3	3	118	13	13	7	7	120	3	N/A
SAUR0845 (SAR_RS04225)	gapA	845044	15	15	N/A	N/A	N/A	N/A	N/A	15	15	15	7	7	7	7	N/A	15	15	15	15	7	N/A	N/A	201	7	72	72	15
SAUR0846 (SAR_RS04230)	pgk	846193	483	483	15	15	15	15	15	15	15	15	44	44	44	44	133	133	133	133	133	44	15	15	385	31	50	190	N/A
SAUR0847 (SAR_RS04235)	tpi	847505	12	12	N/A	12	12	12	12	12	12	12	18	18	18	18	98	98	172	98	98	18	12	12	7	33	98	2	99
tpi	N/A	847817	50	50	N/A	50	50	50	50	50	50	50	5	5	5	5	15	15	15	N/A	15	N/A	50	50	1	5	15	58	225
SAUR0848 (SAR_RS04240)	gpmI	848269	14	14	N/A	14	14	N/A	14	14	14	14	N/A	185	185	N/A	152	152	152	152	152	185	14	14	548	28	197	236	N/A
SAUR0849 (SAR_RS04245)	eno	849916	27	27	27	27	27	27	27	27	27	27	387	283	283	387	88	88	88	88	88	387	27	27	9	27	88	27	52
SAUR0850 (SAR_RS04250)	Q2YSE7	851560	13	13	13	13	13	13	225	13	13	13	N/A	8	8	N/A	51	51	51	51	78	8	13	13	8	8	60	13	13
SAUR0851 (SAR_RS04255)	N/A	852085	3	3	3	3	3	3	3	3	3	3	3	3	3	3	3	3	3	3	3	3	3	3	61	3	3	3	3
SAUR0852 (SAR_RS04260)	est	852447	16	16	16	16	16	16	16	16	16	16	256	368	256	256	39	39	39	39	64	256	16	16	119	121	49	65	N/A
SAUR0853 (SAR_RS04265)	rnr	853221	47	47	47	47	47	N/A	47	47	47	47	N/A	N/A	N/A	N/A	119	119	119	71	119	N	N/A	N/A	231	263	93	121	N/A
SAUR0854 (SAR_RS04270)	ssrP=rapZ	855615	1	1	1	1	1	1	1	1	N/A	1	24	24	24	24	59	59	59	59	59	24	1	1	24	17	70	21	60
SAUR0856 (SAR_RS04280)	Q1YB79	857006	306	306	N/A	106	N/A	N/A	N/A	106	106	N/A	N/A	29	29	29	N/A	N/A	N/A	N/A	N/A	N/A	106	106	9	3	27	27	64
SAUR0857 (SAR_RS04285)	N/A	N/A	N/A	N/A	N/A	N/A	N/A	N/A	N/A	N/A	N/A	N/A	6	6	6	6	N/A	N/A	N/A	N/A	N/A	N/A	N/A	N/A	6	7	N/A	N/A	N/A
SAUR0858 (SAR_RS04290)	Q5HHN0	858329	22	22	N/A	N/A	N/A	N/A	N/A	22	22	22	15	15	15	15	59	59	59	59	59	N/A	22	22	6	N/A	74	18	227
SAUR0859 (SAR_RS04295)	Q1XZI1	858989	14	14	14	14	14	14	14	14	14	14	9	9	9	9	85	85	85	85	85	9	14	14	9	11	85	76	86
SAUR0862 (SAR_RS04310)	emp	864665	17	17	17	N/A	N/A	17	17	17	17	17	125	125	125	N/A	25	72	67	25	25	N/A	17	17	107	123	180	204	168
SAUR0864 (SAR_RS04320)	nuc1	866882	15	15	15	15	15	15	15	15	15	15	N/A	25	25	25	15	193	15	15	15	25	15	15	6	25	100	128	81
SAUR0865 (SAR_RS04325)	N/A	867925	1	1	1	1	1	1	1	1	1	1	1	1	1	1	1	1	1	1	1	1	1	1	1	1	1	1	1
SAUR0866 (SAR_RS04330)	Q1Y1Z8	868624	2	2	N/A	2	2	N/A	2	2	2	2	24	24	24	58	N/A	N/A	N/A	N/A	N/A	24	2	2	4	9	59	67	50
SAUR0867 (SAR_RS04335)	Q2G007	868904	36	36	36	36	36	36	36	36	36	36	42	42	42	42	1	1	1	1	1	N/A	36	36	6	10	43	N/A	1
SAUR0868 (SAR_RS04340)	Q1YB69	869276	62	62	62	62	62	N/A	62	62	62	62	8	8	8	8	N/A	29	29	29	29	8	62	62	8	26	104	124	56
SAUR0869 (SAR_RS04345)	Q6GIJ6	870033	9	9	9	N/A	9	N/A	9	9	9	9	33	33	33	33	27	27	27	27	27	N/A	9	9	3	3	34	27	7
SAUR0870 (SAR_RS04350)	Q6GIJ5	870250	11	11	11	11	11	N/A	11	11	11	11	3	3	3	3	3	3	3	3	3	3	11	11	3	7	39	2	2
SAUR0871 (SAR_RS04355)	N/A	870851	1	1	1	1	1	1	1	1	1	1	1	1	1	1	11	11	11	11	11	1	1	1	1	1	1	1	11
SAUR0872 (SAR_RS04360)	Q6GIJ3	871051	1	1	1	1	1	1	1	1	1	1	3	3	3	3	1	1	1	1	1	3	1	1	4	3	29	1	1
SAUR0873 (SAR_RS04365)	gpmA1=cobC	871512	59	59	59	N/A	N/A	59	59	59	59	59	87	87	87	87	76	76	76	76	76	87	59	59	6	13	263	59	12
SAUR0874 (SAR_RS04370)	visU1	872166	65	65	65	65	65	65	65	65	65	65	N/A	97	97	N/A	76	76	174	76	76	97	65	65	7	19	76	126	76
[illegible]	[illegible]	[illegible]	[illegible]	[illegible]	[illegible]	[illegible]	[illegible]	[illegible]	[illegible]	[illegible]	[illegible]	[illegible]	[illegible]	[illegible]	[illegible]	[illegible]	[illegible]	[illegible]	[illegible]	[illegible]	[illegible]	[illegible]	[illegible]	[illegible]	[illegible]	[illegible]	[illegible]	[illegible]	[illegible]

Locus (cg MLST)	Alternative designation	Position (in ED133)	Study Isolates							CC133		
			Swan isolate 15V8707	Duck isolate 511525-22	Duck isolate V315	Duck isolate 511509-22	Duck isolate 511510-22	Whistling duck isolate [illegible]	Duck isolate V482	CP001996.1, ED133	CP065353.1, AR39 [illegible]	LR134271.1, NCTC7988
SAUR1135 (SAR_RS05675)	pheS	1158568	77	77	77	77	77	77	77	77	77	77
SAUR1136 (SAR_RS05680)	pheT	1159626	154	154	154	154	154	154	N/A	867	491	154
SAUR1137 (SAR_RS05685)	rnhC	1162586	381	381	N/A	N/A	N/A	N/A	N/A	79	79	79
SAUR1138 (SAR_RS05690)	zapA	1163899	2	2	2	2	2	2	2	2	2	2
SAUR1139 (SAR_RS05695)	yshB	1164166	47	47	N/A	47	47	47	47	47	47	47
SAUR1140 (SAR_RS05700)	polX	1164760	107	107	107	107	107	107	107	107	107	107
SAUR1141 (SAR_RS05705)	mutS2	1166482	849	849	85	N/A	85	85	85	85	85	309
SAUR1142 (SAR_RS05710)	trxA1	1169003	14	14	14	14	14	14	14	14	14	14
SAUR1143 (SAR_RS05715)	N/A	1169380	8	8	8	8	8	8	8	8	8	8
SAUR1144 (SAR_RS05720)	uvrC	1169641	N/A	650	102	102	102	102	N/A	102	102	102
SAUR1145 (SAR_RS05725)	sdhC	1171746	47	47	N/A	N/A	N/A	N/A	N/A	47	47	47
SAUR1146 (SAR_RS05730)	sdhA	1172412	97	97	97	97	97	97	97	97	97	97
SAUR1147 (SAR_RS05735)	sdhB	1174178	265	265	50	50	50	50	50	248	50	50
SAUR1148 (SAR_RS05740)	murI	1175232	25	25	25	25	25	25	N/A	25	25	25
SAUR1149 (SAR_RS05745)	ntpA	1176044	21	21	21	21	21	21	21	21	21	21
SAUR1150 (SAR_RS05750)	ysnB	1176624	17	17	17	17	17	17	17	17	17	17
SAUR1151 (SAR_RS05755)	Q6GA61	1177256	6	N/A	6	N/A	6	6	6	6	6	6
SAUR1152 (SAR_RS05760)	ecb	1177617	27	27	27	27	N/A	27	27	27	27	27
SAUR1155 (SAR_RS05775)	Q2YXB9	1179377	15	15	15	N/A	N/A	15	15	15	15	15
SAUR1156 (SAR_RS05780)	efb=fib	1180142	19	19	19	19	19	19	19	19	19	N/A
SAUR1157 (SAR_RS05785)	scc	1180793	13	13	13	13	13	13	13	13	13	13
SAUR1158 (SAR_RS05790)	A5IS45	1181858	18	18	N/A	N/A	N/A	N/A	18	18	18	18
SAUR1161 (SAR_RS05805)	hla	1183375	66	66	66	N/A	N/A	N/A	66	66	66	66
SAUR1162 (SAR_RS05810)	A6U0Y3	1185007	18	18	18	N/A	N/A	N/A	18	18	18	18
SAUR1163 (SAR_RS05815)	Q2FZB4	1185137	12	12	12	N/A	N/A	12	12	12	12	12
SAUR1165 (SAR_RS05825)	setB3	1185893	96	96	96	96	96	96	96	96	96	96
SAUR1166 (SAR_RS05830)	setB2	1186717	79	79	79	79	79	79	79	79	79	79
SAUR1167 (SAR_RS05835)	setB1	1187539	93	93	93	93	93	N/A	93	93	168	93
SAUR1168 (SAR_RS05840)	arcB-L1	1188702	47	47	47	47	47	47	47	47	47	47
SAUR1169 (SAR_RS05845)	arcC-L1	1189726	N/A	75	75	75	75	75	75	75	199	75
SAUR1170 (SAR_RS05850)	arcD-L1	1190829	118	118	118	118	118	118	118	118	118	118
SAUR1171 (SAR_RS05855)	A5IS57	1192694	30	30	30	30	30	30	30	30	30	30
SAUR1172 (SAR_RS05860)	per	1193237	79	79	79	79	79	79	79	79	79	79
SAUR1173 (SAR_RS05865)	N/A	N/A	N/A	N/A	N/A	N/A	N/A	N/A	N/A	N/A	N/A	N/A
SAUR1175 (psmβ1)	psmB1	1195530	1	1	1	1	1	1	1	1	1	1
SAUR2947 (psmβ2)	psmB2	1195721	1	1	1	1	1	1	1	1	1	1
SAUR1176 (SAR_RS05880)	Q1Y2B3	1195988	70	70	N/A	N/A	N/A	N/A	N/A	310	70	67
SAUR1177 (SAR_RS05885)	N/A	1196782	2	2	2	2	2	2	2	29	2	29
SAUR1178 (SAR_RS05890)	bshC	1197417	142	142	142	142	142	142	N/A	801	142	142
SAUR1179 (SAR_RS05895)	mraZ	1199174	1	1	1	1	1	1	1	1	1	1
SAUR1180 (SAR_RS05900)	N/A	1199621	37	37	37	37	37	37	37	37	37	37
SAUR1181 (SAR_RS05905)	N/A	1200570	1	1	1	1	1	1	1	1	1	1
SAUR1182 (SAR_RS05910)	pbpA	1200952	176	176	N/A	N/A	N/A	N/A	N/A	176	N/A	176
SAUR1183 (SAR_RS05915)	mraY	1203478	78	78	78	78	78	78	78	78	78	78
SAUR1184 (SAR_RS05920)	murD	1204445	108	108	108	108	108	108	108	108	108	108
SAUR1185 (SAR_RS05925)	ftsQ	1205810	2	2	2	2	2	2	2	2	2	2
SAUR1186 (SAR_RS05930)	ftsA	1207234	64	64	64	64	64	N/A	64	64	64	64
SAUR1187 (SAR_RS05935)	ftsZ	1208673	2	2	2	2	2	2	2	2	2	2
SAUR1188 (SAR_RS05940)	ylmD	1210103	2	2	2	2	2	2	2	2	2	2
SAUR1189 (SAR_RS05945)	ylmE	1210912	2	2	2	N/A	2	2	2	2	N/A	2
SAUR1190 (SAR_RS05950)	sepF	1211592	2	2	2	2	2	2	2	2	2	2
SAUR1191 (SAR_RS05955)	ylmG	1212158	29	29	29	29	29	29	29	29	29	29
SAUR1192 (SAR_RS05960)	ylmH	1212531	69	69	N/A	69	69	N/A	69	69	69	69
SAUR1194 (SAR_RS05970)	ileS	1214199	167	167	N/A	N/A	N/A	N/A	N/A	167	167	167
SAUR1196 (SAR_RS05980)	PF00903=catE	1217243	82	82	82	82	82	82	N/A	82	82	82
SAUR1197 (SAR_RS05985)	lspA	1219622	1	1	1	1	1	1	1	160	1	1
SAUR1198 (SAR_RS05990)	ylyB	1220113	25	25	25	25	25	25	25	25	25	25
SAUR1199 (SAR_RS05995)	pyrR	1221428	42	42	42	42	42	42	42	173	42	42
SAUR1200 (SAR_RS06000)	pyrP	1222173	109	109	109	109	109	109	N/A	109	109	109
SAUR1201 (SAR_RS06005)	pyrB	1223508	378	378	N/A	N/A	N/A	N/A	N/A	48	48	48
SAUR1202 (SAR_RS06010)	pyrC	1224407	96	96	96	96	96	96	96	96	96	96
SAUR1203 (SAR_RS06015)	pyrAA	1225683	117	117	117	117	117	117	N/A	69	117	69
SAUR1204 (SAR_RS06020)	pyrAB	1226776	174	174	174	174	174	174	174	174	N/A	174
SAUR1205 (SAR_RS06025)	pyrF	1230059	66	66	66	66	66	66	66	66	66	66
SAUR1206 (SAR_RS06030)	pyrE	1230751	43	43	43	43	43	43	43	43	43	43
SAUR1207 (SAR_RS06035)	Q5HGM6	1231392	1	1	1	1	1	1	1	1	1	1
SAUR1208 (SAR_RS06040)	Q5HGM5	1232042	49	49	49	49	49	49	49	49	49	49
SAUR1209 (SAR_RS06045)	fbpA	1232759	129	129	129	129	129	129	129	636	129	129
SAUR1210 (SAR_RS06050)	gmk	1234731	39	39	39	39	39	39	39	39	39	39
gmk	N/A	1234803	2	2	2	2	2	2	2	2	2	2
SAUR1211 (SAR_RS06055)	rpoZ	1235354	1	1	1	1	1	1	1	1	1	1
SAUR1212 (SAR_RS06060)	coaBC	1235788	78	78	78	78	78	78	78	78	78	78
SAUR1213 (SAR_RS06065)	priA	1236987	823	823	N/A	126	126	126	N/A	126	126	126
SAUR1214 (SAR_RS06070)	Q5HGL9	1239896	53	53	53	53	53	53	53	53	53	53
SAUR1215 (SAR_RS06075)	Q5HGL8	1241012	2	2	2	2	2	2	2	2	2	2
SAUR1216 (SAR_RS06080)	defA=def2	1241514	23	23	23	N/A	N/A	23	23	23	105	23
SAUR1217 (SAR_RS06085)	fmt	1241995	2	2	N/A	2	2	N/A	2	2	2	2
SAUR1218 (SAR_RS06090)	rsmB=sun	1242927	111	111	111	111	111	111	N/A	111	111	111
SAUR1219 (SAR_RS06095)	rlmN	1244237	16	16	16	16	16	16	16	16	16	16
SAUR1220 (SAR_RS06100)	proC=stp1	1245338	67	67	N/A	N/A	N/A	N/A	N/A	67	67	67
SAUR1221 (SAR_RS06105)	prkC=pknB	1246078	140	140	N/A	N/A	N/A	N/A	N/A	140	140	140
SAUR1222 (SAR_RS06110)	cpgA=engC=rsgA	1248300	69	69	N/A	N/A	N/A	N/A	N/A	320	69	N/A
SAUR1223 (SAR_RS06115)	rpe	1249176	241	241	2	2	2	2	2	2	2	2
SAUR1224 (SAR_RS06120)	thiN	1249827	43	43	N/A	N/A	N/A	N/A	43	43	43	43
SAUR1225 (SAR_RS06125)	N/A	1250849	1	1	1	1	1	1	1	1	1	1
SAUR1226 (SAR_RS06130)	ylqU	1251480	1	1	1	1	1	1	1	1	1	1
SAUR1227 (SAR_RS06135)	ylqV=fakA	1251869	669	N/A	131	131	131	131	N/A	131	131	131
SAUR1228 (SAR_RS06140)	recG	1253705	246	246	246	246	246	246	246	144	246	144
SAUR1229 (SAR_RS06145)	N/A	1255983	44	44	44	44	44	44	44	44	44	44
SAUR1230 (SAR_RS06150)	plsX	1256545	62	62	62	62	62	62	N/A	62	62	62
SAUR1231 (SAR_RS06155)	fabD	1257524	117	117	N/A	117	117	N/A	117	117	117	117
SAUR1232 (SAR_RS06160)	fabG1	1258443	22	22	22	22	22	22	N/A	22	22	22
SAUR1233 (SAR_RS06165)	N/A	N/A	1	N/A	N/A	N/A	N/A	N/A	N/A	N/A	N/A	N/A
SAUR1234 (SAR_RS06170)	N/A	1259610	1	1	1	1	1	1	1	1	1	1
SAUR1235 (SAR_RS06175)	rnc	1259959	46	46	46	46	46	46	46	46	46	46
SAUR1236 (SAR_RS06180)	smc	1260837	210	210	N/A	N/A	N/A	N/A	N/A	217	210	217
SAUR1237 (SAR_RS06185)	ftsY	1264403	103	103	N/A	N/A	N/A	N/A	N/A	103	103	103
SAUR1238 (SAR_RS06190)	ylxM	1265640	7	7	7	7	7	7	7	7	7	7
SAUR1239 (SAR_RS06195)	ffh	1265998	88	88	88	88	88	88	88	437	88	88
SAUR1240 (SAR_RS06200)	rpsP	1267707	1	1	1	1	1	1	1	1	1	1
SAUR1241 (SAR_RS06205)	rimM	1268170	48	48	48	48	48	48	48	48	48	48
SAUR1242 (SAR_RS06210)	N/A	1268673	26	26	26	26	26	26	26	26	26	26
SAUR1243 (SAR_RS06215)	rplS	1269513	8	8	8	8	8	8	8	8	8	8
SAUR1245 (SAR_RS06225)	yfhO	1270277	1000	1000	156	156	156	156	156	868	156	156
SAUR1246 (SAR_RS06230)	rbgA=ylqF	1273284	19	19	19	19	19	19	N/A	19	19	19
SAUR1247 (SAR_RS06235)	rnhB	1274152	67	67	67	67	67	67	67	67	67	67
SAUR1248 (SAR_RS06240)	sucC	1275028	76	76	76	76	76	76	76	76	76	76
SAUR1249 (SAR_RS06245)	sucD	1276216	36	36	N/A	N/A	N/A	N/A	36	36	36	36
SAUR1253 (SAR_RS06265)	topA	1280058	121	121	121	121	121	121	121	121	121	121
SAUR1254 (SAR_RS06270)	trmFO=gid	1282283	2	2	2	2	2	N/A	2	441	2	2
SAUR1255 (SAR_RS06275)	xerC	1284007	2	2	2	2	2	2	2	78	2	78
SAUR1256 (SAR_RS06280)	hslV	1284900	2	2	2	2	2	2	2	2	2	2
SAUR1257 (SAR_RS06285)	hslU	1285511	117	117	117	117	117	117	117	117	337	117
SAUR1258 (SAR_RS06290)	codY	1286939	64	64	64	64	64	64	64	64	64	64
SAUR1259 (SAR_RS06295)	N/A	1287763	2	2	2	2	2	2	2	2	2	2
SAUR1260 (SAR_RS06300)	rpsB	1288054	1	1	1	1	1	1	83	1	1	1
SAUR1261 (SAR_RS06305)	N/A	1288855	2	2	2	2	2	2	N/A	2	2	2
SAUR1262 (SAR_RS06310)	tsf	1289003	1	1	1	1	1	1	1	1	1	1
SAUR1263 (SAR_RS06315)	pyrH	1290021	40	40	40	40	40	40	40	40	40	40
SAUR1264 (SAR_RS06320)	frr	1290762	16	16	16	16	16	16	16	16	16	16
SAUR1265 (SAR_RS06325)	uppS	1291689	1	1	1	1	1	1	1	1	1	1
SAUR1266 (SAR_RS06330)	cdsA	1292466	27	27	27	27	27	27	27	27	27	27
SAUR1267 (SAR_RS06335)	rseP	1293460	100	100	100	100	100	100	100	462	100	100
SAUR1268 (SAR_RS06340)	proS	1294766	115	115	115	115	115	115	115	115	115	115
SAUR1269 (SAR_RS06345)	N/A	1296727	207	207	N/A	N/A	N/A	N/A	N/A	207	207	207
SAUR1270 (SAR_RS06350)	rimP	1301333	42	42	42	42	42	42	N/A	42	42	42
SAUR1271 (SAR_RS06355)	nusA	1301821	86	86	86	86	86	86	86	84	86	84
SAUR1272 (SAR_RS06360)	ylxR	1303017	2	2	2	N/A	2	2	2	2	2	2
SAUR1273 (SAR_RS06365)	ylxQ	1303298	31	31	31	31	31	31	31	31	31	31
SAUR1274 (SAR_RS06370)	infB	1303620	140	140	N/A	N/A	N/A	N/A	N/A	140	140	140
SAUR1275 (SAR_RS06375)	rbfA	1306123	2	2	2	2	2	2	2	2	2	2
SAUR1276 (SAR_RS06380)	truB	1306643	375	375	25	25	25	25	25	25	25	25
SAUR1277 (SAR_RS06385)	ribF	1307575	N/A	119	119	119	119	119	119	381	119	81
SAUR1278 (SAR_RS06390)	rpsO	1308661	1	1	1	1	1	1	N/A	1	1	1
SAUR1279 (SAR_RS06395)	pnpA	1309299	740	740	N/A	N/A	N/A	N/A	N/A	119	119	119
SAUR1280 (SAR_RS06400)	rniB	1311632	120	120	N/A	N/A	N/A	N/A	120	120	120	120
SAUR1281 (SAR_RS06405)	N/A	1313562	138	138	N/A	N/A	N/A	N/A	N/A	138	138	146
SAUR1282 (SAR_RS06410)	vmfC	1315935	26	26	26	26	26	26	26	26	26	26
SAUR1283 (SAR_RS06415)	vmfF	1316679	537	537	89	89	N/A	89	89	89	89	89
SAUR1284 (SAR_RS06420)	vmfH	1317944	115	115	N/A	N/A	N/A	N/A	N/A	115	115	115
SAUR1285 (SAR_RS06425)	fabG2	1319230	57	57	57	57	57	57	57	57	57	57
SAUR1286 (SAR_RS06430)	vmfK	1320039	53	53	53	53	53	53	53	53	53	53
SAUR1287 (SAR_RS06435)	vmfM	1320885	2	2	2	2	2	2	2	2	2	2
SAUR1288 (SAR_RS06440)	pgsA	1321311	37	37	37	37	37	N/A	37	37	37	37
SAUR1289 (SAR_RS06445)	cinA	1322116	92	92	92	92	92	92	92	92	92	92
SAUR1290 (SAR_RS06450)	recA	1323433	62	62	62	62	62	62	62	62	62	62
SAUR1291 (SAR_RS06455)	rny	1324830	18	18	18	18	18	18	18	18	18	18
SAUR1292 (SAR_RS06460)	Q5HGE4	1326686	1	1	1	1	1	1	1	1	1	1
SAUR1293 (SAR_RS06465)	vmdB	1327075	59	N/A	59	59	59	59	59	262	59	59
SAUR1294 (SAR_RS06470)	porA	1328012	121	121	121	121	121	121	121	121	121	121
SAUR1295 (SAR_RS06475)	porB	1329773	47	47	47	N/A	47	47	N/A	47	47	47
SAUR1296 (SAR_RS06480)	DUF77	1330734	2	2	2	2	2	2	2	39	N/A	39
SAUR1297 (SAR_RS06485)	miaB	1331161	2	2	2	2	2	2	N/A	2	2	2
SAUR1298 (SAR_RS06490)	vmcA	1332706	1	1	1	126	126	1	1	1	1	1
SAUR1299 (SAR_RS06495)	thiW	1333098	244	244	2	N/A	2	2	2	42	2	42
SAUR1300 (SAR_RS06500)	mutS	1333892	138	138	N/A	N/A	N/A	N/A	N/A	138	138	138
SAUR1302 (SAR_RS06510)	N/A	1338538	2	2	2	2	2	2	N/A	2	112	2
SAUR1303 (SAR_RS06515)	glpF	1339555	58	58	N/A	N/A	N/A	N/A	N/A	381	58	58
glpF	N/A	1339654	46	46	46	46	46	46	46	46	46	46
SAUR1304 (SAR_RS06520)	glpK	1340503	97	97	97	N/A	N/A	97	97	97	267	97
SAUR1305 (SAR_RS06525)	glpD	1342157	34	34	34	34	34	34	34	34	34	34
SAUR1306 (SAR_RS06530)	pldB2	1343980	415	415	28	N/A	28	28	28	28	195	28
SAUR1308 (SAR_RS06540)	hfq	1345862	5	5	5	5	5	5	5	5	5	5
SAUR1309 (SAR_RS06545)	gpxA-L1	1346317	14	14	14	14	14	14	14	14	14	14
SAUR1310 (SAR_RS06550)	ynbA=hflX	1346905	103	103	N/A	N/A	N/A	N/A	N/A	97	103	97
SAUR1311 (SAR_RS06555)	ynbB	1348162	93	93	93	93	93	93	93	415	93	93
SAUR1312 (SAR_RS06560)	N/A	1349646	1	1	1	1	1	1	1	1	1	1
SAUR1313 (SAR_RS06565)	glnA	1350030	27	27	27	27	27	27	N/A	27	27	27
SAUR1352 (SAR_RS06760)	A6QGL8	1360505	84	84	84	N/A	N/A	84	84	462	84	84
SAUR1354 (SAR_RS06770)	cls1	1362055	44	N/A	44	44	44	44	44	44	N/A	44
SAUR2949 (MW1206)	N/A	1363717	17	17	17	N/A	17	17	17	17	17	N/A
SAUR2950 (MW1207)	N/A	1364585	45	45	45	45	45	45	45	45	45	45
SAUR2951 (desK)	N/A	1365320	56	56	56	N/A	N/A	N/A	56	380	56	56
SAUR2952 (desR)	N/A	1366408	3	3	3	3	3	3	3	3	3	3
SAUR1359 (SAR_RS06795)	Q99UF3	1367123	26	26	26	26	26	26	26	26	26	26
SAUR1360 (SAR_RS06800)	nuc2	1367450	2	2	2	2	2	2	2	2	2	2
SAUR1361 (SAR_RS06805)	Q5HG96	1368126	56	56	56	56	56	56	56	56	56	56
SAUR1362 (SAR_RS06810)	Q5HG95	1369311	233	233	22	N/A	22	22	22	205	22	22
SAUR1363 (SAR_RS06815)	thrA	1369940	120	120	120	120	120	120	120	120	120	120
SAUR1364 (SAR_RS06820)	hom	1371513	85	85	85	85	85	85	85	85	85	85
SAUR1365 (SAR_RS06825)	thrC	1372799	38	38	38	38	38	38	38	38	38	38
SAUR1366 (SAR_RS06830)	thrB	1373862	63	63	63	63	63	63	63	63	63	63
SAUR1367 (SAR_RS06835)	yxeH	1374834	61	61	61	61	61	61	61	61	61	68
SAUR1368 (SAR_RS06840)	Q5HG88	1375928	1	1	1	1	1	1	1	1	1	1
SAUR1369 (SAR_RS06845)	N/A	1376217	2	2	2	2	2	N/A	2	2	2	2
SAUR1370 (SAR_RS06850)	lysP2=Q5HG87	1376460	74	74	74	74	74	74	74	476	74	74
SAUR1371 (SAR_RS06855)	katA	1378118	98	98	98	98	98	98	98	596	98	98
SAUR1372 (SAR_RS06860)	N/A	1379726	2	2	2	2	2	2	N/A	2	2	2
SAUR1373 (SAR_RS06865)	N/A	1380329	2	2	2	2	2	2	2	2	2	2
SAUR1374 (SAR_RS06870)	guaC	1380755	34	34	34	N/A	34	34	34	34	34	34
SAUR1375 (SAR_RS06875)	N/A	1381729	2	2	2	2	2	2	2	2	2	2
SAUR1376 (SAR_RS06880)	ylbC1	1381907	73	73	73	73	73	73	73	73	73	73
SAUR1377 (SAR_RS06885)	lexA	1383150	2	2	2	2	2	2	2	2	2	2
SAUR1378 (SAR_RS06890)	N/A	1383913	45	N/A	45	45	45	45	45	45	45	45
SAUR1379 (SAR_RS06895)	N/A	1384285	2	2	2	2	2	2	N/A	2	2	2
SAUR1380 (SAR_RS06900)	tkt	1384645	125	125	125	125	125	125	N/A	125	125	125
SAUR1381 (SAR_RS06905)	N/A	1386911	1	1	1	1	1	1	1	1	1	1
SAUR1383 (SAR_RS06915)	ccdC	1387332	2	2	N/A	N/A	N/A	N/A	N/A	2	2	2
SAUR1384 (SAR_RS06920)	sbcD	1387923	493	493	N/A	N/A	N/A	N/A	N/A	75	N/A	75
SAUR1388 (SAR_RS06940)	mscL	1392148	3	3	3	3	3	3	3	3	3	3
SAUR1389 (SAR_RS06945)	opuD1	1392714	86	86	N/A	86	86	N/A	N/A	86	86	86
SAUR1390 (SAR_RS06950)	acnA=citB	1395189	147	147	N/A	N/A	N/A	N/A	N/A	147	147	147
SAUR1391 (SAR_RS06955)	yneP	1398073	215	215	3	N/A	N/A	3	N/A	3	3	3
SAUR1392 (SAR_RS06960)	yneR	1398747	3	3	3	3	3	3	3	3	3	3
SAUR1393 (SAR_RS06965)	plsY	1399421	41	N/A	41	41	41	41	41	41	41	41
SAUR1394 (SAR_RS06970)	grlB=parE	1400235	179	179	179	179	179	179	179	179	179	191
SAUR1395 (SAR_RS06975)	grlA=parC	1402226	N/A	203	203	N/A	N/A	203	203	405	203	203
SAUR1396 (SAR_RS06980)	N/A	1404756	10	N/A	10	10	10	10	10	10	10	10
SAUR1397 (SAR_RS06985)	alkT	1404878	75	75	75	75	75	75	75	75	222	75
SAUR1398 (SAR_RS06990)	glcT	1406838	22	N/A	N/A	N/A	N/A	N/A	N/A	22	22	22
SAUR1399 (SAR_RS06995)	N/A	1407664	1	1	1	1	1	1	1	1	1	1
SAUR1400 (SAR_RS07000)	yubA=cozEb	1407824	45	45	45	45	45	45	45	45	45	45
SAUR1401 (SAR_RS07005)	mprF	1409513	146	146	146	N/A	146	146	N/A	897	146	146
SAUR1402 (SAR_RS07010)	msrA1	1412238	22	22	N/A	N/A	N/A	N/A	22	22	22	22
SAUR1403 (SAR_RS07015)	lcpA=tagT	1412884	35	35	35	35	35	35	N/A	35	35	35
SAUR1405 (SAR_RS07025)	dmpI	1413955	3	3	3	3	3	3	3	3	3	3
SAUR1406 (SAR_RS07030)	uvrX	1414288	88	88	N/A	N/A	N/A	N/A	N/A	88	88	88
SAUR1407 (SAR_RS07035)	tyrA	1415687	90	90	90	90	90	90	90	90	90	90
SAUR1408 (SAR_RS07040)	yhfE	1416943	84	84	N/A	N/A	N/A	N/A	84	84	84	84
SAUR1409 (SAR_RS07045)	trpF	1418467	N/A	37	37	37	37	37	N/A	37	N/A	37
SAUR1410 (SAR_RS07050)	trpG	1419870	12	12	12	12	12	12	12	12	12	12
SAUR1411 (SAR_RS07055)	N/A	1420433	8	8	8	8	8	8	8	8	8	8
SAUR1412 (SAR_RS07060)	trpC	1421442	12	12	N/A	N/A	N/A	12	12	72	12	72
SAUR1413 (SAR_RS07065)	N/A	1422221	341	341	15	15	15	15	15	15	15	N/A
SAUR1414 (SAR_RS07070)	trpB	1422849	2	2	2	2	2	2	2	545	2	2
SAUR1415 (SAR_RS07075)	trpA	1424056	31	31	31	31	31	31	31	31	31	31
SAUR1416 (SAR_RS07080)	femA	1425106	93	93	N/A	93	93	N/A	N/A	93	93	93
SAUR1417 (SAR_RS07085)	femB	1426387	484	484	69	69	69	69	69	69	69	69
SAUR1419 (SAR_RS07095)	N/A	1429158	2	2	2	2	2	2	2	2	2	2
SAUR1420 (SAR_RS07100)	nikF	1429739	74	74	N/A	N/A	N/A	N/A	N/A	74	74	74
SAUR1421 (SAR_RS07105)	nikD	1430433	70	70	70	70	70	70	70	70	70	70
SAUR1422 (SAR_RS07110)	nikC	1431193	64	64	64	64	64	64	N/A	64	64	64
SAUR1423 (SAR_RS07115)	nikB	1432016	62	62	62	62	62	62	62	62	62	62
SAUR1425 (SAR_RS07125)	Q5HG37	1433306	39	39	39	N/A	39	39	39	39	39	39
SAUR1426 (SAR_RS07130)	pepF2	1433855	137	137	137	N/A	N/A	137	828	137	137	137
SAUR1427 (SAR_RS07135)	phoU	1435811	20	20	20	20	20	20	20	20	20	20
SAUR1428 (SAR_RS07140)	pstB	1436459	55	55	N/A	N/A	N/A	N/A	N/A	55	55	55
SAUR1429 (SAR_RS07145)	pstA	1437357	73	73	73	73	73	73	73	73	73	73
SAUR1430 (SAR_RS07150)	pstC	1438276	94	94	94	N/A	N/A	94	94	94	94	94
SAUR1431 (SAR_RS07155)	pstS	N/A	482	482	63	63	63	63	N/A	N/A	63	63
SAUR1432 (SAR_RS07160)	cyfB	1440688	79	79	N/A	N/A	N/A	N/A	N/A	79	79	79
SAUR1433 (SAR_RS07165)	ykpA	1441737	80	80	80	80	80	80	N/A	80	80	80
SAUR1434 (SAR_RS07170)	lysC	1444400	103	103	103	103	103	103	103	103	103	103
SAUR1435 (SAR_RS07175)	asd	1445669	85	85	85	85	85	85	85	78	85	78
SAUR1436 (SAR_RS07180)	dapA	1446660	83	83	83	83	83	83	83	83	83	83
SAUR1437 (SAR_RS07185)	dapB	1447544	13	13	N/A	N/A	N/A	N/A	N/A	13	13	13
SAUR1438 (SAR_RS07190)	dapD	1448293	320	320	N/A	N/A	N/A	N/A	N/A	63	63	63
SAUR1439 (SAR_RS07195)	hipO	1449155	105	105	105	105	105	105	105	105	105	105
SAUR1440 (SAR_RS07200)	alr2	1450311	N/A	513	81	81	81	81	81	81	81	81
SAUR1441 (SAR_RS07205)	lysA	1451386	94	94	94	94	94	94	94	90	94	90
SAUR1442 (SAR_RS07210)	N/A	1453496	40	N/A	N/A	N/A	N/A	N/A	40	40	40	40
SAUR1443 (SAR_RS07215)	N/A	1454094	1	1	1	1	1	1	10	1	1	1
SAUR1444 (SAR_RS07220)	N/A	1454329	1	1	1	N/A	N/A	1	1	1	1	1
SAUR1445 (SAR_RS07225)	msaA	1454465	4	4	4	N/A	N/A	4	4	4	4	4
SAUR1446 (SAR_RS07230)	acyP	1454936	1	1	1	1	1	1	1	1	1	1
SAUR1447 (SAR_RS07235)	xpaC	1455230	54	54	54	54	54	N/A	54	54	54	54
SAUR1448 (SAR_RS07240)	terA	1455885	N/A	94	N/A	94	94	94	94	94	94	94
SAUR1449 (SAR_RS07245)	brnQ3	1457095	104	104	N/A	N/A	N/A	N/A	N/A	104	299	104
SAUR1451 (SAR_RS07255)	norQ	1460563	62	62	62	62	62	N/A	62	62	62	62
SAUR1452 (SAR_RS07260)	yozC	1461535	6	N/A	6	N/A	6	6	6	6	39	6
SAUR1453 (SAR_RS07265)	Q5HG08	1461767	471	471	77	77	77	77	77	77	203	77
SAUR1454 (SAR_RS07270)	odhB=sucB	1463155	101	101	101	101	101	101	101	101	101	101
SAUR1455 (SAR_RS07275)	odhA	1464437	198	198	198	N/A	N/A	198	198	198	198	198
SAUR1456 (arlS)	N/A	1467518	N/A	112	112	N/A	112	N/A	112	112	112	112
SAUR1457 (arlR)	N/A	1468870	63	63	63	63	63	63	63	63	63	63
SAUR1459 (SAR_RS07295)	pgpB	1470848	2	2	2	2	2	2	2	2	2	2
SAUR1460 (SAR_RS07300)	murG	1471479	70	70	70	70	70	70	N/A	398	70	70
SAUR1461 (SAR_RS07305)	Q7A0W4	1472561	13	13	13	13	13	13	13	13	13	13
SAUR1462 (SAR_RS07310)	ctpA	1473470	104	104	104	N/A	104	N/A	104	104	104	104
SAUR1463 (SAR_RS07315)	N/A	1474899	17	17	17	17	17	17	17	17	17	17
SAUR1465 (SAR_RS07325)	yozE	1475142	2	2	2	2	2	2	2	2	2	2
SAUR1466 (SAR_RS07330)	crr	1475363	49	49	49	49	49	49	49	49	49	49
SAUR1467 (SAR_RS07335)	msrB	1475875	32	32	32	32	32	32	32	32	32	32
SAUR1468 (SAR_RS07340)	msrA2	1476296	2	2	N/A	2	2	2	2	26	2	2
SAUR1469 (SAR_RS07345)	degV2=fakB2	1476915	78	78	78	78	78	78	78	78	78	78
SAUR1470 (SAR_RS07350)	folA	1477769	2	2	2	2	2	2	2	2	2	2
SAUR1471 (SAR_RS07355)	thyA-chr	1478448	73	73	73	73	73	73	N/A	73	73	73
SAUR1472 (SAR_RS07360)	cyfC4	1479828	50	N/A	50	50	50	50	50	50	50	50
SAUR1473 (SAR_RS07365)	cyfC3	1480281	96	96	96	96	96	96	N/A	96	96	96

Locus (cg MLST)	CC692				CC522					ST1347	Chinese CC133 MRSA isolates		CC5	ST71	CC182	CC707	CC1956
	CP042650.1, X22	UHAK01000002.1, NCTC9546	PubMLST ID 39948, 973N0056	CP129360, CKMM-401M	Goat isolate 17CS1042	PubMLST ID 24977	PubMLST ID 24358	PubMLST ID 11704	SAMEA1904171	PubMLST ID 39945, 61290	JAOAKU01, GDQ20D75A	JAFEOM01, GDQ20D75A	CP001781.1, ED98	SAMN03247575, MOK-099	CP070991, CBS2016-05	CP029751, SMITH	CP084892, WT19
SAUR1135 (SAR_RS05675)	129	129	129	129	109	109	227	109	109	129	N/A	N/A	8	8	77	172	N/A
SAUR1136 (SAR_RS05680)	N/A	1025	513	513	357	357	527	202	357	513	N/A	N/A	15	112	154	362	N/A
SAUR1137 (SAR_RS05685)	43	397	43	43	103	236	103	103	103	370	79	79	364	21	129	168	N/A
SAUR1138 (SAR_RS05690)	1	1	1	1	3	3	3	3	3	1	2	2	1	5	10	10	3
SAUR1139 (SAR_RS05695)	7	7	7	7	20	20	20	20	20	7	47	47	3	3	2	2	59
SAUR1140 (SAR_RS05700)	337	337	337	337	158	158	158	158	158	337	107	107	11	34	197	257	N/A
SAUR1141 (SAR_RS05705)	N/A	254	254	254	324	324	324	209	324	254	484	484	8	45	136	327	N/A
SAUR1142 (SAR_RS05710)	6	6	6	6	14	14	14	14	14	6	14	14	6	8	14	22	14
SAUR1143 (SAR_RS05715)	9	9	9	9	42	42	42	42	42	N/A	8	8	9	N/A	42	13	3
SAUR1144 (SAR_RS05720)	9	9	9	9	261	261	364	157	261	9	102	102	9	35	194	265	N/A
SAUR1145 (SAR_RS05725)	67	67	67	67	88	88	88	57	88	67	47	47	7	7	2	2	2
SAUR1146 (SAR_RS05730)	162	162	162	162	N/A	312	127	127	127	162	N/A	N/A	7	21	164	214	N/A
SAUR1147 (SAR_RS05735)	74	74	74	74	96	58	58	58	96	74	50	50	6	17	58	58	43
SAUR1148 (SAR_RS05740)	N/A	83	83	83	69	69	69	69	69	83	25	25	5	21	25	25	70
SAUR1149 (SAR_RS05745)	122	122	122	122	N/A	140	55	55	55	122	21	21	6	14	74	74	36
SAUR1150 (SAR_RS05750)	6	6	6	6	49	49	49	49	49	N/A	17	17	144	N/A	62	30	30
SAUR1151 (SAR_RS05755)	9	9	9	9	34	34	34	34	34	N/A	6	6	14	20	N/A	N/A	N/A
SAUR1152 (SAR_RS05760)	105	105	105	105	35	35	35	35	35	105	N/A	N/A	84	12	13	13	21
SAUR1155 (SAR_RS05775)	63	63	63	63	51	51	51	51	51	63	15	15	8	20	64	38	N/A
SAUR1156 (SAR_RS05780)	60	60	60	60	47	47	108	47	47	N/A	19	19	4	N/A	62	19	2
SAUR1157 (SAR_RS05785)	44	44	44	44	36	36	36	36	36	N/A	13	13	14	16	46	N/A	N/A
SAUR1158 (SAR_RS05790)	44	44	44	44	N/A	35	35	35	35	N/A	18	18	19	21	18	50	18
SAUR1161 (SAR_RS05805)	294	294	294	294	167	100	100	100	167	N/A	N/A	N/A	10	11	2	2	N/A
SAUR1162 (SAR_RS05810)	27	27	27	27	2	2	2	2	2	N/A	18	18	5	2	2	9	24
SAUR1163 (SAR_RS05815)	19	19	19	19	19	19	19	19	19	N/A	12	12	11	12	19	32	19
SAUR1165 (SAR_RS05825)	126	126	308	126	111	111	111	111	111	N/A	96	96	49	52	127	146	69
SAUR1166 (SAR_RS05830)	N/A	N/A	49	N/A	N/A	94	94	94	94	N/A	79	79	263	N/A	113	145	95
SAUR1167 (SAR_RS05835)	N/A	177	177	N/A	106	106	106	106	106	N/A	N/A	N/A	53	54	129	149	148
SAUR1168 (SAR_RS05840)	142	142	142	142	122	122	122	122	122	142	47	47	9	10	152	188	47
SAUR1169 (SAR_RS05845)	17	17	17	17	151	151	151	93	151	17	75	75	11	25	115	2	2
SAUR1170 (SAR_RS05850)	203	203	203	203	261	261	261	165	261	203	118	118	13	38	330	263	167
SAUR1171 (SAR_RS05855)	6	6	6	6	18	18	18	18	18	N/A	30	30	6	16	N/A	18	2
SAUR1172 (SAR_RS05860)	134	134	134	134	111	111	111	111	111	134	79	79	378	27	2	171	2
SAUR1173 (SAR_RS05865)	33	33	33	33	N/A	N/A	N/A	N/A	N/A	N/A	N/A	N/A	11	12	19	N/A	19
SAUR1175 (psmβ1)	4	4	4	4	51	1	1	1	1	4	1	1	4	4	1	1	1
SAUR2947 (psmβ2)	1	N/A	1	1	N/A	N/A	N/A	N/A	N/A	N/A	1	1	1	1	20	2	20
SAUR1176 (SAR_RS05880)	108	108	108	108	91	91	91	91	91	108	70	70	6	7	N/A	2	N/A
SAUR1177 (SAR_RS05885)	2	2	2	2	N/A	101	43	43	43	2	2	2	2	2	20	2	20
SAUR1178 (SAR_RS05890)	N/A	235	235	N/A	438	188	188	188	188	235	142	142	7	8	740	312	N/A
SAUR1179 (SAR_RS05895)	8	1	1	1	9	1	1	1	1	1	1	1	1	1	1	1	110
SAUR1180 (SAR_RS05900)	132	132	132	132	105	105	105	105	105	469	37	37	8	7	105	37	105
SAUR1181 (SAR_RS05905)	1	1	1	1	1	1	1	1	1	N/A	1	1	1	1	46	1	1
SAUR1182 (SAR_RS05910)	295	295	1001	295	238	238	238	238	238	295	176	176	93	N/A	901	390	241
SAUR1183 (SAR_RS05915)	127	127	127	127	N/A	109	109	109	109	428	78	78	8	31	110	171	110
SAUR1184 (SAR_RS05920)	169	169	169	169	138	138	138	138	138	169	108	108	9	11	171	228	139
SAUR1185 (SAR_RS05925)	N/A	191	191	N/A	156	156	156	156	251	191	2	2	13	42	95	255	N/A
SAUR1186 (SAR_RS05930)	147	147	147	147	267	284	117	117	117	147	64	64	9	28	117	199	33
SAUR1187 (SAR_RS05935)	90	90	90	90	2	2	2	2	2	90	2	2	10	18	2	2	73
SAUR1188 (SAR_RS05940)	120	120	120	120	103	103	235	103	103	120	N/A	N/A	10	57	359	162	N/A
SAUR1189 (SAR_RS05945)	11	11	284	11	19	19	19	19	112	11	2	2	227	8	19	113	67
SAUR1190 (SAR_RS05950)	2	2	2	2	14	14	14	14	14	2	2	2	5	11	59	26	2
SAUR1191 (SAR_RS05955)	19	19	19	19	13	13	13	13	13	19	29	29	3	3	13	37	N/A
SAUR1192 (SAR_RS05960)	8	8	8	8	30	30	30	30	131	N/A	69	69	7	29	30	N/A	N/A
SAUR1194 (SAR_RS05970)	N/A	952	286	N/A	368	539	368	219	368	286	N/A	N/A	12	50	306	371	N/A
SAUR1196 (SAR_RS05980)	10	10	10	10	27	27	N/A	110	27	10	82	82	10	23	27	2	27
SAUR1197 (SAR_RS05985)	5	5	5	5	1	1	1	1	1	5	1	1	5	4	1	1	1
SAUR1198 (SAR_RS05990)	5	108	5	5	83	83	83	83	83	5	25	25	6	5	83	139	84
SAUR1199 (SAR_RS05995)	12	12	12	12	N/A	13	94	13	13	12	42	42	8	9	58	13	18
SAUR1200 (SAR_RS06000)	N/A	22	22	N/A	N/A	134	134	134	134	523	109	109	11	30	471	217	216
SAUR1201 (SAR_RS06005)	19	19	19	19	48	48	48	96	48	369	48	48	9	51	48	142	48
SAUR1202 (SAR_RS06010)	156	549	156	156	129	129	129	129	129	156	96	96	9	31	157	196	130
SAUR1203 (SAR_RS06015)	218	434	218	218	N/A	62	62	62	62	N/A	117	117	336	29	8	8	98
SAUR1204 (SAR_RS06020)	289	289	289	289	232	232	232	232	392	1100	174	174	11	39	294	396	N/A
SAUR1205 (SAR_RS06025)	N/A	19	19	N/A	174	80	80	80	80	301	66	66	6	19	91	122	29
SAUR1206 (SAR_RS06030)	N/A	7	7	N/A	64	64	64	64	64	7	43	43	7	7	83	99	13
SAUR1207 (SAR_RS06035)	5	5	5	5	17	17	17	17	17	5	1	1	5	9	40	17	17
SAUR1208 (SAR_RS06040)	N/A	70	70	N/A	15	15	15	15	15	70	49	49	7	8	71	82	15
SAUR1209 (SAR_RS06045)	201	201	201	201	158	158	158	158	158	201	129	129	418	48	204	261	159
SAUR1210 (SAR_RS06050)	N/A	5	5	5	6	1	1	1	1	5	39	39	4	5	1	1	1
gmk	N/A	1	1	1	6	2	2	2	2	N/A	2	2	4	1	2	2	2
SAUR1211 (SAR_RS06055)	3	3	3	3	1	1	1	1	1	3	1	1	2	2	21	1	1
SAUR1212 (SAR_RS06060)	128	128	128	128	97	97	97	97	97	128	78	78	10	26	131	97	98
SAUR1213 (SAR_RS06065)	808	402	402	808	287	287	287	180	287	402	126	126	11	48	224	289	N/A
SAUR1214 (SAR_RS06070)	3	3	3	3	N/A	N/A	N/A	N/A	N/A	N/A	53	53	3	6	83	N/A	N/A
SAUR1215 (SAR_RS06075)	N/A	N/A	N/A	N/A	2	42	2	2	2	N/A	2	2	1	1	2	2	17
SAUR1216 (SAR_RS06080)	14	14	14	14	55	55	55	55	55	N/A	23	23	8	15	187	2	2
SAUR1217 (SAR_RS06085)	10	10	10	10	96	96	96	96	96	10	2	2	334	31	127	153	N/A
SAUR1218 (SAR_RS06090)	N/A	181	181	N/A	144	144	310	144	144	578	111	111	10	36	182	230	N/A
SAUR1219 (SAR_RS06095)	N/A	137	137	N/A	117	117	117	117	117	137	N/A	N/A	9	98	139	139	N/A
SAUR1220 (SAR_RS06100)	8	8	8	8	67	67	67	67	149	362	67	67	6	19	108	3	44
SAUR1221 (SAR_RS06105)	252	252	252	252	199	486	199	199	199	N/A	140	140	12	7	253	307	200
SAUR1222 (SAR_RS06110)	104	104	104	104	50	50	50	50	50	104	69	69	7	N/A	105	50	88
SAUR1223 (SAR_RS06115)	N/A	8	8	N/A	N/A	70	70	70	70	8	2	2	7	8	91	2	72
SAUR1224 (SAR_RS06120)	1	1	1	1	57	57	57	57	57	1	43	43	8	1	3	104	59
SAUR1225 (SAR_RS06125)	1	1	1	1	1	1	1	1	1	1	1	1	1	1	1	1	1
SAUR1226 (SAR_RS06130)	1	1	1	1	1	1	1	1	1	1	1	1	4	1	30	1	1
SAUR1227 (SAR_RS06135)	22	22	22	22	157	157	364	157	273	654	131	131	12	36	214	278	N/A
SAUR1228 (SAR_RS06140)	N/A	252	252	N/A	313	487	313	189	313	845	246	246	11	47	753	317	190
SAUR1229 (SAR_RS06145)	5	5	5	5	54	54	54	54	54	5	44	44	5	5	78	4	4
SAUR1230 (SAR_RS06150)	30	30	30	30	93	93	93	93	93	30	62	62	13	30	120	62	62
SAUR1231 (SAR_RS06155)	189	189	189	189	148	148	148	148	148	N/A	117	117	9	39	42	228	42
SAUR1232 (SAR_RS06160)	7	7	7	7	76	76	76	76	76	7	22	22	7	7	89	76	76
SAUR1233 (SAR_RS06165)	5	5	5	5	1	1	1	1	1	N/A	N/A	N/A	2	5	1	1	1
SAUR1234 (SAR_RS06170)	1	1	1	1	1	1	1	1	1	1	1	1	1	1	1	1	1
SAUR1235 (SAR_RS06175)	6	6	6	6	2	2	2	2	2	6	46	46	5	6	2	2	2
SAUR1236 (SAR_RS06180)	N/A	1282	1217	N/A	N/A	725	283	283	283	1216	N/A	N/A	17	46	370	481	284
SAUR1237 (SAR_RS06185)	53	53	53	53	139	139	139	139	139	53	103	103	9	34	442	205	N/A
SAUR1238 (SAR_RS06190)	4	4	112	4	5	5	5	5	5	N/A	7	7	3	4	5	10	3
SAUR1239 (SAR_RS06195)	261	261	261	261	112	112	112	112	112	261	N/A	N/A	11	39	142	112	N/A
SAUR1240 (SAR_RS06200)	1	1	1	1	1	1	1	31	1	1	1	1	1	12	38	1	1
SAUR1241 (SAR_RS06205)	145	145	145	145	19	19	19	19	90	204	48	48	3	146	19	92	48
SAUR1242 (SAR_RS06210)	83	83	83	83	71	71	71	71	71	83	26	26	7	27	85	71	2
SAUR1243 (SAR_RS06215)	1	1	1	1	34	34	34	34	34	131	8	8	3	2	44	1	1
SAUR1245 (SAR_RS06225)	N/A	512	512	N/A	362	214	214	214	362	512	156	156	13	44	270	366	N/A
SAUR1246 (SAR_RS06230)	99	99	372	99	2	2	2	77	128	99	19	19	4	16	105	130	2
SAUR1247 (SAR_RS06235)	N/A	N/A	N/A	N/A	96	96	96	96	96	N/A	67	67	30	44	2	153	2
SAUR1248 (SAR_RS06240)	143	143	143	143	111	275	111	111	111	143	N/A	N/A	12	25	13	181	2
SAUR1249 (SAR_RS06245)	18	18	18	18	75	75	75	101	75	391	36	36	10	34	35	75	N/A
SAUR1253 (SAR_RS06265)	197	197	717	197	N/A	2	2	2	2	716	N/A	N/A	11	171	199	2	2
SAUR1254 (SAR_RS06270)	145	145	145	145	2	2	2	2	2	145	2	2	6	32	146	193	N/A
SAUR1255 (SAR_RS06275)	N/A	9	9	9	110	110	110	110	110	9	2	2	8	10	2	168	2
SAUR1256 (SAR_RS06280)	34	34	34	34	2	2	2	77	2	34	2	2	8	9	2	15	2
SAUR1257 (SAR_RS06285)	182	182	182	182	2	2	2	2	2	683	N/A	N/A	7	22	183	252	2
SAUR1258 (SAR_RS06290)	100	100	100	100	56	56	56	56	56	100	64	64	9	11	56	142	56
SAUR1259 (SAR_RS06295)	2	2	2	2	2	2	2	2	2	N/A	2	2	N/A	8	2	2	2
SAUR1260 (SAR_RS06300)	N/A	88	88	88	12	12	12	12	12	N/A	1	1	12	16	90	1	N/A
SAUR1261 (SAR_RS06305)	2	2	2	2	2	2	2	2	2	2	2	2	2	2	2	2	2
SAUR1262 (SAR_RS06310)	N/A	1	334	N/A	N/A	51	139	51	139	1	1	1	5	21	51	1	2
SAUR1263 (SAR_RS06315)	11	11	11	11	2	2	121	2	2	11	40	40	11	12	2	79	2
SAUR1264 (SAR_RS06320)	71	71	71	71	2	2	2	2	2	71	16	16	3	6	2	16	2
SAUR1265 (SAR_RS06325)	7	7	7	7	54	54	54	54	54	7	141	141	6	7	13	85	N/A
SAUR1266 (SAR_RS06330)	20	20	20	20	47	149	47	47	47	20	27	27	7	25	123	90	N/A
SAUR1267 (SAR_RS06335)	N/A	168	168	N/A	205	141	141	141	205	168	N/A	N/A	10	39	170	206	N/A
SAUR1268 (SAR_RS06340)	347	734	347	347	N/A	363	363	157	249	347	115	115	10	42	199	253	N/A
SAUR1269 (SAR_RS06345)	1300	923	1301	1300	N/A	273	273	273	273	923	N/A	N/A	13	N/A	360	475	276
SAUR1270 (SAR_RS06350)	1	1	1	1	1	1	1	1	1	1	42	42	4	4	62	9	N/A
SAUR1271 (SAR_RS06355)	20	20	20	20	114	114	114	114	114	34	86	86	9	N/A	444	184	115
SAUR1272 (SAR_RS06360)	32	32	32	32	20	20	20	20	20	32	2	2	6	2	20	20	27
SAUR1273 (SAR_RS06365)	50	50	136	50	2	2	2	2	2	50	31	31	3	3	3	3	2
SAUR1274 (SAR_RS06370)	81	81	81	81	N/A	185	185	185	185	81	140	140	665	40	238	325	492
SAUR1275 (SAR_RS06375)	1	1	1	1	2	2	2	2	2	1	2	2	1	1	2	50	2
SAUR1276 (SAR_RS06380)	121	388	121	121	36	36	36	36	36	121	25	25	112	114	36	57	37
SAUR1277 (SAR_RS06385)	22	22	22	22	N/A	99	228	99	99	22	119	119	7	34	37	37	100
SAUR1278 (SAR_RS06390)	2	2	2	2	1	1	1	1	1	2	1	1	1	1	1	1	1
SAUR1279 (SAR_RS06395)	228	228	228	N/A	177	177	177	177	177	228	119	119	14	N/A	231	109	178
SAUR1280 (SAR_RS06400)	N/A	51	51	N/A	248	248	344	151	248	623	120	120	6	34	191	250	120
SAUR1281 (SAR_RS06405)	233	N/A	840	233	N/A	183	451	183	183	233	138	138	10	13	237	314	N/A
SAUR1282 (SAR_RS06410)	99	99	99	99	85	85	85	85	85	99	26	26	8	68	287	132	26
SAUR1283 (SAR_RS06415)	N/A	151	151	N/A	N/A	45	45	45	45	151	89	89	9	30	154	45	59
SAUR1284 (SAR_RS06420)	201	201	201	201	N/A	348	148	148	148	201	115	115	11	123	90	253	N/A
SAUR1285 (SAR_RS06425)	86	86	86	86	2	2	157	2	2	86	57	57	8	22	87	116	2
SAUR1286 (SAR_RS06430)	9	9	9	9	43	43	43	43	43	9	53	53	7	9	81	109	21
SAUR1287 (SAR_RS06435)	70	70	70	70	17	17	17	17	17	70	2	2	1	1	2	17	17
SAUR1288 (SAR_RS06440)	5	5	5	5	49	49	49	49	49	5	37	37	5	16	2	81	N/A
SAUR1289 (SAR_RS06445)	458	143	143	458	240	123	123	123	123	143	92	92	11	44	145	185	92
SAUR1290 (SAR_RS06450)	35	35	35	35	35	35	35	35	35	35	62	62	6	21	104	132	89
SAUR1291 (SAR_RS06455)	N/A	131	131	N/A	245	18	18	18	18	131	18	18	9	117	132	173	30
SAUR1292 (SAR_RS06460)	1	1	1	1	1	1	1	1	1	1	1	1	1	N/A	1	1	1
SAUR1293 (SAR_RS06465)	84	84	84	84	23	23	23	23	23	84	59	59	7	9	85	110	70
SAUR1294 (SAR_RS06470)	196	196	196	196	156	156	156	156	156	615	121	121	8	N/A	197	255	N/A
SAUR1295 (SAR_RS06475)	160	160	160	160	109	109	109	73	109	160	47	47	7	12	2	110	2
SAUR1296 (SAR_RS06480)	6	6	6	6	2	2	2	2	2	129	2	2	3	11	19	18	2
SAUR1297 (SAR_RS06485)	N/A	169	169	N/A	131	324	131	131	131	169	2	2	8	N/A	37	220	N/A
SAUR1298 (SAR_RS06490)	6	6	6	6	42	42	42	42	42	6	1	1	5	1	1	1	1
SAUR1299 (SAR_RS06495)	64	64	64	64	2	2	134	2	2	64	2	2	7	10	2	2	2
SAUR1300 (SAR_RS06500)	N/A	N/A	239	239	192	192	192	192	192	N/A	138	138	15	24	241	321	138
SAUR1302 (SAR_RS06510)	117	117	117	117	57	57	57	57	57	117	2	2	7	8	71	69	2
SAUR1303 (SAR_RS06515)	7	7	388	7	74	74	74	74	74	7	58	58	7	7	22	28	9
glpF	1	1	108	1	45	45	45	45	45	N/A	46	46	1	1	6	6	6
SAUR1304 (SAR_RS06520)	161	161	161	161	204	127	127	127	204	161	N/A	N/A	13	98	166	206	2
SAUR1305 (SAR_RS06525)	N/A	375	375	N/A	122	122	122	122	122	270	34	34	8	10	471	198	34
SAUR1306 (SAR_RS06530)	205	N/A	205	205	68	68	68	68	68	402	28	28	9	10	114	155	N/A
SAUR1308 (SAR_RS06540)	3	3	3	3	2	2	2	2	2	3	N/A	N/A	3	3	2	2	2
SAUR1309 (SAR_RS06545)	82	82	82	82	70	70	70	70	70	82	14	14	6	21	84	51	71
SAUR1310 (SAR_RS06550)	N/A	157	157	N/A	125	125	125	125	125	157	N/A	N/A	10	14	160	211	N/A
SAUR1311 (SAR_RS06555)	147	343	343	147	243	119	119	119	119	147	93	93	9	9	413	N/A	N/A
SAUR1312 (SAR_RS06560)	N/A	N/A	N/A	5	1	1	1	1	1	N/A	1	1	1	N/A	1	1	1
SAUR1313 (SAR_RS06565)	110	110	110	N/A	143	143	143	85	143	110	27	27	290	374	111	146	86
SAUR1352 (SAR_RS06760)	150	150	150	150	114	306	114	114	114	150	84	84	9	125	153	203	N/A
SAUR1354 (SAR_RS06770)	N/A	444	444	N/A	115	62	115	62	115	444	44	44	247	249	N/A	118	63
SAUR2949 (MW1206)	N/A	N/A	N/A	N/A	N/A	N/A	N/A	N/A	N/A	N/A	17	17	1	1	N/A	N/A	N/A
SAUR2950 (MW1207)	N/A	N/A	N/A	N/A	N/A	N/A	N/A	N/A	N/A	N/A	45	45	4	1	N/A	N/A	144
SAUR2951 (desK)	N/A	N/A	N/A	N/A	N/A	N/A	N/A	N/A	N/A	N/A	56	56	4	1	N/A	N/A	75
SAUR2952 (desR)	N/A	N/A	N/A	N/A	N/A	N/A	N/A	N/A	N/A	N/A	3	3	1	1	N/A	N/A	59
SAUR1359 (SAR_RS06795)	3	3	3	3	7	7	7	7	7	N/A	26	26	20	6	40	7	13
SAUR1360 (SAR_RS06800)	3	3	3	3	56	56	56	56	56	3	N/A	N/A	6	6	37	56	37
SAUR1361 (SAR_RS06805)	157	157	157	157	70	178	70	70	70	157	56	56	11	8	23	70	71
SAUR1362 (SAR_RS06810)	23	23	23	23	2	2	2	2	2	23	22	22	8	8	204	22	N/A
SAUR1363 (SAR_RS06815)	N/A	207	207	N/A	357	159	159	159	159	N/A	120	120	10	177	38	256	160
SAUR1364 (SAR_RS06820)	10	10	10	10	242	119	119	119	119	10	85	85	11	12	149	183	N/A
SAUR1365 (SAR_RS06825)	N/A	262	262	262	114	114	114	114	114	262	38	38	15	9	148	188	115
SAUR1366 (SAR_RS06830)	106	106	106	106	89	202	89	89	89	106	N/A	N/A	25	39	109	139	43
SAUR1367 (SAR_RS06835)	104	104	104	104	N/A	208	83	83	83	104	61	61	7	41	14	131	45
SAUR1368 (SAR_RS06840)	17	17	17	17	1	1	1	1	1	17	1	1	128	7	1	1	1
SAUR1369 (SAR_RS06845)	1	1	1	1	1	1	1	1	1	N/A	2	2	4	1	1	1	1
SAUR1370 (SAR_RS06850)	260	260	260	260	266	283	266	101	175	260	74	74	9	9	436	176	31
SAUR1371 (SAR_RS06855)	167	167	167	167	137	137	137	137	137	167	N/A	N/A	485	10	170	215	137
SAUR1372 (SAR_RS06860)	2	2	2	2	9	9	9	9	9	2	2	2	2	2	2	2	2
SAUR1373 (SAR_RS06865)	32	32	32	32	2	2	2	30	2	32	2	2	2	2	2	2	2
SAUR1374 (SAR_RS06870)	137	137	137	137	N/A	34	34	34	34	434	34	34	10	32	138	183	114
SAUR1375 (SAR_RS06875)	3	3	3	3	2	2	2	2	2	3	2	2	2	2	2	2	2
SAUR1376 (SAR_RS06880)	127	127	127	127	3	3	3	N/A	3	127	73	73	319	19	129	162	58
SAUR1377 (SAR_RS06885)	8	8	8	8	3	3	3	3	3	8	N/A	N/A	7	14	3	2	2
SAUR1378 (SAR_RS06890)	76	76	76	76	3	3	3	3	3	76	45	45	3	5	3	1	1
SAUR1379 (SAR_RS06895)	2	2	2	2	31	31	31	31	31	105	2	2	2	2	2	2	2
SAUR1380 (SAR_RS06900)	N/A	826	215	N/A	169	169	169	169	169	215	125	125	12	33	218	2	N/A
SAUR1381 (SAR_RS06905)	1	1	1	1	1	1	1	1	1	1	1	1	1	1	66	1	1
SAUR1383 (SAR_RS06915)	5	5	5	5	2	2	2	2	2	5	2	2	48	5	2	2	2
SAUR1384 (SAR_RS06920)	17	17	17	17	98	98	243	98	98	17	75	75	6	16	119	175	99
SAUR1388 (SAR_RS06940)	3	3	3	3	34	34	34	34	34	3	3	3	6	3	2	92	2
SAUR1389 (SAR_RS06945)	149	510	149	149	115	115	115	115	115	149	N/A	N/A	51	20	151	196	N/A
SAUR1390 (SAR_RS06950)	488	968	488	488	204	204	204	204	354	488	N/A	N/A	16	51	835	358	205
SAUR1391 (SAR_RS06955)	68	68	68	68	3	122	3	3	3	68	3	3	5	5	2	3	3
SAUR1392 (SAR_RS06960)	54	54	54	54	3	3	3	3	3	54	3	3	6	6	2	3	3
SAUR1393 (SAR_RS06965)	65	65	65	65	50	50	50	50	50	65	41	41	6	15	2	3	3
SAUR1394 (SAR_RS06970)	579	579	579	579	459	330	330	221	330	579	179	179	17	N/A	706	331	223
SAUR1395 (SAR_RS06975)	N/A	320	320	N/A	N/A	265	265	265	419	1000	1164	1164	69	73	324	423	266
SAUR1396 (SAR_RS06980)	7	7	7	7	2	2	2	2	2	N/A	10	10	1	N/A	3	2	1
SAUR1397 (SAR_RS06985)	126	126	126	126	26	26	26	26	26	N/A	75	75	9	33	26	26	N/A
SAUR1398 (SAR_RS06990)	N/A	7	7	N/A	46	46	158	46	46	271	22	22	6	26	88	46	67
SAUR1399 (SAR_RS06995)	1	1	1	1	1	1	1	1	1	N/A	1	1	1	1	1	1	1
SAUR1400 (SAR_RS07000)	8	8	8	8	64	64	64	64	64	8	45	45	8	22	76	17	17
SAUR1401 (SAR_RS07005)	913	N/A	223	913	195	195	195	195	195	223	N/A	N/A	7	17	796	306	196
SAUR1402 (SAR_RS07010)	10	10	10	10	22	22	22	63	22	10	22	22	8	10	82	103	36
SAUR1403 (SAR_RS07015)	21	21	21	21	92	92	92	92	92	382	35	35	7	31	118	35	15
SAUR1405 (SAR_RS07025)	3	3	3	3	24	24	24	24	24	3	3	3	3	3	3	3	3
SAUR1406 (SAR_RS07030)	312	312	312	312	104	104	104	104	104	427	88	88	6	7	104	N/A	N/A
SAUR1407 (SAR_RS07035)	642	188	643	642	241	241	363	141	241	188	90	90	41	N/A	189	244	68
SAUR1408 (SAR_RS07040)	130	130	455	130	236	111	111	111	111	130	84	84	6	6	111	107	N/A
SAUR1409 (SAR_RS07045)	168	168	168	168	347	129	129	129	129	622	37	37	40	N/A	549	244	15
SAUR1410 (SAR_RS07050)	N/A	N/A	N/A	N/A	N/A	N/A	N/A	N/A	N/A	N/A	12	12	60	N/A	N/A	N/A	N/A
SAUR1411 (SAR_RS07055)	142	142	142	142	435	435	435	435	435	N/A	8	8	39	39	321	410	58
SAUR1412 (SAR_RS07060)	N/A	125	125	125	106	106	106	106	106	N/A	12	12	24	N/A	126	169	12
SAUR1413 (SAR_RS07065)	246	246	246	246	15	15	15	15	15	N/A	N/A	N/A	23	23	93	N/A	34
SAUR1414 (SAR_RS07070)	N/A	185	185	N/A	147	147	147	147	147	185	2	2	5	33	543	234	45
SAUR1415 (SAR_RS07075)	52	52	52	52	44	44	44	44	44	N/A	N/A	N/A	118	118	53	60	N/A
SAUR1416 (SAR_RS07080)	155	155	155	155	13	13	13	128	13	155	93	93	7	19	156	187	129
SAUR1417 (SAR_RS07085)	143	143	143	143	176	115	115	115	176	143	N/A	N/A	6	18	116	178	N/A
SAUR1419 (SAR_RS07095)	N/A	N/A	N/A	N/A	N/A	N/A	N/A	N/A	N/A	N/A	2	2	N/A	39	N/A	N/A	N/A
SAUR1420 (SAR_RS07100)	N/A	4	4	N/A	93	93	93	93	93	N/A	74	74	8	8	N/A	15	N/A
SAUR1421 (SAR_RS07105)	127	127	127	127	162	37	37	37	162	N/A	70	70	16	16	128	165	N/A
SAUR1422 (SAR_RS07110)	33	33	33	33	93	93	93	93	93	N/A	64	64	351	44	120	160	N/A
SAUR1423 (SAR_RS07115)	226	226	226	226	45	45	45	45	45	N/A	62	62	23	23	N/A	162	N/A
SAUR1425 (SAR_RS07125)	56	56	56	56	44	44	44	44	44	56	39	39	2	12	19	67	45
SAUR1426 (SAR_RS07130)	8	8	8	8	N/A	182	182	182	182	8	137	137	73	8	228	299	268
SAUR1427 (SAR_RS07135)	N/A	7	7	N/A	72	72	72	72	72	7	20	20	6	18	82	105	20
SAUR1428 (SAR_RS07140)	5	5	5	5	2	2	2	2	2	326	55	55	35	5	93	132	79
SAUR1429 (SAR_RS07145)	11	11	11	11	104	104	104	104	104	11	73	73	10	10	15	174	N/A
SAUR1430 (SAR_RS07150)	479	12	12	479	116	116	116	116	116	12	N/A	N/A	11	99	439	196	23
SAUR1431 (SAR_RS07155)	9	496	9	9	251	94	251	94	180	N/A	63	63	8	8	144	181	95
SAUR1432 (SAR_RS07160)	11	11	11	11	99	241	99	99	99	11	79	79	342	N/A	131	73	64
SAUR1433 (SAR_RS07165)	N/A	370	370	N/A	109	109	109	109	109	571	N/A	N/A	9	N/A	505	30	N/A
SAUR1434 (SAR_RS07170)	N/A	99	99	N/A	N/A	141	141	141	141	99	103	103	8	29	184	184	142
SAUR1435 (SAR_RS07175)	89	89	89	89	109	109	109	109	109	89	85	85	8	10	135	171	110
SAUR1436 (SAR_RS07180)	16	16	16	16	222	100	100	100	100	16	83	83	6	10	N/A	164	101
SAUR1437 (SAR_RS07185)	27	27	27	27	N/A	71	71	71	71	27	13	13	47	27	13	106	2
SAUR1438 (SAR_RS07190)	7	7	7	7	124	77	77	77	124	7	63	63	5	12	101	126	28
SAUR1439 (SAR_RS07195)	N/A	115	115	N/A	138	138	138	138	138	115	105	105	19	11	N/A	234	139
SAUR1440 (SAR_RS07200)	279	279	279	279	N/A	108	108	108	108	N/A	N/A	N/A	7	16	144	193	120
SAUR1441 (SAR_RS07205)	N/A	20	20	N/A	127	127	127	127	127	N/A	94	94	432	5	161	210	128
SAUR1442 (SAR_RS07210)	16	16	16	16	43	43	43	43	43	N/A	40	40	12	12	26	77	28
SAUR1443 (SAR_RS07215)	1	1	1	1	1	45	1	1	1	1	1	1	1	1	1	1	1
SAUR1444 (SAR_RS07220)	1	1	1	1	1	1	N/A	1	1	N/A	1	1	29	1	1	1	N/A
SAUR1445 (SAR_RS07225)	N/A	4	4	N/A	31	31	31	31	31	4	4	4	4	1	4	4	N/A
SAUR1446 (SAR_RS07230)	4	4	4	4	25	25	25	25	25	4	1	1	4	4	23	9	1
SAUR1447 (SAR_RS07235)	10	10	10	10	167	71	71	71	71	10	54	54	10	14	51	120	N/A
SAUR1448 (SAR_RS07240)	N/A	77	77	N/A	133	133	133	133	133	77	94	94	11	34	471	N/A	N/A
SAUR1449 (SAR_RS07245)	N/A	610	10	N/A	138	138	322	138	138	10	104	104	10	13	170	222	N/A
SAUR1451 (SAR_RS07255)	7	7	7	7	81	81	81	81	81	7	62	62	8	26	98	126	2
SAUR1452 (SAR_RS07260)	3	3	3	3	6	6	6	6	6	3	39	39	3	3	6	6	2
SAUR1453 (SAR_RS07265)	9	9	458	9	N/A	339	339	339	339	9	203	203	8	8	341	348	N/A
SAUR1454 (SAR_RS07270)	11	11	536	11	137	137	137	137	137	11	101	101	10	10	137	N/A	138
SAUR1455 (SAR_RS07275)	N/A	1281	1211	N/A	N/A	256	256	256	485	N/A	N/A	N/A	12	49	609	491	N/A
SAUR1456 (arlS)	N/A	184	184	N/A	147	147	147	147	147	620	112	112	8	25	562	241	148
SAUR1457 (arlR)	7	7	7	7	80	80	80	80	80	N/A	63	63	6	6	100	135	81
SAUR1459 (SAR_RS07295)	94	94	94	94	179	81	81	81	81	94	2	2	7	9	96	129	2
SAUR1460 (SAR_RS07300)	117	117	117	117	93	93	93	93	93	117	70	70	7	14	118	38	N/A
SAUR1461 (SAR_RS07305)	75	75	75	75	28	28	28	28	28	N/A	13	13	6	8	76	13	13
SAUR1462 (SAR_RS07310)	155	155	155	155	130	130	130	130	130	155	104	104	9	12	172	231	N/A
SAUR1463 (SAR_RS07315)	2	2	2	2	2	2	2	2	2	N/A	17	17	2	2	7	34	2
SAUR1465 (SAR_RS07325)	5	5	5	5	2	2	2	2	2	5	2	2	5	5	7	2	29
SAUR1466 (SAR_RS07330)	9	9	9	9	2	2	2	2	2	9	49	49	6	7	18	2	74
SAUR1467 (SAR_RS07335)	10	10	10	10	32	32	32	32	32	N/A	32	32	7	8	22	22	18
SAUR1468 (SAR_RS07340)	13	13	13	13	61	61	61	61	61	N/A	2	2	9	7	2	2	2
SAUR1469 (SAR_RS07345)	65	65	65	N/A	78	78	78	78	78	65	78	78	7	8	115	25	100
SAUR1470 (SAR_RS07350)	60	60	60	60	93	93	93	93	93	60	2	2	7	9	102	2	34
SAUR1471 (SAR_RS07355)	114	114	114	114	93	93	93	93	93	114	73	73	11	10	116	153	94
SAUR1472 (SAR_RS07360)	N/A	6	6	N/A	60	60	60	60	60	6	50	50	6	6	17	83	17
SAUR1473 (SAR_RS07365)	168	168	168	168	126	126	126	126	126	168	96	96	7	23	177	220	N/A

Locus (cg MLST)	Alternative designation	Position (in ED133)	Study Isolates							CC133			CC692				CC522					ST1347	Chinese CC133 MRSA isolates		CC5	ST71	CC182	CC707	CC1956
			Swan isolate 15V8707	Duck isolate 511525-22	Duck isolate V315	Duck isolate 511509-22	Duck isolate 511510-22	Whistling duck isolate [illegible]	Duck isolate V482	CP001996.1, ED133	CP065353.1, AR39 [illegible]	LR134271.1, NCTC7988	CP042650.1, X22	UHAK01000002.1, NCTC9546	PubMLST ID 39948, 973N0056	CP129360, CKMM-401M	Goat isolate 17CS1042	PubMLST ID 24977	PubMLST ID 24358	PubMLST ID 11704	SAMEA1904171	PubMLST ID 39945, 61290	JAOAKU01, GDQ20D75A	JAFEOM01, GDQ20D75A	CP001781.1, ED98	SAMN03247575, MOK-099	CP070991, CBS2016-05	CP029751, SMITH	CP084892, WT19
SAUR1474 (SAR_RS07370)	cvfC2	1481443	16	16	16	16	16	16	16	16	16	40	54	54	54	54	12	12	12	12	12	54	16	16	6	10	116	16	16
SAUR1475 (SAR_RS07375)	cvfC1	1481706	7	7	N/A	7	7	N/A	7	7	7	7	2	2	2	2	2	2	2	2	2	2	7	7	2	2	7	2	23
SAUR1476 (SAR_RS07380)	Q5HFZ1=yhhQ	1482145	334	334	63	63	63	63	63	320	63	63	N/A	26	26	N/A	189	84	84	84	84	324	63	63	8	N/A	112	85	85
SAUR1477 (SAR_RS07385)	N/A	1483015	2	2	2	2	2	2	2	2	2	2	N/A	N/A	N/A	N/A	N/A	N/A	N/A	N/A	N/A	N/A	2	2	N/A	N/A	2	3	N/A
SAUR1478 (SAR_RS07390)	rnhA	1483091	2	2	N/A	N/A	N/A	N/A	N/A	170	2	2	38	38	38	38	200	200	200	200	200	N/A	2	2	37	41	2	15	2
SAUR1480 (SAR_RS07400)	norB	1513170	33	N/A	33	188	188	33	33	33	33	33	N/A	140	140	140	2	2	2	2	2	N/A	33	33	49	29	142	190	N/A
SAUR1481 (SAR_RS07405)	steT	1514717	34	34	34	34	34	34	34	34	34	34	N/A	150	150	N/A	191	191	191	120	191	N/A	N/A	N/A	7	30	152	194	122
SAUR1482 (SAR_RS07410)	ilvA1=tdcB	1516070	415	415	25	25	25	25	25	25	147	25	N/A	257	257	N/A	N/A	179	45	45	126	N/A	25	25	250	N/A	144	127	30
SAUR1483 (SAR_RS07415)	ald1	1517205	59	59	59	59	59	59	59	75	224	75	N/A	7	7	7	103	268	103	103	103	N/A	N/A	N/A	412	N/A	132	180	104
SAUR1484 (SAR_RS07420)	exo53	1518794	76	76	76	76	76	76	76	76	76	76	122	122	122	122	98	98	98	98	98	122	76	76	8	26	88	158	68
SAUR1487 (SAR_RS07435)	Q5HFY0	1524936	44	44	N/A	N/A	N/A	N/A	N/A	44	44	44	63	63	63	63	3	3	3	3	3	63	44	44	5	24	64	3	1
SAUR1488 (SAR_RS07440)	vpsC	1525351	108	108	108	108	108	108	108	534	108	108	56	56	543	56	140	140	140	140	140	56	108	108	13	N/A	188	226	141
SAUR1489 (SAR_RS07445)	gpsB	1527148	2	2	2	2	2	2	N/A	2	2	2	4	4	4	4	2	2	2	2	2	4	2	2	4	5	2	2	2
SAUR1490 (SAR_RS07450)	vpsA	1527506	25	25	25	N/A	N/A	25	25	25	25	25	9	9	9	9	72	72	72	72	72	9	25	25	8	1	87	113	25
SAUR1491 (SAR_RS07455)	vppE	1528062	47	47	47	47	47	47	47	47	99	47	13	13	13	13	57	57	57	57	57	N/A	N/A	N/A	8	17	18	18	N/A
SAUR1493 (SAR_RS07465)	recU	1528952	2	2	2	2	2	2	2	2	2	2	7	7	7	7	2	2	128	2	2	7	2	2	7	7	79	13	2
SAUR1495 (SAR_RS07475)	Q1YBU7	1532271	40	40	40	40	40	40	40	40	40	40	53	53	53	53	50	50	50	50	50	53	40	40	6	N/A	55	10	2
SAUR1496 (SAR_RS07480)	nth1	1532617	282	282	55	55	55	55	55	234	N/A	55	88	88	88	88	2	2	2	71	2	N/A	55	55	2	2	89	27	14
SAUR1497 (SAR_RS07485)	dnaD	1533266	58	58	58	58	58	58	58	57	58	57	N/A	88	88	N/A	27	27	27	27	27	272	N/A	N/A	9	15	89	113	74
SAUR1498 (SAR_RS07490)	asnS	1534281	93	93	93	93	93	93	N/A	93	93	93	N/A	541	161	161	116	116	274	116	116	161	93	93	8	45	162	194	117
SAUR1499 (SAR_RS07495)	dinG1	1535895	N/A	172	172	172	172	172	172	172	N/A	172	N/A	963	276	N/A	358	358	490	225	358	911	172	172	897	49	277	361	N/A
[illegible]	[illegible]	[illegible]	[illegible]	[illegible]	[illegible]	[illegible]	[illegible]	[illegible]	[illegible]	[illegible]	[illegible]	[illegible]	[illegible]	[illegible]	[illegible]	[illegible]	[illegible]	[illegible]	[illegible]	[illegible]	[illegible]	[illegible]	[illegible]	[illegible]	[illegible]	[illegible]	[illegible]	[illegible]	[illegible]

Locus (cg MLST)	Alternative designation	Position (in ED133)	Study Isolates							CC133			CC692
			Swan isolate 15V8707	Duck isolate 511525-22	Duck isolate V315	Duck isolate 511509-22	Duck isolate 511510-22	Whistling duck isolate	Duck isolate V482	CP001996.1, ED133	CP065353.1, AR39	LR134271.1, NCTC7988	CP042650.1, X22
SAUR1863 (SAR_RS09315)	aroA2	1813306	23	23	N/A	N/A	N/A	N/A	N/A	23	23	23	98
SAUR1866 (SAR_RS09330)	voxC	1816373	34	34	34	34	34	34	34	34	34	34	127
SAUR1867 (SAR_RS09335)	murC	1816938	73	73	73	73	73	73	73	73	73	73	110
SAUR1868 (SAR_RS09340)	Q5HF33	1818275	386	386	386	N/A	N/A	386	386	228	N/A	228	N/A
SAUR1870 (SAR_RS09350)	Q5HF31	1822745	63	63	63	63	N/A	63	63	335	63	63	6
SAUR1871 (SAR_RS09355)	ytpP	1823702	26	26	26	26	26	26	26	26	26	26	6
SAUR1872 (SAR_RS09360)	pepA1	1824078	38	N/A	38	38	38	38	38	407	243	38	28
SAUR1873 (SAR_RS09365)	N/A	1825238	1	1	1	1	1	1	N/A	1	1	1	1
SAUR1874 (SAR_RS09370)	ytnP	1825677	29	29	N/A	N/A	N/A	N/A	N/A	29	29	29	N/A
SAUR1881 (SAR_RS09405)	trmB	1826987	40	40	2	2	2	2	2	2	2	2	13
SAUR1882 (SAR_RS09410)	ytmP	1827646	256	256	53	53	53	53	53	53	53	53	18
SAUR1883 (SAR_RS09415)	daaA=dat	1828988	73	N/A	73	73	N/A	N/A	73	73	73	73	11
SAUR1884 (SAR_RS09420)	ytiP=sapep	1829840	2	2	2	N/A	N/A	2	2	2	2	2	N/A
SAUR1885 (SAR_RS09425)	Q5HF22	1831738	49	N/A	49	49	49	49	N/A	49	49	49	3
SAUR1886 (SAR_RS09430)	rsuA	1832177	62	62	N/A	N/A	N/A	N/A	N/A	62	62	62	15
SAUR1887 (SAR_RS09435)	ytgP	1832869	85	85	85	85	N/A	85	N/A	85	85	85	520
SAUR1888 (SAR_RS09440)	Q1Y9Y5	1834937	96	96	96	96	96	96	96	96	96	96	153
SAUR1889 (SAR_RS09445)	sasC	N/A	N/A	N/A	N/A	N/A	N/A	N/A	N/A	N/A	N/A	N/A	N/A
SAUR1890 (SAR_RS09450)	ytwF=moeB	1843206	2	2	2	N/A	N/A	2	2	2	2	2	42
SAUR1891 (SAR_RS09455)	leuS	1843539	142	N/A	142	142	142	142	142	142	392	142	28
SAUR1892 (SAR_RS09460)	yttB	1846244	77	77	77	77	77	77	95	77	N/A	77	17
SAUR1893 (SAR_RS09465)	ytgA	1847535	65	65	N/A	65	65	65	N/A	65	65	65	373
SAUR1894 (SAR_RS09470)	ytgB	1848485	12	12	12	12	12	12	12	12	12	12	69
SAUR1895 (SAR_RS09475)	rot	1849168	35	35	35	35	35	35	35	35	35	35	5
SAUR1896 (SAR_RS09480)	pldB1	1850140	25	25	N/A	25	25	25	25	25	25	25	353
SAUR1897 (SAR_RS09485)	N/A	1850970	1	1	1	N/A	1	1	1	1	1	1	3
SAUR1898 (SAR_RS09490)	putA	1851201	88	88	88	88	88	88	88	88	88	88	8
SAUR1899 (SAR_RS09495)	ribH=ribE	1852324	41	41	41	41	41	41	41	41	41	41	8
SAUR1900 (SAR_RS09500)	ribA	1852801	74	74	N/A	74	N/A	N/A	N/A	353	74	74	119
SAUR1901 (SAR_RS09505)	ribB	1853993	N/A	59	59	59	59	59	59	59	59	59	19
SAUR1902 (SAR_RS09510)	ribD	1854632	90	90	90	90	90	90	90	404	226	90	144
SAUR1903 (SAR_RS09515)	Q5HF04	N/A	N/A	N/A	N/A	N/A	N/A	N/A	N/A	N/A	N/A	N/A	N/A
SAUR1904 (SAR_RS09520)	arsR	1858180	18	18	18	18	18	18	18	18	18	18	11
SAUR1905 (SAR_RS09525)	N/A	1858494	73	73	73	73	73	73	73	73	73	73	144
SAUR1906 (SAR_RS09530)	sagB=gad	1860550	2	N/A	N/A	N/A	N/A	N/A	N/A	235	2	2	95
SAUR1907 (SAR_RS09535)	Q5HEZ9	1861680	9	9	N/A	N/A	N/A	N/A	N/A	9	9	9	5
SAUR1908 (SAR_RS09540)	sigS	1862103	30	30	30	30	30	N/A	N/A	30	30	30	N/A
SAUR1910 (SAR_RS09550)	Q5HEZ6	1863116	97	97	97	97	97	97	97	97	88	97	69
SAUR1913 (SAR_RS09565)	tal	1864929	25	25	25	25	25	25	N/A	25	25	25	82
SAUR1914 (SAR_RS09570)	Q7A500	1865900	140	140	19	N/A	19	19	19	19	19	19	15
SAUR1915 (SAR_RS09575)	crcB1	1866458	19	19	19	19	19	19	19	19	19	19	55
SAUR1916 (SAR_RS09580)	crcB2	1866820	44	44	44	N/A	44	44	44	44	44	44	61
SAUR1917 (SAR_RS09585)	ytbE=yygN	1867423	92	92	92	92	92	92	92	92	92	92	136
SAUR1918 (SAR_RS09590)	nrd	1868468	80	80	80	80	80	80	N/A	80	192	80	202
SAUR1919 (SAR_RS09595)	metK	1869501	78	78	78	78	78	78	78	78	78	78	254
SAUR1920 (SAR_RS09600)	pckA	1871065	97	97	97	97	97	97	N/A	97	97	97	150
SAUR1921 (SAR_RS09605)	ytmA	1872952	37	37	37	37	37	37	37	37	37	37	282
SAUR1922 (SAR_RS09610)	rppH=nudH=ytkD	1873703	13	13	13	13	13	13	13	13	13	13	4
SAUR1923 (SAR_RS09615)	ytiA=yidD	1874242	2	2	2	2	2	2	2	2	2	2	3
SAUR1924 (SAR_RS09620)	menC	1874496	74	74	74	74	74	74	74	74	74	74	128
SAUR1925 (SAR_RS09625)	menE	1875502	80	80	80	N/A	N/A	80	80	80	80	80	181
SAUR1926 (SAR_RS09630)	Q5HEY1	1877139	52	52	52	52	52	52	52	52	52	52	94
SAUR1927 (SAR_RS09635)	Q5HEY0	1877933	282	282	29	29	29	29	29	29	29	29	N/A
SAUR1928 (SAR_RS09640)	DUF4352	1878628	68	68	N/A	N/A	N/A	N/A	68	68	68	68	N/A
SAUR2941	Q99SP1	N/A	N/A	N/A	N/A	N/A	N/A	N/A	4	N/A	N/A	N/A	N/A
SAUR1973 (SAR_RS09865)	ydeN	1915258	42	42	42	42	42	42	42	42	42	42	63
SAUR1975 (SAR_RS09875)	hemY	1916131	N/A	N/A	104	104	104	104	N/A	77	277	77	167
SAUR1976 (SAR_RS09880)	hemH	1917555	88	N/A	88	88	88	88	88	88	88	88	24
SAUR1977 (SAR_RS09885)	hemE	1918536	91	91	N/A	N/A	N/A	N/A	N/A	91	91	91	11
SAUR1978 (SAR_RS09890)	N/A	1919563	1	1	1	1	1	1	1	1	1	1	N/A
SAUR1979 (SAR_RS09895)	traP	1919835	34	34	34	34	34	34	34	34	34	34	124
SAUR1980 (SAR_RS09900)	ecsB	1920462	95	95	95	N/A	N/A	95	95	101	95	101	513
SAUR1981 (SAR_RS09905)	ecsA	1921678	31	31	31	31	31	31	N/A	80	31	80	127
SAUR1982 (SAR_RS09910)	hit	1922552	2	N/A	2	2	2	2	2	2	2	2	7
SAUR1983 (SAR_RS09915)	gvpP	1923116	43	43	43	43	43	43	43	43	43	43	13
SAUR1984 (SAR_RS09920)	yhaJ	1924213	54	54	54	N/A	N/A	54	54	54	54	54	6
SAUR1985 (SAR_RS09925)	prsA2	1924975	70	N/A	70	70	70	70	70	70	70	70	N/A
SAUR1986 (SAR_RS09930)	cbf=yhaM	1926058	289	289	36	36	36	36	36	36	141	36	86
SAUR1987 (SAR_RS09935)	Q5HET2	1926996	144	144	144	N/A	144	144	N/A	144	455	144	261
SAUR1988 (SAR_RS09940)	Q5HET1	1929922	421	421	29	29	29	29	29	29	29	29	123
SAUR1990 (SAR_RS09950)	UPF0342	1932196	1	1	1	1	1	1	1	1	1	1	26
SAUR1991 (SAR_RS09955)	UPF0754	1932609	108	108	N/A	N/A	N/A	N/A	N/A	108	108	108	155
SAUR1992 (SAR_RS09960)	N/A	1933912	1	1	1	1	1	1	1	1	1	1	36
SAUR1993 (airR)	N/A	1934732	2	2	2	2	2	2	N/A	242	2	2	7
SAUR1994 (airS)	N/A	1935377	76	76	76	76	76	76	76	76	76	76	216
SAUR1995 (SAR_RS09975)	rluA2	1936652	62	62	62	62	62	62	62	62	62	62	117
SAUR1996 (SAR_RS09980)	fumC=citG	1937924	76	76	76	N/A	76	76	76	76	232	76	129
SAUR1997 (SAR_RS09985)	Q5HES3	1939505	2	2	2	N/A	2	2	2	2	2	2	48
SAUR1999 (SAR_RS09995)	Q5HES1	1940643	1	N/A	1	1	1	1	1	1	1	1	5
SAUR2000 (SAR_RS10000)	Q5HES0	1940820	2	2	2	2	2	2	2	2	2	2	N/A
SAUR2001 (SAR_RS10005)	cspR=trmL	1941578	33	33	33	33	33	33	33	33	33	33	4
SAUR2002 (SAR_RS10010)	queG	1942053	111	111	111	111	111	111	111	111	111	192	N/A
SAUR2003 (SAR_RS10015)	artR	1943331	49	49	49	49	49	49	49	49	49	49	99
SAUR2004 (SAR_RS10020)	artQ	1944046	89	89	89	N/A	89	89	89	89	89	89	172
SAUR2005 (SAR_RS10025)	Q1Y9N4	1945761	26	26	26	N/A	26	26	N/A	26	26	26	N/A
SAUR2036 (SAR_RS10180)	perR	1955693	2	2	N/A	N/A	N/A	N/A	N/A	2	2	2	1
SAUR2037 (SAR_RS10185)	Q5HER2	1956236	107	107	107	107	107	107	107	107	107	107	N/A
SAUR2038 (SAR_RS10190)	bcp	1957192	50	50	50	50	50	50	50	50	50	50	13
SAUR2039 (SAR_RS10195)	gsaB	1957728	76	76	76	76	76	76	76	76	76	76	N/A
SAUR2040 (SAR_RS10200)	DUF0939-L1	1959310	21	21	21	N/A	21	21	21	21	21	21	8
SAUR2042 (SAR_RS10210)	msbA2	1960594	133	N/A	N/A	133	133	N/A	133	729	133	133	216
SAUR2043 (SAR_RS10215)	DUF402	1962622	10	10	10	10	10	10	10	10	10	10	8
SAUR2044 (SAR_RS10220)	mutY	1963467	37	37	37	37	37	37	37	76	37	76	125
SAUR2045 (SAR_RS10225)	DUF457	1964656	82	82	N/A	N/A	N/A	N/A	N/A	82	82	82	7
SAUR2046 (SAR_RS10230)	N/A	1965737	2	2	2	2	2	2	2	2	2	2	8
SAUR2047 (SAR_RS10235)	tagG	1965894	80	80	80	N/A	80	80	80	149	192	78	18
SAUR2048 (SAR_RS10240)	tagH	1966739	611	611	104	104	104	104	104	104	104	104	N/A
SAUR2049 (SAR_RS10245)	yfhH	1968271	2	2	2	2	2	2	2	2	2	2	7
SAUR2050 (SAR_RS10250)	recX	1968563	388	388	78	N/A	78	78	78	78	78	78	120
SAUR2051 (SAR_RS10255)	sgtB	1969642	N/A	2	2	2	2	2	N/A	2	2	2	N/A
SAUR2052 (SAR_RS10260)	pfoI	1972454	2	2	2	2	2	2	2	2	2	2	2
SAUR2053 (SAR_RS10265)	Q5HEP8	1973099	1	1	1	1	1	1	1	1	1	1	16
SAUR2054 (SAR_RS10270)	A5IU43=vfkAB	N/A	116	116	116	116	116	116	N/A	N/A	116	N/A	120
SAUR2055 (SAR_RS10275)	A5IU44	2019913	46	46	46	46	46	46	46	46	46	46	73
SAUR2056 (SAR_RS10280)	ampS	2020533	106	106	106	106	106	106	106	106	106	106	168
SAUR2057 (SAR_RS10285)	Q5HEP4	2021792	26	26	26	26	26	26	26	26	26	26	1
SAUR2058 (SAR_RS10290)	ptpA	2022139	2	2	2	2	2	2	2	2	2	2	5
SAUR2059 (SAR_RS10295)	Q5HEP2	2022610	3	3	3	3	3	3	3	3	3	3	4
SAUR2060 (SAR_RS10300)	rbn	2023163	81	81	81	81	81	81	81	81	81	81	8
SAUR2061 (vraR)	N/A	2024493	2	2	2	N/A	N/A	2	2	2	2	2	5
SAUR2062 (vraS)	N/A	2025112	29	29	29	29	29	29	N/A	29	29	29	19
SAUR2063 (yvqF)	N/A	2026152	25	25	25	N/A	N/A	25	25	25	25	25	13
SAUR2064 (MW1827)	N/A	2026868	27	27	27	27	27	27	27	27	27	27	5
SAUR2065 (SAR_RS10325)	ampM	2027471	22	22	22	22	22	22	22	22	22	22	74
SAUR2067 (SAR_RS10335)	N/A	2029979	22	22	22	N/A	N/A	22	N/A	22	22	22	81
SAUR2068 (SAR_RS10340)	N/A	N/A	N/A	N/A	N/A	N/A	N/A	N/A	N/A	N/A	N/A	N/A	1
SAUR2069 (SAR_RS10345)	gatD	2030619	65	65	65	N/A	N/A	65	65	65	65	65	N/A
SAUR2070 (SAR_RS10350)	murT	2031352	34	34	34	34	34	34	34	34	34	34	10
SAUR2071 (SAR_RS10355)	ftnA	2032957	2	2	2	2	2	2	2	2	2	2	10
SAUR2072 (SAR_RS10360)	N/A	2033539	1	1	1	1	1	1	1	1	1	1	1
SAUR2073 (SAR_RS10365)	dinG2	2033848	215	215	2	2	2	2	2	2	2	56	6
SAUR2074 (SAR_RS10370)	dinB	2034469	71	71	71	71	N/A	71	71	71	71	71	9
SAUR2075 (SAR_RS10375)	Q5HEM6	2035785	2	2	N/A	N/A	N/A	N/A	2	2	2	2	6
SAUR2076 (SAR_RS10380)	rlmCD	2036485	17	17	17	17	17	17	17	17	17	17	77
SAUR2077 (SAR_RS10385)	dagK	2037927	11	11	11	11	11	11	11	11	11	11	6
SAUR2078 (SAR_RS10390)	N/A	2039511	10	10	10	N/A	10	10	10	10	10	10	8
SAUR2079 (SAR_RS10395)	gatB	2039696	82	82	82	82	82	82	82	82	82	82	215
SAUR2080 (SAR_RS10400)	gatA	2041136	81	81	81	81	81	81	81	81	81	81	120
SAUR2081 (SAR_RS10405)	N/A	2042595	1	1	1	1	1	1	1	1	42	1	25
SAUR2082 (SAR_RS10410)	putP	2043263	42	42	N/A	N/A	N/A	N/A	42	42	42	42	181
SAUR2083 (SAR_RS10415)	camS	2046680	83	83	83	N/A	83	83	N/A	83	N/A	83	428
SAUR2084 (SAR_RS10420)	ligA	2047892	626	626	100	100	100	100	100	100	100	100	181
SAUR2085 (SAR_RS10425)	pcrA	2049899	115	115	N/A	115	115	N/A	N/A	115	115	115	515
SAUR2086 (SAR_RS10430)	pcrB	2052088	26	26	26	26	26	26	26	26	26	26	38
SAUR2087 (SAR_RS10435)	yerC	2052952	2	2	2	2	2	2	2	2	2	2	2
SAUR2088 (SAR_RS10440)	purB	2053363	69	69	69	N/A	69	69	69	69	69	69	118
SAUR2089 (SAR_RS10445)	sspP=scpA	2055519	96	96	96	N/A	N/A	96	N/A	96	96	96	17
SAUR2091 (SAR_RS10455)	Q5HEL1	2057333	10	10	10	10	10	10	10	10	10	10	5
SAUR2092 (SAR_RS10460)	UPF0316	2057487	6	6	6	6	6	6	6	6	6	6	5
SAUR2093 (SAR_RS10465)	nadE	2058359	60	60	60	60	60	60	60	60	60	60	9
SAUR2094 (SAR_RS10470)	nadC	2059173	488	488	96	96	96	96	96	96	96	96	17
SAUR2095 (SAR_RS10475)	nos	2060826	3	3	3	3	3	3	3	3	3	3	104
SAUR2096 (SAR_RS10480)	pdt	2061922	37	37	37	37	37	37	37	37	37	37	64
SAUR2098 (SAR_RS10490)	sdcS	2062906	118	118	118	118	118	118	118	118	118	118	8
SAUR2099 (SAR_RS10495)	Q5HEK3=pfbA	2064674	78	78	N/A	N/A	N/A	N/A	N/A	78	78	78	406
SAUR2100 (SAR_RS10500)	pncA	2066143	3	3	3	3	3	3	3	3	3	3	3
SAUR2101 (SAR_RS10505)	ppaC	2066756	19	19	19	19	19	19	N/A	19	19	19	72
SAUR2102 (SAR_RS10510)	N/A	2067878	2	2	2	N/A	2	2	2	2	2	2	3
SAUR2103 (SAR_RS10515)	aldH	2068103	100	100	100	100	100	100	100	100	100	100	18
SAUR2104 (SAR_RS10520)	Q5HEJ8	2069602	73	73	73	73	73	73	73	73	73	73	122
SAUR2105 (SAR_RS10525)	Q5HEJ7	2070901	13	13	N/A	N/A	N/A	N/A	N/A	13	13	N/A	1
SAUR2106 (SAR_RS10530)	A6U329	2071359	2	2	2	2	2	N/A	2	2	2	2	2
SAUR2107 (SAR_RS10535)	A6U330	2071983	75	75	75	N/A	75	75	75	400	75	75	N/A
SAUR2108 (SAR_RS10540)	Q5HEJ4	2073080	2	2	2	N/A	N/A	2	2	2	2	2	70
SAUR2109 (SAR_RS10545)	DUF1700	2073919	4	4	4	4	4	4	4	4	4	4	11
SAUR2110 (SAR_RS10550)	Q5HEJ2	2074536	32	32	32	32	32	32	32	32	32	32	22
SAUR2111 (SAR_RS10555)	Q5HEJ1	2075181	2	2	2	2	2	2	2	2	2	36	N/A
SAUR2112 (SAR_RS10560)	pmtD	2075810	47	47	47	N/A	47	47	47	47	47	N/A	9
SAUR2113 (SAR_RS10565)	pmtC	2076550	47	47	47	47	47	47	47	47	47	47	8
SAUR2114 (SAR_RS10570)	pmtB	2077419	52	52	52	N/A	52	52	52	52	52	52	8
SAUR2115 (SAR_RS10575)	pmtA	2078100	2	2	2	2	2	2	2	282	2	2	N/A
SAUR2116 (SAR_RS10580)	pmtR	2078993	2	2	2	2	2	2	2	2	2	2	73
SAUR2117 (SAR_RS10585)	A6U340	2079631	1	1	1	N/A	1	1	1	1	1	1	26
SAUR2118 (SAR_RS10590)	N/A	2080049	2	2	2	N/A	2	2	2	2	2	2	2
SAUR2119 (SAR_RS10595)	Q5HEI3	2080347	65	65	65	65	65	65	N/A	65	65	65	N/A
SAUR2122 (SAR_RS10610)	N/A	N/A	N/A	N/A	N/A	N/A	N/A	N/A	N/A	N/A	N/A	N/A	N/A
SAUR2190 (SAR_RS10950)	N/A	2084201	N/A	N/A	N/A	60	60	N/A	60	60	N/A	60	N/A
SAUR2191 (SAR_RS10955)	lukX=lukG	2085597	362	N/A	113	113	113	113	113	113	113	113	151
SAUR2192 (SAR_RS10960)	lukY=lukH	2086635	108	108	108	108	108	108	N/A	108	108	108	145
SAUR2193 (SAR_RS10965)	dapE	2088126	83	83	83	83	83	83	83	83	83	83	N/A
SAUR2194 (SAR_RS10970)	ktrB	2092610	105	105	105	N/A	N/A	N/A	105	105	105	105	250
SAUR2199 (SAR_RS10995)	groL	2110042	25	25	25	25	25	25	25	25	25	25	244
SAUR2200 (SAR_RS11000)	groS	2111734	10	10	10	10	10	10	10	10	10	10	7
SAUR2201 (SAR_RS11005)	ydiL=mroQ	2112193	29	29	29	N/A	29	29	74	29	29	29	N/A
SAUR2202 (SAR_RS11010)	sdrH	2112961	1219	1219	N/A	N/A	N/A	N/A	N/A	1182	N/A	N/A	N/A
SAUR2204 (SAR_RS11020)	mtnU=ramA	2115339	74	74	74	74	74	74	74	74	74	74	8
SAUR2953 (agrB)	N/A	2117094	49	49	49	N/A	49	N/A	49	49	49	49	2
SAUR2954 (agrD1)	N/A	2117660	1	1	1	1	1	1	1	1	1	1	1
SAUR2958 (agrC)	N/A	N/A	N/A	N/A	951	N/A	951	N/A	N/A	N/A	N/A	N/A	853
SAUR2959 (agrA)	N/A	N/A	N/A	N/A	105	N/A	105	N/A	N/A	N/A	N/A	N/A	1
SAUR2210 (SAR_RS11050)	scrK=frk	2120219	74	74	74	74	74	74	74	74	74	74	N/A
SAUR2211 (SAR_RS11055)	scrB	2121175	103	103	103	103	103	103	N/A	103	293	103	164
SAUR2212 (SAR_RS11060)	scrR	2122808	405	405	2	2	2	2	2	2	190	2	7
SAUR2213 (SAR_RS11065)	nrgA	2123939	562	562	77	77	77	77	N/A	77	77	77	149
SAUR2214 (SAR_RS11070)	Q5HEF7=tusA	2125398	21	21	21	21	21	21	21	21	21	21	N/A
SAUR2215 (SAR_RS11075)	N/A	2125682	79	79	79	79	79	79	79	79	79	79	117
SAUR2216 (SAR_RS11080)	rex	2127066	35	35	35	35	35	35	35	35	35	35	2
SAUR2217 (SAR_RS11085)	yheS	2127954	125	125	125	N/A	N/A	125	N/A	125	125	125	N/A
SAUR2220 (SAR_RS11100)	tsaD	2132284	51	51	51	51	51	51	51	353	51	73	N/A
SAUR2221 (SAR_RS11105)	rimI	2133302	35	35	35	35	35	35	35	35	35	35	6
SAUR2222 (SAR_RS11110)	tsaB	2133739	25	25	25	25	25	25	N/A	25	25	25	50
SAUR2223 (SAR_RS11115)	N/A	2134382	23	23	N/A	N/A	N/A	N/A	N/A	23	23	23	16
SAUR2224 (SAR_RS11120)	N/A	2134881	2	2	2	N/A	2	2	2	2	2	2	6
SAUR2225 (SAR_RS11125)	ilvD	2135354	109	109	N/A	109	109	109	N/A	618	109	109	N/A
SAUR2226 (SAR_RS11130)	ilvB	2137070	129	129	129	129	129	129	129	120	129	129	N/A
SAUR2227 (SAR_RS11135)	ilvH	2138839	2	2	2	2	2	2	2	126	2	2	10
SAUR2228 (SAR_RS11140)	ilvC	2139230	25	25	N/A	25	25	N/A	25	25	25	25	13
SAUR2229 (SAR_RS11145)	leuA1	2140264	127	127	127	127	127	127	127	127	127	127	55
SAUR2230 (SAR_RS11150)	leuB	2141796	97	97	97	N/A	N/A	97	97	96	97	97	17
SAUR2231 (SAR_RS11155)	leuC	2142856	134	134	134	134	134	134	134	134	134	134	23
SAUR2232 (SAR_RS11160)	leuD	2144227	29	29	29	29	29	29	29	29	29	29	N/A
SAUR2233 (SAR_RS11165)	ilvA2	2144814	42	42	42	42	42	42	42	42	42	42	N/A
SAUR2240 (SAR_RS11200)	sprL	2153084	19	19	19	19	19	19	19	19	19	19	61
SAUR2241 (SAR_RS11205)	yhgF	2153532	147	147	147	147	147	147	147	147	147	147	N/A
SAUR2242 (SAR_RS11210)	sigB	2156116	73	73	73	73	73	73	73	73	186	73	N/A
SAUR2243 (SAR_RS11215)	rsbW	2156861	2	2	2	2	2	2	N/A	2	2	2	7
SAUR2244 (SAR_RS11220)	rsbV	2157342	2	2	2	2	2	2	2	2	2	2	46
SAUR2245 (SAR_RS11225)	rsbU	2157787	2	2	2	2	N/A	2	2	2	2	2	33
SAUR2246 (SAR_RS11230)	N/A	2158940	2	2	2	N/A	N/A	N/A	2	2	2	2	4
SAUR2247 (SAR_RS11235)	mazF	2159138	3	3	3	3	3	3	3	3	3	3	6
SAUR2248 (SAR_RS11240)	N/A	2159497	1	1	1	1	1	1	1	1	1	1	1
SAUR2249 (SAR_RS11245)	alr1	2159752	79	79	79	79	79	79	79	79	79	79	131
SAUR2250 (SAR_RS11250)	acpS	2160966	16	16	16	16	16	16	16	16	16	16	10
SAUR2251 (SAR_RS11255)	N/A	2161329	59	59	59	59	59	59	59	59	59	59	N/A
SAUR2252 (SAR_RS11260)	N/A	2161813	12	12	12	12	12	12	12	12	46	16	N/A
SAUR2253 (SAR_RS11265)	Q9ZAH9	2163383	37	37	37	37	37	37	37	37	37	37	N/A
SAUR2254 (SAR_RS11270)	kdpC-chr	2164071	2	2	2	2	2	2	2	2	2	2	75
SAUR2255 (SAR_RS11275)	kdpB-chr	2164651	132	132	132	N/A	N/A	132	N/A	132	132	132	N/A
SAUR2256 (SAR_RS11280)	kdpA-chr	2166697	118	118	N/A	N/A	N/A	N/A	N/A	118	118	118	N/A
SAUR2257 (SAR_RS11285)	kdpF-chr	2168397	18	18	18	18	18	18	18	18	18	18	3
SAUR2258 (SA0067)	N/A	2168645	156	156	156	N/A	156	156	N/A	156	156	156	N/A
SAUR2259 (SA0066)	N/A	2171302	45	45	45	45	45	45	45	43	45	43	16
SAUR2260 (SAR_RS11300)	cshA	2172353	95	95	95	95	95	95	95	95	95	95	25
SAUR2261 (SAR_RS11305)	murF	2174390	93	361	93	N/A	N/A	93	93	93	93	93	26
SAUR2262 (SAR_RS11310)	ddlA	2175763	79	79	N/A	N/A	N/A	N/A	N/A	79	79	79	16
SAUR2263 (SAR_RS11315)	ftsW1=rodA	2177151	92	92	92	92	N/A	92	92	92	92	92	147
SAUR2265 (SAR_RS11325)	N/A	2178620	1	1	1	1	N/A	1	1	1	1	1	1
SAUR2266 (SAR_RS11330)	csoZ	2178885	15	15	15	15	15	15	15	15	15	15	5
SAUR2267 (SAR_RS11335)	csoR	2179106	12	12	N/A	12	12	12	12	12	12	12	N/A
SAUR2268 (SAR_RS11340)	cls2	2179565	581	581	110	110	110	110	110	516	110	110	9
SAUR2269 (SAR_RS11345)	yedJ	2181077	53	53	53	53	53	53	53	256	53	53	13
SAUR2271 (SAR_RS11355)	yidC	2182461	52	52	52	52	52	52	52	242	52	99	13
SAUR2272 (SAR_RS11360)	thiE	2183419	24	24	24	24	24	24	24	24	24	24	N/A
SAUR2273 (SAR_RS11365)	thiM	2184062	349	349	75	75	75	75	75	75	75	75	21
SAUR2274 (SAR_RS11370)	thiD2	2184837	36	36	36	36	36	36	N/A	36	36	36	348
SAUR2275 (SAR_RS11375)	tenA	2185660	318	318	10	10	10	10	10	10	10	10	14
SAUR2276 (SAR_RS11380)	N/A	2186449	1	1	1	N/A	1	1	1	1	1	1	3
SAUR2277 (SAR_RS11385)	sceD	2186736	63	63	63	N/A	N/A	63	63	63	63	63	12
SAUR2278 (SAR_RS11390)	Q5HEA3	2187820	2	2	2	N/A	2	2	2	2	2	2	2
SAUR2279 (SAR_RS11395)	ywpF	2188409	2	2	2	2	2	2	2	2	2	2	7
SAUR2280 (SAR_RS11400)	fabZ	2188906	38	38	38	38	38	38	38	37	38	37	6
SAUR2281 (SAR_RS11405)	murA	2189380	27	27	27	27	27	27	27	27	27	27	390
SAUR2282 (SAR_RS11410)	N/A	2190756	2	2	2	2	2	2	2	2	2	2	2
SAUR2284 (SAR_RS11420)	atpE	2191610	2	2	N/A	N/A	N/A	N/A	N/A	2	2	2	4
SAUR2285 (SAR_RS11425)	atpD	2192034	78	78	78	78	78	78	78	78	78	78	N/A
SAUR2286 (SAR_RS11430)	atpG	2193468	1	1	N/A	N/A	N/A	N/A	1	1	1	112	31
SAUR2287 (SAR_RS11435)	atpA	2194365	44	44	44	44	44	44	44	44	44	44	20
SAUR2288 (SAR_RS11440)	atpH	2195895	50	50	50	50	50	50	50	50	50	50	32
SAUR2289 (SAR_RS11445)	atpF	2196434	2	2	N/A	154	154	154	154	2	2	2	62
SAUR2290 (SAR_RS11450)	N/A	2197154	1	1	1	1	1	1	1	1	1	1	1
SAUR2291 (SAR_RS11455)	atpB	2197409	3	3	3	3	3	3	3	3	3	3	8
SAUR2292 (SAR_RS11460)	atpI	2198158	32	32	N/A	N/A	N/A	N/A	N/A	32	32	32	6
SAUR2293 (SAR_RS11465)	mnaA	2198678	357	357	357	357	357	357	357	357	357	357	112
SAUR2294 (SAR_RS11470)	upp	2199829	17	17	17	17	17	17	17	17	17	17	11
SAUR2295 (SAR_RS11475)	glyA	2200486	107	107	N/A	107	107	107	107	102	107	102	8
SAUR2296 (SAR_RS11480)	ywlG	2201751	N/A	N/A	54	54	54	54	54	54	54	54	34
SAUR2297 (SAR_RS11485)	ptpB	2202382	12	12	12	12	12	12	12	12	12	12	11
SAUR2298 (SAR_RS11490)	tsaC	2202798	79	79	79	79	79	79	79	79	79	79	40
SAUR2299 (SAR_RS11495)	prmC	2203928	71	71	71	71	71	71	71	71	71	71	8
SAUR2300 (SAR_RS11500)	prfA	2204751	62	62	62	62	62	62	62	62	62	62	N/A
SAUR2301 (SAR_RS11505)	tdk	2205828	49	49	49	49	49	49	49	49	49	49	6
SAUR2302 (SAR_RS11510)	N/A	2206420	2	2	2	N/A	2	2	2	2	2	2	2
SAUR2303 (SAR_RS11515)	N/A	2206773	1	1	1	1	1	1	1	1	1	1	1
SAUR2304 (SAR_RS11520)	rho	2207145	91	91	91	91	91	91	91	91	91	91	16
SAUR2305 (SAR_RS11525)	aldA3	2208708	130	130	N/A	N/A	N/A	N/A	N/A	699	356	130	715
SAUR2306 (SAR_RS11530)	arxR	2210374	10	10	10	10	10	10	10	10	10	10	6
SAUR2307 (SAR_RS11535)	murZ	2210798	116	116	116	116	116	116	116	116	116	116	27
SAUR2308 (SAR_RS11540)	fbaA	2212523	57	57	57	57	57	57	57	57	57	57	1
SAUR2309 (SAR_RS11545)	DUF2529	2213601	18	18	18	N/A	N/A	18	18	18	18	18	7
SAUR2310 (SAR_RS11550)	pyrG	2214231	115	115	115	115	N/A	115	N/A	115	115	115	62
SAUR2311 (SAR_RS11555)	rpoE	2216177	2	2	2	148	148	2	2	198	148	2	N/A
SAUR2312 (SAR_RS11560)	Q5HE71	2216819	62	62	62	62	62	62	62	62	62	62	16
SAUR2313 (SAR_RS11565)	coaW	2218104	76	76	76	N/A	N/A	76	76	76	76	76	16
SAUR2314 (SAR_RS11570)	DUF2750	2219037	2	2	2	2	2	2	2	2	2	2	156
SAUR2315 (SAR_RS11575)	A5IUS7	2220107	93	93	93	93	93	93	93	93	93	93	526
SAUR2316 (SAR_RS11580)	hmrA	2221300	33	33	33	33	33	33	N/A	498	33	33	47
SAUR2317 (SAR_RS11585)	luxS	2222794	2	2	2	2	N/A	2	2	2	2	2	5
SAUR2318 (SAR_RS11590)	Q5HE65	2223416	13	13	13	13	13	13	13	13	13	13	5
SAUR2319 (SAR_RS11595)	pdp	2223780	109	109	109	109	109	109	109	109	109	109	15

Locus (cg MLST)	CC692			CC522					ST1347	Chinese CC133 MRSA isolates		CC5	ST71	CC182	CC707	CC1956
	UHAK01000002.1, NCTC9546	PubMLST ID 39948, 973N0056	CP129360, CKMM-401M	Goat isolate 17CS1042	PubMLST ID 24977	PubMLST ID 24358	PubMLST ID 11704	SAMEA1904171	PubMLST ID 39945, 61290	JAOAKU01, GDQ20D75A	JAFEOM01, GDQ20D75A	CP001781.1, ED98	SAMN03247575, MOK-099	CP070991, CBS2016-05	CP029751, SMITH	CP084892, WT19
SAUR1863 (SAR_RS09315)	98	98	98	79	79	79	79	79	98	23	23	6	6	102	132	N/A
SAUR1866 (SAR_RS09330)	5	173	127	14	14	14	14	14	5	34	34	5	4	14	14	14
SAUR1867 (SAR_RS09335)	110	110	110	87	87	87	87	87	110	73	73	6	24	73	143	51
SAUR1868 (SAR_RS09340)	1420	1420	N/A	N/A	309	779	309	309	1420	386	386	1191	59	418	534	N/A
SAUR1870 (SAR_RS09350)	6	6	6	82	199	82	82	82	6	63	63	6	6	103	133	83
SAUR1871 (SAR_RS09355)	6	6	N/A	53	53	53	N/A	53	6	26	26	5	6	1	1	1
SAUR1872 (SAR_RS09360)	28	28	28	118	271	118	118	118	28	38	38	368	17	146	167	119
SAUR1873 (SAR_RS09365)	1	1	1	1	1	1	1	1	1	N/A	N/A	1	5	39	1	1
SAUR1874 (SAR_RS09370)	435	126	126	N/A	166	166	103	166	126	29	29	9	17	131	168	N/A
SAUR1881 (SAR_RS09405)	13	58	13	64	64	134	64	64	13	2	2	10	3	2	2	2
SAUR1882 (SAR_RS09410)	18	18	18	64	64	132	64	64	18	53	53	6	6	2	14	2
SAUR1883 (SAR_RS09415)	11	11	11	31	31	31	31	31	11	73	73	9	25	115	152	2
SAUR1884 (SAR_RS09420)	153	153	N/A	129	129	129	129	129	153	2	2	8	30	479	205	2
SAUR1885 (SAR_RS09425)	3	3	3	23	23	23	23	91	3	49	49	3	14	72	92	N/A
SAUR1886 (SAR_RS09430)	15	15	15	30	30	30	30	30	15	62	62	9	23	83	122	83
SAUR1887 (SAR_RS09435)	554	523	520	107	107	107	107	107	520	N/A	N/A	471	13	141	102	108
SAUR1888 (SAR_RS09440)	153	153	N/A	125	125	125	125	125	N/A	96	96	4	102	N/A	204	77
SAUR1889 (SAR_RS09445)	1829	1743	N/A	N/A	1808	1808	1808	1811	N/A	N/A	N/A	1705	136	N/A	N/A	N/A
SAUR1890 (SAR_RS09450)	42	42	42	2	2	2	2	2	42	2	2	38	42	2	91	75
SAUR1891 (SAR_RS09455)	28	803	28	188	188	188	188	188	28	142	142	13	47	233	304	189
SAUR1892 (SAR_RS09460)	17	480	17	112	112	112	112	112	17	N/A	N/A	381	17	144	186	113
SAUR1893 (SAR_RS09465)	212	212	373	17	17	17	17	17	212	N/A	N/A	12	11	125	164	104
SAUR1894 (SAR_RS09470)	69	69	69	22	22	22	22	22	N	N/A	N/A	5	8	12	89	12
SAUR1895 (SAR_RS09475)	5	5	5	22	22	22	22	22	5	35	35	6	5	65	2	22
SAUR1896 (SAR_RS09480)	379	108	353	4	4	4	4	4	354	25	25	8	10	2	144	N/A
SAUR1897 (SAR_RS09485)	3	3	3	1	1	1	1	1	3	1	1	3	3	1	1	1
SAUR1898 (SAR_RS09490)	8	8	8	116	116	116	116	116	8	88	88	9	11	145	187	117
SAUR1899 (SAR_RS09495)	8	8	8	2	2	2	2	2	N/A	41	41	5	8	53	2	15
SAUR1900 (SAR_RS09500)	119	119	119	96	96	96	96	96	119	74	74	8	22	128	74	74
SAUR1901 (SAR_RS09505)	19	19	19	69	69	69	69	69	19	59	59	11	10	88	12	12
SAUR1902 (SAR_RS09510)	144	144	N/A	115	267	115	115	115	144	90	90	8	N/A	146	185	116
SAUR1903 (SAR_RS09515)	158	158	N/A	123	123	123	123	123	158	N/A	N/A	9	9	162	227	124
SAUR1904 (SAR_RS09520)	11	11	11	30	30	30	30	30	11	18	18	152	7	2	2	18
SAUR1905 (SAR_RS09525)	144	144	144	N/A	110	110	110	110	144	73	73	19	50	N/A	202	112
SAUR1906 (SAR_RS09530)	95	95	95	N/A	72	72	72	72	95	N/A	N/A	8	8	2	2	2
SAUR1907 (SAR_RS09535)	5	5	5	2	2	2	2	2	5	9	9	5	5	9	9	9
SAUR1908 (SAR_RS09540)	N/A	N/A	N/A	80	80	80	80	80	N/A	30	30	N/A	32	44	N/A	81
SAUR1910 (SAR_RS09550)	69	69	69	91	91	91	91	91	N/A	88	88	63	66	62	119	103
SAUR1913 (SAR_RS09565)	82	82	82	64	64	64	64	64	82	25	25	2	6	245	104	58
SAUR1914 (SAR_RS09570)	15	15	15	28	28	28	28	28	N/A	19	19	15	7	44	59	11
SAUR1915 (SAR_RS09575)	55	55	55	44	44	44	44	44	55	19	19	5	6	2	72	16
SAUR1916 (SAR_RS09580)	61	61	61	53	53	53	53	53	61	44	44	25	11	62	78	15
SAUR1917 (SAR_RS09585)	136	136	136	183	183	183	113	183	136	92	92	10	21	137	185	361
SAUR1918 (SAR_RS09590)	202	202	202	93	93	93	93	93	202	N/A	N/A	9	N/A	117	151	N/A
SAUR1919 (SAR_RS09595)	254	254	254	2	2	2	2	2	361	78	78	N/A	N/A	2	156	99
SAUR1920 (SAR_RS09600)	150	150	150	124	124	124	124	124	507	97	97	7	7	453	202	N/A
SAUR1921 (SAR_RS09605)	282	282	N/A	65	65	65	65	65	282	37	37	166	158	74	8	66
SAUR1922 (SAR_RS09610)	4	4	4	4	4	4	4	4	N/A	13	13	8	10	66	81	13
SAUR1923 (SAR_RS09615)	3	3	3	3	3	3	3	3	3	2	2	3	8	15	3	2
SAUR1924 (SAR_RS09620)	128	128	128	106	106	106	106	106	N/A	74	74	346	114	N/A	167	N/A
SAUR1925 (SAR_RS09625)	181	181	181	331	139	139	139	139	181	N/A	N/A	28	51	183	240	140
SAUR1926 (SAR_RS09630)	94	94	94	41	41	41	41	41	94	52	52	6	40	41	120	41
SAUR1927 (SAR_RS09635)	105	266	105	34	34	34	34	34	N/A	29	29	96	98	43	63	N/A
SAUR1928 (SAR_RS09640)	501	N/A	501	N/A	N/A	N/A	N/A	N/A	501	68	68	35	23	350	160	N/A
SAUR2941	N/A	N/A	N/A	N/A	N/A	N/A	N/A	N/A	N/A	N/A	N/A	N/A	N/A	N/A	N/A	N/A
SAUR1973 (SAR_RS09865)	63	63	63	56	56	56	56	56	63	42	42	5	16	64	86	2
SAUR1975 (SAR_RS09875)	167	167	167	131	327	131	131	131	577	N/A	N/A	568	440	169	218	N/A
SAUR1976 (SAR_RS09880)	24	24	24	112	112	112	112	112	24	88	88	11	33	132	176	113
SAUR1977 (SAR_RS09885)	11	11	11	107	107	107	107	107	11	91	91	8	9	2	107	13
SAUR1978 (SAR_RS09890)	N/A	N/A	2	1	1	1	1	1	N/A	1	1	N/A	2	1	1	1
SAUR1979 (SAR_RS09895)	124	124	124	N/A	2	2	2	2	N/A	N/A	N/A	122	121	53	2	44
SAUR1980 (SAR_RS09900)	513	513	513	128	128	298	128	128	513	N/A	N/A	6	26	162	219	131
SAUR1981 (SAR_RS09905)	127	127	127	103	103	103	103	103	N/A	31	31	12	27	129	155	N/A
SAUR1982 (SAR_RS09910)	7	7	7	2	2	2	2	2	7	2	2	8	20	2	2	2
SAUR1983 (SAR_RS09915)	13	13	13	56	56	56	56	56	13	43	43	167	11	13	89	27
SAUR1984 (SAR_RS09920)	6	6	6	67	67	67	67	67	N/A	54	54	6	12	82	2	N/A
SAUR1985 (SAR_RS09925)	213	213	N/A	70	70	70	70	70	396	70	70	7	16	120	157	95
SAUR1986 (SAR_RS09930)	86	86	86	73	73	73	73	73	86	36	36	9	28	88	120	N/A
SAUR1987 (SAR_RS09935)	261	261	N/A	337	337	337	210	337	932	144	144	8	36	262	344	211
SAUR1988 (SAR_RS09940)	123	123	123	98	98	98	98	98	N/A	244	244	11	N/A	106	144	29
SAUR1990 (SAR_RS09950)	26	26	26	1	1	1	1	1	26	1	1	3	1	1	1	1
SAUR1991 (SAR_RS09955)	155	155	155	271	132	132	132	132	155	108	108	16	51	156	199	N/A
SAUR1992 (SAR_RS09960)	36	36	N/A	1	1	1	1	1	36	1	1	1	11	1	1	1
SAUR1993 (airR)	7	7	7	67	67	67	67	67	7	2	2	6	7	2	97	N/A
SAUR1994 (airS)	216	216	216	2	2	2	2	2	216	N/A	N/A	8	23	353	159	76
SAUR1995 (SAR_RS09975)	117	117	N/A	85	85	85	85	85	117	62	62	11	68	119	2	N/A
SAUR1996 (SAR_RS09980)	129	129	129	100	100	249	100	100	129	N/A	N/A	6	25	130	169	101
SAUR1997 (SAR_RS09985)	48	48	48	2	2	2	2	2	48	2	2	6	2	2	2	2
SAUR1999 (SAR_RS09995)	5	5	5	9	9	9	35	9	5	1	1	4	1	48	9	1
SAUR2000 (SAR_RS10000)	140	140	N/A	26	26	26	26	26	140	2	2	7	17	82	82	44
SAUR2001 (SAR_RS10005)	4	4	4	38	38	38	38	38	4	33	33	4	3	51	62	1
SAUR2002 (SAR_RS10010)	164	164	164	135	135	135	135	135	522	N/A	N/A	12	33	165	205	N/A
SAUR2003 (SAR_RS10015)	99	99	99	85	85	85	85	85	N/A	49	49	7	22	42	126	N/A
SAUR2004 (SAR_RS10020)	172	172	172	126	126	126	126	126	172	N/A	N/A	11	13	65	232	127
SAUR2005 (SAR_RS10025)	99	99	N/A	138	30	30	30	138	99	26	26	9	24	100	139	79
SAUR2036 (SAR_RS10180)	1	1	N/A	1	1	1	1	1	1	N/A	N/A	4	1	68	16	1
SAUR2037 (SAR_RS10185)	163	163	N/A	N/A	136	266	136	201	163	107	107	8	44	86	86	N/A
SAUR2038 (SAR_RS10190)	13	13	13	63	63	63	63	63	13	50	50	6	9	79	40	65
SAUR2039 (SAR_RS10195)	19	19	19	103	103	103	103	103	19	76	76	8	29	137	187	N/A
SAUR2040 (SAR_RS10200)	8	8	8	57	57	57	57	57	8	21	21	7	13	21	108	21
SAUR2042 (SAR_RS10210)	783	216	216	284	284	284	174	284	216	133	133	11	N/A	658	287	N/A
SAUR2043 (SAR_RS10215)	8	8	8	10	10	10	10	10	8	10	10	4	11	10	10	10
SAUR2044 (SAR_RS10220)	125	125	125	155	155	155	105	155	125	N/A	N/A	9	79	126	157	106
SAUR2045 (SAR_RS10225)	7	7	7	82	222	82	82	82	7	N/A	N/A	8	N/A	125	158	99
SAUR2046 (SAR_RS10230)	57	57	8	2	2	2	2	2	N/A	2	2	3	8	2	2	N/A
SAUR2047 (SAR_RS10235)	18	18	18	2	2	2	2	2	18	N/A	N/A	12	107	344	159	37
SAUR2048 (SAR_RS10240)	172	172	172	134	360	134	134	134	172	104	104	7	8	173	56	N/A
SAUR2049 (SAR_RS10245)	7	7	7	2	2	2	2	2	N/A	2	2	7	7	2	60	2
SAUR2050 (SAR_RS10250)	120	120	120	N/A	51	51	51	51	120	78	78	6	8	338	69	N/A
SAUR2051 (SAR_RS10255)	105	105	N/A	2	2	2	2	2	105	2	2	9	17	84	134	84
SAUR2052 (SAR_RS10260)	2	2	2	62	62	62	62	62	2	2	2	8	7	78	2	45
SAUR2053 (SAR_RS10265)	16	16	16	1	1	1	1	1	16	N/A	N/A	3	1	1	23	1
SAUR2054 (SAR_RS10270)	120	120	120	N/A	N/A	N/A	N/A	N/A	N/A	116	116	8	9	N/A	170	N/A
SAUR2055 (SAR_RS10275)	73	73	N/A	16	16	16	16	16	73	46	46	214	15	16	16	63
SAUR2056 (SAR_RS10280)	168	168	168	298	144	144	144	144	168	106	106	13	25	461	222	106
SAUR2057 (SAR_RS10285)	1	1	1	9	9	9	9	9	1	26	26	1	N/A	2	1	26
SAUR2058 (SAR_RS10290)	5	5	5	47	47	47	47	47	5	2	2	5	5	56	73	2
SAUR2059 (SAR_RS10295)	4	4	4	3	3	3	3	3	4	3	3	4	5	34	3	N/A
SAUR2060 (SAR_RS10300)	489	8	8	109	261	N/A	109	109	8	81	81	8	29	407	182	36
SAUR2061 (vraR)	5	5	5	2	2	2	2	2	N/A	2	2	5	6	67	7	2
SAUR2062 (vraS)	19	19	19	N/A	231	215	112	159	19	29	29	7	8	132	162	N/A
SAUR2063 (yvqF)	13	13	13	30	30	30	30	30	13	25	25	8	N/A	101	128	51
SAUR2064 (MW1827)	5	5	5	37	37	37	37	37	5	27	27	5	5	131	56	2
SAUR2065 (SAR_RS10325)	74	74	74	65	65	65	65	65	235	22	22	6	21	75	85	62
SAUR2067 (SAR_RS10335)	81	81	81	24	24	24	24	24	81	22	22	77	80	31	43	7
SAUR2068 (SAR_RS10340)	1	1	1	15	15	15	15	15	N/A	N/A	N/A	1	1	N/A	N/A	N/A
SAUR2069 (SAR_RS10345)	14	14	14	24	24	24	24	24	14	65	65	7	23	24	24	24
SAUR2070 (SAR_RS10350)	10	10	10	N/A	95	95	95	95	408	34	34	9	30	118	145	N/A
SAUR2071 (SAR_RS10355)	10	10	10	47	47	47	47	47	10	2	2	6	8	58	2	2
SAUR2072 (SAR_RS10360)	1	1	1	1	1	1	1	1	1	1	1	1	1	1	1	1
SAUR2073 (SAR_RS10365)	6	6	6	65	65	65	65	65	6	2	2	6	14	2	21	2
SAUR2074 (SAR_RS10370)	9	9	9	149	149	149	89	149	N/A	71	71	347	9	114	150	N/A
SAUR2075 (SAR_RS10375)	6	6	6	31	31	31	31	31	204	2	2	6	11	31	2	31
SAUR2076 (SAR_RS10380)	77	77	77	17	17	17	17	17	77	17	17	8	19	148	17	17
SAUR2077 (SAR_RS10385)	6	6	6	11	11	11	11	11	6	11	11	6	6	11	113	11
SAUR2078 (SAR_RS10390)	8	8	8	10	10	10	10	10	N/A	10	10	3	3	32	10	10
SAUR2079 (SAR_RS10395)	215	215	215	102	102	102	102	102	215	82	82	9	29	119	154	N/A
SAUR2080 (SAR_RS10400)	120	120	120	162	242	162	98	162	120	81	81	8	N/A	121	166	71
SAUR2081 (SAR_RS10405)	N/A	25	25	18	18	18	18	31	25	1	1	1	1	1	1	1
SAUR2082 (SAR_RS10410)	181	632	181	251	376	251	147	251	631	42	42	12	25	184	254	39
SAUR2083 (SAR_RS10415)	7	7	428	62	62	62	104	62	7	83	83	7	26	128	171	31
SAUR2084 (SAR_RS10420)	181	181	181	142	142	142	142	241	181	100	100	6	30	182	245	N/A
SAUR2085 (SAR_RS10425)	515	515	515	90	90	90	90	90	703	N/A	N/A	11	37	90	270	N/A
SAUR2086 (SAR_RS10430)	38	38	38	38	38	38	38	106	38	26	26	7	18	38	108	69
SAUR2087 (SAR_RS10435)	2	2	2	2	2	2	2	2	2	2	2	3	5	2	2	2
SAUR2088 (SAR_RS10440)	118	118	118	55	55	55	55	55	118	69	69	6	152	55	142	91
SAUR2089 (SAR_RS10445)	17	505	17	128	307	128	128	128	17	96	96	10	N/A	128	202	129
SAUR2091 (SAR_RS10455)	5	5	5	25	25	25	25	25	N/A	10	10	5	11	11	11	2
SAUR2092 (SAR_RS10460)	5	5	5	6	6	6	6	6	5	6	6	6	6	6	6	6
SAUR2093 (SAR_RS10465)	9	9	N/A	3	3	3	3	3	N/A	60	60	10	16	3	3	3
SAUR2094 (SAR_RS10470)	17	17	17	122	122	122	122	122	17	96	96	12	70	155	203	123
SAUR2095 (SAR_RS10475)	104	104	104	N/A	3	3	3	3	104	240	240	7	7	105	141	3
SAUR2096 (SAR_RS10480)	64	338	64	92	92	92	92	92	64	37	37	10	10	111	111	93
SAUR2098 (SAR_RS10490)	8	8	8	N/A	370	154	154	154	8	118	118	8	36	528	195	N/A
SAUR2099 (SAR_RS10495)	406	406	406	N/A	102	102	102	102	407	N/A	N/A	11	15	102	102	105
SAUR2100 (SAR_RS10500)	3	3	3	44	94	44	44	44	3	3	3	5	3	2	2	N/A
SAUR2101 (SAR_RS10505)	72	227	72	57	57	57	57	57	72	N/A	N/A	6	5	57	57	2
SAUR2102 (SAR_RS10510)	3	3	3	N/A	2	2	2	2	N/A	2	2	3	3	2	N/A	18
SAUR2103 (SAR_RS10515)	18	18	18	N/A	134	282	134	134	18	100	100	9	42	160	203	135
SAUR2104 (SAR_RS10520)	122	122	122	73	73	73	73	73	122	N/A	N/A	9	12	349	73	27
SAUR2105 (SAR_RS10525)	111	1	1	13	13	13	N/A	13	N/A	N/A	N/A	1	N/A	N/A	1	1
SAUR2106 (SAR_RS10530)	2	2	2	2	2	2	2	2	2	2	2	2	2	1	1	1
SAUR2107 (SAR_RS10535)	17	17	N/A	64	64	64	64	64	17	N/A	N/A	9	30	129	167	96
SAUR2108 (SAR_RS10540)	70	70	70	2	2	2	2	2	N/A	2	2	7	16	2	92	2
SAUR2109 (SAR_RS10545)	11	11	11	49	49	49	49	49	11	4	4	6	N/A	4	74	2
SAUR2110 (SAR_RS10550)	22	22	22	46	46	46	46	46	22	N/A	N/A	6	6	2	2	2
SAUR2111 (SAR_RS10555)	54	54	N/A	26	26	26	26	26	172	2	2	2	2	2	2	2
SAUR2112 (SAR_RS10560)	9	9	9	2	2	2	2	2	9	47	47	9	13	47	2	47
SAUR2113 (SAR_RS10565)	8	8	8	N/A	2	2	2	2	8	47	47	6	11	43	100	44
SAUR2114 (SAR_RS10570)	8	8	8	67	67	67	67	67	8	52	52	8	22	40	111	N/A
SAUR2115 (SAR_RS10575)	12	12	N/A	61	61	61	61	61	12	N/A	N/A	4	8	61	2	61
SAUR2116 (SAR_RS10580)	140	9	73	2	2	2	2	2	9	2	2	8	8	2	2	2
SAUR2117 (SAR_RS10585)	26	26	26	32	32	32	32	32	N/A	1	1	26	26	1	1	1
SAUR2118 (SAR_RS10590)	2	2	2	17	2	2	2	2	2	2	2	2	2	2	2	2
SAUR2119 (SAR_RS10595)	122	361	N/A	90	90	222	90	90	122	65	65	10	25	N/A	52	65
SAUR2122 (SAR_RS10610)	65	65	N/A	N/A	N/A	N/A	N/A	N/A	N/A	N/A	N/A	N/A	N/A	31	16	N/A
SAUR2190 (SAR_RS10950)	N/A	N/A	N/A	102	102	220	102	102	N/A	60	60	313	30	340	168	103
SAUR2191 (SAR_RS10955)	151	151	151	133	133	133	133	133	N/A	113	113	51	N/A	96	50	412
SAUR2192 (SAR_RS10960)	145	145	145	N/A	49	49	49	49	N/A	N/A	N/A	50	55	49	171	N/A
SAUR2193 (SAR_RS10965)	254	254	N/A	118	118	118	118	118	N/A	83	83	8	7	221	179	55
SAUR2194 (SAR_RS10970)	523	250	250	126	126	126	126	126	N/A	N/A	N/A	12	68	40	40	40
SAUR2199 (SAR_RS10995)	244	244	244	34	34	34	34	34	244	N/A	N/A	459	131	46	285	12
SAUR2200 (SAR_RS11000)	7	7	7	2	2	2	2	2	7	10	10	6	5	10	10	2
SAUR2201 (SAR_RS11005)	266	N/A	266	N/A	23	23	23	23	266	29	29	102	101	47	11	23
SAUR2202 (SAR_RS11010)	377	377	N/A	601	601	601	601	1130	N/A	N/A	N/A	57	104	840	843	836
SAUR2204 (SAR_RS11020)	8	8	8	97	97	97	97	97	8	74	74	281	18	113	147	98
SAUR2953 (agrB)	178	178	2	49	49	139	49	49	N/A	49	49	19	2	49	95	N/A
SAUR2954 (agrD1)	1	1	1	1	1	1	1	1	N/A	1	1	N/A	1	1	N/A	N/A
SAUR2958 (agrC)	853	854	853	N/A	951	951	951	951	N/A	951	951	N/A	194	N/A	388	N/A
SAUR2959 (agrA)	1	1	1	105	105	378	105	105	N/A	N/A	N/A	1	53	N/A	N/A	N/A
SAUR2210 (SAR_RS11050)	299	299	299	17	17	17	17	17	299	74	74	9	N/A	385	176	74
SAUR2211 (SAR_RS11055)	164	164	164	141	141	141	141	141	164	N/A	N/A	8	30	167	225	77
SAUR2212 (SAR_RS11060)	7	7	7	98	98	98	98	98	7	2	2	6	25	341	145	N/A
SAUR2213 (SAR_RS11065)	149	149	149	127	127	127	127	127	149	N/A	N/A	24	50	155	57	N/A
SAUR2214 (SAR_RS11070)	5	5	N/A	2	2	2	2	2	5	21	21	15	6	2	21	9
SAUR2215 (SAR_RS11075)	425	117	117	96	96	211	96	96	N/A	79	79	8	10	96	144	98
SAUR2216 (SAR_RS11080)	2	2	2	45	45	45	45	45	2	35	35	3	7	161	6	2
SAUR2217 (SAR_RS11085)	739	376	376	164	164	164	164	164	376	125	125	699	130	164	276	166
SAUR2220 (SAR_RS11100)	102	102	102	85	85	85	85	85	102	N/A	N/A	8	23	36	131	51
SAUR2221 (SAR_RS11105)	6	6	6	19	19	19	19	19	N/A	35	35	5	5	19	19	54
SAUR2222 (SAR_RS11110)	273	50	50	70	70	70	70	70	50	25	25	6	21	88	25	71
SAUR2223 (SAR_RS11115)	16	16	16	23	23	23	23	23	N/A	23	23	170	16	79	23	23
SAUR2224 (SAR_RS11120)	6	6	6	2	2	2	2	2	N/A	2	2	6	6	2	2	2
SAUR2225 (SAR_RS11125)	193	193	N/A	154	154	154	154	154	193	N/A	N/A	8	32	346	2	155
SAUR2226 (SAR_RS11130)	748	375	N/A	383	416	383	166	276	375	129	129	10	37	631	278	N/A
SAUR2227 (SAR_RS11135)	10	10	10	19	19	19	19	19	10	2	2	6	10	12	2	2
SAUR2228 (SAR_RS11140)	13	394	13	149	149	149	93	149	13	N/A	N/A	7	23	121	25	2
SAUR2229 (SAR_RS11145)	55	55	55	166	166	166	166	166	55	127	127	9	40	221	268	N/A
SAUR2230 (SAR_RS11150)	17	17	17	2	2	2	2	2	17	97	97	14	32	146	36	114
SAUR2231 (SAR_RS11155)	23	23	23	179	179	179	179	179	656	134	134	15	28	221	269	180
SAUR2232 (SAR_RS11160)	116	116	N/A	2	2	2	2	2	116	29	29	111	111	2	25	18
SAUR2233 (SAR_RS11165)	460	181	181	62	62	62	62	62	181	42	42	180	180	72	21	N/A
SAUR2240 (SAR_RS11200)	61	61	61	51	51	51	51	51	N/A	19	19	6	N/A	64	2	52
SAUR2241 (SAR_RS11205)	229	772	N/A	191	191	191	191	191	771	N/A	N/A	12	38	246	308	N/A
SAUR2242 (SAR_RS11210)	17	17	17	2	2	2	2	2	17	73	73	8	25	112	2	N/A
SAUR2243 (SAR_RS11215)	7	7	7	135	2	2	2	2	N/A	2	2	7	14	68	93	16
SAUR2244 (SAR_RS11220)	46	46	46	1	1	1	1	1	46	2	2	2	1	2	1	2
SAUR2245 (SAR_RS11225)	33	33	33	2	2	2	110	2	33	2	2	6	7	2	2	34
SAUR2246 (SAR_RS11230)	4	4	4	2	2	2	2	2	N/A	2	2	3	4	2	2	2
SAUR2247 (SAR_RS11235)	6	6	6	2	2	2	2	2	6	3	3	6	7	3	2	3
SAUR2248 (SAR_RS11240)	1	1	1	1	1	1	1	1	1	1	1	1	1	1	1	2
SAUR2249 (SAR_RS11245)	131	131	N/A	231	231	231	105	167	131	79	79	6	117	138	172	107
SAUR2250 (SAR_RS11250)	10	10	10	3	3	3	3	3	10	16	16	7	7	16	3	35
SAUR2251 (SAR_RS11255)	94	94	N/A	113	113	113	N/A	113	N/A	59	59	5	5	2	114	78
SAUR2252 (SAR_RS11260)	N/A	N/A	N/A	79	N/A	52	17	29	N/A	N/A	N/A	N/A	194	N/A	N/A	18
SAUR2253 (SAR_RS11265)	55	55	N/A	48	48	48	48	48	N/A	37	37	7	11	59	87	49
SAUR2254 (SAR_RS11270)	75	75	75	N/A	63	63	63	63	75	2	2	7	12	63	104	64
SAUR2255 (SAR_RS11275)	422	422	422	176	176	176	176	300	422	N/A	N/A	11	28	704	301	N/A
SAUR2256 (SAR_RS11280)	746	195	N/A	155	155	155	155	155	709	118	118	8	37	206	269	N/A
SAUR2257 (SAR_RS11285)	3	3	3	20	20	20	20	20	3	18	18	3	3	24	24	18
SAUR2258 (SA0067)	268	268	268	N/A	218	218	218	218	268	156	156	20	50	835	364	219
SAUR2259 (SA0066)	16	16	16	55	55	55	55	55	16	45	45	7	20	70	87	34
SAUR2260 (SAR_RS11300)	25	25	25	129	295	129	129	129	492	95	95	7	103	157	191	66
SAUR2261 (SAR_RS11305)	26	26	N/A	93	93	93	93	93	532	93	93	8	37	150	193	117
SAUR2262 (SAR_RS11310)	16	16	16	90	90	173	90	90	16	79	79	6	24	61	13	N/A
SAUR2263 (SAR_RS11315)	147	469	147	122	122	122	122	122	468	N/A	N/A	8	18	81	185	123
SAUR2265 (SAR_RS11325)	1	1	1	11	11	11	11	11	1	1	1	1	1	1	1	1
SAUR2266 (SAR_RS11330)	5	5	5	N/A	26	26	26	26	5	15	15	5	8	5	15	5
SAUR2267 (SAR_RS11335)	7	7	N/A	2	66	2	2	2	N/A	12	12	7	7	3	12	3
SAUR2268 (SAR_RS11340)	9	568	9	134	134	326	134	134	9	110	110	468	36	180	231	N/A
SAUR2269 (SAR_RS11345)	13	13	13	68	68	68	68	68	13	N/A	N/A	11	16	53	113	69
SAUR2271 (SAR_RS11355)	13	13	13	142	65	141	65	65	13	52	52	7	6	77	20	66
SAUR2272 (SAR_RS11360)	223	223	N/A	N/A	129	129	89	129	14	24	24	7	N/A	24	132	90
SAUR2273 (SAR_RS11365)	21	21	21	75	75	75	75	75	N/A	N/A	N/A	9	28	75	29	2
SAUR2274 (SAR_RS11370)	19	19	348	N/A	77	77	77	77	19	36	36	8	25	36	133	N/A
SAUR2275 (SAR_RS11375)	14	14	14	10	10	10	N/A	10	N/A	10	10	161	N/A	10	84	10
SAUR2276 (SAR_RS11380)	3	3	3	1	1	1	1	1	N/A	1	1	3	10	1	1	1
SAUR2277 (SAR_RS11385)	224	224	12	83	83	83	83	83	12	63	63	9	17	63	136	N/A
SAUR2278 (SAR_RS11390)	2	2	2	2	2	2	50	2	N/A	2	2	2	2	54	67	2
SAUR2279 (SAR_RS11395)	7	7	7	2	2	2	2	2	7	2	2	7	7	47	2	38
SAUR2280 (SAR_RS11400)	6	6	6	81	31	31	31	31	6	38	38	6	6	38	31	31
SAUR2281 (SAR_RS11405)	133	133	390	52	52	52	31	52	133	27	27	128	130	42	27	32
SAUR2282 (SAR_RS11410)	2	2	2	2	2	2	2	2	2	2	2	2	2	2	40	2
SAUR2284 (SAR_RS11420)	4	4	38	4	4	4	4	4	4	2	2	4	4	4	4	4
SAUR2285 (SAR_RS11425)	10	10	N/A	N/A	103	220	103	103	10	78	78	379	11	126	2	104
SAUR2286 (SAR_RS11430)	31	31	31	1	1	1	1	1	31	1	1	5	11	21	1	1
SAUR2287 (SAR_RS11435)	20	20	20	114	114	245	114	114	20	44	44	367	20	152	168	3
SAUR2288 (SAR_RS11440)	241	32	32	3	3	3	3	3	32	50	50	7	7	3	4	3
SAUR2289 (SAR_RS11445)	62	62	62	2	2	2	2	2	62	2	2	2	2	2	2	2
SAUR2290 (SAR_RS11450)	1	1	1	1	26	1	1	1	1	1	1	1	1	1	1	1
SAUR2291 (SAR_RS11455)	8	8	8	3	3	3	3	3	8	3	3	7	8	3	10	3
SAUR2292 (SAR_RS11460)	6	6	6	N/A	40	40	40	40	6	32	32	6	6	13	12	41
SAUR2293 (SAR_RS11465)	112	112	112	218	218	218	172	218	N/A	357	357	454	86	107	368	173
SAUR2294 (SAR_RS11470)	11	11	11	3	3	3	3	3	11	17	17	6	5	55	32	2
SAUR2295 (SAR_RS11475)	614	8	8	N/A	140	322	140	140	8	107	107	478	34	79	218	N/A
SAUR2296 (SAR_RS11480)	34	34	34	74	74	74	74	74	34	N/A	N/A	9	17	2	49	26
SAUR2297 (SAR_RS11485)	11	11	11	51	51	51	51	51	11	12	12	7	7	60	74	22
SAUR2298 (SAR_RS11490)	40	40	N/A	94	94	94	94	94	383	79	79	5	11	121	151	27
SAUR2299 (SAR_RS11495)	8	8	8	97	216	97	97	97	N/A	71	71	6	7	116	156	98
SAUR2300 (SAR_RS11500)	6	6	N/A	173	82	82	82	135	6	62	62	6	N/A	104	24	N/A
SAUR2301 (SAR_RS11505)	6	6	6	58	58	58	58	58	6	N/A	N/A	177	6	22	21	N/A
SAUR2302 (SAR_RS11510)	2	2	2	N/A	N/A	N/A	N/A	N/A	N/A	2	2	2	2	2	2	2
SAUR2303 (SAR_RS11515)	1	1	1	1	1	1	1	1	1	1	1	1	1	1	1	1
SAUR2304 (SAR_RS11520)	506	16	16	23	23	23	23	23	16	91	91	5	8	151	112	13
SAUR2305 (SAR_RS11525)	24	24	715	207	207	207	169	207	24	130	130	73	182	221	272	170
SAUR2306 (SAR_RS11530)	6	6	6	12	76	12	12	12	6	10	10	6	6	12	2	N/A
SAUR2307 (SAR_RS11535)	27	27	27	155	155	155	155	155	27	116	116	8	39	204	256	51
SAUR2308 (SAR_RS11540)	1	1	1	79	79	79	79	79	1	57	57	6	7	79	29	N/A
SAUR2309 (SAR_RS11545)	7	7	7	N/A	47	47	47	47	7	18	18	6	7	2	2	N/A
SAUR2310 (SAR_RS11550)	62	62	62	152	152	152	152	152	62	N/A	N/A	9	34	181	226	102
SAUR2311 (SAR_RS11555)	7	7	N/A	2	2	2	2	2	7	N/A	N/A	6	6	150	27	2
SAUR2312 (SAR_RS11560)	16	16	16	83	83	83	83	83	373	62	62	8	22	103	137	85
SAUR2313 (SAR_RS11565)	16	16	16	93	93	93	93	93	16	76	76	9	N/A	94	94	94
SAUR2314 (SAR_RS11570)	1	1	1	62	62	62	62	62	1	N/A	N/A	4	13	76	2	2
SAUR2315 (SAR_RS11575)	147	147	N/A	202	202	202	120	202	525	93	93	9	10	475	205	N/A
SAUR2316 (SAR_RS11580)	47	47	47	N/A	199	199	118	199	47	33	33	7	19	149	201	119
SAUR2317 (SAR_RS11585)	5	5	5	47	47	47	47	47	5	N/A	N/A	5	6	2	73	2
SAUR2318 (SAR_RS11590)	5	5	5	N/A	39	39	39	39	5	13	13	5	5	13	57	2
SAUR2319 (SAR_RS11595)	15	15	15	37	37	37	37	37	15	109	109	580	33	190	235	152

Locus (cg MLST)	Alternative designation	Position (in ED133)	Study Isolates							CC133			CC692				CC522					ST1347	Chinese CC133 MRSA isolates		CC5	ST71	CC182	CC707	CC1956
			Swan isolate 15V8707	Duck isolate 511525-22	Duck isolate V315	Duck isolate 511509-22	Duck isolate 511510-22	Whistling duck isolate IMT[illegible]	Duck isolate V482	CP001996.1, ED133	CP065353.1, AR39 [illegible]	LR134271.1, NCTC7988	CP042650.1, X22	UHAK01000002.1, NCTC9546	PubMLST ID 39948, 973N0056	CP129360, CKMM-401M	Goat isolate 17CS1042	PubMLST ID 24977	PubMLST ID 24358	PubMLST ID 11704	SAMEA1904171	PubMLST ID 39945, 61290	JAOAKU01, GDQ20D75A	JAFEOM01, GDQ20D75A	CP001781.1, ED98	SAMN03247575, MOK-099	CP070991, CBS2016-05	CP029751, SMITH	CP084892, WT19
SAUR2322 (SAR_RS11610)	deoD-L2	2226337	22	22	22	22	22	22	22	22	22	22	8	8	8	8	63	63	63	63	63	8	22	22	7	7	75	75	64
SAUR2323 (SAR_RS11615)	dpsA	2227168	2	2	2	2	2	2	2	2	2	2	6	6	6	6	2	2	2	2	2	6	3	3	6	7	2	119	2
SAUR2324 (SAR_RS11620)	DUF393	2227811	56	56	56	56	56	56	56	56	108	58	7	7	7	7	51	51	51	51	85	7	56	56	7	7	68	51	56
SAUR2325 (SAR_RS11625)	Q5HE59	2228682	89	89	N/A	89	89	N/A	N/A	89	89	89	140	534	140	140	184	113	113	113	184	140	89	89	9	28	141	187	N/A
SAUR2326 (SAR_RS11630)	Q7A0B3	2230729	1	1	N/A	N/A	N/A	N/A	1	1	1	1	4	4	4	4	1	1	1	1	1	4	1	1	4	4	1	1	2
SAUR2327 (SAR_RS11635)	manA1	2230990	95	95	N/A	N/A	N/A	N/A	N/A	95	95	95	13	13	13	13	71	71	71	71	71	13	N/A	N/A	11	23	83	168	N/A
SAUR2328 (SAR_RS11640)	yhfK1	2232363	30	30	N/A	30	30	N/A	30	30	30	30	84	84	84	84	92	92	92	92	92	84	N/A	N/A	48	27	112	134	N/A
SAUR2329 (SAR_RS11645)	N/A	2233180	3	3	3	3	3	3	3	3	3	3	34	34	34	34	3	3	3	3	3	34	3	3	3	11	2	3	3
SAUR2330 (SAR_RS11650)	czrB=zntA	2233502	82	82	82	82	82	82	82	82	82	82	89	89	89	89	99	99	99	99	99	89	82	82	6	23	117	159	59
SAUR2331 (SAR_RS11655)	Q9ZB00	2234748	85	85	85	N/A	85	85	85	85	85	85	N/A	132	132	N/A	85	85	85	85	174	N/A	N/A	N/A	9	9	85	53	110
SAUR2334 (SAR_RS11670)	Q6GES5	2239300	30	30	N/A	N/A	N/A	N/A	30	30	30	30	199	199	199	199	45	45	45	45	45	N/A	N/A	N/A	31	155	63	85	N/A
SAUR2335 (SAR_RS11675)	ylmA	2240225	98	98	98	98	98	98	98	98	98	103	N/A	30	30	N/A	24	24	57	24	44	N/A	98	98	76	76	24	46	N/A
SAUR2336 (SAR_RS11680)	glmS	2245558	87	87	87	87	87	87	87	87	87	87	N/A	152	152	N/A	N/A	120	120	120	120	152	87	87	501	22	154	10	121
SAUR2337 (SAR_RS11685)	mtlA	2248044	34	34	N/A	N/A	N/A	N/A	34	34	34	34	177	177	579	177	N/A	44	44	44	44	578	34	34	145	222	N/A	65	N/A
SAUR2338 (SAR_RS11690)	mtlR	2249617	157	157	157	N/A	N/A	157	N/A	157	406	157	244	244	244	244	316	316	316	197	316	244	157	157	6	35	756	322	N/A
SAUR2339 (SAR_RS11695)	mtlF	2251761	23	23	N/A	23	23	N/A	N/A	23	23	23	N/A	12	12	12	51	51	51	51	51	12	23	23	7	8	61	3	23
SAUR2340 (SAR_RS11700)	mtlD	2252195	92	92	92	92	92	92	92	92	92	92	37	37	37	37	122	122	122	122	122	454	92	92	51	N/A	406	182	33
SAUR2343 (SAR_RS11715)	glmM	2260013	90	90	N/A	N/A	N/A	90	N/A	87	90	87	18	18	18	18	113	113	249	113	113	444	90	90	11	38	135	175	114
SAUR2344 (SAR_RS11720)	ybbR=cdaR	N/A	68	68	68	N/A	N/A	68	68	N/A	68	68	8	8	8	8	93	93	93	93	93	8	68	68	8	8	62	138	94
SAUR2345 (SAR_RS11725)	cdaA=dacA	2262330	294	294	N/A	43	43	43	43	43	43	43	N/A	9	9	N/A	2	2	2	63	2	9	43	43	8	16	43	96	43
SAUR2346 (SAR_RS11730)	argI=rocF	2263328	59	59	59	59	59	59	N/A	59	168	59	9	9	9	9	N/A	75	75	75	75	N/A	N/A	N/A	297	17	27	27	2
SAUR2356 (SAR_RS11780)	N/A	2270160	24	24	24	24	24	24	N/A	24	24	24	N/A	N/A	N/A	N/A	36	36	36	36	36	N/A	24	24	N/A	N/A	39	N/A	N/A
SAUR2357 (SAR_RS11785)	salA	2270435	453	453	4	4	4	4	N/A	4	4	4	152	465	152	N/A	39	39	39	39	39	152	4	4	152	152	392	72	40
SAUR2358 (SAR_RS11790)	ycnB	2271647	33	33	N/A	33	33	N/A	33	33	33	33	188	188	188	188	126	44	126	44	89	N/A	N/A	N/A	178	180	62	93	46
SAUR2359 (SAR_RS11795)	sepA	2273706	35	35	35	35	35	35	35	35	35	62	N/A	36	36	N/A	35	35	35	35	35	N/A	35	35	36	37	35	35	35
SAUR2361 (SAR_RS11805)	hlIII	2275796	26	26	26	26	26	26	26	26	26	26	N/A	79	79	N/A	34	34	34	34	34	N/A	26	26	78	79	41	47	35
SAUR2362 (SAR_RS11810)	Q5HE34=urtF	2276498	90	90	N/A	N/A	N/A	N/A	N/A	90	90	90	10	10	10	10	114	114	114	114	114	424	90	90	9	10	381	80	N/A
SAUR2363 (SAR_RS11815)	yysG=ydiM	2277729	236	236	58	58	58	58	58	58	58	58	8	8	8	8	2	141	2	2	2	8	58	58	7	13	2	2	2
SAUR2364 (SAR_RS11820)	ynzG	2278421	16	16	16	16	16	16	16	16	16	16	6	6	6	6	27	27	27	27	27	6	16	16	6	7	2	2	16
SAUR2365 (SAR_RS11825)	Q5HE31	2278813	90	90	90	N/A	N/A	90	N/A	90	90	90	153	477	153	153	118	118	118	118	118	153	N/A	N/A	10	N/A	155	195	N/A
SAUR2366 (SAR_RS11830)	htsC=fecD	2280418	59	59	59	59	59	59	N/A	59	N/A	59	133	390	133	133	N/A	107	107	107	107	N/A	N/A	N/A	9	11	310	164	102
SAUR2367 (SAR_RS11835)	htsB	2281383	306	306	11	11	11	11	11	279	11	11	N/A	94	94	94	72	72	72	72	72	94	11	11	4	6	95	27	71
SAUR2368 (SAR_RS11840)	htsA	2282426	11	11	11	11	11	11	11	11	11	11	93	236	307	93	11	11	11	11	11	306	11	11	13	N/A	11	11	11
SAUR2369 (SAR_RS11845)	N/A	2283576	5	5	5	5	5	5	5	5	5	5	29	29	29	29	N/A	N/A	N/A	N/A	N/A	N/A	5	5	19	19	N/A	2	5
SAUR2370 (SAR_RS11850)	Q5HE27=sfnaC	2283802	86	86	N/A	86	86	86	86	86	86	86	40	40	40	40	170	105	105	105	170	40	86	86	7	7	139	172	N/A
SAUR2371 (SAR_RS11855)	rhbC1=sfaB	2284879	108	108	108	108	108	108	N/A	108	108	108	N/A	N/A	N/A	N/A	157	157	157	157	157	15	108	108	560	21	202	265	205
SAUR2372 (SAR_RS11860)	Q5HE25=sfaA	2286623	86	86	86	86	86	86	N/A	86	86	86	141	505	141	141	N/A	270	110	110	110	N/A	86	86	468	N/A	142	N/A	106
SAUR2373 (SAR_RS11865)	rhbC2=sfaD	2287918	141	141	141	N/A	N/A	141	141	134	141	134	N/A	871	826	N/A	318	318	318	188	318	825	141	141	11	41	412	324	190
SAUR2374 (SAR_RS11870)	asp23	2290056	41	41	41	41	41	41	41	41	41	41	40	40	40	40	2	2	2	2	2	165	41	41	5	7	2	75	2
SAUR2375 (SAR_RS11875)	DUF2273	2290629	3	3	3	3	3	3	3	3	3	3	5	5	5	5	28	28	28	28	28	5	3	3	5	5	3	3	3
SAUR2376 (SAR_RS11880)	Q5HE21	2290881	17	17	17	17	17	17	N/A	17	17	17	21	21	21	21	52	52	52	52	52	21	17	17	6	6	17	17	3
SAUR2377 (SAR_RS11885)	opuD2	2291592	180	N/A	180	180	180	180	180	114	180	114	N/A	186	186	N/A	N/A	151	N/A	151	151	186	180	180	11	35	N/A	265	152
SAUR2378 (SAR_RS11890)	Q5HE19	2293462	87	87	87	87	87	87	87	87	87	87	169	169	169	169	137	313	137	137	137	169	87	87	7	22	170	210	128
SAUR2379 (SAR_RS11895)	gorA	2294704	84	84	N/A	84	84	N/A	84	350	84	84	131	131	131	131	N/A	474	474	N/A	N/A	131	84	84	8	28	133	166	39
SAUR2380 (SAR_RS11900)	N/A	2295988	77	77	77	N/A	N/A	77	77	77	77	77	7	7	367	7	99	99	99	99	99	7	N/A	N/A	7	29	122	157	38
SAUR2381 (SAR_RS11905)	lacG	2297123	83	83	83	83	83	83	83	83	83	83	N/A	14	14	N/A	138	354	138	138	138	14	83	83	8	32	191	232	52
SAUR2382 (SAR_RS11910)	lacE	2298553	37	37	37	37	37	37	37	664	37	37	748	195	195	N/A	154	154	154	154	154	195	37	37	734	33	198	266	N/A
SAUR2383 (SAR_RS11915)	lacF	2300271	17	17	17	17	17	17	17	17	17	17	6	6	6	6	3	3	3	3	3	N/A	17	17	5	7	48	59	3
SAUR2384 (SAR_RS11920)	lacD	2300604	2	2	2	N/A	N/A	2	2	2	2	2	N/A	214	214	N/A	103	244	222	103	103	397	139	139	6	52	119	154	28
SAUR2385 (SAR_RS11925)	lacC	2301588	26	26	26	26	26	26	26	26	26	26	157	157	157	157	129	129	129	129	129	157	26	26	36	38	164	214	29
SAUR2386 (SAR_RS11930)	lacB	2302533	23	23	23	23	23	23	23	23	23	23	12	12	12	12	23	23	23	23	23	12	N/A	N/A	8	12	227	121	29
SAUR2387 (SAR_RS11935)	lacA	2303064	24	24	1	1	1	1	1	1	1	1	5	5	5	5	N/A	1	1	1	1	5	1	1	5	5	53	1	19
SAUR2388 (SAR_RS11940)	lacR	2303860	24	24	24	24	24	24	24	286	24	24	98	98	98	98	24	24	24	24	24	98	24	24	6	31	101	131	85
SAUR2389 (SAR_RS11945)	cobB	2304866	72	72	72	72	72	72	N/A	72	72	72	101	101	101	101	34	34	34	89	34	101	72	72	31	52	102	133	N/A
SAUR2394 (SAR_RS11970)	yyeN2	2306318	25	25	25	25	25	25	25	25	25	80	196	196	196	196	92	92	92	92	92	196	N/A	N/A	7	17	107	145	25
SAUR2395 (SAR_RS11975)	adhR	2307236	16	16	16	16	16	16	16	16	16	16	35	35	35	35	57	N/A	57	57	57	N/A	16	16	9	24	66	41	58
SAUR2396 (SAR_RS11980)	N/A	2307896	4	4	4	4	4	4	4	4	4	4	6	6	6	6	4	4	4	4	4	N/A	4	4	N/A	N/A	2	26	26
SAUR2400 (SAR_RS12000)	eap-L2=eapH-1	2312730	32	32	32	32	32	32	32	32	32	32	149	149	149	149	8	8	8	8	8	149	32	32	117	118	14	77	18
SAUR2401 (SAR_RS12005)	alsD-L1=budA	2313530	38	38	38	38	38	38	38	227	38	38	8	8	8	8	52	52	52	52	52	8	38	38	127	310	62	76	17
SAUR2402 (SAR_RS12010)	alsS	2314271	57	57	57	57	57	57	57	57	57	57	304	304	304	304	N/A	241	241	123	N/A	304	N/A	N/A	333	337	N/A	189	125
SAUR2406 (SAR_RS12030)	rpsI	2319435	1	1	1	1	1	1	1	1	1	1	2	2	2	2	1	1	1	1	1	2	1	1	2	2	38	53	2
SAUR2407 (SAR_RS12035)	rplM	2319847	33	33	33	33	33	33	33	33	33	33	2	N/A	128	2	2	2	2	2	2	2	33	33	5	2	1	1	2
SAUR2409 (SAR_RS12045)	ecfT	2321331	62	62	62	62	62	62	62	62	62	62	N/A	104	104	N/A	67	67	67	67	67	N/A	62	62	7	22	105	67	89
SAUR2410 (SAR_RS12050)	ecfA1	2322127	67	67	67	67	67	67	N/A	67	67	67	106	106	322	106	90	90	90	90	90	321	67	67	9	26	107	131	N/A
SAUR2411 (SAR_RS12055)	ecfA2	2322984	63	63	63	63	63	63	63	298	63	63	N/A	115	115	N/A	89	89	89	89	89	334	63	63	329	17	296	N/A	90
SAUR2414 (SAR_RS12070)	N/A	2324331	1	1	1	35	35	1	1	1	1	1	1	1	1	1	1	1	1	1	1	1	1	1	1	1	1	1	1
SAUR2415 (SAR_RS12075)	rpoA	2324716	2	2	2	2	2	2	2	2	2	2	3	3	3	3	2	2	2	2	2	3	2	2	3	45	2	2	2
SAUR2416 (SAR_RS12080)	N/A	2325735	1	1	1	1	1	1	1	1	1	1	1	1	1	1	1	1	1	1	1	1	1	1	6	1	1	1	1
SAUR2417 (SAR_RS12085)	N/A	2326148	20	20	20	20	20	20	20	20	20	20	1	1	1	1	24	24	24	24	24	1	20	20	1	1	1	1	1
SAUR2418 (SAR_RS12090)	N/A	2326536	1	1	1	1	1	1	1	1	1	1	1	1	1	1	1	1	1	1	1	1	1	1	1	1	1	1	1
SAUR2419 (SAR_RS12095)	N/A	2326681	1	1	1	1	1	1	1	1	1	1	1	1	1	1	1	24	1	1	1	1	1	1	1	1	1	1	1
SAUR2420 (SAR_RS12100)	adk	2327092	1	1	1	1	1	N/A	1	1	1	1	3	3	3	3	10	10	10	10	10	3	1	1	3	3	1	1	1
SAUR2421 (SAR_RS12105)	secY1	2327756	82	82	82	82	82	82	N/A	82	82	82	9	9	9	9	11	11	11	11	11	9	82	82	383	19	82	11	102
SAUR2422 (SAR_RS12110)	rplO	2329048	1	1	1	97	97	1	1	1	1	1	1	1	1	1	1	1	1	1	1	1	1	1	4	4	1	21	1
SAUR2423 (SAR_RS12115)	N/A	2329505	1	1	1	1	1	1	1	1	1	1	1	1	1	1	1	1	1	1	1	1	1	1	1	1	1	1	1
SAUR2424 (SAR_RS12120)	N/A	2329701	1	1	1	1	1	1	1	1	1	1	1	1	1	1	1	1	1	1	1	1	1	1	1	1	50	1	1
SAUR2425 (SAR_RS12125)	rplR	2330222	1	1	91	91	91	91	1	1	1	1	2	2	2	2	1	1	1	1	1	2	1	1	1	1	1	1	1
SAUR2426 (SAR_RS12130)	rplF	2330612	2	2	2	N/A	N/A	2	2	2	2	2	22	22	22	22	57	57	57	57	57	22	2	2	2	2	2	2	58
SAUR2427 (SAR_RS12135)	rpsH	2331173	1	1	1	1	1	1	N/A	1	1	1	3	3	3	3	39	39	39	23	39	3	1	1	3	3	1	1	1
SAUR2428 (SAR_RS12140)	N/A	2331603	1	1	1	1	1	1	1	1	1	1	1	1	1	1	1	1	1	1	1	1	1	1	1	1	1	1	1
SAUR2429 (SAR_RS12145)	N/A	2331811	1	1	1	7	7	1	1	1	1	1	135	1	1	1	N/A	39	39	39	39	1	1	1	1	1	1	1	1
SAUR2430 (SAR_RS12150)	rplX	2332377	1	1	1	1	1	1	1	1	1	1	2	2	2	2	1	1	1	1	1	2	1	1	2	2	1	1	1
SAUR2431 (SAR_RS12155)	rplN	2332730	8	8	8	8	8	8	8	8	8	8	5	5	5	5	N/A	1	89	1	69	5	8	8	5	5	49	8	1
SAUR2432 (SAR_RS12160)	rpsQ	2333130	2	2	2	2	2	2	2	2	2	2	2	2	2	2	15	15	15	15	15	2	2	2	2	2	2	2	2
SAUR2433 (SAR_RS12165)	N/A	2333417	1	1	1	1	1	1	1	1	1	1	1	1	1	1	1	1	1	1	1	N/A	1	1	1	1	1	1	1
SAUR2434 (SAR_RS12170)	N/A	2333616	1	1	1	1	1	1	1	1	1	1	19	19	19	19	1	1	1	1	1	19	1	1	124	1	1	1	1
SAUR2435 (SAR_RS12175)	rpsC	2334053	220	220	22	22	22	22	22	22	22	22	2	2	2	2	18	18	18	18	18	2	22	22	2	2	77	18	2
SAUR2436 (SAR_RS12180)	N/A	N/A	1	1	1	1	1	1	1	N/A	1	1	1	1	1	1	1	1	1	1	1	1	1	1	1	1	1	1	1
SAUR2437 (SAR_RS12185)	N/A	2335103	1	1	1	1	1	1	1	1	1	1	1	1	1	1	1	1	1	1	1	1	N/A	N/A	1	1	1	1	1
SAUR2438 (SAR_RS12190)	rplB	2335448	62	62	N/A	62	62	N/A	62	62	62	62	12	12	12	12	18	18	18	18	18	12	62	62	6	2	18	41	41
SAUR2439 (SAR_RS12195)	rplW	2336314	2	2	2	2	2	2	2	2	2	2	3	3	3	3	3	84	3	3	3	3	2	2	3	3	2	2	2
SAUR2440 (SAR_RS12200)	rplD	2336589	2	2	2	2	2	2	2	2	2	2	N/A	2	2	N/A	2	2	2	2	2	2	2	2	2	2	2	159	2
SAUR2441 (SAR_RS12205)	rplC	2337239	2	2	2	2	2	2	2	2	2	2	2	2	2	2	97	242	97	97	97	2	2	2	2	22	126	166	14
SAUR2442 (SAR_RS12210)	rpsJ	2337929	2	2	2	2	2	2	2	2	2	2	5	5	5	5	2	2	2	2	2	5	2	2	5	5	66	2	2
SAUR2443 (SAR_RS12215)	Q5HDV6	2338594	2	N/A	2	N/A	N/A	2	2	2	2	2	65	65	65	65	84	53	53	53	84	65	2	2	5	4	17	17	2
SAUR2444 (SAR_RS12220)	pbuG	2339178	20	20	20	20	20	20	N/A	20	20	20	184	184	184	184	331	220	220	143	220	574	20	20	9	116	20	233	N/A
SAUR2445 (SAR_RS12225)	topB	2340625	150	150	N/A	N/A	N/A	N/A	N/A	930	150	150	N/A	998	251	N/A	203	203	203	203	203	950	N/A	N/A	755	40	836	349	204
SAUR2446 (SAR_RS12230)	N/A	2342786	6	6	6	6	6	6	6	6	6	6	1	1	1	1	6	6	6	6	6	N/A	6	6	N/A	1	6	6	1
SAUR2447 (SAR_RS12235)	Q5HDV3	2342947	75	75	75	75	75	75	75	75	75	75	11	284	284	11	96	96	96	96	96	11	75	75	10	11	113	54	54
SAUR2448 (SAR_RS12240)	glcU	2344015	3	3	N/A	3	3	3	3	3	3	3	111	361	111	111	90	204	90	90	90	111	N/A	N/A	7	8	15	144	91
SAUR2449 (SAR_RS12245)	Q1Y7Y7	2345297	94	94	94	94	94	94	94	94	94	94	159	N/A	N/A	159	260	127	127	127	127	159	94	94	7	24	161	202	N/A
SAUR2450 (SAR_RS12250)	N/A	2346338	2	2	2	2	2	2	2	2	2	2	4	4	4	4	2	2	2	2	2	4	2	2	4	5	2	2	2
SAUR2451 (SAR_RS12255)	Q5HDU8=mspA	2346737	17	17	17	17	17	17	17	17	17	17	8	8	8	8	47	47	47	47	47	8	17	17	20	27	3	3	2
SAUR2452 (SAR_RS12260)	acrB	2347241	1223	1223	208	208	208	N/A	208	208	208	208	N/A	860	860	N/A	N/A	687	283	283	283	860	N/A	N/A	N/A	N/A	1062	444	N/A
SAUR2453 (SAR_RS12265)	fmhB=femX	2350525	109	109	N/A	N/A	N/A	N/A	109	109	N/A	109	477	167	167	477	N/A	136	136	136	136	167	109	109	8	24	168	212	138
SAUR2454 (SAR_RS12270)	N/A	2352179	3	3	3	3	3	3	3	3	3	3	12	12	12	12	2	2	2	2	2	N/A	3	3	6	3	42	51	2
SAUR2455 (SAR_RS12275)	Q5HDU5	2352422	25	25	25	25	25	25	25	25	25	25	6	6	6	6	147	94	94	94	147	6	25	25	6	13	62	149	95
SAUR2456 (SAR_RS12280)	Q5HDU4	2353349	50	50	50	50	50	50	50	50	50	50	64	64	64	64	17	17	17	17	17	N/A	50	50	5	11	66	81	50
SAUR2457 (SAR_RS12285)	ybfD	2353782	90	90	90	90	90	90	90	90	90	90	2	511	2	2	112	112	112	117	112	479	N/A	N/A	11	17	140	178	N/A
SAUR2458 (SAR_RS12290)	sarV	2355095	1	1	1	1	1	1	1	1	1	1	1	1	1	1	1	1	1	1	1	1	1	1	1	1	1	1	1
SAUR2459 (SAR_RS12295)	N/A	2355571	18	18	18	18	18	18	124	18	18	18	N/A	N/A	N/A	N/A	18	18	18	18	18	N/A	N/A	N/A	N/A	212	72	N/A	18
SAUR2461 (SAR_RS12305)	moaA	2356203	63	63	63	63	63	63	63	63	63	63	108	108	108	108	85	85	85	85	85	108	63	63	6	12	112	140	63
SAUR2462 (SAR_RS12310)	mobA	2357245	23	23	23	23	23	23	23	23	23	23	18	18	250	18	66	66	136	66	66	18	23	23	7	18	83	42	67
SAUR2463 (SAR_RS12315)	moaD	2357851	20	20	20	20	20	20	20	20	20	20	6	6	6	6	25	25	25	25	25	6	20	20	5	6	31	31	27
SAUR2464 (SAR_RS12320)	moaE	2358090	33	33	33	33	33	33	33	33	33	33	71	71	71	71	2	2	2	2	56	146	33	33	7	8	12	16	N/A
SAUR2465 (SAR_RS12325)	mobB	2358550	54	54	54	54	54	54	54	54	54	54	76	76	76	76	96	62	62	62	96	76	54	54	6	20	12	98	N/A
SAUR2466 (SAR_RS12330)	moeA	2359032	95	95	95	95	95	95	95	95	95	95	N/A	565	158	N/A	N/A	127	127	127	127	529	N/A	N/A	435	25	162	127	N/A
SAUR2467 (SAR_RS12335)	moaC	2360354	39	39	N/A	39	39	N/A	39	39	39	39	7	7	7	7	46	46	46	46	46	7	39	39	7	16	55	3	3
SAUR2468 (SAR_RS12340)	moaB	2360850	14	14	14	14	14	14	14	14	14	14	13	13	13	13	63	63	63	63	63	189	N/A	N/A	7	17	26	90	14
SAUR2469 (SAR_RS12345)	moeB	2361387	369	369	80	80	80	80	80	80	80	80	N/A	12	12	N/A	105	105	105	105	105	12	80	80	10	32	127	80	N/A
SAUR2470 (SAR_RS12350)	modC	2362560	50	50	50	50	50	50	50	50	50	50	7	7	7	7	67	67	67	67	67	7	50	50	6	7	78	38	38
SAUR2471 (SAR_RS12355)	modB	2363166	17	17	17	17	17	17	17	248	17	17	8	8	8	8	70	70	70	70	70	8	17	17	8	17	27	123	22
SAUR2472 (SAR_RS12360)	modA	2363851	51	51	51	N/A	N/A	51	51	51	51	51	87	87	87	87	46	46	46	46	46	281	51	51	8	N/A	89	118	73
SAUR2473 (SAR_RS12365)	fdhD	2364924	71	71	N/A	N/A	N/A	N/A	71	71	71	71	9	9	9	9	82	82	82	82	82	9	71	71	8	15	95	132	78
SAUR2474 (SAR_RS12370)	Q5HDS6	2365893	48	48	48	48	48	48	48	48	48	48	13	13	13	13	72	72	72	72	72	13	48	48	4	N/A	41	109	N/A
SAUR2475 (SAR_RS12375)	bioY	2366735	47	47	47	47	47	47	47	47	47	47	7	7	7	7	42	42	42	42	42	7	N/A	N/A	6	7	42	37	62
SAUR2476 (SAR_RS12380)	rihB	2367410	443	443	80	80	80	80	80	80	80	80	140	140	140	140	107	107	244	107	107	140	80	80	8	19	385	107	108
SAUR2477 (SAR_RS12385)	fhuD2	2368751	85	85	85	N/A	N/A	85	85	85	85	85	9	9	9	9	110	110	110	110	110	9	85	85	8	N/A	361	71	15
SAUR2478 (SAR_RS12390)	caiA	2369874	97	97	97	97	97	97	97	97	97	97	N/A	21	21	N/A	133	133	133	133	133	21	97	97	8	8	166	202	N/A
SAUR2479 (SAR_RS12395)	utp=yut	2371475	40	40	40	40	40	40	40	40	N/A	40	N/A	221	221	N/A	N/A	56	102	56	56	221	40	40	137	138	70	82	13
SAUR2480 (SAR_RS12400)	N/A	2372640	10	N/A	10	10	10	10	10	125	10	10	9	9	9	9	8	8	8	8	8	9	10	10	1	1	8	8	1
SAUR2481 (SAR_RS12405)	ureB	2372956	12	12	12	12	12	12	12	12	12	N/A	8	8	8	8	32	32	32	32	32	8	12	12	6	7	11	11	48
SAUR2482 (SAR_RS12410)	ureC	2373363	121	121	121	N/A	N/A	121	N/A	121	121	121	494	494	494	494	161	161	161	161	161	N/A	N/A	N/A	10	38	N/A	268	162
SAUR2483 (SAR_RS12415)	ureE	2375091	32	32	32	32	32	32	32	32	32	32	N/A	6	6	N/A	43	43	43	43	43	N/A	32	32	6	6	12	12	20
SAUR2484 (SAR_RS12420)	ureF	2375536	58	58	58	58	58	58	58	58	58	59	85	85	85	85	66	66	66	66	66	N/A	58	58	7	8	86	86	67
SAUR2485 (SAR_RS12425)	ureG	2376238	26	26	26	26	26	26	N/A	26	26	26	6	277	6	6	61	61	61	61	61	6	N/A	N/A	6	7	38	94	62
SAUR2486 (SAR_RS12430)	ureD	2376852	50	50	50	50	50	50	50	50	50	50	10	10	10	10	88	88	88	88	88	10	50	50	8	9	100	100	89
SAUR2487 (SAR_RS12435)	N/A	2377877	1	1	1	1	1	1	1	1	1	1	1	1	1	1	1	1	1	1	1	1	1	1	1	1	1	1	1
SAUR2488 (SAR_RS12440)	N/A	2378522	2	2	2	2	2	N/A	2	2	2	2	3	3	37	3	2	2	2	2	2	N/A	2	2	3	3	N/A	N/A	N/A
SAUR2489 (SAR_RS12445)	rsr	2378652	2	2	2	2	2	N/A	2	2	2	2	2	2	2	2	2	2	2	2	2	2	2	2	6	7	3	3	40
SAUR2490 (SAR_RS12450)	iraD	2379042	2	2	2	2	2	N/A	2	2	2	2	13	13	13	13	2	2	2	2	2	N/A	2	2	7	26	97	97	N/A
SAUR2491 (SAR_RS12455)	iraC	2379811	N/A	N/A	151	N/A	151	N/A	N/A	151	151	151	N/A	838	405	N/A	190	190	190	190	190	405	N/A	N/A	9	36	230	304	N/A
SAUR2492 (SAR_RS12460)	ssaA2	2382376	2	2	2	N/A	N/A	2	2	2	197	2	116	116	116	116	N/A	224	2	2	2	N/A	N/A	N/A	9	27	54	149	95
SAUR2494 (SAR_RS12470)	phaC	2383613	115	115	115	N/A	N/A	115	115	115	N/A	115	N/A	11	11	N/A	151	151	349	151	151	11	N/A	N/A	10	13	189	251	N/A
SAUR2495 (SAR_RS12475)	Q5HDQ7	2385108	2	2	2	2	2	2	2	2	2	2	349	73	73	349	14	14	14	14	14	73	2	2	7	20	2	14	N/A
SAUR2496 (SAR_RS12480)	Q2FEJ1	2386438	195	195	48	48	48	48	48	48	48	48	8	202	8	8	62	62	62	62	62	8	48	48	6	12	45	84	45
SAUR2497 (SAR_RS12485)	ssaA3	2387097	16	16	16	16	16	16	N/A	16	16	16	N/A	21	21	21	2	2	2	2	2	21	16	16	6	6	16	137	34
SAUR2499 (SAR_RS12495)	yycT	2387958	394	394	N/A	N/A	N/A	N/A	N/A	66	66	66	8	8	8	8	154	154	154	97	154	371	66	66	27	8	113	N/A	N/A
SAUR2500 (SAR_RS12500)	Q8NV79	2389003	462	462	87	87	87	87	87	87	87	87	7	7	7	7	113	113	113	113	113	7	87	87	441	20	148	2	N/A
SAUR2502 (SAR_RS12510)	sagA=lytA	2390963	51	51	N/A	N/A	N/A	N/A	N/A	51	51	51	100	100	100	100	51	51	51	51	51	322	51	51	10	11	280	51	N/A
SAUR2503 (SAR_RS12515)	Q5HDQ1	2391761	1	1	1	1	1	1	1	1	1	1	1	1	1	1	1	1	1	1	1	1	1	1	1	1	6	1	1
SAUR2504 (SAR_RS12520)	yrhD	2392289	2	2	2	2	2	2	2	2	2	2	95	95	95	95	7	7	60	7	7	95	2	2	79	79	39	7	29
SAUR2505 (SAR_RS12525)	N/A	2392762	83	83	83	83	83	83	83	1068	83	83	581	1145	581	581	120	120	120	120	120	581	N/A	N/A	988	451	974	206	121
SAUR2506 (SAR_RS12530)	lytR2=lcpC	2396319	66	66	66	66	66	66	66	66	66	66	N/A	109	109	N/A	91	91	91	91	91	109	66	66	8	20	60	91	15
SAUR2507 (SAR_RS12535)	suhB2	2397399	72	72	72	72	72	72	72	72	72	72	11	345	11	11	72	N/A	72	72	72	11	72	72	10	26	111	72	72
SAUR2508 (SAR_RS12540)	Q5HDP6	2398581	263	263	45	45	45	45	45	45	45	45	N/A	6	6	6	45	45	45	63	45	6	45	45	208	22	83	83	45
SAUR2509 (SAR_RS12545)	Q5HDP4=sdpB	2399298	49	49	49	49	49	49	N/A	49	49	49	38	38	38	38	67	162	67	67	67	38	49	49	6	6	87	112	42
SAUR2510 (SAR_RS12550)	Q5HDP3	2400084	6	6	6	N/A	6	6	6	6	6	6	50	50	50	50	40	40	40	40	40	50	6	6	6	7	54	51	41
SAUR2511 (SAR_RS12555)	N/A	2400857	261	261	N/A	N/A	N/A	N/A	53	53	53	53	7	7	7	7	71	71	71	71	71	7	53	53	6	7	96	92	20
SAUR2512 (SAR_RS12560)	proY	2402113	78	78	78	78	78	78	N/A	78	78	78	35	35	35	35	159	98	98	98	159	35	78	78	8	26	122	161	2
SAUR2513 (SAR_RS12565)	Q5HDP0	2403773	19	19	19	19	19	19	19	19	19	19	55	55	55	55	74	74	74	44	74	55	19	19	6	17	19	19	19
SAUR2514 (SAR_RS12570)	Q5HDN9	2404126	1	1	1	1	1	1	1	1	1	1	1	1	1	1	1	1	1	1	1	1	1	1	2	1	1	1	1
SAUR2515 (SAR_RS12575)	N/A	2404407	17	17	17	17	17	17	17	17	11	17	2	2	2	2	11	11	11	11	11	2	11	11	2	2	2	2	2
SAUR2516 (SAR_RS12580)	glpW	2404609	53	53	N/A	N/A	N/A	N/A	53	53	155	53	8	8	8	8	109	109	109	69	109	8	53	53	7	8	6	6	70
SAUR2517 (SAR_RS12585)	yocS	2405319	66	66	N/A	N/A	N/A	N/A	N/A	66	66	66	N/A	105	105	105	84	84	84	84	84	N/A	66	66	8	16	106	138	N/A
SAUR2518 (SAR_RS12590)	Q5HDN5	2406504	36	36	N/A	N/A	N/A	N/A	36	36	36	36	N/A	3	3	N/A	36	36	36	36	36	3	N/A	N/A	3	6	61	77	36
SAUR2519 (SAR_RS12595)	glyC	N/A	N/A	546	N/A	N/A	N/A	N/A	92	N/A	92	92	N/A	30	30	30	N/A	140	140	140	140	30	92	92	29	N/A	294	239	80
SAUR2520 (SAR_RS12600)	glyR	2409034	39	39	39	39	39	39	39	39	39	39	62	62	62	62	29	29	29	29	29	62	N/A	N/A	5	11	63	63	27
SAUR2521 (SAR_RS12605)	Q5HDN2	2409856	2	2	2	2	2	2	2	2	2	2	57	57	57	57	N/A	48	48	48	48	57	2	2	7	7	49	49	2
SAUR2523 (SAR_RS12615)	Q5HDN0	2412068	107	107	107	107	107	107	107	107	107	107	N/A	72	72	72	26	26	26	26	26	N/A	107	107	71	72	182	186	113
SAUR2524 (SAR_RS12620)	yhxD=yghA	2413127	91	91	91	91	91	91	91	91	91	91	N/A	8	8	N/A	91	91	91	91	91	8	91	91	11	16	15	15	109
SAUR2525 (SAR_RS12625)	Q5HDM8=yxeP	2414119	2	2	N/A	2	2	61	2	2	2	2	2	2	2	2	2	2	2	2	2	N/A	2	2	6	13	11	11	2
SAUR2527 (SAR_RS12635)	hutI	2415518	27	27	27	27	27	27	N/A	27	27	27	141	141	141	141	N/A	117	117	117	117	141	27	27	327	N/A	426	185	118
SAUR2528 (SAR_RS12640)	hutU	2416756	111	111	N/A	N/A	N/A	N/A	N/A	617	111	236	N/A	8	8	760	146	368	146	146	247	8	111	111	7	N/A	N/A	134	N/A
SAUR2529 (SAR_RS12645)	hutR	2418577	23	23	N/A	N/A	N/A	N/A	23	67	23	67	N/A	117	117	117	98	98	98	98	98	117	23	23	6	N/A	N/A	138	N/A
SAUR2531 (SAR_RS12655)	hutG	2420318	73	N/A	73	73	73	73	73	73	73	73	N/A	395	104	N/A	94	199	182	94	94	104	N/A	N/A	282	6	105	105	N/A
SAUR2532 (SAR_RS12660)	lyrA=sdpC	2421543	106	106	106	N/A	N/A	106	106	106	249	106	25	25	25	25	269	118	118	118	118	25	106	106	7	N/A	150	192	120
SAUR2533 (SAR_RS12665)	rpiA	2422865	24	24	24	24	24	24	N/A	24	24	24	8	8	8	8	77	77	77	77	77	8	24	24	8	21	94	120	77
SAUR2534 (SAR_RS12670)	yflK=yiiM	2423722	62	62	62	62	62	62	N/A	62	62	62	325	237	237	325	2	2	2	2	2	325	62	62	8	13	99	99	83
SAUR2535 (SAR_RS12675)	N/A	2424399	2	2	2	2	2	2	2	2	2	2	N/A	N/A	N/A	N/A	2	2	2	21	2	N/A	2	2	5	N/A	2	2	2
SAUR2536 (SAR_RS12680)	galM	2424611	2	2	N/A	N/A	N/A	N/A	2	2	2	2	N/A	114	114	N/A	19	19	19	19	19	429	2	2	16	N/A	2	2	2
SAUR2537 (SAR_RS12685)	ypfA	2425660	2	2	2	2	2	2	2	2	2	2	2	2	2	2	2	2	2	2	2	2	2	2	2	2	2	2	38
SAUR2538 (SAR_RS12690)	yhaP	2426204	12	12	12	12	12	12	12	12	12	12	64	541	64	64	122	122	122	122	122	64	12	12	6	26	12	12	12
SAUR2539 (SAR_RS12695)	yhaQ	2427429	32	32	N/A	N/A	N/A	N/A	32	82	32	82	120	120	120	120	102	102	102	102	102	N/A	32	32	7	26	121	147	75
SAUR2540 (SAR_RS12700)	Q5HDL3=yhaI	2428559	111	111	N/A	111	111	111	111	111	111	111	N/A	23	23	N/A	N/A	N/A	N/A	N/A	N/A	N/A	111	111	157	11	292	138	107
SAUR2541 (SAR_RS12705)	yxlJ	2429605	44	44	44	44	44	44	44	44	44	44	16	16	327	16	76	76	76	76	76	16	44	44	7	18	96	128	77
SAUR2542 (SAR_RS12710)	gltS	2430550	81	81	81	81	81	81	N/A	77	81	77	N/A	27	27	N/A	N/A	113	113	113	113	27	N/A	N/A	7	16	144	144	20
SAUR2543 (SAR_RS12715)	fni=idi2	2431806	2	2	2	2	2	2	2	2	191	2	30	30	30	30	89	89	89	89	89	365	191	191	8	11	116	152	2
SAUR2544 (SAR_RS12720)	corA	2432886	69	69	69	69	69	69	69	69	69	69	N/A	104	N/A	N/A	84	84	84	84	84	29	N/A	N/A	7	21	105	105	85
SAUR2545 (SAR_RS12725)	Q5HDK8	2433930	24	24	24	24	24	24	24	43	24	43	19	19	19	19	N/A	2	2	2	2	19	24	24	6	15	2	2	54
SAUR2546 (SAR_RS12730)	Q5HDK7	2434418	34	34	34	34	34	34	34	34	34	34	15	15	15	15	46	46	46	46	46	15	34	34	6	7	58	58	2
SAUR2547 (SAR_RS12735)	N/A	2434945	68	68	68	68	68	68	N/A	68	68	68	N/A	33	33	N/A	86	198	183	86	86	33	68	68	7	15	106	142	87
SAUR2548 (SAR_RS12740)	Q5HDK5	2436073	24	24	24	24	24	24	24	50	24	50	12	12	12	12	39	163	39	67	39	261	24	24	6	18	86	24	2
SAUR2549 (SAR_RS12745)	semB	2436958	52	52	52	52	52	52	52	52	52	52	N/A	254	254	N/A	160	160	160	68	111	254	52	52	237	301	86	86	69
SAUR2550 (SAR_RS12750)	semA	2438902	30	30	30	30	30	30	30	30	30	30	122	122	258	122	28	28	86	28	28	122	30	30	117	119	49	49	12
SAUR2551 (SAR_RS12755)	Q5HDK2	2439670	2	2	2	2	2	2	2	2	2	2	119	119	119	119	2	2	2	2	2	119	2	2	6	121	2	2	2
SAUR2553 (SAR_RS12765)	tcaB	2440451	496	496	90	90	90	90	90	90	90	90	141	N/A	141	141	112	265	112	112	112	141	N/A	N/A	6	33	430	90	90
SAUR2554 (SAR_RS12770)	N/A	2441769	3	3	3	3	3	3	3	3	3	3	6	6	6	6	3	3	3	3	3	N/A	3	3	3	4	3	3	3
SAUR2555 (SAR_RS12775)	tcaA	2441928	57	57	N/A	N/A	N/A	N/A	N/A	528	57	115	N/A	24	24	N/A	139	139	139	139	139	24	57	57	10	N/A	169	130	130
SAUR2556 (SAR_RS12780)	N/A	2443220	1	1	1	1	N/A	1	1	1	1	1	5	87	87	5	2	2	2	2	2	N/A	1	1	5	5	36	2	2
SAUR2557 (SAR_RS12785)	tcaR	2443550	17	17	17	17	17	17	17	17	17	17	1	1	1	1	1	1	1	1	64	1	17	17	3	1	1	66	1
SAUR2558 (SAR_RS12790)	Q5HDJ7	2444200	94	94	94	94	94	94	94	94	N/A	94	43	43	43	43	94	94	94	94	94	43	94	94	8	33	109	109	112
SAUR2559 (SAR_RS12795)	hrtA	2445399	46	46	46	46	46	46	46	52	46	52	8	8	8	8	68	171	68	68	68	8	46	46	8	16	87	119	15
SAUR2560 (SAR_RS12800)	hrtB	2446064	83	83	N/A	N/A	N/A	N/A	83	83	83	83	38	323	323	38	103	103	103	103	103	38	83	83	10	86	137	175	104
SAUR2561 (hssR)	N/A	2447255	24	24	24	24	24	24	24	24	24	24	153	153	153	153	73	73	73	73	73	N/A	24	24	7	11	24	24	74
SAUR2562 (hssS)	N/A	2447922	N/A	N/A	N/A	34	34	34	N/A	34	34	34	N/A	524	524	154	N/A	131	131	131	131	41	34	34	8	31	155	200	N/A
SAUR2563 (SAR_RS12815)	lytT	2449460	2	2	2	2	2	2	N/A	2	2	2	11	11	11	11	23	23	23	23	23	190	2	2	7	8	39	39	23
SAUR2564 (SAR_RS12820)	Q5HDJ1	2449897	2	2	2	2	2	2	2	2	2	2	89	89	89	89	37	37	37	37	37	89	2	2	87	89	43	2	34
SAUR2568 (SAR_RS12840)	mqo	2453161	97	97	97	97	97	97	97	97	97	97	N/A	296	296	N/A	128	128	128	128	128	296	97	97	8	30	160	217	N/A
SAUR2569 (SAR_RS12845)	lctP-locus2	2455020	92	92	92	92	92	92	92	92	92	92	58	58	58	58	92	92	92	92	92	58	92	92	10	36	2	2	131
SAUR2570 (SAR_RS12850)	N/A	2456920	122	122	122	N/A	122	122	122	122	122	122	197	197	197	197	158	158	158	158	158	197	122	122	9	54	199	199	N/A
SAUR2571 (SAR_RS12855)	Q5HDI7	2458761	21	21	N/A	21	21	21	21	21	21	21	7	164	164	7	54	54	54	54	54	N/A	21	21	176	19	69	69	2
SAUR2572 (SAR_RS12860)	paiA	2459704	261	261	24	24	24	24	N/A	24	122	24	8	8	8	8	27	27	27	27	27	8	24	24	8	8	83	83	61
SAUR2573 (SAR_RS12865)	N/A	2460364	30	30	30	30	30	30	30	30	30	30	N/A	20	20	20	112	112	112	112	112	20	30	30	9	N/A	135	172	N/A
SAUR2574 (SAR_RS12870)	Q5HDI4	2461456	10	10	N/A	N/A	N/A	N/A	10	10	N/A	40	141	141	141	141	46	46	46	46	46	141	10	10	13	140	55	55	47
SAUR2575 (SAR_RS12875)	iruO	2461893	N/A	388	N/A	N/A	N/A	N/A	N/A	28	28	28	18	406	18	18	153	153	153	94	153	372	28	28	8	15	117	117	14
SAUR2576 (SAR_RS12880)	N/A	2462938	1	1	1	N/A	1	1	1	1	1	1	6	6	6	6	2	2	2	2	2	N/A	1	1	10	4	1	28	2
SAUR2577 (SAR_RS12885)	Q5HDI2	2463159	1	1	1	1	1	N/A	1	1	1	1	1	1	1	1	1	1	1	1	1	1	1	1	1	1	1	62	33
SAUR2578 (SAR_RS12890)	N/A	2463642	2	2	2	2	2	2	2	2	2	2	5	5	5	5	14	14	14	14	14	N/A	2	2	5	5	2	2	2
SAUR2579 (SAR_RS12895)	pip	2463796	85	N/A	85	N/A	N/A	85	85	85	85	85	19	19	19	19	117	317	117	117	117	N/A	N/A	N/A	9	28	160	206	N/A
SAUR2580 (SAR_RS12900)	Q5HDH9=tetR21	2465234	52	N/A	52	52	52	52	52	52	52	52	17	17	17	17	68	68	68	68	68	17	52	52	7	N/A	37	37	N/A
SAUR2581 (SAR_RS12905)	cobI	2465903	2	2	2	N/A	N/A	2	2	2	2	2	N/A	7	7	N/A	2	2	2	2	2	7	2	2	174	7	67	67	2
SAUR2582 (SAR_RS12910)	scrA	2466933	94	94	94	94	94	94	N/A	94	94	94	N/A	90	90	N/A	131	131	299	131	131	N	N/A	N/A	12	102	155	209	N/A
SAUR2583 (SAR_RS12915)	DUF1722	2468558	47	47	47	47	47	47	N/A	45	47	47	66	66	66	66	58	58	58	58	58	N/A	47	47	6	16	67	90	41
SAUR2584 (SAR_RS12920)	rsp=araC	2470429	868	868	148	148	148	N/A	N/A	148	148	148	N/A	628	628	N/A	190	190	190	190	190	N/A	N/A	N/A	777	47	763	324	191
SAUR2585 (SAR_RS12925)	ydaG	2472605	2	2	2	2	2	2	2	2	2	2	N/A	199	N/A	199	65	33	33	33	33	213	2	2	86	87	2	53	2
SAUR2586 (SAR_RS12930)	Q5HDH2	2473307	2	2	2	2	2	2	2	2	2	2	97	97	97	97	2	2	2	2	2	N/A	N/A	N/A	79	81	2	29	29
SAUR2588 (SAR_RS12940)	gltT	2473918	62	62	62	62	62	62	62	62	62	62	N/A	6	6	6	30	30	30	30	30	N/A	62	62	6	6	118	56	92
SAUR2589 (SAR_RS12945)	DUF3139	2475377	N/A	2	2	N/A	2	2	N/A	2	2	2	71	71	133	71	36	36	36	36	36	71	2	2	6	7	7	17	2
SAUR2590 (SAR_RS12950)	sarZ	2476140	2	2	N/A	N/A	N/A	N/A	2	2	2	2	29	29	343	29	2	208	2	2	2	29	2	2	3	N/A	2	2	2
SAUR2591 (SAR_RS12955)	N/A	2476793	199	N/A	23	23	23	23	23	23	23	23	71	71	71	71	63	63	63	63	63	71	23	23	186	8	72	41	64
SAUR2592 (SAR_RS12960)	narK	2477337	95	95	95	95	95	95	95	95	95	95	151	151	151	151	48	48	255	48	48	151	N/A	N/A	9	37	154	191	127
SAUR2595 (nreC)	N/A	2480767	2	2	2	2	2	2	2	2	2	2	6	6	6	6	2	117	2	2	2	6	2	2	6	10	61	2	61
SAUR2596 (nreB)	N/A	2481443	49	49	49	49	49	49	49	49	49	62	82	82	82	82	49	49	49	49	49	82	49	49	8	25	84	49	N/A
SAUR2597 (nreA)	N/A	2482501	36	36	36	36	36	36	36	36	36	N/A	5	5	5	5	8	109	8	8	8	5	36	36	5	8	59	8	N/A
SAUR2598 (narI)	N/A	2482973	52	52	52	52	52	52	52	52	N/A	52	N/A	77	77	N/A	2	2	2	2	2	77	52	52	207	18	78	98	64
SAUR2599 (narJ)	N/A	2483643	2	2	2	2	2	2	2	2	2	2	65	65	65	65	2	2	2	2	2	N/A	2	2	7	17	56	82	56
SAUR2600 (narH)	N/A	2484211	12	12	12	N/A	N/A	12	N/A	12	12	12	N/A	121	121	121	27	248	27	27	27	N/A	12	12	9	22	N/A	164	89
SAUR2601 (narG)	N/A	2485760	173	173	N/A	N/A	N/A	N/A	173	173	173	N/A	1054	1054	1054	1054	N/A	241	582	241	241	N/A	173	173	9	49	329	428	N/A
SAUR2602 (SAR_RS13010)	nasF=cobA	2489758	116	116	116	116	116	116	116	116	116	116	N/A	149	149	149	135	135	135	135	172	N/A	116	116	47	N/A	151	159	137
SAUR2603 (SAR_RS13015)	nasE=nirD	2490726	2	2	2	2	2	2	2	2	2	2	7	7	7	7	47	47	47	47	47	N/A	2	2	107	7	47	34	2
SAUR2604 (SAR_RS13020)	nasD=nirB	2491044	140	140	140	140	140	140	140	140	140	147	210	655	655	210	173	406	377	173	173	210	N/A	N/A	13	51	212	278	175
SAUR2606 (SAR_RS13030)	Q5HDF4	2494473	3	3	3	3	3	3	3	3	3	3	153	153	153	N/A	27	27	27	27	27	153	3	3	74	74	34	3	28
SAUR2607 (SAR_RS13035)	focA-L2=nirC	2495529	31	31	31	31	31	31	31	31	31	31	167	167	167	167	38	N/A	38	38	38	N/A	31	31	115	116	45	61	39
SAUR2609 (SAR_RS13045)	Q5HDF2	2496825	37	37	37	37	37	37	37	37	37	37	18	18	217	18	6	6	6	6	6	18	37	37	39	18	61	98	61
SAUR2610 (SAR_RS13050)	zinT=adcA	2497478	N/A	669	137	N/A	N/A	137	137	142	137	137	23	692	655	22	379	170	170	170	170	N/A	142	142	545	38	205	272	171
SAUR2614 (SAR_RS13070)	dsbA	2500110	241	241	21	21	21	21	N/A	21	21	21	69	69	69	69	59	59	59	59	59	69	21	21	8	9	70	59	38
SAUR2615 (SAR_RS13075)	N/A	2500728	1	1	N/A	N/A	N/A	N/A	1	1	1	1	1	1	1	1	1	1	1	1	1	1	1	1	1	1	1	1	14
SAUR2616 (SAR_RS13080)	fmhA	2501345	49	49	N/A	49	49	N/A	49	49	49	49	77	77	77	77	63	63	63	63	63	77	49	49	208	210	417	63	34
SAUR2617 (SAR_RS13085)	tcyC	2502688	35	35	35	35	35	35	35	35	35	35	3	3	3	3	2	2	2	2	2	3	N/A	N/A	6	3	70	2	N/A
SAUR2618 (SAR_RS13090)	tcyB	2503416	248	248	11	11	11	11	11	11	11	11	7	7	7	7	75	75	75	75	75	N/A	11	11	7	14	89	10	N/A
SAUR2619 (SAR_RS13095)	tcyA	2504116	N/A	62	62	62	62	62	62	62	62	62	94	94	263	94	168	72	72	72	72	94	62	62	8	22	95	120	37
SAUR2620 (SAR_RS13100)	mdeA	2505016	89	89	89	89	89	89	N/A	89	N/A	89	299	299	299	299	120	120	120	120	216	N/A	89	89	6	24	165	219	78
SAUR2621 (SAR_RS13105)	N/A	2506629	7	7	7	7	7	7	7	7	7	7	2	2	2	2	7	7	7	7	7	2	7	7	5	2	2	25	2
SAUR2622 (SAR_RS13110)	N/A	2506887	46	46	46	46	46	46	46	46	46	46	68	68	208	68	13	13	13	13	13	68	46	46	7	9	70	13	57
SAUR2623 (SAR_RS13115)	cdf	2507900	66	66	N/A	66	66	N/A	66	66	66	66	104	104	104	104	196	84	84	84	84	104	66	66	8	9	58	138	85
SAUR2624 (SAR_RS13120)	N/A	2509578	2	2	2	2	2	2	2	2	2	2	N/A	3	3	N/A	N/A	N/A	N/A	N/A	N/A	N/A	2	2	3	3	N/A	2	2
SAUR2625 (SAR_RS13125)	sbi	N/A	N/A	N/A	N/A	N/A	N/A	N/A	N/A	N/A	N/A	N/A	114	N/A	N/A	N/A	211	91	211	91	139	N/A	N/A	N/A	3	17	N/A	N/A	N/A
SAUR2627 (SAR_RS13135)	hlgA	2511622	75	75	75	N/A	75	75	75	356	N/A	75	N/A	15	15	15	103	103	103	103	103	15	N/A	N/A	13	15	131	103	N/A
SAUR2628 (SAR_RS13140)	lukS=hlgC	2513119	77	77	77	77	77	77	N/A	77	77	77	12	12	N/A	12	98	98	98	98	98	12	N/A	N/A	8	30	116	155	N/A
SAUR2630 (SAR_RS13150)	bioX	2515097	50	50	50	50	50	50	50	50	50	50	13	13	13	13	N/A	N/A	N/A	N/A	N/A	N/A	50	50	6	29	89	112	75
SAUR2631 (SAR_RS13155)	bioW	2515575	61	61	61	61	61	61	61	61	61	61	12	12	12	12	158	54	54	54	54	12	61	61	6	20	250	114	74
SAUR2632 (SAR_RS13160)	bioF	2516278	91	91	91	91	91	91	91	91	91	91	135	135	135	135	111	111	237	111	111	135	91	91	7	468	143	178	112
SAUR2633 (SAR_RS13165)	bioB	2517371	66	66	66	66	66	66	66	66	66	66	101	101	101	N/A	87	87	87	87	87	N/A	N/A	N/A	7	12	102	79	88
SAUR2634 (SAR_RS13170)	bioA	2518380	90	90	90	90	90	90	90	90	90	90	142	142	479	142	181	181	181	117	181	142	285	285	7	38	146	184	118
SAUR2635 (SAR_RS13175)	bioD	2519716	67	67	67	67	67	67	67	67	67	67	N/A	96	96	N/A	87	87	87	87	87	N/A	67	67	6	24	97	122	N/A
SAUR2636 (SAR_RS13180)	N/A	2520457	23	23	23	23	N/A	23	23	23	23	23	4	4	4	4	10	10	10	10	10	N/A	23	23	N/A	4	63	45	N/A
SAUR2637 (SAR_RS13185)	Q5HDC6-L1	2520624	1	1	1	1	1	1	1	1	1	1	29	29	29	29	1	1	1	1	1	N/A	1	1	4	6	1	59	24
SAUR2640 (SAR_RS13200)	Q5HDC6-L2	2524946	1	1	1	1	1	1	1	1	1	1	7	7	7	N/A	1	1	1	1	1	N/A	1	1	N/A	5	9	1	1
SAUR2642 (SAR_RS13210)	gtrA=gtxA	2525320	2	2	N/A	N/A	N/A	N/A	2	34	2	34	25	25	25	25	18	18	18	18	18	25	2	2	25	25	18	18	18
SAUR2643 (SAR_RS13215)	glxK1	2525974	80	80	N/A	N/A	N/A	N/A	80	80	80	80	N/A	142	N/A	N/A	114	114	114	114	193	477	80	80	9	N/A	144	195	N/A
SAUR2644 (SAR_RS13220)	Q5HDC0	2527176	65	65	N/A	N/A	N/A	N/A	65	65	65	65	81	206	206	81	2	2	2	2	2	81	65	65	6	N/A	21	21	76
SAUR2645 (SAR_RS13225)	bcr	2528021	87	87	87	87	87	87	87	87	87	87	N/A	132	132	N/A	106	106	106	106	106	132	87	87	9	19	108	175	108
SAUR2646 (SAR_RS13230)	aarP	2529355	24	24	24	24	24	24	24	24	24	24	N/A	8	8	N/A	3	3	3	3	3	8	24	24	6	13	197	24	24
SAUR2647 (SAR_RS13235)	Q5HDB7	2529941	62	62	N/A	N/A	N/A	N/A	N/A	311	160	62	94	94	94	94	79	79	79	79	79	94	62	62	9	19	80	80	80
SAUR2648 (SAR_RS13240)	Q5HDB6	2530660	89	89	89	N/A	N/A	89	89	89	89	89	N/A	N/A	116	N/A	N/A	55	55	55	55	116	89	89	7	18	34	149	34
SAUR2649 (SAR_RS13245)	aapA3	2531752	109	109	N/A	109	109	109	109	109	109	109	165	165	165	165	143	143	143	143	143	N/A	N/A	N/A	11	40	176	223	43
SAUR2650 (SAR_RS13250)	nhaK2	2533299	93	93	93	93	93	93	N/A	93	286	93	306	306	306	306	321	120	120	120	200	306	286	286	12	42	154	203	121
SAUR2651 (SAR_RS13255)	N/A	2535457	1	1	1	1	1	1	1	1	1	1	N/A	N/A	N/A	N/A	1	1	1	1	1	N/A	1	1	1	1	1	N/A	1

Locus (cg MLST)	Alternative designation	Position (in ED133)	Study Isolates							CC133			CC692				CC522					ST1347	Chinese CC133 MRSA isolates		CC5	ST71	CC182	CC707	CC1956
			Swan isolate 15V8707	Duck isolate 511525-22	Duck isolate V315	Duck isolate 511509-22	Duck isolate 511510-22	Whistling duck isolate	Duck isolate V482	CP001996.1, ED133	CP065353.1, AR39	LR134271.1, NCTC7988	CP042650.1, X22	UHAK01000002.1, NCTC9546	PubMLST ID 39948, 973N0056	CP129360, CKMM-401M	Goat isolate 17CS1042	PubMLST ID 24977	PubMLST ID 24358	PubMLST ID 11704	SAMEA1904171	PubMLST ID 39945, 61290	JAOAKU01, GDQ20D75A	JAFEOM01, GDQ20D75A	CP001781.1, ED98	SAMN03247575, MOK-099	CP070991, CBS2016-05	CP029751, SMITH	CP084892, WT19
SAUR2652 (SAR_RS13260)	ydaO	2535518	102	102	102	102	102	102	102	102	102	102	177	177	177	177	137	137	322	137	137	177	102	102	7	34	293	234	N/A
SAUR2654 (SAR_RS13270)	flp=fmtA	2537579	46	46	46	46	46	46	46	46	46	46	242	242	486	242	512	512	512	512	512	242	46	46	380	232	74	92	N/A
SAUR2655 (SAR_RS13275)	rfbD	2539185	79	79	79	79	79	79	79	79	79	79	9	9	373	9	155	96	96	96	155	N/A	79	79	33	21	118	159	14
SAUR2657 (SAR_RS13285)	panE1	2540360	71	71	71	71	71	71	71	71	71	71	13	13	13	13	2	2	203	2	2	13	N/A	N/A	6	24	108	140	54
SAUR2658 (SAR_RS13290)	mmr	2541521	472	472	89	89	89	89	89	89	89	89	131	131	131	131	107	107	245	107	107	131	N/A	N/A	9	9	132	89	108
SAUR2659 (SAR_RS13295)	opuCD	2543214	13	13	N/A	13	13	N/A	13	13	152	13	8	8	8	8	69	69	69	69	69	8	N/A	N/A	7	7	90	119	70
SAUR2660 (SAR_RS13300)	opuCC	2543909	67	67	67	67	67	67	67	67	67	71	12	12	12	12	139	187	139	89	139	12	67	67	8	8	67	143	61
SAUR2661 (SAR_RS13305)	opuCB	2544867	14	14	14	N/A	N/A	14	14	14	14	14	13	13	13	13	N/A	14	14	14	14	13	N/A	N/A	33	33	14	14	49
SAUR2662 (SAR_RS13310)	opuCA	2545499	487	487	93	93	93	93	93	93	248	93	153	514	153	153	93	93	272	93	196	153	93	93	10	N/A	93	93	N/A
SAUR2667 (SAR_RS13335)	pnbA	2550118	105	105	105	105	105	105	N/A	105	105	243	611	611	613	611	136	136	136	136	136	611	105	105	8	36	177	244	137
SAUR2668 (SAR_RS13340)	pbuE	2551532	86	86	86	86	86	86	86	86	86	99	508	96	96	N/A	N/A	120	120	120	120	96	86	86	8	73	120	120	N/A
SAUR2669 (SAR_RS13345)	yikA=ybbM=fetB	2553114	24	24	24	24	24	24	24	24	24	24	174	174	174	174	20	20	20	20	20	174	24	24	118	120	58	20	46
SAUR2670 (SAR_RS13350)	yikB	2553883	14	14	14	N/A	N/A	14	14	14	14	14	N/A	135	135	135	47	47	47	47	47	N/A	14	14	131	133	59	74	48
SAUR2671 (SAR_RS13355)	pepA2	2554890	448	448	N/A	N/A	N/A	N/A	28	82	28	82	N/A	317	317	N/A	N/A	116	116	116	116	435	28	28	32	123	140	28	117
SAUR2672 (SAR_RS13360)	Q5HD92	N/A	175	175	16	16	16	16	16	N/A	16	16	8	8	8	8	2	2	2	2	2	8	16	16	6	8	20	76	2
SAUR2673 (SAR_RS13365)	verD	2557829	117	117	117	N/A	N/A	117	N/A	615	317	117	622	623	623	622	244	244	350	151	244	100	317	317	10	N/A	185	247	N/A
SAUR2674 (SAR_RS13370)	Q5HD90	2559600	2	2	362	2	2	362	N/A	47	2	N/A	N/A	94	94	N/A	74	171	74	74	74	N/A	2	2	31	38	20	20	N/A
SAUR2675 (SAR_RS13375)	norD	2560573	90	90	90	90	90	90	90	90	90	90	212	443	399	212	171	171	171	114	171	212	90	90	357	32	138	90	N/A
SAUR2676 (SAR_RS13380)	cntF	2561778	63	63	63	N/A	N/A	63	63	63	63	63	N/A	86	86	N/A	73	73	73	73	73	N/A	N/A	N/A	8	25	63	63	15
SAUR2677 (SAR_RS13385)	cntD	2562520	289	289	2	2	2	N/A	2	2	2	34	172	172	172	172	38	38	38	38	38	172	2	2	146	13	50	2	39
SAUR2678 (SAR_RS13390)	cntC	2563332	76	76	N/A	N/A	N/A	N/A	76	76	2	76	101	101	101	101	N/A	93	93	93	93	101	2	2	9	26	102	2	94
SAUR2679 (SAR_RS13395)	cntB	2564198	3	3	3	3	3	3	3	3	3	3	31	31	N/A	31	3	3	3	3	3	31	3	3	11	25	108	3	3
SAUR2680 (SAR_RS13400)	cntA	2565146	N/A	N/A	113	113	113	113	113	113	113	113	35	35	575	35	148	148	148	148	148	574	N/A	N/A	565	N/A	194	242	150
SAUR2681 (SAR_RS13405)	N/A	2566887	77	77	77	77	77	77	77	77	77	77	N/A	N/A	N/A	N/A	125	125	N/A	125	125	N/A	N/A	N/A	11	32	153	203	126
SAUR2682 (SAR_RS13410)	fmrO=cntL	2568181	N/A	N/A	N/A	72	72	N/A	N/A	72	72	72	9	244	244	9	12	12	12	12	12	9	72	72	8	20	103	88	88
SAUR2683 (SAR_RS13415)	dapF=cntK	2569010	100	100	N/A	N/A	N/A	N/A	100	77	100	77	9	9	9	9	15	15	15	15	15	308	100	100	8	23	103	2	87
SAUR2685 (SAR_RS13425)	yxbG	2571398	27	27	27	27	27	27	97	27	27	27	110	110	110	110	34	83	34	34	34	N/A	27	27	102	106	47	57	35
SAUR2686 (SAR_RS13430)	Q5HD78	2572452	78	78	N/A	N/A	N/A	N/A	N/A	78	78	78	584	N/A	584	584	N/A	122	305	122	122	584	N/A	N/A	37	N/A	N/A	214	N/A
SAUR2687 (SAR_RS13435)	aphD	2574102	22	22	22	22	22	22	22	22	22	22	N/A	16	16	N/A	22	22	22	22	22	16	22	22	7	14	68	68	22
SAUR2694 (SAR_RS13470)	bdhA	2575650	34	34	34	34	34	34	34	269	34	34	22	22	22	22	88	134	88	N/A	88	22	34	34	175	15	68	88	2
SAUR2695 (SAR_RS13475)	iraB	2576645	10	10	10	10	10	10	10	10	10	10	66	66	66	66	4	4	4	4	4	N/A	10	10	29	32	4	4	2
SAUR2696 (SAR_RS13480)	N/A	N/A	N/A	N/A	N/A	N/A	N/A	N/A	N/A	N/A	N/A	N/A	N/A	3	3	N/A	N/A	N/A	N/A	N/A	N/A	N/A	N/A	N/A	3	7	N/A	N/A	2
SAUR2697 (SAR_RS13485)	N/A	2577186	15	15	15	15	15	15	15	15	15	15	15	15	15	15	23	23	23	23	23	15	15	15	1	5	23	23	2
SAUR2704 (SAR_RS13520)	pgcA	2585372	91	91	91	91	91	91	91	91	253	N/A	148	148	148	148	125	125	125	125	125	N/A	N/A	N/A	4	18	151	198	N/A
SAUR2705 (SAR_RS13525)	Q5HD60	2587293	1	1	N/A	N/A	N/A	N/A	N/A	1	1	1	1	1	1	1	36	36	36	36	36	1	1	1	4	3	1	1	3
SAUR2706 (SAR_RS13530)	Q5HD59	2587703	24	24	24	24	24	24	24	24	24	24	N/A	182	N/A	182	N/A	N/A	N/A	N/A	N/A	182	24	24	13	17	132	132	84
SAUR2707 (SAR_RS13535)	gtaB=galU	2589701	54	54	54	54	54	54	54	54	54	54	88	88	88	88	160	73	73	73	73	88	54	54	N/A	12	90	41	75
SAUR2719 (SAR_RS13595)	dedA	2610276	64	64	64	64	64	64	64	64	64	64	162	162	162	162	165	176	81	81	81	162	N/A	N/A	7	19	101	127	2
SAUR2723 (SAR_RS13615)	stp	2613040	61	61	N/A	61	61	61	61	61	61	61	150	150	150	150	N/A	77	77	77	77	150	61	61	35	24	27	27	78
SAUR2724 (SAR_RS13620)	smp	2613737	22	22	N/A	N/A	N/A	N/A	N/A	22	22	56	N/A	13	13	13	62	62	62	62	62	N/A	22	22	5	13	22	100	67
SAUR2725 (SAR_RS13625)	fbp	2615012	20	20	N/A	20	20	20	N/A	20	20	20	N/A	225	225	N/A	67	67	67	67	67	N/A	20	20	224	270	86	113	39
SAUR2726 (SAR_RS13630)	Q1Y5K2	2617318	25	25	25	25	25	25	N/A	25	227	25	12	12	12	12	113	113	113	113	113	12	25	25	374	12	142	184	N/A
SAUR2727 (SAR_RS13635)	Q1Y5K1	2618502	18	18	N/A	N/A	N/A	N/A	18	18	18	18	25	25	25	25	71	71	71	71	71	25	18	18	202	25	90	120	72
SAUR2728 (SAR_RS13640)	N/A	2619117	302	302	9	9	9	9	N/A	9	9	9	108	108	108	108	86	86	86	86	86	N/A	N/A	N/A	N/A	15	110	9	N/A
SAUR2729 (SAR_RS13645)	mhqR	2620163	2	2	2	2	2	2	2	193	2	2	10	10	10	10	57	57	57	57	57	10	2	2	2	10	67	2	2
SAUR2730 (SAR_RS13650)	Q5HD32	2620823	2	2	2	2	2	2	2	2	2	2	29	29	29	29	2	2	2	2	2	29	2	2	15	21	1	2	1
SAUR2731 (SAR_RS13655)	catE	2621282	422	422	82	82	82	82	82	82	82	82	9	442	9	9	100	100	100	100	100	9	N/A	N/A	7	9	104	71	37
SAUR2732 (SAR_RS13660)	frp	2622202	116	116	116	116	116	116	116	469	116	116	11	11	11	11	152	152	152	152	152	N/A	116	116	6	11	177	208	75
SAUR2733 (SAR_RS13665)	ldhD	2623143	51	51	51	51	51	51	51	362	51	51	120	120	120	120	96	96	96	96	96	N/A	N/A	N/A	8	10	121	171	3
SAUR2736 (SAR_RS13680)	ywtE	2624382	50	50	50	50	50	50	50	50	50	50	92	92	92	92	77	77	77	77	127	N/A	50	50	6	18	79	130	11
SAUR2737 (SAR_RS13685)	srtA	2627037	46	46	46	46	46	46	46	46	46	46	80	80	80	80	46	46	46	46	46	80	46	46	6	7	17	17	17
SAUR2738 (SAR_RS13690)	ywnH=yncA	2627851	N/A	42	42	42	42	42	42	42	42	42	63	63	63	63	7	7	7	7	7	63	42	42	136	5	N/A	85	7
SAUR2739 (SAR_RS13695)	N/A	2628491	1	1	1	1	1	1	1	1	1	1	1	1	1	1	1	1	1	1	1	1	1	1	1	1	1	22	1
SAUR2740 (SAR_RS13700)	N/A	N/A	N/A	N/A	N/A	N/A	N/A	N/A	N/A	N/A	N/A	N/A	17	17	17	17	9	9	9	9	9	N/A	N/A	N/A	3	19	9	N/A	N/A
SAUR2741 (SAR_RS13705)	sdaA	2628840	25	25	25	25	25	25	25	25	25	25	47	47	47	47	163	2	2	74	110	47	25	25	9	N/A	2	2	37
SAUR2742 (SAR_RS13710)	sdaB	2629753	2	2	2	2	2	2	2	2	2	2	82	82	82	82	2	2	2	2	2	82	N/A	N/A	7	2	2	108	2
SAUR2743 (SAR_RS13715)	ptsEIIC	2630436	28	28	28	N/A	N/A	28	28	28	28	28	106	106	106	106	N/A	86	86	86	86	106	28	28	8	11	86	28	28
SAUR2744 (SAR_RS13720)	Q5HD18	2632028	2	2	2	2	2	2	2	2	2	2	4	4	4	4	2	2	2	2	2	N/A	2	2	5	5	2	2	2
SAUR2745 (SAR_RS13725)	N/A	2632387	22	22	N/A	N/A	N/A	N/A	22	22	22	22	6	6	6	6	77	77	77	77	77	373	22	22	8	21	101	138	2
SAUR2746 (SAR_RS13730)	aes	2633429	16	16	N/A	16	16	N/A	16	16	16	16	N/A	117	117	N/A	35	35	35	35	35	408	N/A	N/A	10	74	119	157	16
SAUR2748 (SAR_RS13740)	Q5HD14	2634916	22	22	22	22	22	22	22	22	22	22	N/A	10	10	10	60	60	60	60	60	N/A	22	22	9	10	62	88	15
SAUR2749 (SAR_RS13745)	glcB=ptsG	2635706	78	78	N/A	N/A	N/A	N/A	78	78	78	78	N/A	334	334	N/A	131	131	131	131	131	334	N/A	N/A	555	19	194	244	133
SAUR2750 (SAR_RS13750)	cidC=pox	2638072	70	70	N/A	N/A	N/A	N/A	70	73	70	73	348	348	348	348	92	92	92	92	92	348	70	70	298	299	99	9	N/A
SAUR2751 (SAR_RS13755)	cidB	2639851	49	49	49	49	49	49	49	49	49	49	7	7	7	7	58	58	58	58	58	7	49	49	7	7	60	2	59
SAUR2752 (SAR_RS13760)	cidA	2640533	14	14	14	N/A	N/A	14	14	14	14	14	7	7	157	7	44	44	83	44	44	N/A	14	14	7	6	14	14	N/A
SAUR2753 (SAR_RS13765)	cidR	2641141	28	28	28	28	28	28	N/A	28	28	28	89	89	89	89	76	173	76	76	119	89	28	28	8	14	77	122	77
SAUR2754 (SAR_RS13770)	N/A	2642186	2	2	2	2	2	N/A	2	2	2	2	2	2	2	2	2	2	2	2	2	67	2	2	1	1	2	28	1
SAUR2755 (SAR_RS13775)	ssaA4	2642794	2	2	2	2	2	2	2	36	2	36	9	9	9	9	12	12	12	12	12	9	2	2	2	9	52	69	2
SAUR2756 (SAR_RS13780)	mvaA	2643366	13	N/A	13	13	13	13	N/A	13	13	13	225	225	225	225	76	76	76	47	76	225	13	13	145	147	64	78	N/A
SAUR2757 (SAR_RS13785)	mvaS	2644901	14	14	14	14	14	14	14	217	14	14	112	112	112	112	14	14	14	14	14	112	14	14	6	12	93	146	92
SAUR2758 (SAR_RS13790)	adaB	2646238	16	16	16	16	16	16	16	16	16	16	82	82	245	82	70	70	70	70	70	82	16	16	22	14	73	98	71
SAUR2759 (SAR_RS13795)	clpL	N/A	N/A	N/A	N/A	N/A	N/A	N/A	N/A	N/A	N/A	N/A	N/A	240	240	240	N/A	N/A	N/A	N/A	N/A	N/A	N/A	N/A	61	64	N/A	N/A	N/A
SAUR2764 (SAR_RS13820)	Q8NUR3=farR	2654562	154	154	154	N/A	154	154	154	154	154	154	106	106	106	106	150	150	150	158	150	N/A	154	154	2	49	163	150	145
SAUR2765 (SAR_RS13825)	Q5HCZ7	2655187	2	2	2	2	2	2	N/A	2	2	2	6	6	6	6	N/A	N/A	N/A	N/A	N/A	N/A	2	2	12	6	22	16	22
SAUR2766 (SAR_RS13830)	rocA=pruA	2655466	92	92	92	92	92	92	92	92	92	92	153	153	538	153	120	120	N/A	120	120	153	92	92	531	28	263	203	121
SAUR2767 (SAR_RS13835)	maa	2657201	23	23	23	23	23	23	23	23	23	23	153	153	153	153	37	37	37	37	37	N/A	23	23	109	110	154	18	38
SAUR2768 (SAR_RS13840)	cwrA	2658039	7	7	7	7	7	7	7	7	7	7	29	29	29	29	7	7	7	7	7	29	7	7	5	13	7	38	7
SAUR2769 (SAR_RS13845)	copA	2658470	139	139	139	139	139	139	N/A	729	139	N/A	N/A	862	821	N/A	N/A	468	434	181	303	820	139	139	803	42	226	306	N/A
SAUR2770 (SAR_RS13850)	copZ	2661162	7	7	7	7	7	7	7	7	7	7	4	4	4	4	1	1	1	1	1	4	7	7	4	4	68	1	1
SAUR2771 (SAR_RS13855)	ddh	2661458	440	440	38	38	38	38	N/A	79	38	79	N/A	16	16	N/A	106	106	106	106	106	16	38	38	8	26	136	164	108
SAUR2772 (SAR_RS13860)	ywfG	2662479	93	93	93	93	93	93	93	93	93	173	150	150	150	150	118	118	118	118	118	481	93	93	9	35	427	183	N/A
SAUR2773 (SAR_RS13865)	crtN	2664060	101	101	101	101	101	101	101	101	101	101	N/A	606	606	N/A	343	150	342	150	150	607	101	101	10	25	162	251	151
SAUR2774 (SAR_RS13870)	crtM	2665580	376	376	6	6	6	6	6	6	6	6	16	16	16	16	99	99	99	99	99	N/A	6	6	7	20	104	104	101
SAUR2775 (SAR_RS13875)	crtO	2666480	87	87	87	N/A	N/A	87	87	87	87	87	151	151	151	151	121	121	121	121	121	151	N/A	N/A	7	23	125	199	N/A
SAUR2776 (SAR_RS13880)	crtP	2667613	103	103	103	103	103	103	N/A	103	103	103	N/A	181	181	N/A	2	2	2	2	2	181	N/A	N/A	7	32	148	242	N/A
SAUR2777 (SAR_RS13885)	crtQ	2669099	2	2	2	2	2	2	2	2	2	2	13	13	13	13	60	124	60	60	60	N/A	2	2	17	13	64	83	35
SAUR2778 (SAR_RS13890)	ssaA1	2669762	25	25	25	25	25	25	25	25	25	25	6	6	6	6	84	84	177	84	84	N/A	25	25	43	360	104	134	85
SAUR2779 (SAR_RS13895)	oatA	2670891	36	N/A	36	36	36	36	36	36	36	36	N/A	154	154	N/A	126	126	126	126	126	154	36	36	428	75	132	203	127
SAUR2780 (SAR_RS13900)	isaA	2673391	29	29	N/A	N/A	N/A	N/A	29	29	29	29	57	57	57	57	29	29	29	29	29	325	29	29	7	12	115	139	80
SAUR2781 (SAR_RS13905)	Q5HCY0	2674701	73	73	73	73	73	73	73	73	73	73	N/A	78	78	N/A	56	56	56	56	56	78	73	73	342	25	122	160	98
SAUR2783 (SAR_RS13915)	N/A	2676165	2	2	N/A	N/A	N/A	N/A	2	2	2	2	18	18	18	18	N/A	121	121	77	121	18	2	2	8	23	47	122	78
SAUR2784 (SAR_RS13920)	Q5HCX8	2677130	29	29	29	29	29	29	29	29	29	29	73	73	73	73	35	35	57	35	35	73	29	29	73	73	37	47	36
SAUR2785 (SAR_RS13925)	ynzC	2677670	26	26	N/A	N/A	N/A	N/A	26	26	26	26	4	4	4	4	2	2	2	2	2	90	26	26	6	4	38	26	1
SAUR2786 (SAR_RS13930)	Q5HCX6	2678121	2	2	2	2	2	2	2	2	2	2	59	59	59	59	12	12	12	12	12	59	2	2	6	6	3	3	12
SAUR2787 (SAR_RS13935)	nmrA	2678515	60	60	60	60	60	60	60	60	60	60	7	7	7	7	80	80	80	80	80	N/A	60	60	7	7	100	136	N/A
SAUR2788 (SAR_RS13940)	Q5HCX4	2679356	11	11	11	11	11	11	11	11	11	11	N/A	N/A	N/A	N/A	38	38	38	38	38	N	11	11	3	30	39	11	39
SAUR2789 (SAR_RS13945)	gbaA	2679786	22	22	22	22	22	22	22	22	22	22	28	28	28	28	62	62	62	62	62	N/A	22	22	14	35	11	2	10
SAUR2790 (SAR_RS13950)	gbaB	2680336	321	321	62	62	62	62	62	62	62	62	96	96	96	96	78	78	78	78	78	96	62	62	16	25	79	74	79
SAUR2791 (SAR_RS13955)	Q5HCX0	2681138	97	97	97	97	97	97	97	97	97	97	248	248	468	248	118	118	118	118	118	N/A	97	97	10	33	143	179	N/A
SAUR2792 (SAR_RS13960)	vraK	2682168	396	396	33	33	33	33	33	33	33	33	N/A	120	120	N/A	154	154	215	N/A	154	N/A	N/A	N/A	6	11	121	158	53
SAUR2793 (SAR_RS13965)	cobW3	2683144	70	70	N/A	70	70	70	N/A	70	70	70	106	355	106	106	90	90	90	90	90	106	70	70	38	11	108	142	N/A
SAUR2794 (SAR_RS13970)	Q6GDL8	N/A	N/A	N/A	N/A	N/A	N/A	N/A	N/A	N/A	282	N/A	N/A	372	372	N/A	138	138	138	138	138	N/A	N/A	N/A	12	25	170	138	139
SAUR2795 (SAR_RS13975)	Q5HCW6	2685387	82	82	N/A	N/A	N/A	N/A	107	82	230	82	131	131	131	131	242	107	N/A	107	107	131	82	82	8	16	132	181	N/A
SAUR2796 (SAR_RS13980)	DUF4176	2686828	2	2	2	2	2	2	35	2	2	2	N/A	2	2	2	35	35	35	35	35	N/A	2	2	6	12	35	45	45
SAUR2797 (SAR_RS13985)	Q8NUP1	2687112	16	16	16	16	16	16	43	16	16	16	53	53	N/A	53	43	43	43	43	43	53	16	16	22	23	43	66	44
SAUR2798 (SAR_RS13990)	Q8NUP0	2687495	25	25	25	25	25	25	12	25	25	25	45	45	45	45	12	12	12	12	12	45	25	25	90	5	46	2	41
SAUR2799 (SAR_RS13995)	Q8NUN9	2688110	65	65	N/A	65	65	N/A	26	65	166	65	106	106	106	106	26	26	26	26	137	106	166	166	259	13	84	139	22
SAUR2800 (SAR_RS14000)	pyrD	2689194	77	77	77	77	77	77	N/A	77	77	77	125	125	125	125	2	2	2	2	2	390	77	77	5	N/A	133	159	2
SAUR2801 (SAR_RS14005)	Q5HCW0	2690413	25	25	25	25	25	25	3	25	25	25	3	3	3	3	3	3	3	3	3	3	25	25	5	3	2	47	3
SAUR2802 (SAR_RS14010)	DUF208	2690874	2	2	N/A	N/A	N/A	N/A	2	2	2	2	64	64	64	64	2	2	2	2	2	64	2	2	31	13	2	91	2
SAUR2803 (SAR_RS14015)	Q5HCV8	2691946	43	43	43	43	43	43	38	43	N/A	43	65	65	65	65	38	38	38	38	38	65	43	43	5	5	67	52	38
SAUR2806 (SAR_RS14030)	pepX	2693206	133	133	133	133	133	133	N/A	133	133	133	N/A	216	216	N/A	277	277	277	170	277	N/A	N/A	N/A	12	30	174	174	171
SAUR2807 (SAR_RS14035)	panD	2695969	17	17	17	17	17	17	17	17	17	17	53	53	53	53	17	17	17	17	17	53	N/A	N/A	7	1	45	63	21
SAUR2808 (SAR_RS14040)	panC	2696354	26	26	309	26	26	309	N/A	26	26	26	N/A	5	5	5	55	55	55	55	55	5	26	26	41	11	82	17	81
SAUR2809 (SAR_RS14045)	panB	2697198	4	4	4	4	4	4	4	4	4	4	N/A	178	178	178	64	64	64	64	64	N/A	4	4	9	22	4	20	65
SAUR2810 (SAR_RS14050)	panE2	2698089	62	62	62	62	62	62	62	62	62	62	112	112	340	112	62	197	62	62	62	112	N/A	N/A	52	29	62	146	96
SAUR2811 (SAR_RS14055)	alsD-L2=budA	2699035	3	3	3	3	3	3	N/A	293	3	3	9	9	9	9	83	197	83	83	83	329	N/A	N/A	9	24	9	135	N/A
SAUR2812 (SAR_RS14060)	ldh2	2700017	2	2	2	2	2	2	N/A	2	2	2	120	120	120	120	99	99	99	99	99	61	2	2	6	25	61	4	194
SAUR2813 (SAR_RS14065)	N/A	2701537	2	2	N/A	N/A	N/A	N/A	2	2	2	2	2	2	2	2	40	2	2	N/A	40	N/A	2	2	2	2	2	2	2
SAUR2814 (SAR_RS14070)	yfnA1	2701663	112	112	112	N/A	N/A	112	N/A	109	112	109	194	194	194	194	151	151	332	145	151	151	N/A	N/A	566	35	151	247	147
SAUR2815 (SAR_RS14075)	yhxA=gabT	2703191	104	104	104	104	104	104	129	104	104	104	377	377	546	377	129	295	278	129	129	N/A	104	104	10	16	153	129	N/A
SAUR2816 (SAR_RS14080)	Q5HCU7	2704782	53	53	N/A	N/A	N/A	N/A	64	53	53	53	78	78	78	78	64	64	64	64	64	64	53	53	7	7	N/A	84	41
SAUR2817 (SAR_RS14085)	fda	2705321	7	7	7	7	7	7	285	279	7	7	164	164	164	164	N/A	40	40	40	40	285	7	7	116	33	53	40	20
SAUR2818 (SAR_RS14090)	lqo	2706405	78	78	78	78	78	78	2	89	78	78	421	126	126	421	2	2	2	2	2	2	78	78	6	81	134	2	13
SAUR2819 (SAR_RS14095)	N/A	2708326	2	2	2	2	2	2	2	2	2	2	17	17	17	17	2	2	2	2	2	N/A	2	2	4	2	2	2	2
SAUR2820 (SAR_RS14100)	acsA2=bclA	2708586	110	110	110	110	110	110	136	110	110	110	304	304	570	304	136	136	316	136	136	136	N/A	N/A	14	115	178	225	138
SAUR2821 (SAR_RS14105)	Q5HCU3	2710378	65	65	65	65	65	65	79	65	65	65	87	87	195	87	79	79	79	79	79	79	65	65	9	67	81	102	80
SAUR2822 (SAR_RS14110)	Q5HCU2	2711101	15	15	15	15	15	15	1	15	15	15	1	1	1	1	1	1	1	1	1	68	15	15	1	11	1	1	1
SAUR2823 (SAR_RS14115)	betA	2711590	94	94	94	94	94	94	N/A	94	94	94	149	149	489	149	117	117	268	117	117	N	N/A	N/A	8	99	441	203	118
SAUR2824 (SAR_RS14120)	betB	2713560	461	461	N/A	52	52	N/A	N/A	52	52	52	141	141	141	141	111	111	111	111	111	111	52	52	7	113	113	184	103
SAUR2825 (SAR_RS14125)	A8Z5A6	2715119	1	1	1	1	1	1	1	1	1	1	1	1	1	1	1	1	1	1	1	1	1	1	1	3	3	1	1
SAUR2826 (SAR_RS14130)	Q79ZX2	2715303	2	2	2	2	2	2	63	2	2	2	8	8	8	8	63	63	63	63	63	63	N/A	N/A	8	52	52	97	64
SAUR2827 (SAR_RS14135)	cudT	2716036	85	85	N/A	N/A	N/A	N/A	85	85	85	85	140	140	140	140	N/A	85	85	85	85	85	N/A	N/A	12	92	76	85	67
SAUR2828 (SAR_RS14140)	N/A	N/A	N/A	N/A	N/A	N/A	N/A	N/A	N/A	N/A	N/A	N/A	115	115	115	115	N/A	N/A	N/A	N/A	N/A	N/A	N/A	N/A	3	3	6	N/A	6
SAUR2829 (SAR_RS14145)	N/A	2717967	4	4	4	4	4	4	4	4	4	4	5	5	5	5	4	4	4	4	4	N/A	4	4	7	N/A	4	4	15
SAUR2830 (SAR_RS14150)	nrdG	2718177	10	10	10	10	10	10	10	10	10	10	8	8	8	8	10	10	10	10	10	151	N/A	N/A	8	18	10	10	10
SAUR2831 (SAR_RS14155)	nrdD	2718710	532	532	94	94	94	94	N/A	94	94	94	361	361	361	361	124	124	124	124	124	512	94	94	8	99	256	200	125
SAUR2832 (SAR_RS14160)	citM	2720807	74	74	74	74	74	74	117	74	74	74	139	139	139	139	117	117	262	117	117	117	N/A	N/A	410	N/A	142	N/A	N/A
SAUR2834 (SAR_RS14170)	cysG	2722652	61	61	61	61	61	61	65	61	61	61	N/A	N/A	N/A	N/A	65	65	65	65	65	N/A	65	65	33	65	52	91	70
SAUR2835 (SAR_RS14175)	cysJ	2723320	768	768	129	N/A	N/A	129	169	129	129	129	741	214	214	741	405	169	169	169	283	169	N/A	N/A	9	130	178	288	N/A
SAUR2836 (SAR_RS14180)	N/A	2725395	2	2	2	2	2	2	2	2	2	2	5	5	5	5	N/A	2	2	2	2	N/A	2	2	5	2	2	N/A	2
SAUR2837 (SAR_RS14185)	gpxA-L2	2725595	10	10	10	10	10	10	10	10	10	10	56	56	56	56	10	10	10	10	10	10	10	10	4	10	48	10	10
SAUR2838 (SAR_RS14190)	A6QKB0	2726102	118	118	25	N/A	N/A	25	3	25	25	25	74	74	74	74	3	3	121	3	3	N/A	3	3	6	3	N/A	3	25
SAUR2839 (SAR_RS14195)	N/A	2726744	14	14	14	14	N/A	N/A	N/A	90	14	14	16	16	16	16	36	69	36	36	36	N/A	36	36	N/A	28	2	3	14
SAUR2841 (SAR_RS14205)	nsaB	2727521	134	134	N/A	N/A	N/A	N/A	N/A	134	134	134	N/A	751	751	N/A	168	431	168	168	168	N/A	168	168	686	N/A	673	291	134
SAUR2842 (SAR_RS14210)	nsaA	2729518	60	60	60	60	60	60	79	60	15	60	29	29	29	29	N/A	79	79	79	79	79	79	79	7	N/A	15	2	15
SAUR2843 (braS)	N/A	2730381	61	61	N/A	N/A	N/A	N/A	2	61	N/A	61	7	7	7	7	87	87	87	87	87	341	2	2	7	2	108	2	2
SAUR2844 (braR)	N/A	2731279	55	55	55	55	55	55	2	55	55	55	N/A	87	87	N/A	2	2	2	2	2	2	2	2	8	57	2	2	2
SAUR2845 (MW2546)	N/A	2731970	2	2	2	2	2	2	2	2	2	2	1	1	1	1	2	2	2	2	2	2	2	2	7	25	N/A	2	2
SAUR2846 (SAR_RS14230)	N/A	2732136	1	1	1	1	1	1	10	1	1	1	1	1	1	1	10	10	10	10	10	N/A	10	10	1	1	1	1	1
SAUR2849 (SAR_RS14245)	slyA	2734075	8	8	N/A	8	8	8	101	8	8	8	8	8	8	8	1	1	1	1	1	101	101	101	1	25	25	56	8
SAUR2850 (SAR_RS14250)	estA	2734775	N/A	68	68	N/A	N/A	68	N/A	68	68	68	107	107	107	107	86	86	86	86	86	86	N/A	N/A	6	73	108	108	87
SAUR2852 (SAR_RS14260)	arcR	2738733	49	49	49	49	49	49	35	49	49	49	15	15	15	15	35	35	35	35	35	35	35	35	6	52	63	97	58
SAUR2853 (SAR_RS14265)	arcC-L2	2739536	N/A	65	65	65	65	65	29	65	163	65	103	103	103	103	29	29	29	29	29	29	N/A	N/A	6	68	29	29	82
arcC	arcC	2739851	6	6	6	6	6	6	18	6	6	6	12	12	12	12	18	18	18	18	18	N/A	N/A	N/A	1	18	18	18	6
SAUR2854 (SAR_RS14270)	arcD-L2	2740494	35	35	N/A	N/A	N/A	N/A	124	35	35	35	156	156	156	156	N/A	124	124	124	124	479	N/A	N/A	392	35	35	204	N/A
SAUR2855 (SAR_RS14275)	arcB-L2	2742015	31	31	31	31	31	31	N/A	31	31	31	8	8	8	8	108	108	108	108	108	N	N/A	N/A	8	31	135	49	2
SAUR2856 (SAR_RS14280)	arcA-L2	2743058	75	75	75	75	75	75	N/A	380	75	75	120	120	120	120	99	99	99	99	99	59	59	59	6	59	59	154	100
SAUR2857 (SAR_RS14285)	N/A	2744470	2	2	105	2	2	105	24	2	2	2	11	11	11	11	29	29	29	29	29	N/A	N/A	N/A	N/A	2	2	52	6
SAUR2858 (SAR_RS14290)	N/A	2744640	34	34	34	N/A	34	34	30	34	15	34	4	4	4	4	30	30	30	30	30	170	170	170	4	15	15	64	30
SAUR2859 (SAR_RS14295)	aur	2745456	200	200	200	200	200	200	N/A	200	200	200	278	479	479	278	253	253	401	253	253	N/A	N/A	N/A	183	234	200	200	254
SAUR2862 (SAR_RS14310)	manR	2748892	N/A	35	35	35	35	35	N/A	35	35	35	N/A	181	181	181	140	140	333	140	241	639	639	639	57	102	182	N/A	141
SAUR2863 (SAR_RS14315)	manP	2750843	42	42	N/A	N/A	N/A	N/A	N/A	42	N/A	42	N/A	207	728	N/A	N/A	163	384	163	163	129	129	129	5	129	171	277	N/A
SAUR2864 (SAR_RS14320)	manA2=yvyI	2752809	65	65	N/A	65	65	65	373	65	65	65	8	8	8	8	57	57	57	57	57	373	373	373	8	68	87	13	N/A
SAUR2865 (SAR_RS14325)	yhgE	2753859	104	104	104	104	104	104	N/A	104	104	104	N/A	275	275	N/A	220	572	220	220	220	N/A	N/A	N/A	9	167	278	372	N/A
SAUR2866 (SAR_RS14330)	scaH=lytZ	2757051	95	95	N/A	N/A	N/A	N/A	144	95	95	95	177	177	177	177	N/A	144	144	144	144	144	144	144	6	112	144	144	144
SAUR2867 (SAR_RS14335)	ywoC1	2760840	38	38	38	38	38	38	54	38	38	38	6	6	6	6	54	54	54	54	54	54	54	54	5	43	58	58	54
SAUR2868 (SAR_RS14340)	sasF	N/A	755	755	139	139	139	139	N/A	N/A	139	139	238	238	731	238	180	446	180	180	180	N/A	N/A	N/A	48	195	660	320	N/A
SAUR2869 (SAR_RS14345)	gtfB	2763721	111	111	111	111	111	111	N/A	111	111	111	170	170	170	170	140	140	140	140	140	N/A	515	515	N/A	111	457	N/A	141
SAUR2870 (SAR_RS14350)	gtfA	2765072	105	105	N/A	N/A	N/A	N/A	N/A	105	105	105	317	410	410	317	135	135	135	135	231	534	534	534	49	99	175	175	136
SAUR2871 (SAR_RS14355)	secA2	2766598	181	181	N/A	N/A	N/A	N/A	N/A	181	469	181	189	189	189	189	234	234	234	234	234	933	N/A	N/A	12	186	N/A	N/A	N/A
SAUR2873 (SAR_RS14365)	asp2	2769915	126	126	N/A	N/A	N/A	N/A	N/A	126	126	126	204	204	204	204	269	269	269	161	269	682	682	682	72	254	208	273	95
SAUR2875 (SAR_RS14375)	secY2	2773037	72	72	N/A	N/A	N/A	N/A	117	72	72	72	N/A	141	141	N/A	117	117	117	117	192	117	N/A	N/A	11	86	143	197	118
SAUR2877 (SAR_RS14385)	A6U540	2781621	30	30	30	30	30	30	2	281	30	30	157	157	286	157	2	2	2	2	2	N/A	2	2	128	37	48	30	9
SAUR2879 (SAR_RS14395)	Q8NUJ1	N/A	63	63	63	N/A	N/A	63	18	N/A	45	63	28	28	28	28	18	18	18	16	18	N/A	18	18	N/A	N/A	N/A	N/A	N/A
SAUR2880 (SAR_RS14400)	Q5HCP0	N/A	N/A	N/A	N/A	N/A	N/A	N/A	19	N/A	N/A	N/A	N/A	57	57	57	19	19	19	19	19	N/A	93	93	41	N/A	20	20	2
SAUR2881 (SAR_RS14405)	Q5HCN9	2787137	6	6	6	6	6	6	36	6	6	6	17	17	17	17	36	36	97	36	36	36	36	36	13	39	39	39	53
SAUR2882 (SAR_RS14410)	ywrF	2787582	20	20	N/A	N/A	N/A	N/A	26	20	20	20	10	10	10	N/A	26	26	26	26	26	26	N/A	N/A	6	N/A	206	2	68
SAUR2883 (SAR_RS14415)	msrA3	2788272	39	39	16	16	16	16	N/A	39	39	39	77	77	77	77	68	68	68	68	68	16	N/A	N/A	8	N/A	39	95	2
SAUR2884 (SAR_RS14420)	Q5HCN6	2788751	2	2	26	26	26	26	26	2	2	2	75	75	75	75	26	26	26	26	26	26	26	26	8	N/A	76	20	2
SAUR2885 (SAR_RS14425)	capC-L2	2789486	333	333	85	N/A	85	85	N/A	50	50	50	102	102	102	102	186	85	85	85	85	85	N/A	N/A	8	N/A	50	24	14
SAUR2886 (SAR_RS14430)	capB-L2	2790250	18	18	76	76	76	76	76	310	18	18	N/A	102	102	N/A	N/A	76	76	76	76	76	76	76	10	N/A	84	138	76
SAUR2887 (SAR_RS14435)	capA-L2	2790959	49	49	81	81	81	81	81	49	49	49	104	104	104	N/A	81	81	178	81	81	81	N/A	N/A	5	N/A	84	127	N/A
SAUR2888 (SAR_RS14440)	icaR	2792469	46	46	2	N/A	2	2	2	46	46	46	69	69	204	69	2	2	2	2	2	2	N/A	N/A	7	N/A	2	20	54
SAUR2889 (SAR_RS14445)	icaA	2793193	2	2	65	65	65	65	N/A	2	2	2	137	137	137	137	N/A	65	65	65	65	506	613	613	9	N/A	N/A	64	65
SAUR2890 (SAR_RS14450)	icaD	2794395	2	2	2	N/A	2	2	2	2	2	2	33	33	33	33	N/A	2	2	2	2	N/A	2	2	2	N/A	28	2	2
SAUR2891 (SAR_RS14455)	icaB	2794697	69	69	95	N/A	95	95	95	69	69	69	37	37	37	37	95	227	95	95	95	N/A	N/A	N/A	8	N/A	112	N/A	89
SAUR2892 (SAR_RS14460)	icaC	2795556	2	2	214	N/A	214	N/A	214	2	2	2	132	132	132	N/A	N/A	214	214	N/A	157	N/A	N/A	N/A	331	N/A	133	158	133
SAUR2893 (SAR_RS14465)	lip1	2796999	135	135	N/A	N/A	N/A	N/A	N/A	117	135	140	N/A	527	746	527	N/A	166	166	166	166	745	N/A	N/A	7	N/A	227	290	168
SAUR2895 (SAR_RS14475)	hisI	2800562	56	56	74	74	74	74	74	56	56	56	88	88	88	88	74	74	74	74	74	N/A	74	74	6	N/A	77	114	N/A
SAUR2896 (SAR_RS14480)	hisF	2801191	69	69	97	97	97	97	N/A	306	69	69	263	263	263	263	97	97	97	97	97	97	97	97	9	N/A	24	149	98
SAUR2897 (SAR_RS14485)	hisA	2801946	54	54	80	80	80	80	80	54	148	54	N/A	22	22	N/A	80	80	80	80	80	265	265	265	5	N/A	238	122	81
SAUR2898 (SAR_RS14490)	hisH	2802643	19	19	N/A	N/A	N/A	N/A	19	19	19	19	209	28	28	209	19	19	19	19	19	N/A	19	19	8	N/A	88	125	86
SAUR2899 (SAR_RS14495)	hisB	2803218	227	227	8	8	8	8	8	39	39	N/A	76	76	76	76	136	8	8	8	8	N/A	8	8	19	N/A	39	103	8
SAUR2900 (SAR_RS14500)	hisC1	2803765	37	37	37	37	37	37	37	307	307	307	177	429	177	177	37	37	37	37	37	37	37	37	122	N/A	308	N/A	30
SAUR2901 (SAR_RS14505)	hisD	2804794	563	563	563	563	563	563	62	115	110	N/A	N/A	164	164	N/A	N/A	62	62	62	62	62	62	62	11	N/A	166	N/A	N/A
SAUR2902 (SAR_RS14510)	hisG	2806037	61	61	61	61	61	61	61	20	20	20	182	182	182	182	25	25	25	25	25	N/A	61	61	73	N/A	28	N/A	26
SAUR2903 (SAR_RS14515)	hisZ	2806669	76	76	76	76	76	76	N/A	16	16	16	98	98	98	98	76	N/A	76	76	76	76	76	76	8	N/A	N/A	2	N/A
SAUR2904 (SAR_RS14520)	Q5HCL6	2807792	425	425	425	425	425	425	425	66	68	66	104	104	104	N/A	146	146	146	84	146	146	146	146	4	N/A	107	149	85
SAUR2910 (SAR_RS14550)	yceI	2809329	63	63	63	63	63	63	63	45	45	45	N/A	42	42	N/A	63	63	63	63	63	186	63	63	6	N/A	2	92	2
SAUR2911 (SAR_RS14555)	N/A	2809994	389	389	389	389	389	389	N/A	26	26	26	135	425	135	N/A	421	421	421	421	421	389	389	389	118	N/A	49	N/A	41
SAUR2912 (SAR_RS14560)	Q5HCK8	2811209	157	157	157	157	157	157	157	73	73	73	119	119	119	119	157	225	157	96	157	157	157	157	7	N/A	101	159	97
SAUR2913 (SAR_RS14565)	pcp	2812317	N/A	177	177	177	177	177	177	2	2	2	N/A	148	148	N/A	22	22	22	22	22	177	177	177	60	N/A	N/A	22	23
SAUR2914 (SAR_RS14570)	Q2FUS3=bstA	2813058	17	17	17	17	17	17	17	41	41	41	25	25	25	25	17	17	17	17	17	17	17	17	4	50	78	17	N/A
SAUR2916 (SAR_RS14580)	Q7WS08	2813761	135	135	135	135	135	135	135	103	103	103	166	166	166	166	135	135	135	135	135	135	135	135	7	112	167	210	137
SAUR2917 (SAR_RS14585)	rarD	2815604	371	371	95	95	95	95	N/A	67	67	N/A	123	N/A	N/A	123	95	95	95	95	95	95	95	95	23	83	126	293	96
SAUR2918 (SAR_RS14590)	Q5HCK1	2816734	215	215	215	N/A	N/A	215	215	16	16	16	67	67	67	67	57	57	57	57	57	N/A	N/A	N/A	141	53	59	85	N/A
SAUR2919 (SAR_RS14595)	N/A	2817437	375	375	375	N/A	N/A	375	375	16	16	16	220	N/A	N/A	220	N/A	102	102	102	102	375	N/A	N/A	25	85	N/A	168	N/A
SAUR2920 (SAR_RS14600)	N/A	N/A	N/A	N/A	N/A	N/A	N/A	N/A	N/A	N/A	N/A	N/A	138	138	138	138	N/A	N/A	N/A	N/A	N/A	N/A	N/A	N/A	45	N/A	N/A	N/A	N/A
SAUR2921 (SAR_RS14605)	hdeD	2819840	35	35	35	35	35	35	35	28	28	28	N/A	92	92	N/A	35	35	35	35	35	35	35	35	92	30	N/A	62	2
SAUR2922 (SAR_RS14610)	vraD	2820642	83	83	83	N/A	N/A	83	83	63	63	63	N/A	10	10	N/A	83	83	83	83	83	N/A	N/A	N/A	9	68	87	126	2
SAUR2923 (SAR_RS14615)	vraE	2821390	122	122	122	122	122	N/A	122	47	47	47	455	455	704	455	172	122	122	66	122	703	122	122	270	54	96	123	N/A
SAUR2929 (SAR_RS14645)	N/A	2823946	1	1	1	N/A	N/A	1	1	1	1	1	1	1	1	1	1	1	1	1	1	1	N/A	N/A	1	1	1	1	1
SAUR2930 (SAR_RS14650)	Q5HCI9=immR	2824273	18	18	18	18	18	18	18	49	49	49	N/A	75	75	75	18	18	18	18	18	18	18	18	7	52	49	92	N/A
SAUR2931 (SAR_RS14655)	N/A	2825102	1	1	1	1	1	1	1	27	27	27	4	4	4	4	1	1	1	1	1	1	1	1	1	1	33	7	1
SAUR2932 (SAR_RS14660)	DUF3147_L2	2825565	2	2	2	2	2	2	2	22	22	22	6	6	6	6	62	62	62	62	62	2	2	2	9	3	2	22	22
SAUR2934 (SAR_RS14670)	noc	2826586	13	13	13	13	13	13	13	25	25	25	N/A	86	86	N/A	68	68	68	68	103	13	13	13	6	59	13	13	2
SAUR2935 (SAR_RS14675)	rsmG=gidB	2827468	74	74	74	74	74	74	74	62	62	62	11	11	11	11	74	74	74	74	74	74	74	74	7	63	94	111	N/A
SAUR2936 (SAR_RS14680)	mnmG=gidA	2828187	537	537	214	214	214	214	214	101	N/A	101	N/A	10	10	N/A	209	302	209	124	209	214	214	214	9	77	129	214	125
SAUR2937 (SAR_RS14685)	mnmE=trmE	2830131	106	106	N/A	N/A	N/A	N/A	106	46	46	46	8	8	8	8	237	106	106	106	106	106	N/A	N/A	9	89	110	159	68
SAUR2938 (SAR_RS14690)	rnpA	2831653	4	4	N/A	N/A	N/A	N/A	N/A	4	4	4	4	4	4	4	4	4	4	4	4	4	170	170	4	39	4	69	18
SAUR2939 (SAR_RS14695)	N/A	2832127	2	2	2	2	2	2	2	2	2	2	2	2	2	2	2	2	2	2	2	2	2	2	2	2	2	2	2
