# Supplementary material for: Molecular Characterization of Chimeric Staphylococcus aureus Strains from Waterfowl
Source: Microorganisms. 2024 Jan 3;12(1):96. doi: 10.3390/microorganisms12010096 (PMC10821479; doi:10.3390/microorganisms12010096)
Supplement: Supplementary file 1 [file microorganisms-12-00096-s001.zip › Supplemental file 3b_Numerical values used for construction of Figure 1a to 3a_2023-12-12.pdf]

Number/ Position in the figures	Gene ID	ID in PubMLST cg scheme	ED133 vs. swan isolate 15V8707	X22 vs. swan isolate 15V8707	CC522 goat isolate 17CS1042 vs. swan isolate 15V8707	ED133 vs. duck isolate V315	X22 vs. duck isolate V315	CC522 goat isolate 17CS1042 vs. duck isolate V315	ED133 vs. duck isolate V482	X22 vs. duck isolate V482	CC522 goat isolate 17CS1042 vs. duck isolate V482
>0001	dnaA	SAUR0001 (SAR_RS00005)	0,81	0,07	1,10	0,73	0,00	1,03	0,81	0,07	1,10
>0002	dnaN	SAUR0002 (SAR_RS00010)	0,71	0,09	0,27	0,71	0,09	0,27	0,88	0,27	0,44
>0003	yaaA	SAUR0003 (SAR_RS00015)	0,41	0,00	0,41	0,41	0,00	0,41	0,41	0,00	0,41
>0004	recF	SAUR0004 (SAR_RS00020)	0,18	0,09	0,36	0,18	0,09	0,36	0,18	0,09	0,36
>0005	gyrB	SAUR0005 (SAR_RS00025)	0,47	0,00	0,62	0,47	0,00	0,62	0,47	0,00	0,62
>0006	gyrA	SAUR0006 (SAR_RS00030)	0,34	1,27	0,67	1,61	0,07	1,65	1,61	0,07	1,65
>0007	nnrD	SAUR0007 (SAR_RS00035)	0,12	1,68	0,24	1,56	0,00	1,68	1,56	0,00	1,68
>0008	hutH	SAUR0008 (SAR_RS00040)	0,00	1,32	0,66	1,32	0,00	1,32	1,39	0,07	1,39
>0009	serS	SAUR0009 (SAR_RS00045)	0,00	0,62	0,62	0,62	0,00	0,47	0,70	0,08	0,54
>0010	azlC=ygaZ	SAUR0010 (SAR_RS00050)	0,00	1,58	0,29	1,58	0,00	1,58	1,58	0,00	1,58
>0011	azlD	SAUR0011 (SAR_RS00055)	0,00	1,21	1,21	1,52	0,30	1,52	1,52	0,30	1,52
>0012	metX	SAUR0012 (SAR_RS00060)	0,10	2,27	1,65	2,17	0,00	2,99	2,17	0,00	2,99
>0013	yybS=DUF2232	SAUR0013 (SAR_RS00065)	0,11	0,76	0,54	0,65	0,00	0,43	0,65	0,00	0,43
>0014	gdpP	SAUR0014 (SAR_RS00070)	0,00	1,22	0,66	1,22	0,00	1,27	1,22	0,00	1,27
>0015	rplI	SAUR0015 (SAR_RS00075)	0,00	0,67	0,45	0,67	0,00	0,67	0,67	0,00	0,67
>0016	dnaC	SAUR0016 (SAR_RS00080)	0,00	1,28	0,86	1,28	0,00	1,86	1,28	0,00	1,86
>0017	purA	SAUR0017 (SAR_RS00085)	0,00	2,26	0,47	2,26	0,00	2,57	2,26	0,00	2,57
>0018	walR=yycF=vicR	SAUR0020 (SAR_RS00100)	0,00	1,00	1,00	1,00	0,00	1,14	1,14	0,14	1,28
>0019	walK	SAUR0021 (SAR_RS00105)	0,00	0,71	0,66	0,71	0,00	0,60	0,82	0,11	0,71
>0020	walH=yycH	SAUR0022 (SAR_RS00110)	0,07	1,12	0,75	1,05	0,15	1,12	1,05	0,15	1,12
>0021	walI=yycI	SAUR0023 (SAR_RS00115)	0,13	1,27	0,51	1,39	0,00	1,01	1,39	0,00	1,01
>0022	walJ=yycJ	SAUR0024 (SAR_RS00120)	0,00	0,87	1,12	0,87	0,00	1,00	0,87	0,00	1,00
>0023	sasH=adsA	SAUR0025 (SAR_RS00125)	0,00	2,29	1,29	2,29	0,00	1,90	2,33	0,04	1,94
>0024	orfX	SAUR0026 (SAR_RS00130)	0,00	2,92	2,50	2,92	1,25	2,08	2,71	1,04	2,29
>0031	C1PH96	(N/A)	0,00	>=5,00	0,00	>=5,00	0,00	>=5,00	>=5,00	0,00	>=5,00
>0036	lrpC	(N/A)	0,00	>=5,00	0,00	>=5,00	0,24	>=5,00	>=5,00	0,24	>=5,00
>0037	G0LPR5	(N/A)	0,00	0,00	>=5,00	0,00	0,00	>=5,00	0,00	0,00	>=5,00
>0043	hsdR2_CC22/93/425	(N/A)	0,00	0,00	>=5,00	0,00	0,00	>=5,00	0,00	0,00	>=5,00
>0055	Q6GD44	(N/A)	0,00	>=5,00	0,00	>=5,00	0,00	>=5,00	>=5,00	0,00	>=5,00
>0056	DUF81-GI	(N/A)	0,13	0,66	0,26	>=5,00	>=5,00	>=5,00	>=5,00	>=5,00	>=5,00
>0057	cstR-GI	(N/A)	0,00	1,15	0,00	>=5,00	>=5,00	>=5,00	>=5,00	>=5,00	>=5,00
>0058	cstA-GI	(N/A)	1,31	0,84	0,56	>=5,00	>=5,00	>=5,00	>=5,00	>=5,00	>=5,00
>0059	cstB-GI	(N/A)	0,00	0,45	0,37	>=5,00	>=5,00	>=5,00	>=5,00	>=5,00	>=5,00
>0060	sqr=fccB	(N/A)	0,00	1,01	0,67	>=5,00	>=5,00	>=5,00	>=5,00	>=5,00	>=5,00
>0061	dusC	(N/A)	0,00	1,32	1,32	1,42	0,51	1,52	1,52	0,61	1,62
>0062	A6TXM6=DUF6007	(N/A)	0,00	>=5,00	>=5,00	>=5,00	0,00	>=5,00	>=5,00	0,00	>=5,00
>0063	A6QD71	(N/A)	0,00	>=5,00	>=5,00	>=5,00	0,00	4,62	>=5,00	0,00	4,62
>0069	Q5HJT2	(N/A)	0,00	0,00	>=5,00	0,00	0,00	>=5,00	0,00	0,00	>=5,00
>0070	Q6GD34	(N/A)	0,00	0,00	>=5,00	0,00	0,00	>=5,00	0,00	0,00	>=5,00
>0071	A6QD75	(N/A)	0,00	0,00	>=5,00	0,00	0,00	>=5,00	0,00	0,00	>=5,00
>0076	Q6GKK6	(N/A)	0,00	4,08	>=5,00	4,25	0,16	>=5,00	4,25	0,16	>=5,00
>0077	Q7A890	(N/A)	0,00	4,69	2,89	4,66	0,03	3,96	4,72	0,10	4,03
>0079	Q2YUT2=yut2	SAUR2960	0,41	2,48	1,86	2,48	0,00	2,69	2,48	0,00	2,69
>0080	plc	SAUR2961 (plc)	0,00	2,74	3,14	2,74	0,00	4,46	2,74	0,00	4,46
>0081	Q8NYT6	SAUR2962	0,00	3,04	1,52	2,99	0,04	2,95	2,99	0,04	2,95
>0082	Q8NYT5	SAUR2963	0,17	1,53	0,76	1,69	0,34	1,61	1,53	0,17	1,44
>0083	norC	SAUR2964	0,00	3,02	1,37	3,10	0,22	3,10	3,10	0,22	3,10
>0084	nptA	SAUR2965	0,00	0,90	0,36	0,90	0,00	1,14	0,90	0,00	1,14
>0085	Q2YUS5	SAUR2966	0,00	3,83	0,79	3,83	0,00	3,66	3,89	0,06	3,72
>0086	DUF1648	SAUR0106 (SAR_RS00530)	0,21	0,84	0,21	0,42	0,21	0,42	0,42	0,21	0,42
>0087	lctP-locus1	SAUR0107 (SAR_RS00535)	0,13	1,13	1,00	1,32	0,06	0,56	1,26	0,00	0,50
>0088	spa	(N/A)	3,53	>=5,00	>=5,00	>=5,00	>=5,00	4,27	>=5,00	>=5,00	>=5,00
>0089	sarS	SAUR0109 (SAR_RS00545)	0,13	0,53	0,13	0,66	0,00	0,66	0,66	0,00	0,66
>0090	sirC	SAUR0110 (SAR_RS00550)	0,00	0,40	0,30	0,30	0,30	0,20	0,20	0,20	0,10
>0091	sirB	SAUR0111 (SAR_RS00555)	0,00	0,90	0,20	0,80	0,10	0,80	0,80	0,10	0,80
>0092	sirA	SAUR0112 (SAR_RS00560)	0,00	0,20	0,30	0,20	0,00	0,30	0,20	0,00	0,30
>0093	sbnA=cysK/L1	SAUR0113 (SAR_RS00565)	0,00	0,41	0,10	0,41	0,00	0,31	0,41	0,00	0,31
>0094	sbnB	SAUR0114 (SAR_RS00570)	0,00	0,99	0,49	0,99	0,00	0,69	0,99	0,00	0,69
>0095	sbnC	SAUR0115 (SAR_RS00575)	0,06	1,60	1,08	1,65	0,11	1,88	1,60	0,06	1,82
>0096	sbnD	SAUR0116 (SAR_RS00580)	0,00	1,35	1,59	1,35	0,00	1,99	1,35	0,00	1,99
>0097	sbnE	SAUR0117 (SAR_RS00585)	0,06	2,01	1,04	1,96	0,00	2,01	2,01	0,06	2,07
>0098	sbnF=iucC	SAUR0118 (SAR_RS00590):	0,06	1,67	0,34	1,78	0,06	1,72	1,78	0,06	1,72
>0099	sbnG	SAUR0119 (SAR_RS00595)	0,00	1,29	0,13	1,42	0,13	1,54	1,29	0,00	1,42
>0100	sbnH	SAUR0120 (SAR_RS00600)	0,00	1,75	0,75	1,75	0,17	2,00	1,75	0,17	2,00
>0101	sbnI	SAUR0121 (SAR_RS00605)	0,13	0,13	0,26	0,26	0,00	0,13	0,26	0,00	0,13
>0102	Q5HJP3	(N/A)	0,00	>=5,00	0,38	>=5,00	0,00	>=5,00	>=5,00	0,00	>=5,00
>0103	butA	SAUR0124 (SAR_RS00620)	0,00	0,77	0,13	0,77	0,00	0,90	0,77	0,00	0,90
>0104	Q5HIL8=galE	SAUR0126 (SAR_RS00630)	0,00	1,13	0,31	1,03	0,10	0,72	1,03	0,10	0,72
>0105	wcaJ=tuaA	SAUR0127 (SAR_RS00635)	0,00	4,47	0,14	4,62	0,14	4,76	4,76	0,29	4,91
>0106	epsF	SAUR0128 (SAR_RS00640)	0,00	0,86	0,34	0,77	0,09	1,11	0,77	0,09	1,11
>0107	wzy	SAUR0129 (SAR_RS00645)	0,08	2,58	1,61	2,42	0,08	1,94	2,42	0,08	1,94
>0108	wzx	SAUR0130 (SAR_RS00650)	0,07	1,26	1,75	1,33	0,00	2,24	1,33	0,00	2,24
>0109	sodA-L1	SAUR0131 (SAR_RS00655)	>=5,00	1,17	0,83	>=5,00	0,00	1,00	>=5,00	0,00	1,00
>0110	sasD	(N/A)	0,00	>=5,00	0,00	>=5,00	0,00	>=5,00	>=5,00	0,00	>=5,00
>0111	Q5HJN3	SAUR0133 (SAR_RS00665)	0,00	0,93	0,40	0,93	0,00	1,06	0,93	0,00	1,06
>0112	deoD-L1	SAUR0135 (SAR_RS00675)	0,00	0,71	0,28	0,71	0,00	0,99	0,71	0,00	0,99
>0113	tet(38)=norB2	SAUR0136 (SAR_RS00680)	0,07	1,63	0,52	1,48	0,07	1,48	1,63	0,22	1,55
>0114	deoC-L1	SAUR1390 (SAR_RS06950)	0,30	1,21	0,75	1,06	0,15	1,21	1,06	0,15	1,21
>0115	deoB	SAUR0138 (SAR_RS00690)	0,08	0,59	0,51	0,51	0,00	0,76	0,51	0,00	0,76
>0116	phnE2	SAUR0139 (SAR_RS00695)	0,00	1,47	0,61	1,59	0,12	1,72	1,59	0,12	1,72
>0117	phnE1	SAUR0140 (SAR_RS00700)	0,12	0,75	0,00	1,50	0,62	1,37	1,50	0,62	1,37
>0118	phnC	SAUR0141 (SAR_RS00705)	0,26	1,94	1,03	1,68	0,00	2,45	1,68	0,00	2,45
>0119	phnB	SAUR0142 (SAR_RS00710)	0,00	1,15	0,42	1,15	0,00	1,36	1,15	0,00	1,36
>0120	Q5HJM4	SAUR0143 (SAR_RS00715)	0,00	1,17	0,06	1,10	0,06	1,17	1,17	0,13	1,23
>0121	cpdB	SAUR0144 (SAR_RS00720)	0,13	1,43	0,00	1,50	0,20	1,37	1,50	0,20	1,37
>0122	Q1Y4B9	(N/A)	0,09	>=5,00	0,77	>=5,00	0,00	>=5,00	>=5,00	0,00	>=5,00
>0123	adhE	SAUR0147 (SAR_RS00735)	0,04	0,80	0,80	0,77	0,08	0,65	0,61	0,23	0,80
>0124	capA-L1	SAUR0148 (SAR_RS00740)	0,00	0,45	0,15	0,45	0,00	0,60	0,00	0,45	0,15
>0125	capB-L1	SAUR0149 (SAR_RS00745)	0,00	3,06	0,00	3,20	0,15	3,20	0,00	3,06	0,00
>0126	capC-L1	SAUR0150 (SAR_RS00750)	0,00	0,52	0,00	0,39	0,13	0,39	0,13	0,65	0,13
>0127	capD	SAUR0151 (SAR_RS00755)	0,05	0,77	0,49	0,77	0,16	1,10	0,05	0,77	0,49
>0128	capE	SAUR0152 (SAR_RS00760)	0,00	0,97	0,29	0,97	0,00	0,68	0,00	0,97	0,29
>0129	capF	SAUR0153 (SAR_RS00765)	0,00	1,80	0,99	1,80	0,00	1,71	0,00	1,80	0,99
>0130	capG	SAUR0154 (SAR_RS00770)	0,00	1,07	0,44	1,07	0,18	0,80	0,98	0,27	0,71
>0131	capH	(N/A)	0,09	>=5,00	0,74	>=5,00	0,00	>=5,00	>=5,00	0,09	>=5,00
>0132	capI	(N/A)	0,00	>=5,00	0,64	>=5,00	0,00	>=5,00	>=5,00	0,00	>=5,00
>0133	capJ	(N/A)	0,00	>=5,00	0,00	>=5,00	0,00	>=5,00	>=5,00	0,26	>=5,00
>0134	capK	(N/A)	0,00	>=5,00	0,31	>=5,00	0,00	>=5,00	>=5,00	0,08	>=5,00
>0135	capL	SAUR0159 (SAR_RS00795)	0,08	1,16	0,41	1,08	0,00	1,08	1,08	0,00	1,08
>0136	capM	SAUR0160 (SAR_RS00800)	0,18	0,54	0,36	0,36	0,00	0,54	0,36	0,00	0,54
>0137	capN	SAUR0161 (SAR_RS00805)	0,00	1,91	0,68	1,80	0,11	1,35	1,80	0,11	1,35
>0138	capO	SAUR0162 (SAR_RS00810)	0,00	1,11	0,32	1,03	0,08	1,03	1,03	0,08	1,03
>0139	capP	SAUR0163 (SAR_RS00815)	0,00	1,11	0,51	1,11	0,00	1,02	1,19	0,09	1,11
>0140	isdI	SAUR0164 (SAR_RS00820)	0,00	1,22	0,00	0,92	0,31	0,92	0,92	0,31	0,92
>0141	ybaN	SAUR0165 (SAR_RS00825)	0,00	0,52	0,00	0,52	0,00	0,52	0,52	0,00	0,52
>0142	aldA1	SAUR0166 (SAR_RS00830)	0,00	1,68	0,60	1,68	0,00	1,34	1,68	0,00	1,34
>0143	czcD	SAUR0167 (SAR_RS00835)	0,00	1,46	0,83	1,35	0,10	1,77	1,35	0,10	1,77
>0144	Q5HJK0=srpF	SAUR0169 (SAR_RS00845)	0,00	1,56	0,00	1,56	0,00	1,56	1,56	0,00	1,56
>0145	tauB	SAUR0170 (SAR_RS00850)	0,13	4,05	0,13	3,91	0,00	3,91	3,91	0,00	3,91
>0146	tauA	SAUR0171 (SAR_RS00855)	0,10	2,56	1,23	2,46	0,00	2,56	2,46	0,00	2,56
>0147	tauC	SAUR0172 (SAR_RS00860)	0,00	1,57	1,05	1,57	0,00	1,31	1,57	0,00	1,31
>0148	Q5HJJ8	SAUR0173 (SAR_RS00865)	0,00	3,68	1,07	3,68	0,00	3,59	3,68	0,00	3,59
>0149	lmrP	SAUR0176 (SAR_RS00880)	0,16	>=5,00	0,48	>=5,00	0,08	>=5,00	>=5,00	0,00	>=5,00
>0150	ausA	SAUR0177 (SAR_RS00885)	0,00	0,64	0,39	0,65	0,01	0,60	0,64	0,00	0,59
>0151	ausB	SAUR0178 (SAR_RS00890)	0,00	2,17	2,17	2,17	0,00	1,55	2,17	0,00	1,55
>0152	Q5HJJ2=yagU=DUF1440/5080	SAUR0179 (SAR_RS00895)	0,00	3,43	3,43	3,43	0,00	>=5,00	3,43	0,00	>=5,00
>0153	argB	SAUR0180 (SAR_RS00900)	0,00	>=5,00	1,69	>=5,00	0,00	>=5,00	>=5,00	0,00	>=5,00
>0154	argJ	SAUR0181 (SAR_RS00905)	0,00	>=5,00	0,81	3,46	>=5,00	3,46	3,46	>=5,00	3,46
>0155	argC	SAUR0182 (SAR_RS00910)	0,10	1,94	0,48	2,13	0,10	1,94	2,13	0,10	1,94
>0156	rocD1	SAUR0183 (SAR_RS00915)	0,00	1,01	1,43	1,01	0,00	2,11	1,01	0,00	2,11
>0157	brnQ1	SAUR0184 (SAR_RS00920)	0,07	1,25	0,22	1,33	0,00	1,18	1,33	0,00	1,18
>0158	yecD=ywoC	SAUR0185 (SAR_RS00925)	>=5,00	>=5,00	>=5,00	>=5,00	0,36	>=5,00	>=5,00	>=5,00	>=5,00
>0159	ipdC	SAUR0186 (SAR_RS00930)	0,00	2,07	0,67	2,01	0,06	2,25	2,07	0,06	2,32
>0160	glcA=ptsG	SAUR0187 (SAR_RS00935)	0,00	2,69	0,29	2,64	0,05	2,74	2,64	0,05	2,74
>0161	DUF0871	SAUR0188 (SAR_RS00940)	0,19	0,47	0,47	0,66	0,00	0,57	0,66	0,00	0,57
>0162	murQ	SAUR0189 (SAR_RS00945)	0,00	0,55	1,66	0,44	0,11	1,66	0,55	0,22	1,78
>0163	Q5HJI0	SAUR0190 (SAR_RS00950)	0,00	1,03	0,96	1,03	0,00	1,31	1,03	0,00	1,31
>0164	rpiRB	SAUR0191 (SAR_RS00955)	0,11	0,34	1,37	0,23	0,00	1,03	0,23	0,00	1,03
>0165	hsdR	SAUR0193 (SAR_RS00965)	0,11	1,18	0,65	1,25	0,11	1,15	1,25	0,11	1,15
>0166	Q9RL82	(N/A)	0,00	1,52	>=5,00	1,52	0,00	>=5,00	1,52	0,00	>=5,00
>0167	oppF2	SAUR0195 (SAR_RS00975)	0,06	1,32	0,50	1,32	0,06	1,38	1,26	0,00	1,32
>0168	oppB2=gsiC	(N/A)	0,00	0,61	0,38	0,61	0,00	0,84	0,61	0,00	0,84
>0169	oppC2	SAUR0197 (SAR_RS00985)	0,00	0,60	0,43	0,69	0,09	0,95	0,77	0,17	1,03
>0170	oppA2	SAUR0198 (SAR_RS00990)	0,06	1,13	0,51	1,07	0,11	1,18	1,07	0,11	1,18
>0171	ggt	SAUR0199 (SAR_RS00995)	0,00	>=5,00	>=5,00	>=5,00	0,00	>=5,00	>=5,00	0,00	>=5,00
>0172	acpD	SAUR0200 (SAR_RS01000)	0,00	2,39	0,32	2,39	0,00	2,23	2,39	0,00	2,23
>0173	Q5HJG7=lytM23	SAUR0201 (SAR_RS01005)	>=5,00	>=5,00	>=5,00	1,55	0,00	1,73	1,55	0,00	1,73
>0174	malK=ugpC	SAUR0202 (SAR_RS01010)	0,09	0,64	0,27	0,73	0,00	0,55	0,73	0,00	0,55
>0175	malE	SAUR0203 (SAR_RS01015)	0,08	0,86	0,31	0,94	0,00	0,71	0,94	0,00	0,71
>0176	malC	SAUR0204 (SAR_RS01020)	0,00	1,42	0,00	1,42	0,00	1,42	1,50	0,08	1,50
>0177	malD	SAUR0205 (SAR_RS01025)	0,00	0,83	0,36	0,83	0,00	0,95	0,83	0,00	0,95
>0178	yrbE	SAUR0206 (SAR_RS01030)	0,00	1,49	0,84	1,49	0,00	1,39	1,49	0,00	1,39
>0179	mviM	SAUR0207 (SAR_RS01035)	0,38	2,98	2,69	3,27	0,10	2,31	3,17	0,00	2,21
>0180	iolE	SAUR0208 (SAR_RS01040)	0,00	1,24	0,62	1,14	0,10	0,93	1,14	0,10	0,93
>0181	Q7A1X1	SAUR0209 (SAR_RS01045)	0,20	0,61	0,20	0,40	0,00	0,40	0,61	0,20	0,61
>0182	uhpT	SAUR0210 (SAR_RS01050)	0,07	1,30	0,65	1,38	0,14	1,38	1,30	0,07	1,30
>0183	yesN	SAUR0211 (SAR_RS01055)	0,00	1,32	0,40	1,32	0,00	1,19	1,32	0,00	1,19
>0184	yesM	SAUR0212 (SAR_RS01060)	0,06	1,28	0,83	1,41	0,06	1,16	1,41	0,06	1,16

Number/ Position in the figures	Gene ID	ID in PubMLST cg scheme	ED133 vs. swan isolate 15V8707	X22 vs. swan isolate 15V8707	CC522 goat isolate 17CS1042 vs. swan isolate 15V8707	ED133 vs. duck isolate V315	X22 vs. duck isolate V315	CC522 goat isolate 17CS1042 vs. duck isolate V315	ED133 vs. duck isolate V482	X22 vs. duck isolate V482	CC522 goat isolate 17CS1042 vs. duck isolate V482
>0185	hptA	SAUR0213 (SAR_RS01065)	0,10	1,65	1,24	1,86	0,31	1,55	1,65	0,10	1,34
>0186	pflB	SAUR0214 (SAR_RS01070)	0,00	0,49	0,44	0,53	0,04	0,40	0,53	0,04	0,40
>0187	pflA	SAUR0215 (SAR_RS01075)	0,00	0,00	0,26	0,00	0,00	0,26	0,00	0,00	0,26
>0188	Q2YV51	SAUR0216 (SAR_RS01080)	0,00	>=5,00	0,00	>=5,00	0,00	>=5,00	>=5,00	0,00	>=5,00
>0189	glpQ1	SAUR0217 (SAR_RS01085)	0,00	0,91	0,68	0,85	0,06	1,08	0,85	0,06	1,08
>0190	Q2YV49	SAUR0218 (SAR_RS01090)	0,00	1,16	1,16	1,16	0,00	1,74	1,16	0,00	1,74
>0191	coa	(N/A)	3,87	>=5,00	>=5,00	>=5,00	0,19	>=5,00	>=5,00	>=5,00	>=5,00
>0192	fadA	SAUR0222 (SAR_RS01110)	0,00	0,84	1,01	0,84	0,00	1,27	0,84	0,00	1,27
>0193	fadB	SAUR0223 (SAR_RS01115)	0,00	1,02	1,06	1,02	0,00	0,93	1,02	0,00	0,93
>0194	fadD	SAUR0224 (SAR_RS01120)	0,08	1,40	0,66	1,40	0,08	0,99	1,40	0,08	0,99
>0195	fadE	SAUR0225 (SAR_RS01125)	0,00	>=5,00	>=5,00	>=5,00	0,00	1,86	>=5,00	0,00	1,86
>0196	fadX	SAUR0226 (SAR_RS01130)	1,01	0,95	0,63	1,96	0,00	1,08	1,96	0,00	1,08
>0197	prsW=prsS	(N/A)	0,09	>=5,00	0,79	>=5,00	0,00	>=5,00	>=5,00	0,00	>=5,00
>0198	nikA	SAUR0229 (SAR_RS01145)	0,07	1,42	0,61	1,56	0,27	1,42	1,42	0,14	1,29
>0199	DUF0488	SAUR0230 (SAR_RS01150)	0,00	1,40	1,96	1,40	0,00	1,68	1,40	0,00	1,68
>0200	Q5HJD9	SAUR0231 (SAR_RS01155)	0,00	>=5,00	>=5,00	>=5,00	0,00	2,30	>=5,00	0,00	2,30
>0201	hmp	SAUR0232 (SAR_RS01160)	0,00	2,62	0,17	2,53	0,09	2,71	2,53	0,09	2,71
>0202	lctE	SAUR0234 (SAR_RS01170)	0,10	0,21	0,10	0,31	0,00	0,31	0,31	0,00	0,31
>0203	ptsI=ptsIPIBC	SAUR0236 (SAR_RS01180)	0,07	0,65	0,26	0,78	0,07	0,98	0,78	0,07	0,98
>0204	rihA	SAUR0237 (SAR_RS01185)	0,11	1,39	0,53	1,28	0,00	0,96	1,28	0,00	0,96
>0205	bglG=licR	SAUR2967	0,00	0,95	0,86	0,95	0,10	0,86	0,91	0,05	0,81
>0206	Q5HJD2	(N/A)	0,00	0,64	1,07	0,64	0,00	0,43	0,64	0,00	0,43
>0207	Q5HJD1=gatB1	SAUR2968	0,00	0,72	0,00	0,72	0,00	0,72	0,72	0,36	0,72
>0208	Q2G2C8=gatC1	SAUR2969	0,00	0,71	0,40	0,79	0,08	1,03	0,71	0,00	0,95
>0209	gutB	SAUR2970 (gutB)	0,00	1,33	0,47	1,33	0,00	1,33	1,33	0,00	1,33
>0210	Q5HJC7	SAUR2971	0,00	2,04	1,36	2,04	0,00	0,68	2,04	0,00	0,68
>0211	Q5HJC6	SAUR2972	0,00	1,05	1,63	0,86	0,19	1,92	0,86	0,19	1,92
>0212	tarI1=ispD1	(N/A)	0,00	0,84	2,79	0,00	0,84	2,79	0,00	0,84	2,79
>0213	tarJ1	SAUR2973	0,00	0,78	2,14	0,00	0,78	2,14	0,00	0,78	2,14
>0214	tarL1	SAUR2974	0,06	2,30	1,24	0,06	2,30	1,24	0,06	2,30	1,24
>0215	tarF	SAUR2975	0,17	1,62	0,26	0,00	1,45	0,09	0,00	1,45	0,09
>0216	tarJ2=gutb	SAUR2976	0,00	0,68	0,00	0,00	0,68	0,00	0,00	0,68	0,00
>0217	tarL=tafB/F	SAUR2977	0,00	0,41	0,06	0,06	0,47	0,12	0,06	0,47	0,12
>0218	tarS	SAUR2940	>=5,00	1,16	0,17	>=5,00	1,22	0,23	>=5,00	1,16	0,17
>0219	lytS	SAUR0256 (SAR_RS01280)	0,00	0,40	>=5,00	0,00	0,40	>=5,00	0,00	0,40	>=5,00
>0220	lytR	SAUR0257 (SAR_RS01285)	0,00	0,94	0,00	0,00	0,94	0,00	0,00	0,94	0,00
>0221	lrgA	SAUR0258 (SAR_RS01290)	0,00	0,00	0,00	0,00	0,00	0,00	0,00	0,00	0,00
>0222	lrgB	SAUR0259 (SAR_RS01295)	0,00	0,57	0,14	0,00	0,57	0,14	0,00	0,57	0,14
>0223	Q5HJB2	SAUR0261 (SAR_RS01305)	0,00	1,13	0,14	0,00	1,13	0,14	0,00	1,13	0,14
>0224	Q1YAD5=ptsG3	SAUR0262 (SAR_RS01310)	0,13	>=5,00	2,02	0,13	>=5,00	2,02	0,13	>=5,00	2,02
>0225	bglA	SAUR0263 (SAR_RS01315)	0,07	1,60	0,97	0,00	1,53	0,90	0,00	1,53	0,90
>0226	rbsK	SAUR0267 (SAR_RS01335)	0,00	1,09	0,66	0,00	1,09	0,66	0,00	1,09	0,66
>0227	rbsD	SAUR0268 (SAR_RS01340)	0,00	1,73	1,48	0,00	1,73	1,48	0,00	1,73	1,48
>0228	rbsU	SAUR0269 (SAR_RS01345)	0,00	2,61	1,47	0,00	2,61	1,47	0,00	2,61	1,47
>0229	rbsR=degA	SAUR0270 (SAR_RS01350)	0,00	2,00	1,50	0,00	2,00	1,50	0,00	2,00	1,50
>0230	yusP	SAUR0273 (SAR_RS01365)	0,00	1,23	0,80	>=5,00	>=5,00	>=5,00	0,00	1,23	0,80
>0231	yxeI	SAUR0274 (SAR_RS01370)	0,00	0,81	0,50	0,00	0,81	0,50	0,00	0,81	0,50
>0232	lytM	SAUR0275 (SAR_RS01375)	0,00	1,47	0,21	0,00	1,47	0,21	0,00	1,47	0,21
>0233	Q5HJ98=ybhF	SAUR0276 (SAR_RS01380)	0,00	0,91	0,15	0,00	0,91	0,15	0,00	0,91	0,15
>0234	Q5HJ97	SAUR0277 (SAR_RS01385)	0,11	0,76	>=5,00	0,00	0,65	>=5,00	0,11	0,76	>=5,00
>0235	Q5HJ96	SAUR0278 (SAR_RS01390)	0,00	1,11	1,03	0,00	1,11	1,03	0,00	1,11	1,03
>0236	Q5HJ95	SAUR0279 (SAR_RS01395)	0,13	0,66	0,66	0,20	0,72	0,72	0,26	0,79	0,79
>0237	ssaA	SAUR0280 (SAR_RS01400)	0,00	1,00	0,78	0,00	1,00	0,78	0,00	1,00	0,78
>0238	esxA	SAUR0281 (SAR_RS01405)	0,00	0,34	0,00	0,00	0,34	0,00	0,00	0,34	0,00
>0239	esaA	SAUR0282 (SAR_RS01410)	0,03	1,06	0,86	0,03	1,06	0,86	0,03	1,06	0,86
>0240	essA	SAUR2978	0,00	0,44	0,00	0,00	0,44	0,00	0,00	0,44	0,00
>0241	esaB	SAUR0284 (SAR_RS01420)	0,00	1,23	0,00	0,00	1,23	0,00	0,00	1,23	0,00
>0242	essB	SAUR0285 (SAR_RS01425)	0,00	0,67	0,60	0,07	0,75	0,67	0,00	0,67	0,60
>0243	essC	SAUR0286 (SAR_RS01430)	0,05	0,92	0,79	0,00	0,88	0,74	0,00	0,88	0,74
>0244	esxB	(N/A)	0,00	0,63	0,63	0,00	0,63	0,63	0,00	0,63	0,63
>0245	Q1Y4S0=DUF4467	SAUR2979	>=5,00	>=5,00	>=5,00	0,00	1,47	>=5,00	0,00	1,47	>=5,00
>0246	Q2G173=DUF4064	(N/A)	0,00	1,25	0,50	0,00	1,25	0,50	0,00	1,25	0,50
>0247	focA-L1=nirC	SAUR0301 (SAR_RS01505)	0,00	1,82	2,18	0,12	1,94	2,30	0,00	1,82	2,18
>0248	brnQ2	SAUR0302 (SAR_RS01510)	0,08	>=5,00	1,30	0,23	>=5,00	1,45	0,23	>=5,00	1,45
>0249	Q99WS0=sapS	SAUR0303 (SAR_RS01515)	0,00	0,90	0,34	0,00	0,90	0,34	0,00	0,90	0,34
>0250	Q2G168	SAUR0305 (SAR_RS01525)	1,33	1,90	2,10	0,00	0,57	0,76	0,00	0,57	0,76
>0251	A5IPH6	SAUR0306 (SAR_RS01530)	0,00	0,74	1,03	0,00	0,74	1,03	0,00	0,74	1,03
>0252	pfoR	SAUR0307 (SAR_RS01535)	0,00	1,45	1,45	0,00	1,45	1,45	0,00	1,45	1,45
>0253	Q5HJ57	SAUR0308 (SAR_RS01540)	0,09	0,27	>=5,00	0,00	0,18	>=5,00	0,00	0,18	>=5,00
>0254	psuG	SAUR0309 (SAR_RS01545)	0,00	1,84	>=5,00	0,00	1,84	>=5,00	0,00	1,84	>=5,00
>0255	nupC2	SAUR0310 (SAR_RS01550)	0,00	1,15	>=5,00	0,00	1,15	>=5,00	0,00	1,15	>=5,00
>0256	nanT	SAUR0311 (SAR_RS01555)	0,00	3,39	1,50	0,07	3,46	1,57	0,00	3,39	1,50
>0257	nanA	SAUR0312 (SAR_RS01560)	0,00	1,47	1,02	0,11	1,59	1,13	0,11	1,59	1,13
>0258	nanK	SAUR0313 (SAR_RS01565)	0,00	1,51	0,35	0,00	1,51	0,35	0,00	1,51	0,35
>0259	nanR	SAUR0314 (SAR_RS01570)	0,00	0,87	0,50	0,00	0,87	0,50	0,00	0,87	0,50
>0260	nanE	SAUR0315 (SAR_RS01575)	0,00	1,05	0,60	0,00	1,05	0,60	0,15	1,20	0,75
>0261	Q5HJ49	SAUR0316 (SAR_RS01580)	0,08	0,53	0,99	0,08	0,53	0,99	0,08	0,53	0,99
>0262	lip2	SAUR0317 (SAR_RS01585)	>=5,00	>=5,00	>=5,00	>=5,00	>=5,00	0,00	>=5,00	>=5,00	0,00
>0263	A5IPQ2	SAUR0318 (SAR_RS01590)	0,00	1,81	>=5,00	0,00	1,81	>=5,00	0,00	1,81	>=5,00
>0264	Q6GJZ4=namA	SAUR0319 (SAR_RS01595)	0,08	1,58	>=5,00	0,08	1,58	>=5,00	0,08	1,58	>=5,00
>0265	limB1	SAUR0321 (SAR_RS01605)	0,30	1,30	2,29	0,10	1,10	2,09	0,10	1,10	2,09
>0266	gcvH1	SAUR0322 (SAR_RS01610)	0,00	0,60	1,20	0,00	0,60	1,20	0,00	0,60	1,20
>0267	UPF0189=ymdB	SAUR0323 (SAR_RS01615)	0,12	1,50	1,75	0,12	1,50	1,75	0,12	1,50	1,75
>0268	sir2	SAUR0324 (SAR_RS01620)	0,11	2,65	0,95	0,21	2,75	1,06	0,11	2,65	0,95
>0269	lplA1	SAUR0325 (SAR_RS01625)	0,00	3,03	1,27	0,00	3,03	1,27	0,00	3,03	1,27
>0270	ulaA	SAUR0327 (SAR_RS01635)	0,22	1,56	0,89	0,22	1,56	0,89	0,15	1,48	0,82
>0271	Q5HIW4	SAUR0328 (SAR_RS01640)	0,00	0,35	0,00	0,00	0,35	0,00	0,00	0,35	0,00
>0272	Q5HIW3	SAUR0329 (SAR_RS01645)	0,00	1,35	0,90	0,23	1,58	1,13	0,23	1,58	1,13
>0273	A8YZF7=licR2	SAUR0330 (SAR_RS01650)	0,05	2,20	1,18	0,00	2,15	1,12	0,00	2,15	1,12
>0274	mepR	SAUR0331 (SAR_RS01655)	0,00	1,43	0,48	0,00	1,43	0,48	0,00	1,43	0,48
>0275	mepA	SAUR0332 (SAR_RS01660)	0,00	1,77	0,81	0,00	1,77	0,81	0,00	1,77	0,81
>0276	mepB	(N/A)	0,00	2,24	1,79	0,00	2,24	1,79	2,69	3,14	2,69
>0277	glpT	SAUR0334 (SAR_RS01670)	0,00	0,52	0,37	0,07	0,59	0,44	0,07	0,59	0,44
>0278	mhqA-1	SAUR0335 (SAR_RS01675)	0,00	1,73	>=5,00	0,00	1,73	>=5,00	>=5,00	>=5,00	0,54
>0279	ssuD=luxA	SAUR0336 (SAR_RS01680)	0,00	2,07	1,22	0,00	2,07	1,22	0,00	2,07	1,22
>0280	ssuE	SAUR0337 (SAR_RS01685)	0,00	2,12	1,06	0,00	2,12	1,06	0,00	2,12	1,06
>0281	yeiH	SAUR0338 (SAR_RS01690)	0,00	1,31	0,50	0,00	1,31	0,50	0,00	1,31	0,50
>0282	rimL=ydaF	SAUR0340 (SAR_RS01700)	0,18	4,58	1,83	0,18	4,95	1,83	0,00	4,76	1,65
>0283	fepA	SAUR0341 (SAR_RS01705)	>=5,00	0,58	1,17	>=5,00	0,47	1,05	>=5,00	0,47	1,05
>0284	fepB	SAUR0342 (SAR_RS01710)	0,08	0,41	0,65	0,16	0,49	0,73	0,16	0,49	0,73
>0285	fepC	SAUR0343 (SAR_RS01715)	0,06	1,75	0,99	0,12	1,81	1,05	0,06	1,75	0,99
>0286	tatC	SAUR0344 (SAR_RS01720)	0,00	3,85	2,85	0,00	3,85	2,85	0,00	3,85	2,85
>0287	tatA	SAUR0345 (SAR_RS01725)	0,00	0,44	>=5,00	0,00	0,44	>=5,00	0,00	0,44	>=5,00
>0288	DUF1398	SAUR0346 (SAR_RS01730)	0,26	>=5,00	0,77	0,26	>=5,00	0,77	0,26	>=5,00	0,77
>0289	Q5HIU5	SAUR0347 (SAR_RS01735)	0,00	2,94	>=5,00	0,00	2,94	>=5,00	0,00	2,94	>=5,00
>0290	DUF3169	SAUR0348 (SAR_RS01740)	>=5,00	>=5,00	>=5,00	0,28	>=5,00	>=5,00	0,28	>=5,00	>=5,00
>0291	Q5HIU3	SAUR0349 (SAR_RS01745)	0,00	3,32	2,61	0,00	3,32	2,61	0,00	3,32	2,61
>0292	Q5HIU2	SAUR0350 (SAR_RS01750)	0,00	1,27	0,48	0,00	1,27	0,48	0,00	1,27	0,48
>0293	ltrA-L1	SAUR2980	0,00	1,66	0,35	0,79	2,45	1,14	0,00	1,66	0,35
>0294	thlA=yqil	SAUR0353 (SAR_RS01765)	0,00	1,61	0,85	0,00	1,61	0,85	0,00	1,61	0,85
>0295	mdh	SAUR2981	0,00	1,06	0,27	0,00	1,06	0,27	0,13	1,20	0,40
>0296	metE	SAUR0355 (SAR_RS01775)	0,04	1,35	0,58	0,00	1,30	0,54	0,04	1,35	0,58
>0297	metF	SAUR0356 (SAR_RS01780)	0,11	1,09	0,87	0,05	1,03	0,81	0,05	1,03	0,81
>0298	metC	SAUR0357 (SAR_RS01785)	0,09	1,21	0,69	0,00	1,12	0,60	0,00	1,12	0,60
>0299	metI	SAUR0358 (SAR_RS01790)	0,00	>=5,00	>=5,00	0,09	>=5,00	>=5,00	0,09	>=5,00	>=5,00
>0300	spo0J=parB	SAUR0359 (SAR_RS01795)	0,00	2,72	0,59	0,00	2,72	0,59	0,00	2,72	0,59
>0301	ykuT	SAUR0360 (SAR_RS01800)	0,00	>=5,00	0,23	>=5,00	>=5,00	>=5,00	>=5,00	>=5,00	>=5,00
>0302	yyzM	SAUR0361 (SAR_RS01805)	0,00	>=5,00	0,00	0,00	>=5,00	0,00	0,00	>=5,00	0,00
>0303	yyaF=engD=ychF	SAUR0362 (SAR_RS01810)	0,00	>=5,00	0,64	0,00	>=5,00	0,64	0,00	>=5,00	0,64
>0304	Q5HIT0	SAUR0363 (SAR_RS01815)	0,00	>=5,00	0,00	0,00	>=5,00	0,00	0,00	>=5,00	0,00
>0305	rpsF	(N/A)	0,00	0,36	0,00	0,00	0,36	0,00	0,00	0,36	0,00
>0306	ssbP=Q6G7V6	SAUR2649 (SAR_RS13245)	0,00	0,40	0,20	0,00	0,40	0,20	0,00	0,40	0,20
>0307	rpsR	SAUR0366 (SAR_RS01830)	0,00	0,00	0,00	0,00	0,00	0,00	0,00	0,00	0,00
>0308	setC	(N/A)	0,33	2,78	>=5,00	0,16	2,61	>=5,00	0,16	2,61	>=5,00
>0309	Q2G106	SAUR0392 (SAR_RS01960)	0,00	1,63	0,00	0,00	1,63	0,00	0,00	1,63	0,00
>0310	Q1Y9M1	SAUR0393 (SAR_RS01965)	0,00	0,70	0,00	0,00	0,70	0,00	0,00	0,70	0,00
>0311	Q5HIS2	SAUR0394 (SAR_RS01970)	0,00	0,76	0,38	0,00	0,76	0,38	0,00	0,76	0,38
>0312	Q5HIS1	SAUR0395 (SAR_RS01975)	0,00	>=5,00	0,39	0,00	>=5,00	0,39	0,00	>=5,00	0,39
>0313	Q7A7J4	SAUR0397 (SAR_RS01985)	0,00	2,58	1,20	0,00	2,58	1,20	0,00	2,58	1,20
>0314	Q5HIR9	SAUR0398 (SAR_RS01990)	0,26	1,30	0,00	0,26	1,30	0,00	0,26	1,30	0,00
>0315	A5IPV8	SAUR0399 (SAR_RS01995)	0,00	0,96	0,96	0,00	0,96	0,96	0,00	0,96	0,96
>0316	Q6GJR9-short	SAUR0488 (SAR_RS02440)	0,00	>=5,00	4,42	0,00	>=5,00	4,42	0,34	>=5,00	4,76
>0317	ahpF	SAUR0401 (SAR_RS02005)	0,00	1,38	0,26	0,00	1,38	0,26	0,00	1,38	0,26
>0318	ahpC	SAUR0402 (SAR_RS02010)	0,18	0,53	0,70	0,18	0,53	0,70	0,35	0,70	0,88
>0319	nfrA	SAUR0403 (SAR_RS02015)	0,00	0,66	0,66	0,00	0,66	0,66	0,13	0,79	0,79
>0320	tcyP	SAUR0404 (SAR_RS02020)	0,14	1,94	1,51	0,14	1,94	1,51	0,07	1,87	1,44
>0321	Q5HIR2	SAUR0406 (SAR_RS02030)	0,10	2,61	1,67	0,10	2,61	1,67	0,10	2,61	1,67
>0322	Q5HIR1	SAUR0407 (SAR_RS02035)	0,00	1,36	0,75	0,00	1,36	0,75	0,00	1,36	0,75
>0323	yflT	SAUR0408 (SAR_RS02040)	0,00	>=5,00	>=5,00	0,00	>=5,00	>=5,00	0,00	>=5,00	>=5,00
>0324	xpt	SAUR0409 (SAR_RS02045)	0,17	1,04	0,86	0,17	1,04	0,86	0,17	1,04	0,86
>0325	pbuX	SAUR0410 (SAR_RS02050)	0,00	0,79	0,63	0,00	0,79	0,63	0,00	0,79	0,63
>0326	guaB	SAUR0411 (SAR_RS02055)	0,00	1,36	0,48	0,00	1,36	0,48	0,00	1,36	0,48
>0327	guaA	SAUR0412 (SAR_RS02060)	0,00	2,59	1,10	0,06	2,66	1,17	0,00	2,59	1,10
>0328	Q2YVN4	(N/A)	0,00	>=5,00	>=5,00	0,00	>=5,00	>=5,00	0,00	>=5,00	>=5,00
>0329	Q5HIQ1=DUF1304	SAUR0418 (SAR_RS02090)	0,28	>=5,00	4,16	0,28	>=5,00	4,16	0,28	>=5,00	4,16
>0330	Q2G0Y0	SAUR0419 (SAR_RS02095)	0,00	1,65	1,18	0,00	1,65	1,18	0,00	1,65	1,18
>0331	ssl01=set6	SAUR0420 (SAR_RS02100)	0,00	>=5,00	>=5,00	0,00	>=5,00	>=5,00	0,00	>=5,00	>=5,00
>0332	ssl02=set7	SAUR0421 (SAR_RS02105)	0,00	0,72	0,14	0,00	0,72	0,14	0,00	0,72	0,14

Number/ Position in the figures	Gene ID	ID in PubMLST cg scheme	ED133 vs. swan isolate 15V8707	X22 vs. swan isolate 15V8707	CC522 goat isolate 17CS1042 vs. swan isolate 15V8707	ED133 vs. duck isolate V315	X22 vs. duck isolate V315	CC522 goat isolate 17CS1042 vs. duck isolate V315	ED133 vs. duck isolate V482	X22 vs. duck isolate V482	CC522 goat isolate 17CS1042 vs. duck isolate V482
>0333	ssl03=set8	(N/A)	0,00	>=5,00	>=5,00	>=5,00	>=5,00	0,00	0,00	>=5,00	>=5,00
>0334	ssl04=set9	(N/A)	0,00	>=5,00	>=5,00	0,28	>=5,00	>=5,00	0,00	>=5,00	>=5,00
>0335	ssl05=set3	(N/A)	0,00	>=5,00	>=5,00	0,00	>=5,00	>=5,00	0,00	>=5,00	>=5,00
>0336	ssl06=set21	(N/A)	0,00	>=5,00	>=5,00	0,00	>=5,00	>=5,00	0,00	>=5,00	>=5,00
>0337	ssl07=set1	SAUR0426 (SAR_RS02130)	0,14	>=5,00	>=5,00	0,14	>=5,00	>=5,00	0,14	>=5,00	>=5,00
>0338	ssl08=set12	(N/A)	0,14	1,86	>=5,00	0,00	1,72	>=5,00	0,14	1,86	>=5,00
>0339	ssl09=set5	SAUR0428 (SAR_RS02140)	0,00	>=5,00	>=5,00	0,00	>=5,00	>=5,00	0,00	>=5,00	>=5,00
>0340	ssl10=set4	SAUR0428 (SAR_RS02140)	0,00	>=5,00	>=5,00	0,00	>=5,00	>=5,00	0,00	>=5,00	>=5,00
>0341	hsdM-ssl	(N/A)	0,06	4,11	3,73	0,26	4,17	3,92	0,39	4,30	4,05
>0342	hsdS-ssl	(N/A)	0,00	>=5,00	>=5,00	0,08	>=5,00	>=5,00	0,00	>=5,00	>=5,00
>0343	ssl11=set2	(N/A)	0,00	>=5,00	>=5,00	0,00	>=5,00	>=5,00	0,00	>=5,00	>=5,00
>0344	Q5HIM7	SAUR0445 (SAR_RS02225)	0,00	>=5,00	>=5,00	0,00	>=5,00	>=5,00	0,00	>=5,00	>=5,00
>0345	cobW1	SAUR0446 (SAR_RS02230)	0,08	3,66	0,42	0,00	3,57	0,33	0,00	3,57	0,33
>0346	psmA4	(N/A)	0,00	0,00	0,00	0,00	0,00	0,00	0,00	0,00	0,00
>0347	psmA3	(N/A)	0,00	0,00	0,00	0,00	0,00	0,00	0,00	0,00	0,00
>0348	psmA2	(N/A)	0,00	0,00	0,00	0,00	0,00	0,00	0,00	0,00	0,00
>0349	psmA1	(N/A)	0,00	0,00	0,00	0,00	0,00	0,00	0,00	0,00	0,00
>0350	mpsA=nuoL	SAUR0448 (SAR_RS02240)	0,00	2,83	1,21	0,00	2,83	1,21	0,00	2,83	1,21
>0351	mpsB=ybcC	SAUR0449 (SAR_RS02245)	0,04	2,44	2,18	0,04	2,44	2,18	0,04	2,44	2,18
>0352	mpsC=ybcI	SAUR0450 (SAR_RS02250)	0,00	1,65	0,00	0,00	1,65	0,00	0,00	1,65	0,00
>0353	Q5HIM2	(N/A)	0,00	2,01	0,86	0,00	2,01	0,86	0,00	2,01	0,86
>0355	Q5HIM1	SAUR0452 (SAR_RS02260)	0,15	1,63	>=5,00	0,15	1,63	>=5,00	0,15	1,63	>=5,00
>0356	Q5HIM0=est1	SAUR0453 (SAR_RS02265)	0,14	1,50	0,00	0,14	1,50	0,00	0,14	1,50	0,00
>0357	Q5HIL8=galE	SAUR0126 (SAR_RS00630)	0,07	1,42	0,52	0,00	1,35	0,45	0,00	1,35	0,45
>0358	mccA-var906	SAUR0456 (SAR_RS02280)	0,00	>=5,00	3,41	0,11	>=5,00	3,52	0,11	>=5,00	3,52
>0359	mccB	SAUR0457 (SAR_RS02285)	0,00	1,40	0,17	0,00	1,40	0,17	0,09	1,49	0,26
>0360	metN2	SAUR0458 (SAR_RS02290)	0,00	1,17	0,49	0,00	1,17	0,49	0,00	1,17	0,49
>0361	metP2	SAUR0459 (SAR_RS02295)	0,00	1,06	0,61	0,00	1,06	0,61	0,00	1,06	0,61
>0362	metQ2=spn=gmpC	SAUR0460 (SAR_RS02300)	0,00	2,02	0,95	0,00	2,02	0,95	0,12	2,14	1,07
>0363	aaa=sle1	(N/A)	0,00	1,79	1,59	0,00	1,79	1,59	0,10	1,88	1,69
>0364	Q5HIL1	SAUR0462 (SAR_RS02310)	0,00	0,74	1,11	0,00	0,74	1,11	0,00	0,74	1,11
>0365	nudG	SAUR0463 (SAR_RS02315)	0,00	0,76	0,25	0,00	0,76	0,25	0,00	0,76	0,25
>0366	bltD	SAUR0464 (SAR_RS02320)	0,00	1,65	2,26	0,00	1,65	2,26	0,00	1,65	2,26
>0367	yibF	SAUR0465 (SAR_RS02325)	0,00	0,89	0,64	0,00	0,89	0,64	0,00	0,89	0,64
>0368	yibE	SAUR0466 (SAR_RS02330)	0,09	1,44	0,63	0,00	1,35	0,54	0,00	1,35	0,54
>0369	gltC	SAUR0467 (SAR_RS02335)	0,00	1,92	1,47	0,00	1,92	1,47	0,00	1,92	1,47
>0370	gltB	SAUR0468 (SAR_RS02340)	0,07	0,80	0,42	0,07	0,80	0,42	0,07	0,80	0,42
>0371	gltD	SAUR0469 (SAR_RS02345)	0,00	0,41	0,75	0,00	0,41	0,75	0,00	0,41	0,75
>0372	treP	SAUR0471 (SAR_RS02355)	0,07	1,12	0,77	0,07	1,12	0,77	0,07	1,12	0,77
>0373	treC	SAUR0472 (SAR_RS02360)	0,06	0,91	0,49	0,06	0,91	0,49	0,06	0,91	0,49
>0374	treR	SAUR0473 (SAR_RS02365)	0,00	1,51	0,69	0,00	1,51	0,69	0,00	1,51	0,69
>0375	Q5HIK0	SAUR0475 (SAR_RS02375)	0,19	1,71	0,00	0,19	1,71	0,00	0,19	1,71	0,00
>0376	dnaX	SAUR0476 (SAR_RS02380)	0,06	1,41	0,29	0,12	1,47	0,35	0,12	1,47	0,35
>0377	yaaK	SAUR0477 (SAR_RS02385)	0,00	0,00	0,00	0,00	0,00	0,00	0,00	0,00	0,00
>0378	recR	SAUR0478 (SAR_RS02390)	0,00	0,67	0,00	0,00	0,67	0,00	0,00	0,67	0,00
>0379	yaaO	SAUR0484 (SAR_RS02420)	0,00	1,35	0,60	0,15	1,49	0,75	0,07	1,42	0,67
>0380	tmk	SAUR0485 (SAR_RS02425)	0,00	0,65	0,49	0,00	0,65	0,49	0,00	0,65	0,49
>0381	darA	SAUR0486 (SAR_RS02430)	0,00	0,00	0,00	0,00	0,00	0,00	0,00	0,00	0,00
>0382	holB	SAUR0488 (SAR_RS02440)	0,00	0,65	0,32	0,00	0,65	0,32	0,00	0,65	0,32
>0383	yaaT=tpl	SAUR0489 (SAR_RS02445)	0,12	1,12	0,62	0,12	1,12	0,62	0,12	1,12	0,62
>0384	yabA	SAUR0490 (SAR_RS02450)	0,00	0,00	0,00	0,00	0,00	0,00	0,00	0,00	0,00
>0385	yabB	SAUR0491 (SAR_RS02455)	0,00	1,24	0,96	0,00	1,24	0,96	0,00	1,24	0,96
>0386	yazA	(N/A)	0,00	0,40	>=5,00	0,00	0,40	>=5,00	0,00	0,40	>=5,00
>0387	rsmI	SAUR0493 (SAR_RS02465)	0,00	0,48	0,60	0,00	0,48	0,60	0,00	0,48	0,60
>0388	metS=metG	SAUR0495 (SAR_RS02475)	0,05	0,96	0,86	0,00	0,91	0,81	0,00	0,91	0,81
>0389	tatP=tatD=ycfH	SAUR0496 (SAR_RS02480)	0,00	0,65	0,52	0,00	0,65	0,52	0,00	0,65	0,52
>0390	rnmV	SAUR0497 (SAR_RS02485)	0,00	>=5,00	0,56	0,00	>=5,00	0,56	0,00	>=5,00	0,56
>0391	ksgA=rsmA	(N/A)	0,00	>=5,00	0,56	0,11	>=5,00	0,67	0,11	>=5,00	0,67
>0392	veg	(N/A)	0,00	0,00	0,00	0,00	0,00	0,00	0,00	0,00	0,00
>0393	ispE	SAUR0500 (SAR_RS02500)	0,00	0,94	1,06	0,00	0,94	1,06	0,00	0,94	1,06
>0394	purR	SAUR0501 (SAR_RS02505)	0,00	0,24	0,00	0,12	0,36	0,12	0,24	0,48	0,24
>0395	yabJ=ridA	SAUR0502 (SAR_RS02510)	0,00	2,10	0,26	0,00	2,10	0,26	0,00	2,10	0,26
>0396	spoVG	SAUR0503 (SAR_RS02515)	0,00	0,00	0,00	0,00	0,00	0,00	0,00	0,00	0,00
>0397	glmU	SAUR0505 (SAR_RS02525)	0,07	0,89	0,22	0,00	0,81	0,15	0,00	0,81	0,15
>0398	prs	SAUR0506 (SAR_RS02530)	0,00	0,10	0,21	0,00	0,10	0,21	0,00	0,10	0,21
>0399	rplY	SAUR0508 (SAR_RS02540)	0,00	0,31	0,31	0,00	0,31	0,31	0,00	0,31	0,31
>0400	pth	SAUR0509 (SAR_RS02545)	0,00	1,05	0,70	0,00	1,05	0,70	0,00	1,05	0,70
>0401	mfd	SAUR0510 (SAR_RS02550)	0,00	>=5,00	0,68	0,06	>=5,00	0,74	0,06	>=5,00	0,74
>0402	yabM	SAUR0511 (SAR_RS02555)	0,00	3,34	1,31	0,00	3,34	1,31	0,00	3,34	1,31
>0403	mazG	SAUR0512 (SAR_RS02560)	0,00	0,50	0,84	0,08	0,59	0,92	0,08	0,59	0,92
>0404	hslR	SAUR0513 (SAR_RS02565)	0,38	0,38	0,38	0,38	0,38	0,38	0,38	0,38	0,38
>0405	divIC	SAUR0514 (SAR_RS02570)	0,00	0,25	0,51	0,00	0,25	0,51	0,00	0,25	0,51
>0406	yabR	SAUR0515 (SAR_RS02575)	0,00	0,25	0,25	0,00	0,25	0,25	0,00	0,25	0,25
>0407	tilS	SAUR0516 (SAR_RS02580)	0,00	1,00	0,69	0,00	1,00	0,69	0,00	1,00	0,69
>0408	hpt	SAUR0517 (SAR_RS02585)	0,19	0,37	0,56	0,19	0,37	0,56	0,19	0,37	0,56
>0409	ftsH	SAUR0518 (SAR_RS02590)	0,00	0,33	0,33	0,00	0,33	0,33	0,00	0,33	0,33
>0410	hslO	SAUR0519 (SAR_RS02595)	0,00	0,34	0,00	0,00	0,34	0,00	0,00	0,34	0,00
>0411	cysK	SAUR0520 (SAR_RS02600)	0,11	0,64	0,54	0,00	0,54	0,43	0,11	0,64	0,54
>0412	folP	SAUR0521 (SAR_RS02605)	0,00	3,98	0,75	0,12	4,10	0,87	0,00	3,98	0,75
>0413	folB	SAUR0522 (SAR_RS02610)	0,00	0,55	0,27	0,00	0,55	0,27	0,00	0,55	0,27
>0414	folK	SAUR0523 (SAR_RS02615)	0,00	1,68	>=5,00	0,00	1,68	>=5,00	0,00	1,68	>=5,00
>0415	lysS_leader	(N/A)	0,00	1,08	1,08	0,00	1,08	1,08	0,00	1,08	1,08
>0416	lysS	SAUR0525 (SAR_RS02625)	0,00	0,74	0,47	0,00	0,74	0,47	0,00	0,74	0,47
>0417	pdxR	SAUR0541 (SAR_RS02705)	>=5,00	>=5,00	>=5,00	0,00	4,19	0,58	0,07	4,27	0,65
>0418	pdxS	SAUR0542 (SAR_RS02710)	0,00	3,27	0,45	0,00	3,27	0,45	0,00	3,27	0,45
>0419	pdxT	SAUR0543 (SAR_RS02715)	0,00	0,18	0,00	0,00	0,18	0,00	0,00	0,18	0,00
>0420	nupC1	SAUR0545 (SAR_RS02725)	0,00	0,58	0,25	0,00	0,58	0,25	0,00	0,58	0,25
>0421	ctsR	SAUR0546 (SAR_RS02730)	0,00	1,30	0,43	0,00	1,30	0,43	0,00	1,30	0,43
>0422	mcsA	SAUR0547 (SAR_RS02735)	0,00	0,18	0,35	0,00	0,18	0,35	0,00	0,18	0,35
>0423	mcsB	SAUR0548 (SAR_RS02740)	0,00	0,20	0,20	0,10	0,30	0,30	0,10	0,30	0,30
>0424	clpC	SAUR0549 (SAR_RS02745)	0,08	0,57	0,49	0,00	0,49	0,41	0,00	0,49	0,41
>0425	radA	SAUR0551 (SAR_RS02755)	0,07	0,37	0,37	0,00	0,29	0,29	0,00	0,29	0,29
>0426	pilT	SAUR0552 (SAR_RS02760)	0,00	1,58	0,28	0,09	1,68	0,37	0,09	1,68	0,37
>0427	gltX	SAUR0553 (SAR_RS02765)	0,00	1,03	0,62	0,07	1,10	0,69	0,07	1,10	0,69
>0428	cysE	SAUR0554 (SAR_RS02770)	0,00	0,16	0,31	0,00	0,16	0,31	0,00	0,16	0,31
>0429	cysS	SAUR0555 (SAR_RS02775)	0,00	1,21	0,43	0,00	1,21	0,43	0,00	1,21	0,43
>0430	mrnC	SAUR0556 (SAR_RS02780)	0,00	0,49	0,00	0,00	0,49	0,00	0,00	0,49	0,00
>0431	yacO=rlmB	SAUR0557 (SAR_RS02785)	0,00	1,07	0,94	0,00	1,07	0,94	0,00	1,07	0,94
>0432	yacP	SAUR0558 (SAR_RS02790)	0,00	0,76	0,76	0,00	0,76	0,76	0,19	0,95	0,95
>0433	sigH	SAUR0559 (SAR_RS02795)	0,00	0,88	1,05	0,00	0,88	1,05	0,00	0,88	1,05
>0434	rpmG3	(N/A)	0,00	0,69	0,00	0,00	0,69	0,00	0,00	0,69	0,00
>0435	secE	(N/A)	0,00	0,00	0,00	0,00	0,00	0,00	0,00	0,00	0,00
>0436	nusG	SAUR0561 (SAR_RS02805)	0,00	0,73	0,55	0,00	0,73	0,55	0,00	0,73	0,55
>0437	rplK	SAUR0565 (SAR_RS02825)	0,24	0,47	0,24	0,00	0,24	0,00	0,00	0,24	0,00
>0438	rplA	SAUR0563 (SAR_RS02815)	0,00	0,29	0,29	0,14	0,43	0,43	0,14	0,43	0,43
>0439	rplJ	SAUR0564 (SAR_RS02820)	0,00	0,20	0,00	0,00	0,20	0,00	0,00	0,20	0,00
>0440	rplL	SAUR0565 (SAR_RS02825)	0,00	0,27	0,00	0,00	0,27	0,00	0,00	0,27	0,00
>0441	ybxB=rsmC=rlmG	SAUR0566 (SAR_RS02830)	0,00	1,15	0,33	0,00	1,15	0,33	0,00	1,15	0,33
>0442	rpoB	SAUR0567 (SAR_RS02835)	0,03	0,59	0,28	0,03	0,59	0,28	0,03	0,59	0,28
>0443	rpoC	SAUR0568 (SAR_RS02840)	0,03	0,52	0,44	0,00	0,50	0,41	0,00	0,50	0,41
>0444	ybxF	SAUR0569 (SAR_RS02845)	0,00	0,00	0,39	0,00	0,00	0,39	0,00	0,00	0,39
>0445	rpsL	SAUR0570 (SAR_RS02850)	0,00	0,00	0,00	0,00	0,00	0,00	0,00	0,00	0,00
>0446	rpsG	SAUR0571 (SAR_RS02855)	0,00	0,42	0,00	0,00	0,42	0,00	0,00	0,42	0,00
>0447	efg=fusA	SAUR0572 (SAR_RS02860)	0,00	0,29	0,05	0,00	0,29	0,05	0,05	0,34	0,10
>0448	tuf	SAUR0573 (SAR_RS02865)	0,00	0,17	0,08	0,00	0,17	0,08	0,00	0,17	0,08
>0449	yhaA=amaA	SAUR0574 (SAR_RS02870)	0,00	1,11	0,34	0,00	1,11	0,34	0,00	1,11	0,34
>0450	kbl	SAUR0575 (SAR_RS02875)	0,08	1,09	0,08	0,00	1,01	0,00	0,00	1,01	0,00
>0451	hchA	SAUR0576 (SAR_RS02880)	0,00	1,02	0,57	0,00	1,02	0,57	0,00	1,02	0,57
>0452	araB	SAUR0577 (SAR_RS02885)	0,06	1,16	1,10	0,12	1,22	1,16	0,12	1,22	1,16
>0453	Q5HIC2	SAUR0578 (SAR_RS02890)	0,00	0,62	1,04	0,00	0,62	1,04	0,10	0,72	1,14
>0454	ilvE	SAUR0579 (SAR_RS02895)	0,09	0,65	0,56	0,00	0,56	0,46	0,00	0,56	0,46
>0455	ppaX1	SAUR0580 (SAR_RS02900)	0,00	1,02	0,00	0,00	1,02	0,00	0,15	1,17	0,15
>0456	dck	SAUR0581 (SAR_RS02905)	0,00	4,07	0,00	0,00	4,07	0,00	0,15	4,22	0,15
>0457	dgk	SAUR0582 (SAR_RS02910)	0,00	0,81	0,16	0,00	0,81	0,16	0,00	0,81	0,16
>0458	tadA	SAUR0583 (SAR_RS02915)	0,21	0,21	0,00	0,21	0,21	0,00	0,21	0,21	0,00
>0459	A5IQB3	SAUR0584 (SAR_RS02920)	0,00	0,80	0,46	0,00	0,80	0,46	0,00	0,80	0,46
>0460	azo1	SAUR0585 (SAR_RS02925)	0,00	1,06	0,35	0,00	1,06	0,35	0,00	1,06	0,35
>0461	sdrC	SAUR0586 (SAR_RS02930)	0,24	>=5,00	>=5,00	0,00	>=5,00	>=5,00	0,07	>=5,00	>=5,00
>0462	sdrD	(N/A)	0,00	>=5,00	1,61	0,00	>=5,00	1,61	2,01	>=5,00	3,62
>0463	bbp=sdrE	(N/A)	2,03	4,18	>=5,00	1,12	4,44	>=5,00	3,27	>=5,00	>=5,00
>0464	sdgA	SAUR0588 (SAR_RS02940)	0,07	2,04	1,36	0,07	2,04	1,36	0,07	2,04	1,36
>0465	sdgB	SAUR0589 (SAR_RS02945)	0,13	3,82	1,27	0,13	3,82	1,27	0,20	3,89	1,34
>0466	folE2	SAUR0590 (SAR_RS02950)	0,00	0,80	0,80	0,00	0,80	0,80	0,00	0,80	0,80
>0467	bshB2	SAUR0591 (SAR_RS02955)	0,00	0,75	0,45	0,00	0,75	0,45	0,00	0,75	0,45
>0468	yojF=DUF18096	SAUR0592 (SAR_RS02960)	0,00	0,83	0,55	0,00	0,83	0,55	0,00	0,83	0,55
>0469	nagB	SAUR0593 (SAR_RS02965)	0,00	1,31	0,66	0,00	1,31	0,66	0,00	1,31	0,66
>0470	hxlA	SAUR0594 (SAR_RS02970)	0,00	0,79	0,00	0,00	0,79	0,00	0,00	0,79	0,00
>0471	sis=hxlB	SAUR0595 (SAR_RS02975)	0,00	2,19	0,18	0,00	2,19	0,18	0,00	2,19	0,18
>0472	Q2FJ68	SAUR0596 (SAR_RS02980)	0,15	3,24	0,15	0,15	3,24	0,15	0,15	3,24	0,15
>0473	proP	SAUR0597 (SAR_RS02985)	0,07	1,78	1,07	0,07	1,78	1,07	0,07	1,78	1,07
>0474	vraA	SAUR0598 (SAR_RS02990)	0,07	2,18	0,44	0,00	2,10	0,36	0,00	2,10	0,36
>0475	vraB	SAUR0599 (SAR_RS02995)	0,09	1,58	0,70	0,00	1,49	0,61	0,00	1,49	0,61
>0476	vraC	SAUR0600 (SAR_RS03000)	0,00	2,19	1,09	0,00	2,19	1,09	0,00	2,19	1,09
>0477	Q5HI98	SAUR0601 (SAR_RS03005)	0,00	>=5,00	>=5,00	0,00	>=5,00	>=5,00	0,00	>=5,00	>=5,00
>0478	thiD1=pdxK	SAUR0603 (SAR_RS03015)	0,00	0,84	0,36	0,12	0,96	0,48	0,12	0,96	0,48
>0479	ung	SAUR0604 (SAR_RS03020)	0,00	0,91	0,15	0,00	0,91	0,15	0,00	0,91	0,15
>0480	ywdI	SAUR0605 (SAR_RS03025)	0,00	1,84	0,26	0,26	2,10	0,52	0,26	2,10	0,52
>0481	ygdD=ywdK=DUF0423	SAUR0606 (SAR_RS03030)	0,00	0,27	0,27	0,27	0,54	0,54	0,27	0,54	0,54

Number/ Position in the figures	Gene ID	ID in PubMLST cg scheme	ED133 vs. swan isolate 15V8707	X22 vs. swan isolate 15V8707	CC522 goat isolate 17CS1042 vs. swan isolate 15V8707	ED133 vs. duck isolate V315	X22 vs. duck isolate V315	CC522 goat isolate 17CS1042 vs. duck isolate V315	ED133 vs. duck isolate V482	X22 vs. duck isolate V482	CC522 goat isolate 17CS1042 vs. duck isolate V482
>0482	yfnA2	SAUR0607 (SAR_RS03035)	0,07	0,81	0,40	0,00	0,74	0,34	0,07	0,81	0,40
>0483	DUF3815	SAUR0608 (SAR_RS03040)	0,00	>=5,00	0,44	0,00	>=5,00	0,44	0,00	>=5,00	0,44
>0484	DUF1212_L1	SAUR0609 (SAR_RS03045)	0,00	1,44	1,18	0,00	1,44	1,18	0,00	1,44	1,18
>0485	hemQ	SAUR0610 (SAR_RS03050)	0,00	0,66	0,40	0,00	0,66	0,40	0,00	0,66	0,40
>0486	pta	SAUR0611 (SAR_RS03055)	0,20	0,61	0,20	0,00	0,40	0,00	0,10	0,51	0,10
>0487	lipL	SAUR0612 (SAR_RS03060)	0,12	0,60	0,48	0,12	0,60	0,48	0,12	0,60	0,48
>0488	mvaK1	SAUR0613 (SAR_RS03065)	0,00	1,30	0,33	0,00	1,30	0,33	0,00	1,30	0,33
>0489	mvaD	SAUR0614 (SAR_RS03070)	0,20	1,42	0,61	0,10	1,32	0,51	0,20	1,42	0,61
>0490	mvaK2	SAUR0615 (SAR_RS03075)	0,00	0,37	0,46	0,00	0,37	0,46	0,00	0,37	0,46
>0491	DUF1450	SAUR0616 (SAR_RS03080)	0,29	0,29	0,58	0,00	0,00	0,29	0,00	0,00	0,29
>0492	ykgC	SAUR0617 (SAR_RS03085)	0,08	2,34	0,38	0,00	2,27	0,30	0,00	2,27	0,30
>0493	Q5HI81	SAUR0618 (SAR_RS03090)	0,00	3,17	0,23	0,00	3,17	0,23	0,00	3,17	0,23
>0494	Q5HI79	SAUR0620 (SAR_RS03100)	0,07	>=5,00	>=5,00	0,00	>=5,00	>=5,00	0,00	>=5,00	>=5,00
>0495	Q5HI67=iolS	SAUR0625 (SAR_RS03125)	0,00	0,64	0,64	0,00	0,64	0,64	0,00	0,64	0,64
>0496	Q5HI66	SAUR0626 (SAR_RS03130)	0,00	0,37	0,19	0,00	0,37	0,19	0,00	0,37	0,19
>0497	Q8NXU4	SAUR0627 (SAR_RS03135)	0,21	0,21	0,63	0,21	0,21	0,63	0,21	0,21	0,63
>0498	Q5HI65	SAUR0628 (SAR_RS03140)	0,08	0,69	0,31	0,00	0,62	0,23	0,00	0,62	0,23
>0499	ywhD	SAUR0629 (SAR_RS03145)	0,00	0,00	0,19	0,00	0,00	0,19	0,00	0,00	0,19
>0500	adhA=adhP	SAUR0630 (SAR_RS03150)	0,00	0,49	0,30	0,00	0,49	0,30	0,00	0,49	0,30
>0501	ywiB=DUF1934	SAUR0632 (SAR_RS03160)	0,00	0,70	0,70	0,00	0,70	0,70	0,00	0,70	0,70
>0502	argS	SAUR0633 (SAR_RS03165)	0,00	0,66	0,42	0,00	0,66	0,42	0,00	0,66	0,42
>0503	nth2	SAUR0634 (SAR_RS03170)	0,00	2,83	0,00	0,00	2,83	0,00	0,00	2,83	0,00
>0504	A5IQG3	SAUR0635 (SAR_RS03175)	0,00	0,90	0,34	0,00	0,90	0,34	0,00	0,90	0,34
>0505	yvrB=yibE	SAUR2983	0,00	1,26	1,16	0,00	1,26	1,16	0,11	1,37	1,26
>0506	ppaX2	SAUR0637 (SAR_RS03185)	0,14	0,97	1,11	0,00	0,83	0,97	0,00	0,83	0,97
>0507	ydjP	SAUR0638 (SAR_RS03190)	0,00	1,12	0,87	0,25	1,37	1,12	0,25	1,37	1,12
>0508	Q5HI54	SAUR0639 (SAR_RS03195)	0,00	0,99	0,20	0,00	0,99	0,20	0,00	0,99	0,20
>0509	Q5HI53	SAUR0640 (SAR_RS03200)	0,00	0,28	0,28	0,00	0,28	0,28	0,00	0,28	0,28
>0510	Q5HI52=pip	SAUR0641 (SAR_RS03205)	0,13	1,14	0,38	0,00	1,01	0,25	0,00	1,01	0,25
>0511	sarA	(N/A)	0,00	0,00	0,00	0,00	0,00	0,00	0,00	0,00	0,00
>0512	Q2YSV8	SAUR0643 (SAR_RS03215)	0,00	>=5,00	0,11	0,00	>=5,00	0,11	0,00	>=5,00	0,11
>0513	A6QET1=DUF2922	SAUR0644 (SAR_RS03220)	0,00	0,44	0,00	0,00	0,44	0,00	0,00	0,44	0,00
>0514	Q7A1N3=DUF1659	SAUR0645 (SAR_RS03225)	0,00	3,43	0,00	0,00	3,43	0,00	0,00	3,43	0,00
>0515	A5IQH4	SAUR0646 (SAR_RS03230)	0,00	1,07	0,53	0,00	1,07	0,53	0,00	1,07	0,53
>0516	mrpA	SAUR0647 (SAR_RS03235)	0,04	2,62	2,87	0,00	2,58	2,83	0,04	2,62	2,87
>0517	mrpB	SAUR0648 (SAR_RS03240)	0,00	>=5,00	>=5,00	0,00	>=5,00	>=5,00	0,00	>=5,00	>=5,00
>0518	mrpC	SAUR0649 (SAR_RS03245)	0,00	1,45	0,87	0,29	1,73	1,16	0,00	1,45	0,87
>0519	mrpD	SAUR0650 (SAR_RS03250)	0,00	4,81	4,21	0,00	4,81	4,21	0,00	4,81	4,21
>0520	mrpE	SAUR0651 (SAR_RS03255)	0,00	1,04	0,41	0,00	1,04	0,41	0,00	1,04	0,41
>0521	mrpF	SAUR0652 (SAR_RS03260)	0,00	0,66	0,00	0,00	0,66	0,00	0,00	0,66	0,00
>0522	mrpG	(N/A)	0,00	1,37	0,00	0,00	1,37	0,00	0,00	1,37	0,00
>0523	nhaK1	SAUR0654 (SAR_RS03270)	0,00	0,49	0,34	0,00	0,49	0,34	0,00	0,49	0,34
>0524	mntC	SAUR0658 (SAR_RS03290)	0,11	0,43	0,43	0,00	0,32	0,32	0,00	0,32	0,32
>0525	mntB	SAUR0659 (SAR_RS03295)	0,00	0,12	0,24	0,00	0,12	0,24	0,00	0,12	0,24
>0526	mntA	SAUR0660 (SAR_RS03300)	0,00	0,00	0,27	0,00	0,00	0,27	0,00	0,00	0,27
>0527	mntR	SAUR0661 (SAR_RS03305)	0,00	1,24	1,09	0,00	1,24	1,09	0,16	1,40	1,24
>0528	Q5HI33	SAUR0662 (SAR_RS03310)	0,00	0,00	0,13	0,00	0,00	0,13	0,00	0,00	0,13
>0529	tarA=tagA	SAUR0663 (SAR_RS03315)	0,13	1,31	0,26	0,00	1,18	0,13	0,00	1,18	0,13
>0530	tarH=tagH	SAUR0664 (SAR_RS03320)	0,00	0,38	0,00	0,00	0,38	0,00	0,00	0,38	0,00
>0531	tarG=tagG	SAUR0665 (SAR_RS03325)	0,00	0,60	0,00	0,00	0,60	0,00	0,00	0,60	0,00
>0532	tarB=tagB	SAUR0666 (SAR_RS03330)	0,00	1,27	0,91	0,00	1,27	0,91	0,00	1,27	0,91
>0533	tarX	SAUR0667 (SAR_RS03335)	0,09	1,32	0,38	0,09	1,32	0,38	0,09	1,32	0,38
>0534	tarD=tagD	SAUR0668 (SAR_RS03340)	0,00	0,00	0,50	0,00	0,00	0,50	0,00	0,00	0,50
>0535	pbpD=pbp4=dacA	SAUR0669 (SAR_RS03345)	0,00	1,39	0,62	0,00	1,39	0,62	0,00	1,39	0,62
>0536	msbA1	SAUR0670 (SAR_RS03350)	0,00	1,16	0,00	0,00	1,16	0,00	0,00	1,16	0,00
>0537	nupG	SAUR0671 (SAR_RS03355)	0,00	0,57	0,24	0,00	0,57	0,24	0,00	0,57	0,24
>0538	yxkD=yitT=DUF2197	SAUR0673 (SAR_RS03365)	0,00	0,96	0,00	0,00	0,96	0,00	0,00	0,96	0,00
>0539	fhuC	SAUR0674 (SAR_RS03370)	0,00	0,75	0,25	0,00	0,75	0,25	0,13	0,88	0,38
>0540	fhuB	SAUR0675 (SAR_RS03375)	0,00	0,30	0,00	0,00	0,30	0,00	0,10	0,40	0,10
>0541	fhuG	SAUR0676 (SAR_RS03380)	0,29	1,47	0,20	0,39	1,57	0,29	0,39	1,57	0,29
>0542	dakK=dhaK	SAUR0677 (SAR_RS03385)	0,10	1,76	0,00	0,10	1,76	0,00	0,10	1,76	0,00
>0543	dakL=dhaL	SAUR0678 (SAR_RS03390)	0,00	1,20	0,00	0,00	1,20	0,00	0,00	1,20	0,00
>0544	dakP=dhaM	SAUR0679 (SAR_RS03395)	0,00	1,10	0,00	0,55	1,65	0,55	0,28	1,38	0,28
>0545	Q5HI15	SAUR0680 (SAR_RS03400)	0,00	0,20	0,20	0,00	0,20	0,20	0,00	0,20	0,20
>0546	Q5HI14	SAUR0681 (SAR_RS03405)	0,00	1,03	0,38	0,00	1,03	0,38	0,00	1,03	0,38
>0547	lipA	SAUR0903 (SAR_RS04515)	0,10	1,44	1,44	0,00	1,34	1,34	0,00	1,34	1,34
>0548	Q5HI12	SAUR0684 (SAR_RS03420)	0,00	2,80	1,17	0,00	2,80	1,17	0,00	2,80	1,17
>0549	Q5HI11	SAUR0685 (SAR_RS03425)	0,00	0,20	0,59	0,00	0,20	0,59	0,00	0,20	0,59
>0550	graX	SAUR0686 (SAR_RS03430)	0,00	0,65	0,00	0,00	0,65	0,00	0,00	0,65	0,00
>0551	graR	SAUR0687 (SAR_RS03435)	0,15	2,96	0,44	0,00	2,81	0,30	0,15	2,96	0,44
>0552	graS	SAUR0688 (SAR_RS03440)	0,10	0,48	0,48	0,10	0,48	0,48	0,10	0,48	0,48
>0553	vraF	SAUR0689 (SAR_RS03445)	0,00	>=5,00	0,39	0,00	>=5,00	0,39	0,00	>=5,00	0,39
>0554	vraG	SAUR0690 (SAR_RS03450)	0,00	1,11	0,63	0,11	1,22	0,74	0,05	1,16	0,69
>0555	ykaA=pitR	SAUR0691 (SAR_RS03455)	0,00	0,49	0,00	0,00	0,49	0,00	0,00	0,49	0,00
>0556	pitA	SAUR0692 (SAR_RS03460)	0,00	3,08	0,00	0,00	3,08	0,00	0,00	3,08	0,00
>0557	ssaA5	SAUR0693 (SAR_RS03465)	0,00	0,50	0,38	0,00	0,50	0,38	0,13	0,63	0,50
>0558	yetJ	SAUR0694 (SAR_RS03470)	0,00	0,47	0,47	0,00	0,47	0,47	0,00	0,47	0,47
>0559	rbf	SAUR0695 (SAR_RS03475)+SAU	0,00	1,72	0,56	0,00	1,72	0,56	0,00	1,72	0,56
>0560	sarX	SAUR0697 (SAR_RS03485)	0,23	0,94	0,00	0,23	0,94	0,00	0,23	0,94	0,00
>0561	yeeI	SAUR0698 (SAR_RS03490)	0,00	0,14	0,70	0,00	0,14	0,70	0,00	0,14	0,70
>0562	DUF0985	SAUR0699 (SAR_RS03495)	0,00	1,48	0,21	0,00	1,48	0,21	0,00	1,48	0,21
>0563	A5IQM5=DUF0402	SAUR0701 (SAR_RS03505)	0,00	1,09	0,16	0,00	1,09	0,16	>=5,00	>=5,00	>=5,00
>0564	ccpE	SAUR0702 (SAR_RS03510)	0,00	2,54	0,23	0,00	2,54	0,23	0,00	2,54	0,23
>0565	A8Z196=setA	SAUR0703 (SAR_RS03515)	0,00	1,15	0,33	0,00	1,15	0,33	0,00	1,15	0,33
>0566	Q2YSN2=DUF456	(N/A)	0,00	1,23	1,64	0,00	1,23	1,64	0,00	1,23	1,64
>0567	Q2YSQ6=DUF1129	SAUR2984	0,14	1,59	0,72	0,14	1,59	0,72	0,14	1,59	0,72
>0568	A5IQN0	SAUR0740 (SAR_RS03700)	0,00	>=5,00	0,00	0,00	>=5,00	0,00	0,00	>=5,00	0,00
>0569	A8YZU8	SAUR0741 (SAR_RS03705)	0,00	>=5,00	>=5,00	0,00	>=5,00	>=5,00	0,00	>=5,00	>=5,00
>0570	Q5HHZ0	SAUR0742 (SAR_RS03710)	0,00	1,67	2,00	0,00	1,67	2,00	0,00	1,67	2,00
>0571	ykkB	SAUR0743 (SAR_RS03715)	0,18	0,74	0,55	0,18	0,74	0,55	0,18	0,74	0,55
>0572	yvdD	SAUR0744 (SAR_RS03720)	0,00	1,23	0,88	0,00	1,23	0,88	0,00	1,23	0,88
>0573	yqxD	SAUR0745 (SAR_RS03725)	0,00	1,74	0,22	0,00	1,74	0,22	0,22	1,74	0,44
>0574	Q5HHY6	SAUR0746 (SAR_RS03730)	0,00	1,02	0,29	0,00	1,02	0,29	0,15	1,17	0,44
>0575	uppP	SAUR0747 (SAR_RS03735)	0,11	1,94	0,11	0,23	2,05	0,23	0,23	2,05	0,23
>0576	cydD	SAUR0748 (SAR_RS03740)	0,06	1,04	0,12	0,00	0,98	0,06	0,00	0,98	0,06
>0577	cydC	SAUR0749 (SAR_RS03745)	0,00	1,25	0,84	0,00	1,25	0,84	0,06	1,25	0,84
>0578	mgrA	(N/A)	0,00	0,45	0,00	0,00	0,45	0,00	0,00	0,45	0,00
>0579	cobW2	SAUR0751 (SAR_RS03755)	0,00	1,40	0,54	0,00	1,40	0,54	0,00	1,40	0,54
>0580	ycsN	SAUR0752 (SAR_RS03760)	>=5,00	>=5,00	>=5,00	>=5,00	>=5,00	>=5,00	>=5,00	>=5,00	>=5,00
>0581	Q5HHX9	SAUR0753 (SAR_RS03765)	0,00	0,69	0,00	0,00	0,69	0,00	0,00	0,69	0,00
>0582	yflS	SAUR0754 (SAR_RS03770)	0,00	1,87	0,58	0,00	1,87	0,58	0,00	1,87	0,58
>0583	phrB	SAUR0755 (SAR_RS03775)	0,00	>=5,00	2,77	0,00	>=5,00	2,77	0,00	>=5,00	2,77
>0584	Q2G0A5	SAUR0756 (SAR_RS03780)	0,35	>=5,00	0,34	0,00	>=5,00	0,00	0,00	>=5,00	0,00
>0585	Q5HHX6-DUF1361	(N/A)	0,16	>=5,00	1,29	0,16	>=5,00	1,29	0,32	>=5,00	1,45
>0586	Q5HHX5=DUF0296	SAUR0758 (SAR_RS03790)	0,00	>=5,00	>=5,00	0,00	>=5,00	>=5,00	0,00	>=5,00	>=5,00
>0587	norA	SAUR0759 (SAR_RS03795)	0,00	4,54	0,94	0,00	4,54	0,94	0,00	4,54	0,94
>0588	yedL	SAUR0760 (SAR_RS03800)	0,00	1,32	0,22	0,00	1,32	0,22	0,00	1,32	0,22
>0589	ybaK	SAUR0761 (SAR_RS03805)	0,00	2,69	0,00	0,00	2,69	0,00	0,00	2,69	0,00
>0590	fruR	SAUR0762 (SAR_RS03810)	0,00	0,79	0,26	0,00	0,79	0,26	0,00	0,79	0,26
>0591	fruB=pfkB	SAUR0763 (SAR_RS03815)	0,00	2,39	0,87	0,11	2,50	0,98	0,00	2,39	0,87
>0592	fruA	SAUR0764 (SAR_RS03820)	0,00	4,38	1,32	0,00	4,38	1,32	0,05	4,43	1,37
>0593	nagA	SAUR0766 (SAR_RS03830)	0,00	1,95	0,17	0,00	1,95	0,17	0,00	1,95	0,17
>0594	corC=mpfA=ytfL	SAUR0767 (SAR_RS03835)	0,00	0,74	0,15	0,00	0,74	0,15	0,00	0,74	0,15
>0595	yvgN1	SAUR0768 (SAR_RS03840)	0,00	0,71	0,48	0,00	0,71	0,48	0,00	0,71	0,48
>0596	csbB	SAUR0769 (SAR_RS03845)	0,00	2,34	0,61	0,00	2,34	0,61	0,00	2,34	0,61
>0597	saeS	SAUR0770 (SAR_RS03850)	0,09	0,47	0,47	0,00	0,38	0,38	0,00	0,38	0,38
>0598	saeR	SAUR0771 (SAR_RS03855)	0,00	0,15	0,00	0,00	0,15	0,00	0,00	0,15	0,00
>0599	saeQ	SAUR0772 (SAR_RS03860)	0,00	0,63	0,42	0,21	0,84	0,63	0,21	0,84	0,63
>0600	saeP	SAUR0773 (SAR_RS03865)	0,00	0,68	3,17	0,00	0,68	3,17	0,00	0,68	3,17
>0601	Q5HHW1	SAUR2985	0,00	0,51	0,17	0,00	0,51	0,17	0,00	0,51	0,17
>0602	queE	SAUR0775 (SAR_RS03875)	0,00	0,42	0,42	0,00	0,42	0,42	0,00	0,42	0,42
>0603	queD	SAUR0776 (SAR_RS03880)	0,00	1,43	0,00	0,00	1,43	0,00	0,00	1,43	0,00
>0604	queC	SAUR0777 (SAR_RS03885)	0,00	0,45	0,45	0,00	0,45	0,45	0,00	0,45	0,45
>0605	pabA	SAUR0778 (SAR_RS03890)	0,00	0,67	0,17	0,00	0,67	0,17	0,00	0,67	0,17
>0606	pabB	SAUR0779 (SAR_RS03895)	0,00	0,95	0,26	0,00	0,95	0,26	0,00	0,95	0,26
>0607	pabC	SAUR0780 (SAR_RS03900)	0,00	1,64	0,66	0,00	1,64	0,66	0,00	1,64	0,66
>0608	Q6GIS6	SAUR0781 (SAR_RS03905)	0,00	1,45	>=5,00	0,00	1,45	>=5,00	0,00	1,45	>=5,00
>0609	ahs1=kipI	SAUR0782 (SAR_RS03910)	0,00	1,27	0,28	0,00	1,27	0,28	0,00	1,27	0,28
>0610	ahs2	SAUR0783 (SAR_RS03915)	0,00	0,70	0,20	0,00	0,70	0,20	0,10	0,80	0,30
>0611	ltaS	SAUR0784 (SAR_RS03920)	0,00	0,46	0,21	0,00	0,46	0,21	0,15	0,62	0,36
>0612	uup	SAUR0786 (SAR_RS03930)	>=5,00	>=5,00	>=5,00	0,05	0,85	0,53	0,05	0,85	0,53
>0613	recQ	SAUR0787 (SAR_RS03935)	0,00	0,84	0,28	0,00	0,84	0,28	0,06	0,90	0,34
>0614	opuBA	SAUR0788 (SAR_RS03940)	0,00	1,43	0,41	0,00	1,43	0,41	0,00	1,43	0,41
>0615	opuBB	SAUR0789 (SAR_RS03945)	0,00	0,86	0,20	0,00	0,86	0,20	0,00	0,86	0,20
>0616	hisC2	SAUR0790 (SAR_RS03950)	0,00	0,85	0,66	0,00	0,85	0,66	0,00	0,85	0,66
>0617	yorS	SAUR0791 (SAR_RS03955)	0,00	1,64	1,64	0,00	1,64	1,64	0,00	1,64	1,64
>0618	bmrU=dgkA1	SAUR0792 (SAR_RS03960)	0,00	1,74	0,22	0,00	1,74	0,22	0,00	1,74	0,22
>0619	dtpT	SAUR0794 (SAR_RS03970)	0,00	1,06	0,46	0,00	1,06	0,46	0,00	1,06	0,46
>0620	queF	SAUR0795 (SAR_RS03975)	1,59	1,59	1,20	1,59	1,59	1,20	1,59	1,59	1,20
>0621	yxxF	SAUR0796 (SAR_RS03980)	0,00	0,69	0,58	0,12	0,81	0,69	0,12	0,81	0,69
>0622	nrdI	(N/A)	0,00	0,75	0,50	0,00	0,75	0,50	0,00	0,75	0,50
>0623	nrdE	SAUR0798 (SAR_RS03990)	0,00	1,04	0,57	0,00	1,04	0,57	0,00	1,04	0,57
>0624	nrdF	SAUR0799 (SAR_RS03995)	0,00	0,62	0,10	0,00	0,62	0,10	0,00	0,62	0,10
>0625	sstA	SAUR0801 (SAR_RS04005)	0,00	0,41	0,62	0,00	0,41	0,62	0,00	0,41	0,62
>0626	sstB	SAUR0802 (SAR_RS04010)	0,00	1,15	0,63	0,00	1,15	0,63	0,10	1,25	0,73
>0627	sstC	SAUR0803 (SAR_RS04015)	0,00	1,05	0,79	0,00	1,05	0,79	0,00	1,05	0,79
>0628	sstD	SAUR0804 (SAR_RS04020)	0,10	1,26	1,07	0,00	1,17	0,97	0,00	1,17	0,97
>0629	Q5HHT3	SAUR0805 (SAR_RS04025)	0,00	1,27	1,59	0,00	1,27	1,59	0,00	1,27	1,59

Number/ Position in the figures	Gene ID	ID in PubMLST cg scheme	ED133 vs. swan isolate 15V8707	X22 vs. swan isolate 15V8707	CC522 goat isolate 17CS1042 vs. swan isolate 15V8707	ED133 vs. duck isolate V315	X22 vs. duck isolate V315	CC522 goat isolate 17CS1042 vs. duck isolate V315	ED133 vs. duck isolate V482	X22 vs. duck isolate V482	CC522 goat isolate 17CS1042 vs. duck isolate V482
>0630	murB	SAUR0806 (SAR_RS04030)	0,00	0,65	0,43	0,00	0,65	0,43	0,00	0,65	0,43
>0631	grpB	SAUR0807 (SAR_RS04035)	0,00	1,16	0,19	0,00	1,16	0,19	0,00	1,16	0,19
>0632	Q5HHT0	SAUR0808 (SAR_RS04040)	0,00	0,91	0,68	0,00	0,91	0,68	0,11	1,02	0,80
>0633	ytxJ	SAUR0809 (SAR_RS04045)	0,00	0,31	0,00	0,00	0,31	0,00	0,00	0,31	0,00
>0634	glxK2	SAUR0810 (SAR_RS04050)	0,00	1,07	>=5,00	0,00	1,07	>=5,00	0,00	1,07	>=5,00
>0635	pepT	SAUR0811 (SAR_RS04055)	0,00	0,65	0,81	0,00	0,65	0,81	0,00	0,65	0,81
>0636	Q5HHS6	SAUR0812 (SAR_RS04060)	0,00	0,81	0,20	0,00	0,81	0,20	0,00	0,81	0,20
>0637	DUF1212_L2=yjjP	SAUR0813 (SAR_RS04065)	0,00	0,79	0,79	0,00	0,79	0,79	0,13	0,92	0,92
>0638	gdpS	SAUR0814 (SAR_RS04070)	0,00	0,84	0,47	0,09	0,93	0,56	0,19	1,03	0,65
>0639	tarO	SAUR0815 (SAR_RS04075)	0,00	1,04	0,28	0,09	1,14	0,38	0,00	1,04	0,28
>0640	Q5HHS2	SAUR0816 (SAR_RS04080)	0,16	1,09	0,78	0,00	0,93	0,62	0,00	0,93	0,62
>0641	degV1=fakB1	(N/A)	0,12	0,46	0,58	0,12	0,46	0,58	0,23	0,58	0,69
>0642	comFA	SAUR0818 (SAR_RS04090)	0,00	1,39	0,28	0,00	1,39	0,28	0,00	1,39	0,28
>0643	comFC	SAUR0819 (SAR_RS04095)	0,00	0,29	0,88	0,00	0,29	0,88	0,00	0,29	0,88
>0644	yfiA=raiA	SAUR0820 (SAR_RS04100)	0,17	0,35	0,35	0,00	0,17	0,17	0,00	0,17	0,17
>0645	secA1	SAUR0821 (SAR_RS04105)	0,00	0,71	0,67	0,00	0,71	0,67	0,00	0,71	0,67
>0646	prfB	SAUR0823 (SAR_RS04115)	0,00	>=5,00	>=5,00	0,00	>=5,00	>=5,00	0,00	>=5,00	>=5,00
>0647	lysM	SAUR0825 (SAR_RS04125)	0,00	2,74	0,24	0,00	2,74	0,24	0,00	2,74	0,24
>0648	yfbR	SAUR0826 (SAR_RS04130)	0,00	1,07	0,15	0,00	1,07	0,15	0,00	1,07	0,15
>0649	DUF2198	SAUR0827 (SAR_RS04135)	0,00	1,68	2,52	0,00	1,68	2,52	0,00	1,68	2,52
>0650	uvrB	SAUR0828 (SAR_RS04140)	0,00	1,96	0,25	0,00	1,96	0,25	0,05	2,01	0,30
>0651	uvrA	SAUR0829 (SAR_RS04145)	0,00	1,54	0,67	0,11	1,65	0,77	0,04	1,58	0,70
>0652	hpr=hprK	SAUR0831 (SAR_RS04155)	0,00	0,86	0,43	0,11	0,96	0,54	0,11	0,96	0,54
>0653	lgt	SAUR0832 (SAR_RS04160)	0,00	0,95	0,12	0,00	0,95	0,12	0,00	0,95	0,12
>0654	yvoF	SAUR0833 (SAR_RS04165)	0,00	>=5,00	0,00	0,00	>=5,00	0,00	0,00	>=5,00	0,00
>0655	yvcD	SAUR0834 (SAR_RS04170)	0,07	1,39	0,76	0,14	1,46	0,83	0,14	1,46	0,83
>0656	trxB	SAUR0835 (SAR_RS04175)	0,00	0,64	0,32	0,00	0,64	0,32	0,00	0,64	0,32
>0657	yvcJ=rapZ	SAUR0836 (SAR_RS04180)	0,00	1,43	0,11	0,11	1,54	0,22	0,00	1,43	0,11
>0658	mgfK=yvcK	SAUR0837 (SAR_RS04185)	0,00	0,90	0,90	0,00	0,90	0,90	0,00	0,90	0,90
>0659	whiA	SAUR0838 (SAR_RS04190)	0,00	0,32	0,00	0,00	0,32	0,00	0,00	0,32	0,00
>0660	clpP	SAUR0840 (SAR_RS04200)	0,00	0,51	0,17	0,00	0,51	0,17	0,00	0,51	0,17
>0661	yfcH	SAUR0842 (SAR_RS04210)	0,00	1,11	0,89	0,11	1,11	1,00	0,00	1,11	0,89
>0662	Q5HHP8=DUF4887	SAUR0843 (SAR_RS04215)	0,00	0,63	0,32	0,00	0,63	0,32	0,00	0,63	0,32
>0663	gapR	SAUR0844 (SAR_RS04220)	0,00	0,69	0,39	0,10	0,79	0,49	0,10	0,79	0,49
>0664	gapA	SAUR0845 (SAR_RS04225)	0,00	0,30	0,10	0,10	0,40	0,20	0,10	0,40	0,20
>0665	pgk	SAUR0846 (SAR_RS04230)	0,08	1,01	0,50	0,00	0,92	0,42	0,00	0,92	0,42
>0666	tpi	SAUR0847 (SAR_RS04235)	0,00	0,79	0,79	0,13	0,92	0,92	0,00	0,79	0,79
>0667	gpmI	SAUR0848 (SAR_RS04240)	0,00	1,25	0,72	0,07	1,32	0,79	0,00	1,25	0,72
>0668	eno	SAUR0849 (SAR_RS04245)	0,00	0,23	0,23	0,00	0,23	0,23	0,00	0,23	0,23
>0669	Q2YSE7	SAUR0850 (SAR_RS04250)	0,00	1,31	0,44	0,00	1,31	0,44	0,22	1,53	0,65
>0670	secG	SAUR0851 (SAR_RS04255)	0,00	0,00	0,00	0,00	0,00	0,00	0,00	0,00	0,00
>0671	est	SAUR0852 (SAR_RS04260)	0,00	>=5,00	0,13	0,00	>=5,00	0,13	0,00	>=5,00	0,13
>0672	rnr	SAUR0853 (SAR_RS04265)	0,00	>=5,00	1,26	0,00	>=5,00	1,26	0,00	>=5,00	1,26
>0673	ssrP=rapZ	SAUR0854 (SAR_RS04270)	0,00	3,01	0,22	0,00	3,01	0,22	0,00	3,01	0,22
>0674	Q1YB79=DUF5067	SAUR0856 (SAR_RS04280)	0,13	1,96	0,92	4,58	>=5,00	>=5,00	4,58	>=5,00	>=5,00
>0675	Q5HHN0	SAUR0858 (SAR_RS04290)	0,00	1,15	0,77	0,19	1,34	0,96	0,19	1,34	0,96
>0676	Q1XZI1	SAUR0859 (SAR_RS04295)	0,00	1,69	0,94	0,00	1,69	0,94	0,00	1,69	0,94
>0677	clfA	(N/A)	>=5,00	>=5,00	>=5,00	>=5,00	>=5,00	>=5,00	4,61	>=5,00	>=5,00
>0678	vwb	(N/A)	0,07	>=5,00	>=5,00	0,00	>=5,00	>=5,00	0,07	>=5,00	>=5,00
>0679	emp	SAUR0862 (SAR_RS04310)	0,00	4,01	1,08	0,00	4,01	1,08	0,00	4,01	1,08
>0680	vwb2	SAUR0862 (SAR_RS04310)	0,00	>=5,00	>=5,00	0,00	>=5,00	>=5,00	0,19	>=5,00	>=5,00
>0681	nuc1	SAUR0864 (SAR_RS04320)	0,00	1,02	0,00	0,00	1,02	0,00	0,00	1,02	0,00
>0682	cspC-L1	(N/A)	0,00	0,00	0,00	0,00	0,00	0,00	0,00	0,00	0,00
>0683	Q1Y1Z8	SAUR0866 (SAR_RS04330)	0,00	0,91	0,46	>=5,00	>=5,00	>=5,00	0,00	0,91	0,46
>0684	Q2G007	SAUR0867 (SAR_RS04335)	0,00	1,05	0,35	0,00	1,05	0,35	0,00	1,05	0,35
>0685	Q1YB69	SAUR0868 (SAR_RS04340)	0,00	0,88	0,53	0,00	0,88	0,53	0,00	0,88	0,53
>0686	Q6GIJ6	SAUR0869 (SAR_RS04345)	0,00	3,17	1,59	0,00	3,17	1,59	0,00	3,17	1,59
>0687	Q6GIJ5	SAUR0870 (SAR_RS04350)	0,00	0,38	0,38	0,00	0,38	0,38	0,00	0,38	0,38
>0688	DUF1250	SAUR0871 (SAR_RS04355)	0,00	0,00	0,49	0,00	0,00	0,49	0,00	0,00	0,49
>0689	Q6GIJ3	SAUR0872 (SAR_RS04360)	0,00	0,42	0,00	0,00	0,42	0,00	0,00	0,42	0,00
>0690	gpmA1=cobC	SAUR0873 (SAR_RS04365)	0,00	>=5,00	0,85	0,00	>=5,00	0,85	0,00	>=5,00	0,85
>0691	yisU1	SAUR0874 (SAR_RS04370)	0,00	1,62	1,94	0,00	1,62	1,94	0,00	1,62	1,94
>0692	Q5HHL4	SAUR0875 (SAR_RS04375)	0,00	0,40	0,20	0,00	0,40	0,20	0,00	0,40	0,20
>0693	ohrB	SAUR0876 (SAR_RS04380)	0,00	0,95	1,18	0,00	0,95	1,18	0,00	0,95	1,18
>0694	aroD	SAUR0877 (SAR_RS04385)	0,00	1,67	1,12	0,00	1,67	1,12	0,00	1,67	1,12
>0695	ntrA	SAUR0878 (SAR_RS04390)	0,19	0,37	0,00	0,19	0,37	0,00	0,19	0,37	0,00
>0696	trxA2	SAUR0879 (SAR_RS04395)	0,00	0,93	0,00	0,00	0,93	0,00	0,00	0,93	0,00
>0697	yusI	SAUR0880 (SAR_RS04400)	0,00	1,12	0,56	0,00	1,12	0,56	0,00	1,12	0,56
>0698	gcvH2	SAUR0881 (SAR_RS04405)	0,00	0,52	0,26	0,00	0,52	0,26	0,00	0,52	0,26
>0699	ywqG	SAUR0883 (SAR_RS04415)	0,00	1,71	0,57	0,00	1,71	0,57	0,11	1,82	0,68
>0700	yusF	SAUR0884 (SAR_RS04420)	0,00	1,29	1,03	0,00	1,29	1,03	0,00	1,29	1,03
>0701	yusE	SAUR0885 (SAR_RS04425)	0,00	1,01	0,67	0,00	1,01	0,67	0,00	1,01	0,67
>0702	metN1	SAUR0886 (SAR_RS04430)	0,00	0,68	0,29	0,00	0,68	0,29	0,10	0,78	0,39
>0703	metP1	SAUR0887 (SAR_RS04435)	0,00	0,86	0,72	0,00	0,86	0,72	0,00	0,86	0,72
>0704	metQ1	SAUR0888 (SAR_RS04440)	0,00	1,22	0,97	0,00	1,22	0,97	0,00	1,22	0,97
>0705	csbD-L1	SAUR0889 (SAR_RS04445)	0,00	0,51	0,51	0,00	0,51	0,51	0,00	0,51	0,51
>0706	DUF0368	SAUR0890 (SAR_RS04450)	0,35	1,53	0,59	0,23	1,41	0,47	0,23	1,41	0,47
>0707	sufC=yurY	SAUR0891 (SAR_RS04455)	0,00	0,52	0,26	0,00	0,52	0,26	0,00	0,52	0,26
>0708	sufD	SAUR0892 (SAR_RS04460)	0,15	0,92	0,69	0,15	0,92	0,69	0,15	0,92	0,69
>0709	sufS	SAUR0893 (SAR_RS04465)	0,00	0,72	0,24	0,00	0,72	0,24	0,00	0,72	0,24
>0710	sufN=uscU	SAUR0894 (SAR_RS04470)	0,00	0,00	0,00	0,22	0,22	0,22	0,22	0,22	0,22
>0711	sufB	SAUR0895 (SAR_RS04475)	0,00	0,29	0,36	0,00	0,29	0,36	0,07	0,36	0,43
>0712	Q2YWM5	SAUR0897 (SAR_RS04485)	0,00	1,27	0,32	0,32	1,59	0,63	0,32	1,59	0,63
>0713	corB	SAUR0898 (SAR_RS04490)	0,00	1,06	0,38	0,00	1,06	0,38	0,00	1,06	0,38
>0714	npd	SAUR0899 (SAR_RS04495)	0,00	>=5,00	0,26	0,00	>=5,00	0,26	0,00	>=5,00	0,26
>0715	yunF=yecE	SAUR0900 (SAR_RS04500)	0,00	0,47	0,94	0,00	0,47	0,94	0,00	0,47	0,94
>0716	yunE	SAUR0901 (SAR_RS04505)	0,00	>=5,00	2,29	0,00	>=5,00	2,29	0,00	>=5,00	2,29
>0717	yunD	SAUR0902 (SAR_RS04510)	0,00	0,98	0,61	0,00	0,98	0,61	0,08	1,06	0,68
>0718	lipA	SAUR0903 (SAR_RS04515)	0,00	0,22	0,00	0,00	0,22	0,00	0,00	0,22	0,00
>0719	utp-yutD=DUF1027	SAUR0904 (SAR_RS04520)	0,25	0,51	0,00	0,25	0,51	0,00	0,25	0,51	0,00
>0720	DUF3055	(N/A)	0,00	0,39	1,16	0,00	0,39	1,16	0,00	0,39	1,16
>0721	utp-yutE=DUF0086	SAUR2986	0,00	0,46	0,46	0,00	0,46	0,46	0,00	0,46	0,46
>0722	utp-yutF	SAUR0907 (SAR_RS04535)	0,00	1,54	0,26	0,00	1,54	0,26	0,00	1,54	0,26
>0723	gyaR	SAUR0908 (SAR_RS04540)	0,42	1,67	0,83	0,10	1,35	0,52	0,10	1,35	0,52
>0724	dltX	(N/A)	0,00	0,00	0,00	0,00	0,00	0,00	0,00	0,00	0,00
>0725	dltA	SAUR0911 (SAR_RS04555)	0,07	0,48	0,34	0,00	0,41	0,27	0,00	0,41	0,27
>0726	dltB	SAUR0912 (SAR_RS04560)	0,00	0,33	0,00	0,00	0,33	0,00	0,00	0,33	0,00
>0727	dltC	(N/A)	0,00	1,27	0,42	0,00	1,27	0,42	0,00	1,27	0,42
>0728	dltD	SAUR0914 (SAR_RS04570)	0,00	0,43	0,34	0,00	0,43	0,34	0,00	0,43	0,34
>0729	nfuA	SAUR0915 (SAR_RS04575)	0,00	0,90	0,45	0,00	0,90	0,45	0,00	0,90	0,45
>0730	yuzD	SAUR0916 (SAR_RS04580)	0,00	0,93	0,00	0,00	0,93	0,00	0,00	0,93	0,00
>0731	utp-yutJ=ndhF=yjlD	SAUR0917 (SAR_RS04585)	0,09	0,94	0,47	0,09	0,94	0,47	0,09	0,94	0,47
>0732	yuzB	SAUR0918 (SAR_RS04590)	0,00	0,00	0,00	0,00	0,00	0,00	0,00	0,00	0,00
>0733	sufA	SAUR0919 (SAR_RS04595)	0,00	1,11	0,83	0,00	1,11	0,83	0,00	1,11	0,83
>0734	ndhC=yumB	SAUR0920 (SAR_RS04600)	0,00	0,25	0,25	0,00	0,25	0,25	0,00	0,25	0,25
>0735	pepZ=ampA=pepA	SAUR0921 (SAR_RS04605)	0,00	0,88	1,49	0,00	0,88	1,49	0,00	0,88	1,49
>0736	nhaC3	SAUR0922 (SAR_RS04610)	0,00	1,67	0,53	0,00	1,67	0,53	0,00	1,67	0,53
>0737	yuxO	SAUR0923 (SAR_RS04615)	0,27	1,60	0,53	0,00	1,33	0,27	0,00	1,33	0,27
>0738	Q5HHE0	SAUR0924 (SAR_RS04620)	0,00	1,13	0,52	0,00	1,13	0,52	0,00	1,13	0,52
>0739	mnhG	SAUR0925 (SAR_RS04625)	0,00	1,12	0,84	0,00	1,12	0,84	0,00	1,12	0,84
>0740	mnhF	SAUR0926 (SAR_RS04630)	0,00	1,02	0,68	0,00	1,02	0,68	0,00	1,02	0,68
>0741	mnhE	SAUR0927 (SAR_RS04635)	0,00	1,46	1,46	0,00	1,46	1,46	0,00	1,46	1,46
>0742	mnhD	SAUR0928 (SAR_RS04640)	0,00	1,47	0,60	0,00	1,47	0,60	0,00	1,47	0,60
>0743	mnhC	SAUR0929 (SAR_RS04645)	0,00	0,58	0,00	0,00	0,58	0,00	0,00	0,58	0,00
>0744	mnhB	SAUR0930 (SAR_RS04650)	0,00	0,47	0,00	0,00	0,47	0,00	0,00	0,47	0,00
>0745	mnhA	SAUR0931 (SAR_RS04655)	0,00	0,37	0,29	0,04	0,42	0,33	0,04	0,42	0,33
>0746	kapB	SAUR0932 (SAR_RS04660)	0,00	0,78	1,04	0,00	0,78	1,04	0,00	0,78	1,04
>0747	prsA1	SAUR0933 (SAR_RS04665)	0,00	0,84	0,34	0,00	0,84	0,34	0,00	0,84	0,34
>0748	yugI=ygs	SAUR0934 (SAR_RS04670)	0,00	0,00	0,26	0,00	0,00	0,26	0,00	0,00	0,26
>0749	namA	SAUR0935 (SAR_RS04675)	0,09	0,98	0,89	0,00	0,89	0,80	0,09	0,98	0,89
>0750	rocD2	SAUR0936 (SAR_RS04680)	0,00	0,59	0,59	0,00	0,59	0,59	0,00	0,59	0,59
>0751	gluD	SAUR0937 (SAR_RS04685)	0,00	0,32	0,24	0,08	0,40	0,32	0,08	0,40	0,32
>0752	glpQ2	SAUR0938 (SAR_RS04690)	0,00	0,97	0,65	0,00	0,97	0,65	0,00	0,97	0,65
>0753	argH	SAUR0939 (SAR_RS04695)	0,00	0,58	>=5,00	0,00	0,58	>=5,00	0,00	0,58	>=5,00
>0754	argG	SAUR0940 (SAR_RS04700)	0,00	1,33	0,33	0,00	1,33	0,33	0,00	1,33	0,33
>0755	pgi	SAUR0941 (SAR_RS04705)	0,00	0,75	0,45	0,00	0,75	0,45	0,00	0,75	0,45
>0756	yhjE=ydjZ	SAUR0942 (SAR_RS04710)	0,00	2,26	0,52	0,00	2,26	0,52	0,00	2,26	0,52
>0757	spsA	SAUR0943 (SAR_RS04715)	0,00	0,57	0,38	0,00	0,57	0,38	0,00	0,57	0,38
>0758	spsB	SAUR0944 (SAR_RS04720)	0,00	1,74	0,17	0,00	1,74	0,17	0,00	1,74	0,17
>0759	rexB=addB	SAUR0945 (SAR_RS04725)	0,00	2,73	1,15	0,00	2,73	1,15	0,00	2,73	1,15
>0760	rexA=addA	SAUR0946 (SAR_RS04730)	0,00	1,34	0,74	0,03	1,37	0,77	0,05	1,40	0,79
>0761	Q5HHB6	SAUR0947 (SAR_RS04735)	0,00	0,44	0,33	0,00	0,44	0,33	0,00	0,44	0,33
>0762	UPF0344	SAUR0948 (SAR_RS04740)	0,00	4,87	0,77	0,00	4,87	0,77	0,00	4,87	0,77
>0763	cdr	SAUR0950 (SAR_RS04750)	0,00	1,29	0,68	0,00	1,29	0,68	0,08	1,37	0,76
>0764	yitU=yidA	SAUR0951 (SAR_RS04755)	0,00	0,61	0,12	0,00	0,61	0,12	0,12	0,73	0,24
>0765	sufT=paaD	SAUR0952 (SAR_RS04760)	0,00	0,00	0,97	0,00	0,00	0,97	0,00	0,00	0,97
>0766	Q5HHB1	SAUR0953 (SAR_RS04765)	0,06	0,94	0,66	0,06	0,94	0,66	0,11	0,99	0,72
>0767	clpB	SAUR0954 (SAR_RS04770)	0,04	0,69	0,46	0,04	0,69	0,46	0,04	0,69	0,46
>0768	Q5HHA9=lysR	SAUR0955 (SAR_RS04775)	0,00	1,49	1,49	0,00	1,49	1,49	0,00	1,49	1,49
>0769	Q5HHA5	SAUR0959 (SAR_RS04795)	0,19	1,55	0,19	0,00	1,36	0,00	0,00	1,36	0,00
>0770	eapH-2	(N/A)	0,00	1,61	0,23	0,00	1,61	0,23	0,00	1,61	0,23
>0771	A6U088=DUF2929	SAUR0961 (SAR_RS04805)	0,00	0,00	0,00	0,00	0,00	0,00	0,00	0,00	0,00
>0772	fabH	SAUR0962 (SAR_RS04810)	0,00	0,11	0,00	0,00	0,11	0,00	0,00	0,11	0,00
>0773	fabF	SAUR0963 (SAR_RS04815)	0,00	0,64	0,64	0,00	0,64	0,64	0,08	0,72	0,72
>0774	Q2YWV0=DUF3899	SAUR0964 (SAR_RS04820)	0,00	0,54	0,27	0,54	1,08	0,81	0,00	0,54	0,27
>0775	oppB	SAUR0965 (SAR_RS04825)	0,00	0,86	0,54	0,00	0,86	0,54	0,00	0,86	0,54
>0776	oppC	SAUR0966 (SAR_RS04830)	0,00	0,56	0,47	0,09	0,65	0,56	0,00	0,56	0,47
>0777	oppD	SAUR0967 (SAR_RS04835)	0,00	0,28	0,28	0,00	0,28	0,28	0,00	0,28	0,28

Number/ Position in the figures	Gene ID	ID in PubMLST cg scheme	ED133 vs. swan isolate 15V8707	X22 vs. swan isolate 15V8707	CC522 goat isolate 17CS1042 vs. swan isolate 15V8707	ED133 vs. duck isolate V315	X22 vs. duck isolate V315	CC522 goat isolate 17CS1042 vs. duck isolate V315	ED133 vs. duck isolate V482	X22 vs. duck isolate V482	CC522 goat isolate 17CS1042 vs. duck isolate V482
>0778	oppF	SAUR0968 (SAR_RS04840)	0,11	>=5,00	0,42	0,00	>=5,00	0,32	0,00	>=5,00	0,32
>0779	oppA	SAUR0969 (SAR_RS04845)	0,00	2,78	1,63	0,00	2,78	1,63	0,00	2,78	1,63
>0780	appA	(N/A)	0,12	1,98	0,12	0,12	1,98	0,12	0,17	2,04	0,17
>0781	appD	(N/A)	0,00	1,82	0,61	0,10	1,93	0,71	0,00	1,82	0,61
>0782	appF	(N/A)	0,10	3,06	0,10	0,00	2,96	0,00	0,00	2,96	0,00
>0783	appB	(N/A)	0,00	3,84	0,10	0,10	3,94	0,21	0,10	3,94	0,21
>0784	appC	(N/A)	0,00	0,57	0,34	0,11	0,68	0,45	0,00	0,57	0,34
>0785	trpS	SAUR0980 (SAR_RS04900)	0,00	2,83	0,40	0,10	2,93	0,51	0,10	2,93	0,51
>0786	spxA	SAUR0981 (SAR_RS04905)	0,00	0,00	0,00	0,00	0,00	0,00	0,00	0,00	0,00
>0787	trfA	SAUR0982 (SAR_RS04910)	0,00	0,83	0,14	0,00	0,83	0,14	0,00	0,83	0,14
>0788	trfB	SAUR0983 (SAR_RS04915)	0,00	1,62	0,41	0,00	1,62	0,41	0,00	1,62	0,41
>0789	pepF1	SAUR0984 (SAR_RS04920)	0,00	0,66	0,06	0,00	0,66	0,06	0,00	0,66	0,06
>0790	yjbH	SAUR0985 (SAR_RS04925)	0,12	0,37	>=5,00	0,00	0,25	>=5,00	0,00	0,25	>=5,00
>0791	yjbI	SAUR0986 (SAR_RS04930)	0,00	0,55	0,55	>=5,00	>=5,00	>=5,00	>=5,00	>=5,00	>=5,00
>0792	yjbK	SAUR0987 (SAR_RS04935)	0,17	1,52	0,84	0,00	1,35	0,67	0,00	1,35	0,67
>0793	yjbL	SAUR0988 (SAR_RS04940)	0,29	0,86	0,86	0,00	0,57	0,57	0,00	0,57	0,57
>0794	relQ	SAUR0989 (SAR_RS04945)	0,00	0,31	0,16	0,00	0,31	0,16	0,00	0,31	0,16
>0795	ppnK	SAUR0990 (SAR_RS04950)	0,12	0,25	0,12	0,00	0,12	0,00	0,00	0,12	0,00
>0796	rluE	SAUR0991 (SAR_RS04955)	0,12	1,64	0,00	0,12	1,64	0,00	0,12	1,64	0,00
>0797	mgtE	SAUR0992 (SAR_RS04960)	0,00	1,44	0,22	0,07	1,52	0,29	0,07	1,52	0,29
>0798	cpaA	SAUR0993 (SAR_RS04965)	0,00	0,43	0,22	0,00	0,43	0,22	0,00	0,43	0,22
>0799	fabI	(N/A)	0,00	0,91	0,78	0,00	0,91	0,78	0,00	0,91	0,78
>0800	UPF0118=cozEa	(N/A)	0,09	0,28	0,64	0,18	0,37	0,74	0,18	0,37	0,74
>0801	yrbD	SAUR0996 (SAR_RS04980)	0,06	1,15	0,96	0,00	1,08	0,89	0,00	1,08	0,89
>0802	yjcH	SAUR0997 (SAR_RS04985)	0,00	1,98	0,13	0,00	1,98	0,13	0,13	2,11	0,26
>0803	UPF0477	SAUR1000 (SAR_RS05000)	0,00	0,00	0,20	0,00	0,00	0,20	0,00	0,00	0,20
>0804	ltaA	SAUR1001 (SAR_RS05005)	0,00	0,92	0,67	0,00	0,92	0,67	0,00	0,92	0,67
>0805	ugtP	SAUR1002 (SAR_RS05010)	0,00	0,68	0,60	0,00	0,68	0,60	0,00	0,68	0,60
>0806	murE	SAUR1003 (SAR_RS05015)	0,00	0,81	0,47	0,07	0,88	0,54	0,00	0,81	0,47
>0807	yueH	SAUR1004 (SAR_RS05020)	0,00	2,38	1,59	0,00	2,38	1,59	0,00	2,38	1,59
>0808	prfC	SAUR1005 (SAR_RS05025)	0,00	0,70	0,83	0,00	0,70	0,83	0,00	0,70	0,83
>0809	terC	SAUR1006 (SAR_RS05030)	0,00	0,62	0,50	0,00	0,62	0,50	0,00	0,62	0,50
>0810	htrA=degP	SAUR1007 (SAR_RS05035)	0,00	1,39	1,26	0,13	1,52	1,39	0,13	1,47	1,39
>0811	ktrD=trkH	SAUR1008 (SAR_RS05040)	0,00	0,59	0,59	0,07	0,66	0,66	0,07	0,66	0,66
>0812	Q1XY52=yfkN3	SAUR1009 (SAR_RS05045)	0,00	0,86	0,13	0,00	0,86	0,13	0,00	0,86	0,13
>0813	comK	SAUR1012 (SAR_RS05060)	0,00	0,53	0,18	0,00	0,53	0,18	0,00	0,53	0,18
>0814	Q6GI58	SAUR1013 (SAR_RS05065)	0,00	0,44	0,44	0,00	0,44	0,44	0,00	0,44	0,44
>0815	lplA2	SAUR1014 (SAR_RS05070)	0,00	1,22	1,01	0,00	1,22	1,01	0,00	1,22	1,01
>0816	Q6GI56	(N/A)	0,00	0,00	0,00	0,56	0,56	0,56	0,00	0,00	0,00
>0817	Q5HH56	SAUR1016 (SAR_RS05080)	0,00	0,50	0,33	0,00	0,50	0,33	0,00	0,50	0,33
>0818	txpA-var1	(N/A)	0,00	0,93	>=5,00	0,00	0,93	>=5,00	0,00	0,93	>=5,00
>0819	Q7A194	(N/A)	0,00	>=5,00	0,00	0,00	>=5,00	0,00	0,00	>=5,00	0,00
>0820	yujE=DUF1430	SAUR1019 (SAR_RS05095)	0,00	1,42	0,61	0,00	1,42	0,61	0,00	1,42	0,61
>0821	yxeA	SAUR1020 (SAR_RS05100)	0,00	1,25	0,93	0,00	1,25	0,93	0,00	1,25	0,93
>0822	yujD	SAUR1021 (SAR_RS05105)	0,00	1,25	0,00	0,00	1,25	0,00	0,00	1,25	0,00
>0823	Q6GI49	SAUR1022 (SAR_RS05110)	0,00	0,34	0,00	0,34	0,69	0,34	0,34	0,69	0,34
>0824	Q6GI46	SAUR1026 (SAR_RS05130)	0,00	0,84	0,00	0,00	0,84	0,00	0,00	0,84	0,00
>0825	Q5HH47	SAUR1027 (SAR_RS05135)	0,00	>=5,00	0,73	0,00	>=5,00	0,73	0,00	>=5,00	0,73
>0826	shpA	SAUR1028 (SAR_RS05140)	0,00	0,85	0,85	0,00	0,85	0,85	0,00	0,85	0,85
>0827	Q7A191-(291 bp)	SAUR1029 (SAR_RS05145)	0,00	0,34	0,00	0,00	0,34	0,00	0,00	0,34	0,00
>0828	Q6GAH2	SAUR1030 (SAR_RS05150)	0,00	1,09	0,91	0,18	1,27	1,09	0,18	1,27	1,09
>0829	menA	SAUR1031 (SAR_RS05155)	0,00	0,64	0,85	0,00	0,64	0,85	0,00	0,64	0,85
>0830	menF	SAUR1032 (SAR_RS05160)	0,00	1,25	0,22	0,00	1,25	0,22	0,00	1,25	0,22
>0831	menD	SAUR1033 (SAR_RS05165)	0,00	2,27	0,18	0,00	2,27	0,18	0,00	2,27	0,18
>0832	menH	SAUR1034 (SAR_RS05170)	0,00	0,87	0,75	0,00	0,87	0,75	0,12	0,75	0,87
>0833	menB	SAUR1035 (SAR_RS05175)	0,00	0,36	0,73	0,00	0,36	0,73	0,00	0,36	0,73
>0834	sspC	SAUR1036 (SAR_RS05180)	0,00	0,91	0,61	0,00	0,91	0,61	0,00	0,91	0,61
>0835	sspB	SAUR1037 (SAR_RS05185)	0,08	1,35	1,95	0,00	1,27	1,86	0,00	1,27	1,86
>0836	sspA	SAUR1038 (SAR_RS05190)	0,29	>=5,00	0,87	0,10	>=5,00	0,67	0,00	>=5,00	0,58
>0837	aspC	SAUR1040 (SAR_RS05200)	0,00	1,21	1,04	0,00	1,21	1,04	0,00	1,21	1,04
>0838	ykrP	SAUR1041 (SAR_RS05205)	0,00	0,69	0,59	0,00	0,69	0,59	0,20	0,89	0,79
>0839	Q5HH32	SAUR1042 (SAR_RS05210)	0,00	0,48	0,00	0,00	0,48	0,00	0,00	0,48	0,00
>0840	atl	SAUR1906 (SAR_RS09530)	0,05	1,79	1,39	0,03	1,76	1,37	0,03	1,76	1,37
>0841	UPF0039	SAUR1045 (SAR_RS05225)	0,00	1,61	0,92	0,00	1,61	0,92	0,00	1,61	0,92
>0842	Q5HH29=DUF2538	SAUR1046 (SAR_RS05230)	0,00	2,76	0,42	0,00	2,76	0,42	0,00	2,76	0,42
>0843	tagV=lcpB=ywtF	SAUR1047 (SAR_RS05235)	0,00	>=5,00	0,48	0,00	>=5,00	0,48	0,00	>=5,00	0,48
>0844	fmtA	SAUR1048 (SAR_RS05240)	0,08	4,19	1,42	0,00	4,10	1,34	0,00	4,10	1,34
>0845	qoxD	SAUR1049 (SAR_RS05245)	0,00	0,00	0,00	0,00	0,00	0,00	0,00	0,00	0,00
>0846	qoxC	SAUR1050 (SAR_RS05250)	0,00	0,33	0,00	0,00	0,33	0,00	0,00	0,33	0,00
>0847	qoxB	SAUR1051 (SAR_RS05255)	0,05	0,25	0,10	0,00	0,20	0,05	0,00	0,20	0,05
>0848	qoxA	SAUR1052 (SAR_RS05260)	0,00	0,27	0,36	0,00	0,27	0,36	0,00	0,27	0,36
>0849	iraE	(N/A)	0,00	0,00	0,31	0,00	0,00	0,31	0,00	0,00	0,31
>0850	folD	SAUR1056 (SAR_RS05280)	0,00	1,63	0,93	0,00	1,63	0,93	0,12	1,74	1,05
>0851	purE	SAUR1057 (SAR_RS05285)	0,00	1,04	1,86	0,00	1,04	1,86	0,00	1,04	1,86
>0852	purK	SAUR1058 (SAR_RS05290)	0,00	>=5,00	>=5,00	0,05	>=5,00	>=5,00	0,00	>=5,00	>=5,00
>0853	purC	SAUR1059 (SAR_RS05295)	0,00	4,26	0,57	0,00	4,26	0,57	0,00	4,26	0,57
>0854	purS=yexA	SAUR1060 (SAR_RS05300)	0,00	0,38	0,00	0,00	0,38	0,00	0,00	0,38	0,00
>0855	purQ	SAUR1061 (SAR_RS05305)	0,00	2,07	0,59	0,00	2,07	0,59	0,00	2,07	0,59
>0856	purL	SAUR1062 (SAR_RS05310)	0,05	1,05	0,59	0,00	1,00	0,55	0,05	1,05	0,59
>0857	purF	SAUR1063 (SAR_RS05315)	0,00	1,21	0,88	0,00	1,21	0,88	0,00	1,21	0,88
>0858	purM	SAUR1064 (SAR_RS05320)	0,10	1,65	0,87	0,10	1,65	0,87	0,10	1,65	0,87
>0859	purN	SAUR1065 (SAR_RS05325)	0,00	0,88	0,18	0,00	0,88	0,18	0,00	0,88	0,18
>0860	purH	SAUR1066 (SAR_RS05330)	0,00	0,61	0,20	0,00	0,61	0,20	0,00	0,61	0,20
>0861	purD	SAUR1067 (SAR_RS05335)	0,16	0,80	0,48	0,16	0,80	0,48	0,24	0,88	0,56
>0862	ykoC	SAUR1068 (SAR_RS05340)	0,00	1,36	0,12	0,00	1,36	0,12	0,00	1,36	0,12
>0863	ykoD	SAUR1069 (SAR_RS05345)	0,07	1,00	0,64	0,00	0,93	0,57	0,00	0,93	0,57
>0864	ykoE	SAUR1070 (SAR_RS05350)	0,00	1,04	0,17	0,00	1,04	0,17	0,17	1,22	0,35
>0865	graF	SAUR1072 (SAR_RS05360)	0,00	0,74	0,00	0,00	0,74	0,00	0,00	0,74	0,00
>0866	Q5HH05	SAUR1073 (SAR_RS05365)	0,00	0,46	0,31	0,00	0,46	0,31	0,00	0,46	0,31
>0867	ywbD	SAUR1074 (SAR_RS05370)	0,00	1,11	0,09	0,00	1,11	0,09	0,00	1,11	0,09
>0868	Q5HH03=DUF0697	SAUR1075 (SAR_RS05375)	0,00	>=5,00	0,92	0,00	>=5,00	0,92	0,00	>=5,00	0,92
>0869	ptsH	SAUR1076 (SAR_RS05380)	0,00	0,37	0,00	0,00	0,37	0,00	0,00	0,37	0,00
>0870	ptsI=ptsIP	SAUR1077 (SAR_RS05385)	0,00	0,70	0,47	0,00	0,70	0,47	0,00	0,70	0,47
>0871	nrdH	SAUR1078 (SAR_RS05390)	0,00	0,85	1,28	0,00	0,85	1,28	0,00	0,85	1,28
>0872	cydA	SAUR1079 (SAR_RS05395)	0,15	0,66	0,73	0,15	0,66	0,73	0,15	0,66	0,73
>0873	cydB	SAUR1080 (SAR_RS05400)	0,00	0,88	0,69	0,00	0,88	0,69	>=5,00	>=5,00	>=5,00
>0874	ktrA	SAUR1081 (SAR_RS05405)	0,15	0,45	0,45	0,15	0,45	0,45	0,15	0,45	0,45
>0875	rnjA	SAUR1083 (SAR_RS05415)	0,00	0,59	0,12	0,00	0,59	0,12	0,00	0,59	0,12
>0876	rnpZA	SAUR1084 (SAR_RS05420)	0,00	0,00	0,46	0,00	0,00	0,46	0,00	0,00	0,46
>0877	defB=def1	SAUR1086 (SAR_RS05430)	0,00	1,45	1,45	0,18	1,63	1,63	0,18	1,63	1,63
>0878	ykyA	SAUR1087 (SAR_RS05435)	0,16	3,51	0,48	0,00	3,35	0,32	0,00	3,35	0,32
>0879	pdhA	SAUR1088 (SAR_RS05440)	0,00	0,45	0,00	0,00	0,45	0,00	0,00	0,45	0,00
>0880	pdhB	SAUR1089 (SAR_RS05445)	0,20	0,82	0,31	0,00	0,61	0,10	0,00	0,61	0,10
>0881	pdhC	SAUR1090 (SAR_RS05450)	0,00	1,24	0,15	0,08	1,31	0,23	0,08	1,31	0,23
>0882	pdhD=ptsIP	SAUR1091 (SAR_RS05455)	0,00	0,50	0,21	0,00	0,50	0,21	0,00	0,50	0,21
>0883	UPF0223	SAUR1092 (SAR_RS05460)	0,00	0,72	0,00	0,00	0,72	0,00	0,00	0,72	0,00
>0884	puuR	SAUR1093 (SAR_RS05465)	0,00	0,56	0,37	0,00	0,56	0,37	0,00	0,56	0,37
>0885	potA	SAUR1094 (SAR_RS05470)	0,00	0,91	0,37	0,00	0,91	0,37	0,00	0,91	0,37
>0886	potB	SAUR1095 (SAR_RS05475)	0,00	1,13	0,25	0,00	1,13	0,25	0,00	1,13	0,25
>0887	potC	SAUR1096 (SAR_RS05480)	0,12	0,25	0,25	0,12	0,25	0,25	0,00	0,12	0,12
>0888	potD	SAUR1097 (SAR_RS05485)	0,00	1,68	1,30	0,00	1,68	1,30	0,00	1,68	1,30
>0889	Q5HGY1=DUF4064	SAUR1098 (SAR_RS05490)	0,00	1,08	0,20	0,00	1,08	0,20	0,00	1,08	0,20
>0890	mntH	SAUR1100 (SAR_RS05500)	0,07	1,33	0,30	0,07	1,33	0,30	0,15	1,40	0,37
>0891	yktB=DUF1054	SAUR2987	0,00	0,00	0,00	0,00	0,00	0,00	0,00	0,00	0,00
>0892	suhB1	SAUR1102 (SAR_RS05510)	0,24	4,61	0,00	0,24	4,61	0,00	0,24	4,61	0,00
>0893	ylaF	SAUR1103 (SAR_RS05515)	0,52	0,52	0,52	0,00	0,00	0,00	0,52	0,52	0,52
>0894	typA	SAUR1104 (SAR_RS05520)	0,05	0,86	0,43	0,05	0,86	0,43	0,16	0,97	0,54
>0895	ylaL	SAUR1106 (SAR_RS05530)	0,00	0,62	0,00	0,21	0,83	0,21	0,00	0,62	0,00
>0896	ylaN	SAUR1107 (SAR_RS05535)	0,00	0,72	0,00	0,00	0,72	0,00	0,00	0,72	0,00
>0897	ftsW2	SAUR1108 (SAR_RS05540)	0,00	1,22	0,49	0,00	1,22	0,49	0,00	1,22	0,49
>0898	pycA	SAUR1109 (SAR_RS05545)	0,03	0,58	0,29	0,09	0,58	0,35	0,09	0,58	0,35
>0899	ctaA	SAUR1110 (SAR_RS05550)	0,00	0,11	0,00	0,00	0,11	0,00	0,00	0,11	0,00
>0900	ctaB	SAUR1111 (SAR_RS05555)	0,00	0,22	0,11	0,11	0,33	0,22	0,00	0,22	0,11
>0901	ctaM=yozB	SAUR1112 (SAR_RS05560)	0,00	0,87	0,22	0,22	1,08	0,43	0,22	1,08	0,43
>0902	ylbC2	(N/A)	0,00	0,67	0,11	0,00	0,67	0,11	0,00	0,67	0,11
>0903	ylbF	SAUR1115 (SAR_RS05575)	0,00	1,38	0,46	0,00	1,38	0,46	0,23	1,61	0,69
>0904	yhdW=ugpQ-chr	SAUR1116 (SAR_RS05580)	0,00	2,41	4,40	0,00	2,41	4,40	0,00	2,41	4,40
>0905	ylbG	SAUR1117 (SAR_RS05585)	0,00	0,39	0,39	0,00	0,39	0,39	0,00	0,39	0,39
>0906	Q5HGW1	SAUR1118 (SAR_RS05590)	0,00	0,26	0,51	0,00	0,26	0,51	0,00	0,26	0,51
>0907	rsmD	SAUR1119 (SAR_RS05595)	0,00	0,92	0,55	0,00	0,92	0,55	0,00	0,92	0,55
>0908	coaD	SAUR1120 (SAR_RS05600)	0,00	0,62	1,04	0,00	0,62	1,04	0,00	0,62	1,04
>0909	ylbM	SAUR1121 (SAR_RS05605)	0,00	2,02	0,61	0,00	2,02	0,61	0,00	2,02	0,61
>0910	ylbN=Q4L5E0	SAUR1122 (SAR_RS05610)	0,00	0,54	0,18	0,00	0,54	0,18	0,00	0,54	0,18
>0911	rpmF	SAUR1124 (SAR_RS05620)	0,00	0,00	0,00	0,00	0,00	0,00	0,00	0,00	0,00
>0912	isdB	SAUR1125 (SAR_RS05625)	0,00	3,46	1,24	>=5,00	>=5,00	>=5,00	>=5,00	>=5,00	>=5,00
>0913	isdA	SAUR1126 (SAR_RS05630)	0,09	4,32	1,22	0,19	4,41	1,31	0,28	4,51	1,41
>0914	isdC	SAUR1127 (SAR_RS05635)	>=5,00	1,61	0,29	>=5,00	1,90	0,58	>=5,00	1,90	0,29
>0915	isdD	SAUR1128 (SAR_RS05640)	0,00	0,83	0,93	0,00	0,83	0,93	0,09	0,93	1,02
>0916	isdE	SAUR1129 (SAR_RS05645)	0,11	0,57	0,23	0,11	0,57	0,23	0,11	0,57	0,23
>0917	isdF	(N/A)	0,00	1,55	0,83	0,00	1,55	0,83	0,00	1,55	0,83
>0918	srtB	SAUR2988 (srtB)	0,14	0,54	0,27	0,14	0,54	0,27	0,14	0,54	0,27
>0919	isdG	SAUR1132 (SAR_RS05660)	0,00	1,23	0,31	0,00	1,23	0,31	0,00	1,23	0,31
>0920	spoU	SAUR1134 (SAR_RS05670)	0,00	1,21	0,54	0,13	1,35	0,67	0,13	1,35	0,67
>0921	pheS	SAUR1135 (SAR_RS05675)	0,00	0,47	0,28	0,00	0,47	0,28	0,00	0,47	0,28
>0922	pheT	SAUR1136 (SAR_RS05680)	0,04	1,58	0,71	0,04	1,58	0,71	0,08	1,62	0,75
>0923	rnhC	SAUR1137 (SAR_RS05685)	0,11	1,60	0,43	0,11	1,60	0,43	0,11	1,60	0,43
>0924	zapA	SAUR1138 (SAR_RS05690)	0,00	0,37	0,75	0,00	0,37	0,75	0,00	0,37	0,75
>0925	yshB	SAUR1139 (SAR_RS05695)	0,00	0,57	0,77	0,19	0,77	0,96	0,00	0,57	0,77

Number/ Position in the figures	Gene ID	ID in PubMLST cg scheme	ED133 vs. swan isolate 15V8707	X22 vs. swan isolate 15V8707	CC522 goat isolate 17CS1042 vs. swan isolate 15V8707	ED133 vs. duck isolate V315	X22 vs. duck isolate V315	CC522 goat isolate 17CS1042 vs. duck isolate V315	ED133 vs. duck isolate V482	X22 vs. duck isolate V482	CC522 goat isolate 17CS1042 vs. duck isolate V482
>0926	polX	SAUR1140 (SAR_RS05700)	0,00	0,70	0,76	0,00	0,70	0,76	0,00	0,70	0,76
>0927	mutS2	SAUR1141 (SAR_RS05705)	0,09	0,81	0,60	0,00	0,81	0,60	0,00	0,81	0,60
>0928	trxA1	SAUR1142 (SAR_RS05710)	0,00	0,63	0,00	0,00	0,63	0,00	0,00	0,63	0,00
>0929	uvrC	SAUR1144 (SAR_RS05720)	0,11	2,08	0,79	0,00	1,96	0,67	0,06	2,02	0,73
>0930	sdhC	SAUR1145 (SAR_RS05725)	0,00	0,49	0,33	0,33	0,81	0,65	0,16	0,65	0,49
>0931	sdhA	SAUR1146 (SAR_RS05730)	0,00	0,45	0,40	0,00	0,45	0,40	0,00	0,45	0,40
>0932	sdhB	SAUR1147 (SAR_RS05735)	0,25	1,23	1,59	0,12	1,10	1,47	0,12	1,10	1,47
>0933	murI	SAUR1148 (SAR_RS05740)	0,00	1,12	0,12	0,00	1,12	0,12	0,12	1,25	0,25
>0934	ntpA	SAUR1149 (SAR_RS05745)	0,00	0,85	0,85	0,00	0,85	0,85	0,00	0,85	0,85
>0935	ysnB	SAUR1150 (SAR_RS05750)	0,00	0,79	0,60	0,00	0,79	0,60	0,00	0,79	0,60
>0936	ecb	SAUR1152 (SAR_RS05760)	0,00	3,64	>=5,00	0,00	3,64	>=5,00	0,00	3,64	>=5,00
>0937	flr	(N/A)	0,25	>=5,00	>=5,00	>=5,00	>=5,00	0,00	0,00	>=5,00	>=5,00
>0938	Q2YXB9	SAUR1155 (SAR_RS05775)	0,00	0,39	1,18	0,00	0,39	1,18	0,00	0,39	1,18
>0939	efb=fib	SAUR1156 (SAR_RS05780)	0,00	2,01	1,61	0,00	2,01	1,61	0,00	2,01	1,61
>0940	scc	SAUR1157 (SAR_RS05785)	0,00	>=5,00	0,28	0,00	>=5,00	0,28	0,00	>=5,00	0,28
>0941	A5IS45	SAUR1158 (SAR_RS05790)	0,00	1,07	1,07	>=5,00	>=5,00	>=5,00	0,00	1,07	1,07
>0942	Q6GHS5	(N/A)	0,00	>=5,00	0,40	0,40	>=5,00	0,80	0,00	>=5,00	0,40
>0943	A5IS47	0	0,00	0,85	0,85	0,00	0,85	0,85	0,00	0,85	0,85
>0944	hla	SAUR1161 (SAR_RS05805)	0,00	0,63	0,42	0,00	0,63	0,42	0,00	0,63	0,42
>0945	A6U0Y3	SAUR1162 (SAR_RS05810)	0,00	1,36	0,68	0,00	1,36	0,68	0,00	1,36	0,68
>0946	Q2FZB4	SAUR1163 (SAR_RS05815)	0,00	0,51	0,51	0,00	0,51	0,51	0,00	0,51	0,51
>0947	setB3	SAUR1165 (SAR_RS05825)	0,00	1,12	0,56	0,00	1,12	0,56	0,00	1,12	0,56
>0948	setB2	SAUR1166 (SAR_RS05830)	0,00	1,79	>=5,00	0,00	1,79	>=5,00	0,00	1,79	>=5,00
>0949	setB1	SAUR1167 (SAR_RS05835)	0,00	1,24	0,96	0,00	1,24	0,96	0,00	1,24	0,96
>0950	arcB-L1	SAUR1168 (SAR_RS05840)	0,00	1,40	>=5,00	0,00	1,40	>=5,00	0,00	1,40	>=5,00
>0951	arcC-L1	SAUR1169 (SAR_RS05845)	>=5,00	>=5,00	>=5,00	0,00	0,32	0,43	0,00	0,32	0,43
>0952	arcD-L1	SAUR1170 (SAR_RS05850)	0,00	1,03	0,64	0,00	1,03	0,64	0,00	1,03	0,64
>0953	A5IS57	SAUR1171 (SAR_RS05855)	0,00	1,75	1,75	0,00	1,75	1,75	0,00	1,75	1,75
>0954	per	SAUR1172 (SAR_RS05860)	0,00	1,69	0,63	0,00	1,69	0,63	0,00	1,69	0,63
>0955	psmB1	SAUR1175 (psmβ1)	0,00	1,48	0,74	0,00	1,48	0,74	0,00	1,48	0,74
>0956	Q1Y2B3=yfnB	SAUR1176 (SAR_RS05880)	0,29	1,15	0,57	0,43	1,29	0,72	0,43	1,29	0,72
>0957	Q931T2	SAUR1177 (SAR_RS05885)	0,23	0,00	0,45	0,23	0,00	0,45	0,23	0,00	0,45
>0958	bshC	SAUR1178 (SAR_RS05890)	0,06	0,68	0,50	0,06	0,68	0,50	0,12	0,74	0,56
>0959	mraZ	SAUR1179 (SAR_RS05895)	0,00	>=5,00	0,23	0,00	>=5,00	0,23	0,00	>=5,00	0,23
>0960	mraW=rsmH	(N/A)	0,00	0,43	2,46	0,00	0,43	2,46	0,00	0,43	2,46
>0961	ftsL	(N/A)	0,00	0,00	0,00	0,00	0,00	0,00	0,00	0,00	0,00
>0962	pbpA=ftsI1	SAUR1182 (SAR_RS05910)	0,00	0,54	0,31	0,09	0,63	0,40	0,09	0,63	0,40
>0963	mraY	SAUR1183 (SAR_RS05915)	0,00	0,83	0,83	0,00	0,83	0,83	0,00	0,83	0,83
>0964	murD	SAUR1184 (SAR_RS05920)	0,00	0,81	0,30	0,00	0,81	0,30	0,00	0,81	0,30
>0965	ftsQ=divIB	SAUR1185 (SAR_RS05925)	0,00	0,76	0,23	0,00	0,76	0,23	0,00	0,76	0,23
>0966	ftsA	SAUR1186 (SAR_RS05930)	0,00	0,78	0,14	0,00	0,78	0,14	0,00	0,78	0,14
>0967	ftsZ	SAUR1187 (SAR_RS05935)	0,00	0,51	0,00	0,00	0,51	0,00	0,00	0,51	0,00
>0968	ylmD=pgeF	SAUR1188 (SAR_RS05940)	0,00	0,76	0,51	0,00	0,76	0,51	0,00	0,76	0,51
>0969	ylmE=yggS	SAUR1189 (SAR_RS05945)	0,00	0,44	0,15	0,00	0,44	0,15	0,00	0,44	0,15
>0970	sepF	SAUR1190 (SAR_RS05950)	0,00	0,00	0,18	0,00	0,00	0,18	0,00	0,00	0,18
>0971	ylmG	SAUR1191 (SAR_RS05955)	0,00	1,03	0,69	0,00	1,03	0,69	0,00	1,03	0,69
>0972	ylmH	SAUR1192 (SAR_RS05960)	0,00	0,74	0,37	0,12	0,87	0,50	0,00	0,74	0,37
>0973	divIVA	SAUR1193 (SAR_RS05965)	0,00	0,49	0,81	>=5,00	>=5,00	>=5,00	>=5,00	>=5,00	>=5,00
>0974	ileS	SAUR1194 (SAR_RS05970)	0,00	0,73	0,69	0,07	0,80	0,76	0,07	0,80	0,76
>0975	PF00903=catE	SAUR1196 (SAR_RS05980)	0,00	0,75	0,38	0,00	0,75	0,38	0,13	0,88	0,50
>0976	lspA	SAUR1197 (SAR_RS05985)	0,20	0,41	0,00	0,20	0,41	0,00	0,20	0,41	0,00
>0977	ylyB	SAUR1198 (SAR_RS05990)	0,00	0,76	0,22	0,00	0,76	0,22	0,00	0,76	0,22
>0978	pyrR	SAUR1199 (SAR_RS05995)	0,19	1,33	0,38	0,19	1,33	0,38	0,19	1,33	0,38
>0979	pyrP	SAUR1200 (SAR_RS06000)	0,00	1,07	0,61	0,00	1,07	0,61	>=5,00	>=5,00	>=5,00
>0980	pyrB	SAUR1201 (SAR_RS06005)	0,23	0,45	0,23	0,11	0,34	0,11	0,23	0,45	0,23
>0981	pyrC	SAUR1202 (SAR_RS06010)	0,00	0,55	0,55	0,00	0,55	0,55	0,00	0,55	0,55
>0982	pyrAA	SAUR1203 (SAR_RS06015)	0,09	0,18	0,27	0,09	0,18	0,27	0,18	0,27	0,36
>0983	pyrAB	SAUR1204 (SAR_RS06020)	0,00	0,82	0,25	0,00	0,82	0,25	0,00	0,82	0,25
>0984	pyrF	SAUR1205 (SAR_RS06025)	0,00	1,44	0,58	0,00	1,44	0,58	0,00	1,44	0,58
>0985	pyrE	SAUR1206 (SAR_RS06030)	0,00	0,49	>=5,00	0,00	0,49	>=5,00	0,00	0,49	>=5,00
>0986	Q5HGM6	SAUR1207 (SAR_RS06035)	0,00	0,47	0,47	0,00	0,47	0,47	0,00	0,47	0,47
>0987	Q5HGM5	SAUR1208 (SAR_RS06040)	0,00	1,49	0,25	0,00	1,49	0,25	0,00	1,49	0,25
>0988	fbpA	SAUR1209 (SAR_RS06045)	0,06	1,18	0,59	0,06	1,18	0,59	0,06	1,18	0,59
>0989	gmk	SAUR1210 (SAR_RS06050)	0,00	1,12	0,32	0,00	1,12	0,32	0,00	1,12	0,32
>0990	rpoZ	SAUR1211 (SAR_RS06055)	0,00	0,46	0,00	0,00	0,46	0,00	0,00	0,46	0,00
>0991	coaBC	SAUR1212 (SAR_RS06060)	0,00	0,58	0,50	0,00	0,58	0,50	0,00	0,58	0,50
>0992	priA	SAUR1213 (SAR_RS06065)	0,04	0,95	0,54	0,04	0,95	0,54	0,04	0,95	0,54
>0993	Q5HGL9	SAUR1214 (SAR_RS06070)	0,00	1,85	>=5,00	0,00	1,85	>=5,00	0,00	1,85	>=5,00
>0994	Q5HGL8=xynA	SAUR1215 (SAR_RS06075)	>=5,00	>=5,00	>=5,00	0,00	3,58	0,36	0,00	3,58	0,36
>0995	defA=def2	SAUR1216 (SAR_RS06080)	0,00	0,61	1,23	0,00	0,61	1,23	0,00	0,61	1,23
>0996	fmt	SAUR1217 (SAR_RS06085)	0,00	0,85	1,07	0,11	0,96	1,18	0,00	0,85	1,07
>0997	rsmB=sun	SAUR1218 (SAR_RS06090)	0,00	1,53	0,92	0,00	1,53	0,92	0,08	1,61	0,99
>0998	rlmN	SAUR1219 (SAR_RS06095)	0,00	0,37	0,37	0,00	0,37	0,37	0,00	0,37	0,37
>0999	prpC=stp1	SAUR1220 (SAR_RS06100)	0,00	0,67	0,00	0,13	0,81	0,13	0,13	0,81	0,13
>1000	prkC=pknB	SAUR1221 (SAR_RS06105)	0,00	1,30	0,40	0,05	1,35	0,45	0,05	1,35	0,45
>1001	cpgA=engC=rsgA	SAUR1222 (SAR_RS06110)	0,11	1,26	0,46	0,23	1,37	0,57	0,34	1,48	0,68
>1002	rpe	SAUR1223 (SAR_RS06115)	0,16	1,09	0,47	0,00	0,93	0,31	0,00	0,93	0,31
>1003	thiN	SAUR1224 (SAR_RS06120)	0,00	0,47	0,16	0,16	0,62	0,31	0,00	0,47	0,16
>1004	rpmB	SAUR1225 (SAR_RS06125)	0,00	0,00	0,00	0,00	0,00	0,00	0,00	0,00	0,00
>1005	yloU	SAUR1226 (SAR_RS06130)	0,00	0,00	0,00	0,00	0,00	0,00	0,00	0,00	0,00
>1006	yloV=fakA	SAUR1227 (SAR_RS06135)	0,06	0,97	0,67	0,00	0,91	0,61	0,12	1,03	0,73
>1007	recG	SAUR1228 (SAR_RS06140)	0,05	1,12	0,73	0,05	1,12	0,73	0,05	1,12	0,73
>1008	fapR	SAUR1229 (SAR_RS06145)	0,00	0,36	0,54	0,00	0,36	0,54	0,00	0,36	0,54
>1009	plsX	SAUR1230 (SAR_RS06150)	0,00	0,71	0,30	0,00	0,71	0,30	0,10	0,81	0,41
>1010	fabD	SAUR1231 (SAR_RS06155)	0,00	1,94	0,22	0,11	2,05	0,32	0,00	1,94	0,22
>1011	fabG1	SAUR1232 (SAR_RS06160)	0,00	0,54	0,82	0,00	0,54	0,82	0,14	0,68	0,95
>1012	acpP=hmrB	(N/A)	0,00	0,00	0,00	0,00	0,00	0,00	0,00	0,00	0,00
>1013	rnc	SAUR1235 (SAR_RS06175)	0,00	0,41	0,27	0,00	0,41	0,27	0,00	0,41	0,27
>1014	smc	SAUR1236 (SAR_RS06180)	0,03	1,09	0,28	0,08	1,15	0,34	0,06	1,12	0,31
>1015	ftsY	SAUR1237 (SAR_RS06185)	0,00	0,48	0,72	0,08	0,56	0,80	0,16	0,64	0,88
>1016	ylxM	SAUR1238 (SAR_RS06190)	0,00	1,20	0,90	0,00	1,20	0,90	0,00	1,20	0,90
>1017	ffh	SAUR1239 (SAR_RS06195)	0,07	0,88	0,15	0,07	0,88	0,15	0,07	0,88	0,15
>1018	rpsP	SAUR1240 (SAR_RS06200)	0,00	0,00	0,00	0,00	0,00	0,00	0,00	0,00	0,00
>1019	rimM	SAUR1241 (SAR_RS06205)	0,00	4,56	0,60	0,00	4,56	0,60	0,00	4,56	0,60
>1020	trmD	SAUR1242 (SAR_RS06210)	0,00	4,07	1,08	0,00	4,07	1,08	0,00	4,07	1,08
>1021	rplS	SAUR1243 (SAR_RS06215)	0,00	0,28	0,57	0,00	0,28	0,57	0,00	0,28	0,57
>1022	yfhO	SAUR1245 (SAR_RS06225)	0,11	1,61	0,73	0,08	1,57	0,69	0,08	1,57	0,69
>1023	rbgA=ylqF	SAUR1246 (SAR_RS06230)	0,00	1,69	1,02	0,00	1,69	1,02	>=5,00	>=5,00	>=5,00
>1024	rnhB	SAUR1247 (SAR_RS06235)	0,00	4,30	1,04	0,00	4,30	1,04	0,00	4,30	1,04
>1025	sucC	SAUR1248 (SAR_RS06240)	0,00	0,69	0,43	0,00	0,69	0,43	0,00	0,69	0,43
>1026	sucD	SAUR1249 (SAR_RS06245)	0,00	0,55	0,22	0,11	0,66	0,33	0,11	0,66	0,33
>1027	dprA	(N/A)	0,00	>=5,00	0,45	0,23	>=5,00	0,68	0,00	>=5,00	0,45
>1028	topA	SAUR1253 (SAR_RS06265)	0,00	0,87	0,53	0,00	0,87	0,53	0,00	0,87	0,53
>1029	trmFO=gid	SAUR1254 (SAR_RS06270)	0,08	1,07	0,00	0,08	1,07	0,00	0,08	1,07	0,00
>1030	xerC	SAUR1255 (SAR_RS06275)	0,11	0,67	0,11	0,11	0,67	0,11	0,11	0,67	0,11
>1031	hslV	SAUR1256 (SAR_RS06280)	0,00	0,37	0,00	0,00	0,37	0,00	0,00	0,37	0,00
>1032	hslU	SAUR1257 (SAR_RS06285)	0,00	0,71	0,14	0,00	0,71	0,14	0,00	0,71	0,14
>1033	codY	SAUR1258 (SAR_RS06290)	0,00	1,03	0,39	0,00	1,03	0,39	0,00	1,03	0,39
>1034	rpsB	SAUR1260 (SAR_RS06300)	0,00	0,52	0,13	0,00	0,52	0,13	0,13	0,65	0,26
>1035	tsf	SAUR1262 (SAR_RS06310)	0,00	0,11	0,45	0,00	0,11	0,45	0,00	0,11	0,45
>1036	pyrH	SAUR1263 (SAR_RS06315)	0,00	0,55	0,41	0,00	0,55	0,41	0,00	0,55	0,41
>1037	frr	SAUR1264 (SAR_RS06320)	0,00	0,90	0,18	0,00	0,90	0,18	0,00	0,90	0,18
>1038	uppS	SAUR1265 (SAR_RS06325)	0,00	0,65	0,39	0,00	0,65	0,39	0,00	0,65	0,39
>1039	cdsA	SAUR1266 (SAR_RS06330)	0,00	0,51	0,26	0,00	0,51	0,26	0,00	0,51	0,26
>1040	rseP	SAUR1267 (SAR_RS06335)	0,08	1,09	0,23	0,08	1,09	0,23	0,08	1,09	0,23
>1041	proS	SAUR1268 (SAR_RS06340)	0,00	1,35	0,41	0,00	1,35	0,41	0,00	1,35	0,41
>1042	polC	SAUR1269 (SAR_RS06345)	0,00	1,07	0,30	0,07	1,14	0,37	0,02	1,09	0,32
>1043	rimP	SAUR1270 (SAR_RS06350)	0,00	0,21	0,21	0,00	0,21	0,21	0,21	0,43	0,43
>1044	nusA	SAUR1271 (SAR_RS06355)	>=5,00	0,34	0,26	>=5,00	0,34	0,26	>=5,00	0,34	0,26
>1045	ylxR	SAUR1272 (SAR_RS06360)	0,00	0,35	0,70	0,00	0,35	0,70	0,00	0,35	0,70
>1046	ylxQ	SAUR1273 (SAR_RS06365)	0,00	0,94	0,31	0,00	0,94	0,31	0,00	0,94	0,31
>1047	infB	SAUR1274 (SAR_RS06370)	0,00	0,47	0,14	0,05	0,52	0,19	0,09	0,57	0,24
>1048	rbfA	SAUR1275 (SAR_RS06375)	0,00	0,28	0,00	0,00	0,28	0,00	0,00	0,28	0,00
>1049	truB	SAUR1276 (SAR_RS06380)	0,11	4,25	0,65	0,00	4,14	0,54	0,00	4,14	0,54
>1050	ribF	SAUR1277 (SAR_RS06385)	0,31	1,03	0,62	0,21	0,93	0,51	0,21	0,93	0,51
>1051	rpsO	SAUR1278 (SAR_RS06390)	0,00	0,37	0,00	0,00	0,37	0,00	0,74	1,11	0,74
>1052	pnpA	SAUR1279 (SAR_RS06395)	0,05	0,76	0,38	0,10	0,81	0,43	0,10	0,81	0,43
>1053	rnjB	SAUR1280 (SAR_RS06400)	0,00	0,48	0,06	0,06	0,54	0,12	0,00	0,48	0,06
>1054	ftsK=spoIIIE	SAUR1281 (SAR_RS06405)	0,00	0,62	0,40	0,04	0,67	0,45	0,04	0,67	0,45
>1055	ymfC	SAUR1282 (SAR_RS06410)	0,00	0,28	0,70	0,00	0,28	0,70	0,00	0,28	0,70
>1056	ymfF	SAUR1283 (SAR_RS06415)	0,08	1,03	0,55	0,00	0,95	0,47	0,00	0,95	0,47
>1057	ymfH	SAUR1284 (SAR_RS06420)	0,00	1,17	>=5,00	0,08	1,24	>=5,00	0,16	1,32	>=5,00
>1058	fabG2	SAUR1285 (SAR_RS06425)	0,00	0,99	0,43	0,00	0,99	0,43	0,00	0,99	0,43
>1059	ymfK=DUF3388	SAUR1286 (SAR_RS06430)	0,00	0,97	0,97	0,00	0,97	0,97	0,00	0,97	0,97
>1060	ymfM	SAUR1287 (SAR_RS06435)	0,00	0,51	0,51	0,00	0,51	0,51	0,00	0,51	0,51
>1061	pgsA	SAUR1288 (SAR_RS06440)	0,00	0,52	0,35	0,00	0,52	0,35	0,00	0,52	0,35
>1062	cinA	SAUR1289 (SAR_RS06445)	0,00	0,87	0,87	0,00	0,87	0,87	0,00	0,87	0,87
>1063	recA	SAUR1290 (SAR_RS06450)	0,00	0,19	0,19	0,00	0,19	0,19	0,00	0,19	0,19
>1064	rny	SAUR1291 (SAR_RS06455)	0,00	0,26	0,06	0,00	0,26	0,06	0,00	0,26	0,06
>1065	Q5HGE4	SAUR1292 (SAR_RS06460)	0,00	0,00	0,00	0,00	0,00	0,00	0,00	0,00	0,00
>1066	ymdB	SAUR1293 (SAR_RS06465)	0,13	0,63	0,75	0,13	0,63	0,75	0,13	0,63	0,75
>1067	porA	SAUR1294 (SAR_RS06470)	0,00	0,68	0,57	0,00	0,68	0,57	0,00	0,68	0,57
>1068	porB	SAUR1295 (SAR_RS06475)	0,00	1,27	0,58	0,00	1,27	0,58	0,12	1,38	0,69
>1069	DUF0077	SAUR1296 (SAR_RS06480)	0,34	0,68	0,00	0,34	0,68	0,00	0,34	0,68	0,00
>1070	miaB	SAUR1297 (SAR_RS06485)	0,00	1,49	0,13	0,00	1,49	0,13	0,06	1,55	0,19
>1071	ymcA	SAUR1298 (SAR_RS06490)	0,00	0,82	0,27	0,00	0,82	0,27	0,00	0,82	0,27
>1072	thiW	SAUR1299 (SAR_RS06495)	0,41	0,81	0,20	0,20	0,61	0,00	0,20	0,61	0,00
>1073	mutS	SAUR1300 (SAR_RS06500)	0,00	1,07	0,61	0,04	1,11	0,65	0,04	1,11	0,65

Number/ Position in the figures	Gene ID	ID in PubMLST cg scheme	ED133 vs. swan isolate 15V8707	X22 vs. swan isolate 15V8707	CC522 goat isolate 17CS1042 vs. swan isolate 15V8707	ED133 vs. duck isolate V315	X22 vs. duck isolate V315	CC522 goat isolate 17CS1042 vs. duck isolate V315	ED133 vs. duck isolate V482	X22 vs. duck isolate V482	CC522 goat isolate 17CS1042 vs. duck isolate V482
>1074	mutL	(N/A)	0,05	0,80	0,40	0,10	0,85	0,45	0,05	0,80	0,40
>1075	glpP	(N/A)	0,00	2,62	0,75	0,00	2,62	0,75	0,37	3,00	1,12
>1076	glpF	SAUR1303 (SAR_RS06515)	0,12	0,85	0,73	0,24	0,98	0,85	0,24	0,98	0,85
>1077	glpK	SAUR1304 (SAR_RS06520)	0,00	0,73	0,40	0,00	0,73	0,40	0,00	0,73	0,40
>1078	glpD	SAUR1305 (SAR_RS06525)	0,00	1,51	0,17	0,00	1,51	0,17	0,00	1,51	0,17
>1079	pldB2	SAUR1306 (SAR_RS06530)	0,11	1,31	0,87	0,00	1,20	0,77	0,00	1,20	0,77
>1080	miaA	SAUR1307 (SAR_RS06535)	0,00	2,35	0,21	0,00	2,35	0,21	0,00	2,35	0,21
>1081	hfq	SAUR1308 (SAR_RS06540)	0,00	0,85	0,43	0,00	0,85	0,43	0,00	0,85	0,43
>1082	gpxA-L1=bsaA1	SAUR1309 (SAR_RS06545)	0,00	1,05	0,42	0,00	1,05	0,42	0,00	1,05	0,42
>1083	ynbA=hflX	SAUR1310 (SAR_RS06550)	0,08	0,56	0,81	0,16	0,65	0,89	0,16	0,65	0,89
>1084	ynbB	SAUR1311 (SAR_RS06555)	0,08	1,69	1,61	0,08	1,69	1,61	0,08	1,69	1,61
>1085	glnR=femC	(N/A)	0,00	0,81	0,00	0,00	0,81	0,00	0,00	0,81	0,00
>1086	glnA	SAUR1313 (SAR_RS06565)	0,00	3,06	0,45	0,00	3,06	0,45	0,07	3,13	0,52
>1087	A6U1C8	(N/A)	0,00	2,02	2,02	0,00	2,02	2,02	0,00	2,02	2,02
>1088	Q2FYY4	(N/A)	0,00	2,22	3,56	>=5,00	>=5,00	>=5,00	>=5,00	>=5,00	>=5,00
>1089	A5ISJ3	(N/A)	0,00	>=5,00	0,48	0,00	>=5,00	0,48	0,00	>=5,00	0,48
>1090	Q2YXQ4	(N/A)	0,00	0,00	0,00	0,00	0,00	0,00	0,00	0,00	0,00
>1091	A6QGL8	SAUR1352 (SAR_RS06760)	0,10	1,66	1,36	0,10	1,66	1,36	0,10	1,66	1,36
>1092	A5ISJ9	(N/A)	0,00	2,02	1,01	0,00	2,02	1,01	0,00	2,02	1,01
>1093	cls1	SAUR1354 (SAR_RS06770)	0,00	>=5,00	0,54	0,00	>=5,00	0,54	0,00	>=5,00	0,54
>1094	Q99UF3	SAUR1359 (SAR_RS06795)	0,00	0,53	2,12	0,00	0,53	2,12	0,00	0,53	2,12
>1095	nuc2=nucH	SAUR1360 (SAR_RS06800)	0,00	0,37	0,56	0,00	0,37	0,56	0,00	0,37	0,56
>1096	Q5HG96	SAUR1361 (SAR_RS06805)	0,00	0,70	0,23	0,00	0,70	0,23	0,00	0,70	0,23
>1097	Q5HG95	SAUR1362 (SAR_RS06810)	0,34	0,85	1,02	0,17	0,68	0,85	0,17	0,68	0,85
>1098	thrA	SAUR1363 (SAR_RS06815)	0,00	0,65	0,43	0,00	0,65	0,43	0,00	0,65	0,43
>1099	hom=dhoM	SAUR1364 (SAR_RS06820)	0,00	0,55	0,55	0,00	0,55	0,55	0,00	0,55	0,55
>1100	thrC	SAUR1365 (SAR_RS06825)	0,00	0,94	>=5,00	0,00	0,94	>=5,00	0,00	0,94	>=5,00
>1101	thrB	SAUR1366 (SAR_RS06830)	0,00	1,75	0,98	0,00	1,75	0,98	0,00	1,75	0,98
>1102	yxeH	SAUR1367 (SAR_RS06835)	0,00	2,24	2,24	0,00	2,24	2,24	0,00	2,24	2,24
>1103	Q5HG88	SAUR1368 (SAR_RS06840)	0,00	0,32	0,00	0,00	0,32	0,00	0,00	0,32	0,00
>1104	lysP2=Q5HG87	SAUR1370 (SAR_RS06850)	0,07	0,69	0,76	0,07	0,69	0,76	0,07	0,69	0,76
>1105	katA	SAUR1371 (SAR_RS06855)	0,07	1,91	0,13	0,07	1,91	0,13	0,07	1,91	0,13
>1106	rpmG	SAUR1372 (SAR_RS06860)	0,00	0,00	0,66	0,00	0,00	0,66	0,66	0,66	1,32
>1107	rpsN	SAUR1373 (SAR_RS06865)	0,00	>=5,00	0,00	0,00	>=5,00	0,00	0,00	>=5,00	0,00
>1108	guaC	SAUR1374 (SAR_RS06870)	0,00	0,61	0,10	0,00	0,61	0,10	0,00	0,61	0,10
>1109	ylbC1	SAUR1376 (SAR_RS06880)	0,00	1,85	1,27	0,00	1,85	1,27	0,00	1,85	1,27
>1110	lexA	SAUR1377 (SAR_RS06885)	0,00	1,12	0,48	0,00	1,12	0,48	0,00	1,12	0,48
>1111	Q5HG79	(N/A)	0,00	1,28	0,85	0,00	1,28	0,85	0,00	1,28	0,85
>1112	DUF896	SAUR1379 (SAR_RS06895)	0,00	0,00	0,41	0,00	0,00	0,41	0,41	0,41	0,83
>1113	tkt	SAUR1380 (SAR_RS06900)	0,00	1,16	0,15	0,00	1,16	0,15	0,10	1,26	0,25
>1114	UPF0154	(N/A)	0,00	0,00	0,00	0,00	0,00	0,00	0,00	0,00	0,00
>1115	ccdC	SAUR1383 (SAR_RS06915)	0,00	0,21	0,00	0,21	0,43	0,21	0,21	0,43	0,21
>1116	sbcD	SAUR1384 (SAR_RS06920)	0,09	1,43	1,07	0,09	1,43	1,07	0,18	1,52	1,16
>1117	sbcC	(N/A)	0,00	1,65	0,73	0,00	1,65	0,73	0,03	1,68	0,76
>1118	mscL	SAUR1388 (SAR_RS06940)	0,00	0,00	0,28	0,00	0,00	0,28	0,00	0,00	0,28
>1119	opuD1	SAUR1389 (SAR_RS06945)	0,00	0,97	0,91	0,06	1,03	0,97	0,18	1,15	1,09
>1120	acnA=citB	SAUR1390 (SAR_RS06950)	0,00	0,78	0,33	0,07	0,85	0,41	0,07	0,85	0,41
>1121	yneP	SAUR1391 (SAR_RS06955)	1,27	1,48	1,27	0,00	0,21	0,00	0,21	0,42	0,21
>1122	yneR	SAUR1392 (SAR_RS06960)	0,00	1,01	0,00	0,00	1,01	0,00	0,00	1,01	0,00
>1123	plsY	SAUR1393 (SAR_RS06965)	0,00	0,83	0,33	0,00	0,83	0,33	0,00	0,83	0,33
>1124	grlB=gyrB=parE	SAUR1394 (SAR_RS06970)	0,00	0,95	0,55	0,00	0,95	0,55	0,00	0,95	0,55
>1125	grlA=parC	SAUR1395 (SAR_RS06975)	0,08	0,50	0,54	0,04	0,54	0,50	0,04	0,54	0,50
>1126	alsT	SAUR1397 (SAR_RS06985)	0,00	>=5,00	>=5,00	0,00	>=5,00	>=5,00	0,00	>=5,00	>=5,00
>1127	glcT	SAUR1398 (SAR_RS06990)	0,00	1,29	0,82	0,12	1,41	0,94	0,12	1,41	0,94
>1128	yubA=cozEb=yhhT2	SAUR1400 (SAR_RS07000)	0,00	0,83	0,08	0,00	0,83	0,08	0,00	0,83	0,08
>1129	mprF	SAUR1401 (SAR_RS07005)	0,08	3,01	1,11	0,08	3,01	1,11	0,12	3,05	1,15
>1130	msrA1	SAUR1402 (SAR_RS07010)	0,00	0,39	0,00	0,20	0,59	0,20	0,00	0,39	0,00
>1131	lcpA=tagT=msrR	SAUR1403 (SAR_RS07015)	0,00	0,41	0,10	0,00	0,41	0,10	>=5,00	>=5,00	>=5,00
>1132	dmpI	SAUR1405 (SAR_RS07025)	0,00	0,00	0,54	0,00	0,00	0,54	0,00	0,00	0,54
>1133	uvrX	SAUR1406 (SAR_RS07030)	0,00	1,03	1,11	0,16	1,19	1,27	0,24	1,27	1,35
>1134	tyrA	SAUR1407 (SAR_RS07035)	0,00	1,01	1,19	0,00	1,01	1,19	0,00	1,01	1,19
>1135	yhfE	SAUR1408 (SAR_RS07040)	0,00	0,78	0,48	0,10	0,87	0,58	0,00	0,78	0,48
>1136	trpE	SAUR1409 (SAR_RS07045)	0,07	2,63	1,07	0,00	2,56	0,99	0,07	2,63	1,07
>1137	trpG	SAUR1410 (SAR_RS07050)	0,00	>=5,00	>=5,00	0,00	>=5,00	>=5,00	0,00	>=5,00	>=5,00
>1138	trpD	(N/A)	0,00	3,90	3,20	0,00	3,90	3,20	0,00	3,90	3,20
>1139	trpC	SAUR1412 (SAR_RS07060)	0,13	2,55	1,78	0,25	2,68	1,91	0,13	2,55	1,78
>1140	trpF	(N/A)	0,16	1,90	0,16	0,00	2,05	0,00	0,00	2,05	0,00
>1141	trpB	SAUR1414 (SAR_RS07070)	>=5,00	>=5,00	>=5,00	>=5,00	>=5,00	>=5,00	>=5,00	>=5,00	>=5,00
>1142	trpA	SAUR1415 (SAR_RS07075)	0,00	1,23	0,69	0,00	1,23	0,69	0,00	1,23	0,69
>1143	femA	SAUR1416 (SAR_RS07080)	0,00	3,30	3,30	3,07	0,38	0,38	3,07	0,38	0,38
>1144	femB	SAUR1417 (SAR_RS07085)	0,08	0,56	0,79	0,00	0,48	0,71	0,00	0,48	0,71
>1145	cof	(N/A)	0,00	>=5,00	2,45	0,00	>=5,00	2,45	0,00	>=5,00	2,45
>1146	Q5HG42	SAUR1419 (SAR_RS07095)	0,00	1,90	1,27	0,00	1,90	1,27	0,00	1,90	1,27
>1147	nikF	SAUR1420 (SAR_RS07100)	0,00	1,85	0,57	0,28	2,14	0,85	0,28	2,14	0,85
>1148	nikD=gsiA	SAUR1421 (SAR_RS07105)	0,00	2,33	0,26	0,00	2,33	0,26	0,00	2,33	0,26
>1149	nikC	SAUR1422 (SAR_RS07110)	0,00	2,28	0,24	0,00	2,28	0,24	0,12	2,40	0,36
>1150	nikB	SAUR1423 (SAR_RS07115)	0,00	1,62	0,30	0,00	1,62	0,30	0,00	1,62	0,30
>1151	Q5HG37	SAUR1425 (SAR_RS07125)	0,00	1,16	1,16	0,00	1,16	1,16	0,00	1,16	1,16
>1152	pepF2	SAUR1426 (SAR_RS07130)	0,00	0,72	0,44	0,00	0,72	0,44	0,06	0,77	0,50
>1153	phoU	SAUR1427 (SAR_RS07135)	0,00	0,78	0,93	0,00	0,78	0,93	0,00	0,78	0,93
>1154	pstB	SAUR1428 (SAR_RS07140)	0,00	1,76	0,23	0,12	1,88	0,35	0,12	1,88	0,35
>1155	pstA	SAUR1429 (SAR_RS07145)	0,00	0,65	0,65	0,00	0,65	0,65	0,00	0,65	0,65
>1156	pstC	SAUR1430 (SAR_RS07150)	0,00	1,19	1,40	0,00	1,19	1,40	0,00	1,19	1,40
>1157	pstS	SAUR1431 (SAR_RS07155)	0,20	0,71	0,81	0,10	0,61	0,71	0,30	0,81	0,91
>1158	cvfB	SAUR1432 (SAR_RS07160)	0,00	1,77	0,55	0,11	1,88	0,66	0,11	1,88	0,66
>1159	ykpA	SAUR1433 (SAR_RS07165)	0,00	4,24	0,50	0,00	4,24	0,50	0,12	4,37	0,62
>1160	lysC	SAUR1434 (SAR_RS07170)	0,00	0,75	0,41	0,00	0,75	0,41	0,00	0,75	0,41
>1161	asd	SAUR1435 (SAR_RS07175)	0,10	3,33	0,30	0,10	3,33	0,30	0,10	3,33	0,30
>1162	dapA	SAUR1436 (SAR_RS07180)	0,00	3,27	2,25	0,00	3,27	2,25	0,00	3,27	2,25
>1163	dapB	SAUR1437 (SAR_RS07185)	0,00	2,77	0,69	0,14	2,90	0,83	0,14	2,90	0,83
>1164	dapD	SAUR1438 (SAR_RS07190)	0,14	1,81	0,83	0,14	1,81	0,83	0,14	1,81	0,83
>1165	hipO=yxeP	SAUR1439 (SAR_RS07195)	0,00	1,04	0,61	0,00	1,04	0,61	0,00	1,04	0,61
>1166	alr2	SAUR1440 (SAR_RS07200)	0,18	1,38	1,01	0,00	1,20	0,83	0,00	1,20	0,83
>1167	lysA	SAUR1441 (SAR_RS07205)	0,08	1,34	0,79	0,08	1,34	0,79	0,08	1,34	0,79
>1168	cspC-L2=msaB=cspA	(N/A)	0,00	0,00	0,00	0,00	0,00	0,00	0,50	0,50	0,50
>1169	msaA	SAUR1445 (SAR_RS07225)	0,00	0,32	0,32	0,00	0,32	0,32	0,00	0,32	0,32
>1170	acyP	SAUR1446 (SAR_RS07230)	0,00	0,37	0,37	0,00	0,37	0,37	0,00	0,37	0,37
>1171	xpaC	SAUR1447 (SAR_RS07235)	0,00	0,79	0,48	0,00	0,79	0,48	0,00	0,79	0,48
>1172	terA	SAUR1448 (SAR_RS07240)	0,09	0,88	0,44	0,09	0,88	0,44	0,00	0,79	0,35
>1173	brnQ3=braB	SAUR1449 (SAR_RS07245)	0,00	0,97	1,19	0,07	1,04	1,26	0,07	1,04	1,26
>1174	cobT=norD	SAUR2989	0,00	1,43	1,06	0,00	1,43	1,06	0,00	1,43	1,06
>1175	norQ	SAUR1451 (SAR_RS07255)	0,00	1,39	1,01	0,00	1,39	1,01	0,00	1,39	1,01
>1176	yozC=DUF6501	SAUR1452 (SAR_RS07260)	0,00	1,47	0,00	0,00	1,47	0,00	0,00	1,47	0,00
>1177	Q5HG08	SAUR1453 (SAR_RS07265)	0,25	1,98	3,83	0,00	1,73	3,58	0,00	1,73	3,58
>1178	odhB=sucB	SAUR1454 (SAR_RS07270)	0,00	1,26	0,32	0,00	1,26	0,32	0,00	1,26	0,32
>1179	odhA	SAUR1455 (SAR_RS07275)	0,00	0,86	0,93	0,00	0,86	0,93	0,00	0,86	0,93
>1180	arlS	SAUR1456 (SAR_RS07280)	1,31	2,55	2,04	0,00	1,24	0,73	0,00	1,24	0,73
>1181	arlR	SAUR1457 (SAR_RS07285)	0,00	1,21	0,61	0,00	1,21	0,61	0,00	1,21	0,61
>1182	pgpB	SAUR1459 (SAR_RS07295)	0,00	0,65	0,81	0,00	0,65	0,81	0,00	0,65	0,81
>1183	murG	SAUR1460 (SAR_RS07300)	0,09	0,75	0,84	0,09	0,75	0,84	0,19	0,84	0,93
>1184	Q7A0W4	SAUR1461 (SAR_RS07305)	0,00	0,20	0,98	0,00	0,20	0,98	0,00	0,20	0,98
>1185	ctpA	SAUR1462 (SAR_RS07310)	0,00	1,68	0,80	0,00	1,68	0,80	0,00	1,68	0,80
>1186	yozE=DUF1250	SAUR1465 (SAR_RS07325)	0,00	0,45	0,00	0,00	0,45	0,00	0,00	0,45	0,00
>1187	crr	SAUR1466 (SAR_RS07330)	0,00	2,00	0,40	0,00	2,00	0,40	0,00	2,00	0,40
>1188	msrB	SAUR1467 (SAR_RS07335)	0,00	1,17	0,00	0,00	1,17	0,00	0,00	1,17	0,00
>1189	msrA2	SAUR1468 (SAR_RS07340)	0,19	0,19	0,19	0,37	0,37	0,37	0,19	0,19	0,19
>1190	degV2=fakB2	SAUR1469 (SAR_RS07345)	0,00	1,43	0,00	0,00	1,43	0,00	0,00	1,43	0,00
>1191	folA	SAUR1470 (SAR_RS07350)	0,00	0,63	0,21	0,00	0,63	0,21	0,00	0,63	0,21
>1192	thyA-chr	SAUR1471 (SAR_RS07355)	0,00	1,15	0,52	0,00	1,15	0,52	0,10	1,25	0,63
>1193	cvfC4	SAUR1472 (SAR_RS07360)	0,00	4,64	1,32	0,00	4,64	1,32	0,00	4,64	1,32
>1194	cvfC3	SAUR1473 (SAR_RS07365)	0,00	1,51	>=5,00	0,00	1,51	>=5,00	0,09	1,60	>=5,00
>1195	cvfC2	SAUR1474 (SAR_RS07370)	0,00	1,19	0,79	0,00	1,19	0,79	0,00	1,19	0,79
>1196	cvfC1	SAUR1475 (SAR_RS07375)	0,00	0,51	0,51	0,51	1,03	1,03	0,00	0,51	0,51
>1197	Q5HFZ1=yhhQ	SAUR1476 (SAR_RS07380)	0,28	1,42	1,13	0,14	1,28	0,99	0,14	1,28	0,99
>1198	rnhA	SAUR1478 (SAR_RS07390)	0,25	>=5,00	>=5,00	0,50	>=5,00	>=5,00	0,50	>=5,00	>=5,00
>1199	ebh	(N/A)	>=5,00	1,09	>=5,00	>=5,00	1,09	>=5,00	>=5,00	1,09	>=5,00
>1200	norB	SAUR1480 (SAR_RS07400)	0,00	1,15	0,57	0,00	1,15	0,57	0,00	1,15	0,57
>1201	steT	SAUR1481 (SAR_RS07405)	0,00	1,13	1,06	0,00	1,13	1,06	0,00	1,13	1,06
>1202	ilvA1=tdcB	SAUR1482 (SAR_RS07410)	0,10	>=5,00	0,58	0,00	>=5,00	0,48	0,00	>=5,00	0,48
>1203	ald1	SAUR1483 (SAR_RS07415)	0,09	1,16	0,71	0,09	1,16	0,71	0,09	1,16	0,71
>1204	exo53	SAUR1484 (SAR_RS07420)	0,00	1,37	0,80	0,00	1,37	0,80	0,00	1,37	0,80
>1205	Q2FGZ8	SAUR2990	0,00	1,34	0,87	0,06	1,39	0,93	0,06	1,39	0,93
>1206	piuB	(N/A)	0,00	1,57	0,89	0,00	1,57	0,89	0,00	1,57	0,89
>1207	Q5HFY0=DUF4889	SAUR1487 (SAR_RS07435)	0,00	2,40	0,90	0,30	2,70	1,20	0,30	2,70	1,20
>1208	ypsC	SAUR1488 (SAR_RS07440)	0,09	1,22	0,87	0,09	1,22	0,87	0,09	1,22	0,87
>1209	gpsB	SAUR1489 (SAR_RS07445)	0,00	0,87	0,00	0,00	0,87	0,00	0,29	1,16	0,29
>1210	ypsA	SAUR1490 (SAR_RS07450)	0,00	0,71	0,53	0,00	0,71	0,53	0,00	0,71	0,53
>1211	yppE=DUF1798	SAUR1491 (SAR_RS07455)	0,00	1,71	0,85	0,00	1,71	0,85	0,00	1,71	0,85
>1212	recU	SAUR1493 (SAR_RS07465)	0,00	0,48	0,00	0,00	0,48	0,00	0,00	0,48	0,00
>1213	pbpB	SAUR1494 (SAR_RS07470)	0,00	0,69	0,23	0,00	0,69	0,23	0,00	0,69	0,23
>1214	Q1YBU7	SAUR1495 (SAR_RS07475)	0,00	>=5,00	1,17	0,00	>=5,00	1,17	0,00	>=5,00	1,17
>1215	nth1	SAUR1496 (SAR_RS07480)	0,30	0,61	0,30	0,15	0,76	0,15	0,15	0,76	0,15
>1216	dnaD	SAUR1497 (SAR_RS07485)	0,15	1,31	0,15	0,15	1,31	0,15	0,15	1,31	0,15
>1217	asnS	SAUR1498 (SAR_RS07490)	0,00	1,31	0,93	0,00	1,31	0,93	0,08	1,39	1,01
>1218	dinG1	SAUR1499 (SAR_RS07495)	>=5,00	>=5,00	>=5,00	0,00	1,15	0,78	0,00	1,15	0,78
>1219	birA	SAUR1500 (SAR_RS07500)	0,10	1,13	0,72	0,00	1,03	0,62	0,00	1,03	0,62
>1220	cca=papS	SAUR1501 (SAR_RS07505)	0,00	1,66	1,16	0,00	1,66	1,16	0,00	1,66	1,16
>1221	bshA	SAUR1502 (SAR_RS07510)	0,09	0,79	0,79	0,17	0,87	0,87	0,09	0,79	0,79

Number/ Position in the figures	Gene ID	ID in PubMLST cg scheme	ED133 vs. swan isolate 15V8707	X22 vs. swan isolate 15V8707	CC522 goat isolate 17CS1042 vs. swan isolate 15V8707	ED133 vs. duck isolate V315	X22 vs. duck isolate V315	CC522 goat isolate 17CS1042 vs. duck isolate V315	ED133 vs. duck isolate V482	X22 vs. duck isolate V482	CC522 goat isolate 17CS1042 vs. duck isolate V482
>1222	ypjD	SAUR1503 (SAR_RS07515)	0,00	0,63	0,31	0,00	0,63	0,31	0,31	0,94	0,63
>1223	yugP	SAUR1504 (SAR_RS07520)	0,00	0,56	0,56	0,00	0,56	0,56	0,00	0,56	0,56
>1224	ypjA=DUF1405	SAUR1505 (SAR_RS07525)	0,00	>=5,00	>=5,00	0,00	>=5,00	>=5,00	0,00	>=5,00	>=5,00
>1225	ypiB	SAUR1506 (SAR_RS07530)	0,00	1,04	0,87	0,00	1,04	0,87	0,00	1,04	0,87
>1226	ypiA	SAUR1507 (SAR_RS07535)	0,08	0,72	0,88	0,08	0,72	0,88	0,08	0,72	0,88
>1227	aroA	SAUR1508 (SAR_RS07540)	0,00	4,46	0,69	0,00	4,46	0,69	0,00	4,46	0,69
>1228	aroB	SAUR1509 (SAR_RS07545)	0,00	3,38	1,03	0,00	3,47	1,13	0,00	3,38	1,03
>1229	aroC	SAUR1510 (SAR_RS07550)	0,00	>=5,00	0,43	0,09	>=5,00	0,43	0,00	>=5,00	0,43
>1230	ndk	SAUR1511 (SAR_RS07555)	0,00	0,89	0,67	0,00	0,89	0,67	0,00	0,89	0,67
>1231	gerCC=hepT	SAUR1512 (SAR_RS07560)	0,10	1,25	0,94	0,10	1,25	0,94	0,10	1,25	0,94
>1232	ubiE	SAUR1513 (SAR_RS07565)	0,00	1,10	0,41	0,00	1,10	0,41	0,00	1,10	0,41
>1233	gerCA	SAUR1514 (SAR_RS07570)	0,00	1,05	0,87	0,00	1,05	0,87	0,00	1,05	0,87
>1234	hup	(N/A)	0,00	0,00	0,00	0,00	0,00	0,00	0,00	0,00	0,00
>1235	gpdA	SAUR1517 (SAR_RS07585)	0,00	1,40	0,50	0,10	1,50	0,60	0,00	1,40	0,50
>1236	engA=der	SAUR1518 (SAR_RS07590)	0,00	1,30	0,76	0,08	1,37	0,84	0,00	1,30	0,76
>1237	rpsA	SAUR1519 (SAR_RS07595)	0,00	0,60	0,26	0,00	0,60	0,26	0,00	0,60	0,26
>1238	Q2FYF8	SAUR1520 (SAR_RS07600)	0,00	3,13	2,60	0,00	3,13	2,60	0,00	3,13	2,60
>1239	cmk	SAUR1523 (SAR_RS07615)	0,00	>=5,00	0,76	0,00	>=5,00	0,76	0,00	>=5,00	0,76
>1240	ansA	SAUR1524 (SAR_RS07620)	0,00	1,96	1,55	0,00	1,96	1,55	0,00	1,96	1,55
>1241	ypdA=czcO	SAUR1525 (SAR_RS07625)	0,00	0,91	0,10	0,00	0,91	0,10	0,00	0,91	0,10
>1242	ebpS	SAUR2991	0,00	1,16	1,98	0,00	1,16	1,98	0,00	1,16	1,98
>1243	recQ2	SAUR1528 (SAR_RS07640)	0,00	3,26	0,58	0,00	3,26	0,58	0,00	3,26	0,58
>1244	A5IT10	SAUR1529 (SAR_RS07645)	0,00	1,05	2,10	0,00	1,05	2,10	0,00	1,05	2,10
>1245	fer	SAUR1530 (SAR_RS07650)	0,00	0,80	0,00	0,00	0,80	0,00	0,00	0,80	0,00
>1246	ribU	SAUR1531 (SAR_RS07655)	0,00	1,28	0,92	0,00	1,28	0,92	0,00	1,28	0,92
>1247	A5IT17	SAUR1313 (SAR_RS06565)	0,00	>=5,00	>=5,00	0,00	>=5,00	>=5,00	0,00	>=5,00	>=5,00
>1248	srrB	SAUR1605 (SAR_RS08025)	0,00	1,20	1,03	0,00	1,20	1,03	0,00	1,20	1,03
>1249	srrA	SAUR1606 (SAR_RS08030)	0,00	0,13	2,55	0,00	0,13	2,55	0,00	0,13	2,55
>1250	rluB	SAUR1607 (SAR_RS08035)	0,14	3,66	2,44	0,14	3,66	2,44	0,14	3,66	2,44
>1251	scpB	SAUR1608 (SAR_RS08040)	0,00	>=5,00	0,37	0,00	>=5,00	0,37	0,00	>=5,00	0,37
>1252	scpA	SAUR1609 (SAR_RS08045)	0,00	1,23	0,27	0,00	1,23	0,27	0,00	1,23	0,27
>1253	Q2FY75=DUF0309	SAUR1610 (SAR_RS08050)	0,00	0,79	0,39	0,00	0,79	0,39	0,00	0,79	0,39
>1254	xerD	SAUR1611 (SAR_RS08055)	0,11	1,13	0,23	0,23	1,24	0,34	0,23	1,24	0,34
>1255	fur	SAUR1612 (SAR_RS08060)	0,00	0,67	0,22	0,00	0,67	0,22	0,00	0,67	0,22
>1256	nudF	SAUR1613 (SAR_RS08065)	>=5,00	>=5,00	>=5,00	0,00	0,55	0,55	0,00	0,55	0,55
>1257	yhdN	SAUR1614 (SAR_RS08070)	0,11	1,21	0,55	0,00	1,10	0,44	0,00	1,10	0,44
>1258	Q5HFS1	SAUR1615 (SAR_RS08075)	0,00	0,40	0,80	0,00	0,40	0,80	0,00	0,40	0,80
>1259	yqjQ	SAUR2992	0,00	1,85	0,93	0,00	1,85	0,93	0,00	1,85	0,93
>1260	proC	SAUR1617 (SAR_RS08085)	0,00	0,12	0,61	0,00	0,12	0,61	0,00	0,12	0,61
>1261	rnz	SAUR1618 (SAR_RS08090)	0,00	0,54	1,09	0,11	0,65	1,19	0,00	0,54	1,09
>1262	zwf	SAUR1619 (SAR_RS08095)	0,00	1,08	0,74	0,00	1,08	0,74	0,00	1,08	0,74
>1263	graE=marA	(N/A)	0,12	0,23	0,00	0,12	0,23	0,00	0,12	0,23	0,00
>1264	malA=yugT	SAUR1621 (SAR_RS08105)	0,00	1,58	0,73	0,00	1,58	0,73	0,00	1,58	0,73
>1265	malR=kdgR	SAUR1622 (SAR_RS08110)	0,00	2,45	2,65	0,00	2,45	2,65	0,00	2,45	2,65
>1266	Q5HFR3	SAUR1624 (SAR_RS08120)	0,00	1,88	0,81	0,00	1,88	0,81	0,27	2,15	1,08
>1267	Q931R4	(N/A)	0,00	>=5,00	0,00	0,00	>=5,00	0,00	0,00	>=5,00	0,00
>1268	gnd	SAUR1627 (SAR_RS08135)	0,14	0,92	0,64	0,07	0,85	0,57	0,07	0,85	0,57
>1269	Q5HFR1=pepT2	SAUR1628 (SAR_RS08140)	0,00	1,68	1,15	0,00	1,68	1,15	0,00	1,68	1,15
>1270	yqjA	SAUR1630 (SAR_RS08150)	0,00	>=5,00	0,41	0,00	>=5,00	0,41	0,00	>=5,00	0,41
>1271	yqjW=fadE	(N/A)	0,00	>=5,00	>=5,00	>=5,00	0,13	0,13	>=5,00	0,13	0,13
>1272	bfmBB	SAUR1632 (SAR_RS08160)	0,00	3,06	0,08	0,00	3,06	0,08	0,00	3,06	0,08
>1273	bfmBAB	SAUR2994	0,10	4,57	0,71	0,10	4,57	0,71	0,20	4,57	0,71
>1274	bfmBAA	SAUR1634 (SAR_RS08170)	0,10	1,71	0,60	0,00	1,61	0,50	0,00	1,61	0,50
>1275	lpdA	SAUR1635 (SAR_RS08175)	0,00	0,98	0,28	0,00	0,98	0,28	0,00	0,98	0,28
>1276	recN	SAUR1636 (SAR_RS08180)	0,00	0,95	0,60	0,00	0,95	0,60	0,00	0,95	0,60
>1277	ahrC=argR	SAUR1637 (SAR_RS08185)	0,00	0,44	0,44	0,00	0,44	0,44	0,00	0,44	0,44
>1278	ispA	SAUR1638 (SAR_RS08190)	0,00	1,02	1,02	0,00	1,02	1,02	0,11	1,13	1,13
>1279	xseB	SAUR1639 (SAR_RS08195)	0,00	0,43	0,00	0,00	0,43	0,00	0,00	0,43	0,00
>1280	xseA	SAUR1640 (SAR_RS08200)	0,07	0,52	0,30	0,00	0,45	0,22	0,00	0,45	0,22
>1281	nusB	SAUR1641 (SAR_RS08205)	0,00	0,26	0,77	0,00	0,26	0,77	0,00	0,26	0,77
>1282	yqhY	SAUR1642 (SAR_RS08210)	0,00	0,00	0,00	0,00	0,00	0,00	0,00	0,00	0,00
>1283	accC=cfiB	SAUR1643 (SAR_RS08215)	0,00	0,52	0,81	0,00	0,52	0,81	0,00	0,52	0,81
>1284	accB	SAUR1644 (SAR_RS08220)	0,00	0,43	0,43	0,00	0,43	0,43	0,00	0,43	0,43
>1285	efp	SAUR1645 (SAR_RS08225)	0,18	0,18	0,18	0,00	0,00	0,00	0,00	0,00	0,00
>1286	yqhT	SAUR1646 (SAR_RS08230)	0,00	3,64	0,09	0,09	3,73	0,18	0,00	3,64	0,09
>1287	Q5HFM8	SAUR1647 (SAR_RS08235)	0,00	0,17	>=5,00	0,00	0,17	>=5,00	0,00	0,17	>=5,00
>1288	Q5HFM7	SAUR1648 (SAR_RS08240)	0,00	0,91	0,46	0,00	0,91	0,46	0,00	0,91	0,46
>1289	lipM	SAUR1649 (SAR_RS08245)	0,00	0,84	0,48	0,12	0,96	0,60	0,00	0,84	0,48
>1290	yqhL	SAUR1650 (SAR_RS08250)	0,00	0,77	0,00	0,00	0,77	0,00	0,26	1,03	0,26
>1291	gcvPB	SAUR1651 (SAR_RS08255)	0,00	1,43	1,09	0,07	1,49	1,15	0,00	1,43	1,09
>1292	gcvPA	SAUR1652 (SAR_RS08260)	0,00	1,11	1,04	0,07	1,19	1,11	0,07	1,19	1,11
>1293	gcvT	SAUR1653 (SAR_RS08265)	0,00	0,46	0,82	0,00	0,46	0,82	0,00	0,46	0,82
>1294	aroK	SAUR1654 (SAR_RS08270)	0,00	0,95	0,57	0,00	0,95	0,57	0,00	0,95	0,57
>1295	comGF	SAUR1656 (SAR_RS08280)	0,00	>=5,00	>=5,00	0,00	>=5,00	>=5,00	>=5,00	>=5,00	>=5,00
>1296	comGE	SAUR1657 (SAR_RS08285)	0,00	0,67	1,67	0,00	0,67	1,67	0,00	0,67	1,67
>1297	comGD	(N/A)	0,22	3,80	0,45	0,00	3,58	0,22	0,00	3,58	0,22
>1298	comGC	SAUR1659 (SAR_RS08295)	0,00	>=5,00	>=5,00	0,00	>=5,00	>=5,00	0,00	>=5,00	>=5,00
>1299	comGB	SAUR1660 (SAR_RS08300)	0,00	1,12	0,56	0,09	1,21	0,65	0,09	1,21	0,65
>1300	comGA	SAUR1661 (SAR_RS08305)	0,00	0,72	0,62	0,00	0,72	0,62	0,00	0,72	0,62
>1301	yqgX	SAUR1662 (SAR_RS08310)	0,00	0,64	>=5,00	0,00	0,64	>=5,00	0,00	0,64	>=5,00
>1302	yqgV	SAUR1663 (SAR_RS08315)	0,00	0,30	0,91	0,00	0,30	0,91	0,00	0,30	0,91
>1303	glk	SAUR1664 (SAR_RS08320)	0,10	0,41	0,10	0,10	0,41	0,10	0,20	0,41	0,20
>1304	yqgQ	(N/A)	0,00	0,00	0,00	0,00	0,00	0,00	0,00	0,00	0,00
>1305	gluP	SAUR1666 (SAR_RS08330)	0,07	0,55	0,14	0,07	0,55	0,14	0,07	0,55	0,14
>1306	yqgN	(N/A)	0,00	1,11	0,19	0,00	1,11	0,19	0,00	1,11	0,19
>1307	rpmG	SAUR1372 (SAR_RS06860)	0,00	0,00	>=5,00	0,00	0,00	>=5,00	0,00	0,00	>=5,00
>1308	pbpC=pbp3=ftsI3	SAUR1669 (SAR_RS08345)	0,00	0,58	0,67	0,05	0,63	0,72	0,00	0,58	0,67
>1309	sodA-L2	SAUR1670 (SAR_RS08350)	0,00	0,50	0,17	0,00	0,50	0,17	0,00	0,50	0,17
>1310	zur	SAUR1671 (SAR_RS08355)	0,00	0,24	0,00	0,00	0,24	0,00	0,00	0,24	0,00
>1311	znuB=mreB	SAUR1672 (SAR_RS08360)	0,00	0,46	0,46	0,00	0,46	0,46	0,00	0,46	0,46
>1312	znuC	SAUR1673 (SAR_RS08365)	0,00	0,64	0,64	>=5,00	>=5,00	>=5,00	0,13	0,76	0,76
>1313	nfo	SAUR1674 (SAR_RS08370)	0,00	0,56	0,11	0,00	0,56	0,11	0,00	0,56	0,11
>1314	cshB	SAUR1675 (SAR_RS08375)	0,00	0,82	0,22	0,00	0,82	0,22	0,07	0,89	0,30
>1315	ybgI	SAUR1676 (SAR_RS08380)	0,00	0,91	>=5,00	>=5,00	>=5,00	0,09	0,00	0,91	>=5,00
>1316	trmK	SAUR1677 (SAR_RS08385)	0,00	1,62	1,18	0,00	1,62	1,18	0,00	1,62	1,18
>1317	sigA=rpoD	SAUR1678 (SAR_RS08390)	0,00	0,54	0,45	0,00	0,54	0,45	0,00	0,54	0,45
>1318	dnaG-var1800	SAUR1679 (SAR_RS08395)	0,00	1,33	0,56	0,06	1,39	0,61	0,06	1,39	0,61
>1319	yqfL	SAUR1680 (SAR_RS08400)	0,00	1,10	1,10	0,00	1,10	1,10	0,00	1,10	1,10
>1320	ccpN	SAUR1681 (SAR_RS08405)	0,00	0,32	0,32	0,00	0,32	0,32	0,16	0,48	0,48
>1321	glyS	SAUR1682 (SAR_RS08410)	0,07	0,43	0,43	0,00	0,36	0,36	0,00	0,36	0,36
>1322	recO	SAUR1683 (SAR_RS08415)	0,00	1,46	0,66	0,00	1,46	0,66	0,00	1,46	0,66
>1323	era	SAUR1684 (SAR_RS08420)	0,00	0,67	0,33	0,00	0,67	0,33	0,00	0,67	0,33
>1324	cdd	SAUR1685 (SAR_RS08425)	0,00	0,99	0,49	0,00	0,99	0,49	0,00	0,99	0,49
>1325	dgkA	SAUR1686 (SAR_RS08430)	0,00	1,74	1,16	0,00	1,74	1,16	0,00	1,74	1,16
>1326	ybeY	SAUR1687 (SAR_RS08435)	0,00	0,43	0,85	0,00	0,43	0,85	0,00	0,43	0,85
>1327	phoH=ybeZ	SAUR1688 (SAR_RS08440)	0,11	0,42	0,32	0,00	0,32	0,21	0,00	0,32	0,21
>1328	Q5HFI8	SAUR1689 (SAR_RS08445)	0,00	0,72	0,43	0,14	0,86	0,57	0,14	0,86	0,57
>1329	floA	SAUR1690 (SAR_RS08450)	0,00	0,91	1,01	0,00	0,91	1,01	0,00	0,91	1,01
>1330	yqeZ	(N/A)	0,00	4,10	3,53	3,67	0,99	0,42	0,00	4,10	3,53
>1331	rpsU	(N/A)	0,00	0,00	0,00	0,00	0,00	0,00	0,00	0,00	0,00
>1332	mtaB	SAUR1693 (SAR_RS08465)	0,00	0,59	0,00	0,07	0,67	0,07	0,00	0,59	0,00
>1333	rsmE	SAUR1694 (SAR_RS08470)	0,00	1,06	0,13	0,00	1,06	0,13	0,00	1,06	0,13
>1334	prmA	SAUR1695 (SAR_RS08475)	0,00	1,17	0,75	0,00	1,17	0,75	0,00	1,17	0,75
>1335	dnaJ	SAUR1696 (SAR_RS08480)	0,00	0,61	0,26	0,00	0,61	0,26	0,00	0,61	0,26
>1336	dnaK	SAUR1697 (SAR_RS08485)	0,00	0,71	0,33	0,00	0,71	0,33	0,00	0,71	0,33
>1337	grpE	SAUR1698 (SAR_RS08490)	0,00	0,64	0,16	0,00	0,64	0,16	0,00	0,64	0,16
>1338	hrcA	SAUR1699 (SAR_RS08495)	0,00	0,00	0,00	0,10	0,10	0,10	0,00	0,00	0,00
>1339	hemN	SAUR1700 (SAR_RS08500)	0,00	1,04	0,60	0,00	1,04	0,60	0,00	1,04	0,60
>1340	lepA	SAUR1701 (SAR_RS08505)	0,00	1,48	0,88	0,05	1,54	0,93	0,00	1,48	0,88
>1341	rpsT	(N/A)	0,00	0,00	0,00	0,00	0,00	0,00	0,00	0,00	0,00
>1342	holA	SAUR1703 (SAR_RS08515)	0,00	0,72	0,31	0,00	0,72	0,31	0,00	0,72	0,31
>1343	comEC=rec2	SAUR1704 (SAR_RS08520)	0,00	0,86	0,77	0,05	0,91	0,82	0,05	0,91	0,82
>1344	comEB	SAUR1705 (SAR_RS08525)	0,00	0,23	0,00	0,00	0,23	0,00	0,00	0,23	0,00
>1345	comEA	SAUR1706 (SAR_RS08530)	0,15	1,60	0,58	0,15	1,60	0,58	0,15	1,60	0,58
>1346	yqeM=cypM	SAUR1707 (SAR_RS08535)	0,14	0,42	0,00	0,14	0,42	0,00	0,14	0,42	0,00
>1347	rsfS	SAUR1708 (SAR_RS08540)	0,00	0,28	0,00	0,00	0,28	0,00	0,00	0,28	0,00
>1348	yqeK	SAUR1709 (SAR_RS08545)	0,17	1,03	0,85	0,00	0,85	0,68	0,00	0,85	0,68
>1349	nadD	SAUR1710 (SAR_RS08550)	0,00	1,05	1,05	0,00	1,05	1,05	0,00	1,05	1,05
>1350	yhbY	SAUR1711 (SAR_RS08555)	0,00	0,69	1,03	0,00	0,69	1,03	0,00	0,69	1,03
>1351	aroE	SAUR1712 (SAR_RS08560)	0,00	1,98	1,49	0,00	1,98	1,49	0,00	1,98	1,49
>1352	yqeH	SAUR1713 (SAR_RS08565)	0,00	0,54	>=5,00	0,00	0,54	>=5,00	0,00	0,54	>=5,00
>1353	yqeG=mtnN	SAUR1714 (SAR_RS08570)	0,19	0,57	0,38	0,19	0,57	0,38	0,19	0,57	0,38
>1354	mtnN	SAUR1715 (SAR_RS08575)	0,43	0,87	1,01	0,43	0,87	1,01	0,58	1,01	1,15
>1355	Q5HFG1	SAUR1717 (SAR_RS08585)	0,00	0,00	0,00	0,00	0,00	0,00	0,00	0,00	0,00
>1356	entX	SAUR1718 (SAR_RS08590)	0,00	>=5,00	0,93	0,13	>=5,00	1,06	0,13	>=5,00	1,06
>1357	ycsG	SAUR1722 (SAR_RS08610)	0,00	0,65	0,49	0,00	0,65	0,49	0,00	0,65	0,49
>1358	ycsF=pxpA	SAUR1723 (SAR_RS08615)	0,00	1,46	0,93	0,00	1,46	0,93	0,00	1,46	0,93
>1359	accC2	SAUR1724 (SAR_RS08620)	0,00	0,73	>=5,00	0,00	0,73	>=5,00	0,00	0,73	>=5,00
>1360	accB2	SAUR1725 (SAR_RS08625)	0,00	0,67	0,44	0,00	0,67	0,44	0,00	0,67	0,44
>1361	kipA	SAUR1726 (SAR_RS08630)	0,00	1,29	0,99	0,00	1,29	0,99	0,10	1,38	1,09
>1362	kipI=pxpB	SAUR1727 (SAR_RS08635)	0,14	1,63	1,50	0,00	1,50	1,36	0,00	1,50	1,36
>1363	greA	SAUR1728 (SAR_RS08640)	0,00	0,42	0,42	0,00	0,42	0,42	0,00	0,42	0,42
>1364	udk	SAUR1729 (SAR_RS08645)	0,00	0,16	0,16	0,00	0,16	0,16	0,00	0,16	0,16
>1365	yrrO	SAUR1730 (SAR_RS08650)	0,08	0,63	0,24	0,08	0,63	0,24	0,08	0,63	0,24
>1366	yrrN	SAUR1731 (SAR_RS08655)	0,00	0,32	0,22	0,00	0,32	0,22	0,00	0,32	0,22
>1367	yrrM	SAUR1732 (SAR_RS08660)	0,00	0,31	0,47	0,00	0,31	0,47	0,00	0,31	0,47
>1368	yrzB	SAUR1733 (SAR_RS08665)	0,00	0,65	0,65	0,00	0,65	0,65	0,00	0,65	0,65
>1369	yrrK=ruvX	SAUR1734 (SAR_RS08670)	0,00	0,23	0,00	0,00	0,23	0,00	0,00	0,23	0,00

Number/ Position in the figures	Gene ID	ID in PubMLST cg scheme	ED133 vs. swan isolate 15V8707	X22 vs. swan isolate 15V8707	CC522 goat isolate 17CS1042 vs. swan isolate 15V8707	ED133 vs. duck isolate V315	X22 vs. duck isolate V315	CC522 goat isolate 17CS1042 vs. duck isolate V315	ED133 vs. duck isolate V482	X22 vs. duck isolate V482	CC522 goat isolate 17CS1042 vs. duck isolate V482
>1370	yrzL	SAUR1735 (SAR_RS08675)	0,00	0,77	0,00	0,00	0,77	0,00	0,00	0,77	0,00
>1371	alaS	SAUR1736 (SAR_RS08680)	0,00	0,42	0,19	0,04	0,46	0,23	0,04	0,46	0,23
>1372	recD	SAUR1737 (SAR_RS08685)	0,04	1,45	>=5,00	0,00	1,41	>=5,00	0,00	1,41	>=5,00
>1373	yrrB	SAUR1738 (SAR_RS08690)	0,00	1,49	0,45	0,00	1,49	0,45	0,00	1,49	0,45
>1374	mnmA=trmU	SAUR1739 (SAR_RS08695)	0,09	0,63	0,36	0,09	0,63	0,36	0,09	0,63	0,36
>1375	csd1	SAUR1740 (SAR_RS08700)	0,17	0,79	0,00	0,17	0,79	0,00	0,17	0,79	0,00
>1376	limB2	SAUR1741 (SAR_RS08705)	0,00	1,48	0,20	0,00	1,48	0,20	0,00	1,48	0,20
>1377	Q5HFD8	SAUR1742 (SAR_RS08710)	0,68	2,04	2,04	0,00	1,36	1,36	0,00	1,36	1,36
>1378	csbD-L2	(N/A)	0,00	0,55	0,55	0,00	0,55	0,55	0,00	0,55	0,55
>1379	cymR	SAUR1744 (SAR_RS08720)	0,00	0,24	0,24	0,00	0,24	0,24	0,00	0,24	0,24
>1380	rarA	SAUR1745 (SAR_RS08725)	0,08	0,78	0,63	0,08	0,78	0,63	0,08	0,78	0,63
>1381	yrvM	SAUR1746 (SAR_RS08730)	0,00	0,65	0,13	0,00	0,65	0,13	0,00	0,65	0,13
>1382	aspS	SAUR1748 (SAR_RS08740)	0,00	1,08	0,68	0,00	1,08	0,68	0,00	1,08	0,68
>1383	hisS	SAUR1749 (SAR_RS08745)	0,00	0,32	0,00	0,00	0,32	0,00	0,00	0,32	0,00
>1384	lytH	SAUR1750 (SAR_RS08750)	0,11	1,03	0,23	0,00	0,91	0,11	0,00	0,91	0,11
>1385	dtd	SAUR1751 (SAR_RS08755)	0,00	1,32	0,44	0,00	1,32	0,44	0,00	1,32	0,44
>1386	relA	SAUR1752 (SAR_RS08760)	1,00	0,90	0,27	1,04	0,95	0,32	1,04	0,95	0,32
>1387	apt	SAUR1753 (SAR_RS08765)	0,19	0,58	0,19	0,00	0,39	0,00	0,00	0,39	0,00
>1388	recJ	SAUR1754 (SAR_RS08770)	0,00	1,45	0,40	0,04	1,50	0,44	0,04	1,50	0,44
>1389	secDF	SAUR1755 (SAR_RS08775)	0,00	0,70	0,26	0,00	0,70	0,26	>=5,00	>=5,00	>=5,00
>1390	yajC	SAUR1756 (SAR_RS08780)	0,00	0,00	0,77	0,00	0,00	0,77	0,00	0,00	0,77
>1391	tgt	SAUR1757 (SAR_RS08785)	0,00	0,26	0,00	0,09	0,35	0,09	0,09	0,35	0,09
>1392	queA	SAUR1758 (SAR_RS08790)	0,00	0,88	0,58	0,00	0,88	0,58	0,00	0,88	0,58
>1393	ruvB	SAUR1759 (SAR_RS08795)	0,10	1,09	0,60	0,00	1,00	0,50	0,20	1,09	0,70
>1394	ruvA	SAUR1760 (SAR_RS08800)	0,00	0,33	0,17	0,00	0,33	0,17	0,00	0,33	0,17
>1395	pheB	SAUR1761 (SAR_RS08805)	0,22	0,22	0,00	0,66	0,66	0,44	0,44	0,44	0,22
>1396	obgE=cgtA	SAUR1762 (SAR_RS08810)	0,00	1,01	0,23	0,00	1,01	0,23	0,08	1,08	0,31
>1397	rpmA	(N/A)	0,00	0,00	0,00	0,00	0,00	0,00	0,00	0,00	0,00
>1398	DUF0464	SAUR1764 (SAR_RS08820)	0,00	0,00	0,00	0,31	0,31	0,31	0,31	0,31	0,31
>1399	rplU	SAUR1765 (SAR_RS08825)	0,00	0,32	0,32	0,00	0,32	0,32	0,00	0,32	0,32
>1400	mreD	SAUR1766 (SAR_RS08830)	0,00	1,32	0,19	0,00	1,32	0,19	0,00	1,32	0,19
>1401	mreC	SAUR2996 (mreC)	0,24	1,30	0,47	0,00	1,07	0,24	0,12	1,19	0,36
>1402	Q5HFB3-DUF4930	SAUR2997	0,00	0,84	0,00	0,00	0,84	0,00	0,00	0,84	0,00
>1403	Q5HFB2	SAUR2998	0,00	1,05	1,40	0,00	1,05	1,40	0,00	1,05	1,40
>1404	radC	(N/A)	0,15	>=5,00	0,29	0,15	>=5,00	0,29	0,15	>=5,00	0,29
>1405	comC=outO	SAUR1778 (SAR_RS08890)	0,28	1,69	0,71	0,14	1,55	0,56	0,14	1,55	0,56
>1406	folC	SAUR1780 (SAR_RS08900)	0,16	0,63	0,24	0,08	0,55	0,16	0,08	0,55	0,16
>1407	valS	SAUR1781 (SAR_RS08905)	0,04	0,72	0,65	0,08	0,76	0,68	0,04	0,72	0,65
>1408	tag	SAUR1782 (SAR_RS08910)	0,00	1,60	1,07	0,00	1,60	1,07	0,18	1,78	1,25
>1409	abrB	SAUR1783 (SAR_RS08915)	0,00	1,49	0,84	0,00	1,49	0,84	0,00	1,49	0,84
>1410	gsaA	SAUR1785 (SAR_RS08925)	0,00	1,17	0,47	0,00	1,17	0,47	0,00	1,17	0,47
>1411	hemB	SAUR1786 (SAR_RS08930)	0,00	1,23	1,13	0,00	1,23	1,13	0,10	1,33	1,23
>1412	hemD	SAUR1787 (SAR_RS08935)	0,00	0,59	1,19	0,15	0,74	1,33	0,15	0,74	1,33
>1413	hemC	SAUR1788 (SAR_RS08940)	0,00	0,43	0,86	0,00	0,43	0,86	0,00	0,43	0,86
>1414	hemX	SAUR1789 (SAR_RS08945)	0,00	0,86	0,49	0,00	0,86	0,49	0,00	0,86	0,49
>1415	hemA1	SAUR1790 (SAR_RS08950)	0,00	0,30	0,52	0,07	0,37	0,59	0,15	0,45	0,67
>1416	engB=yihA	SAUR1791 (SAR_RS08955)	0,00	0,85	0,17	0,00	0,85	0,17	0,00	0,85	0,17
>1417	clpX	SAUR1792 (SAR_RS08960)	0,00	0,63	0,08	0,00	0,63	0,08	0,00	0,63	0,08
>1418	tig	SAUR1793 (SAR_RS08965)	0,15	0,92	0,23	0,15	0,92	0,23	0,15	0,92	0,23
>1419	ysoA	SAUR1795 (SAR_RS08975)	0,00	1,29	0,32	0,00	1,29	0,32	0,00	1,29	0,32
>1420	ymaB	SAUR1796 (SAR_RS08980)	0,00	0,66	0,00	0,16	0,82	0,16	0,00	0,66	0,00
>1421	rplT	SAUR1798 (SAR_RS08990)	>=5,00	>=5,00	>=5,00	0,28	0,84	0,28	0,28	0,84	0,28
>1422	rpmI	SAUR1799 (SAR_RS08995)	0,00	0,00	0,00	0,00	0,00	0,00	0,00	0,00	0,00
>1423	infC	SAUR1800 (SAR_RS09000)	0,00	0,19	0,00	0,00	0,19	0,00	0,00	0,19	0,00
>1424	lysP1	SAUR1801 (SAR_RS09005)	0,00	>=5,00	0,47	0,13	>=5,00	0,60	0,07	>=5,00	0,54
>1425	thrS	SAUR1802 (SAR_RS09010)	0,00	1,65	0,05	0,00	1,65	0,05	0,00	1,65	0,05
>1426	dnaI	SAUR1804 (SAR_RS09020)	0,11	0,33	0,43	0,11	0,33	0,43	0,22	0,43	0,54
>1427	dnaB	SAUR1805 (SAR_RS09025)	0,00	0,79	0,14	0,07	0,86	0,21	0,00	0,79	0,14
>1428	nrdR	SAUR1806 (SAR_RS09030)	0,00	0,42	0,21	0,00	0,42	0,21	0,00	0,42	0,21
>1429	gapB	SAUR1807 (SAR_RS09035)	0,00	0,78	0,29	0,00	0,78	0,29	0,00	0,78	0,29
>1430	coaE	SAUR1808 (SAR_RS09040)	0,00	0,80	0,32	0,00	0,80	0,32	0,16	0,96	0,48
>1431	mutM	SAUR1809 (SAR_RS09045)	0,00	1,37	0,34	0,00	1,37	0,34	0,00	1,37	0,34
>1432	polA	SAUR1810 (SAR_RS09050)	0,08	0,91	0,42	0,00	0,84	0,34	0,04	0,87	0,38
>1433	Q2YTE9=DUF6056	SAUR1811 (SAR_RS09055)	0,07	1,68	0,87	0,13	1,75	0,94	0,13	1,75	0,94
>1434	phoR	SAUR1812 (SAR_RS09060)	0,00	1,68	1,38	0,00	1,68	1,38	0,00	1,68	1,38
>1435	phoP	SAUR1813 (SAR_RS09065)	0,00	0,43	0,28	0,00	0,43	0,28	0,00	0,43	0,28
>1436	citC=icd	SAUR1815 (SAR_RS09075)	0,00	0,79	0,55	0,00	0,79	0,55	0,00	0,79	0,55
>1437	citZ	SAUR1816 (SAR_RS09080)	0,00	0,89	0,53	0,09	0,98	0,62	0,00	0,89	0,53
>1438	cycA=aapA	SAUR1817 (SAR_RS09085)	0,00	0,95	0,59	0,00	0,95	0,59	0,07	1,03	0,66
>1439	pykA	SAUR1818 (SAR_RS09090)	0,00	0,57	0,34	0,00	0,57	0,34	0,00	0,57	0,34
>1440	pfkA	SAUR1819 (SAR_RS09095)	0,00	0,31	0,21	0,00	0,31	0,21	0,00	0,31	0,21
>1441	accA	SAUR1820 (SAR_RS09100)	0,11	1,16	0,32	0,11	1,16	0,32	0,00	1,06	0,21
>1442	accD	SAUR1821 (SAR_RS09105)	0,00	0,70	0,00	0,00	0,70	0,00	0,00	0,70	0,00
>1443	maeB	SAUR1822 (SAR_RS09110)	0,08	0,98	0,49	0,00	0,89	0,41	0,08	0,98	0,41
>1444	dnaE	SAUR1823 (SAR_RS09115)	0,03	3,50	0,78	0,00	3,47	0,75	0,03	3,50	0,78
>1445	nrnA	SAUR1824 (SAR_RS09120)	0,11	2,23	0,96	0,00	2,12	0,85	0,00	2,12	0,85
>1446	ytoI	SAUR1825 (SAR_RS09125)	0,00	1,00	0,92	0,00	1,00	0,92	0,00	1,00	0,92
>1447	uspA	SAUR1826 (SAR_RS09130)	0,00	0,24	0,00	0,00	0,24	0,00	0,00	0,24	0,00
>1448	ytkL	SAUR1827 (SAR_RS09135)	0,00	0,72	1,01	0,00	0,72	1,01	0,00	0,72	1,01
>1449	pepQ	SAUR1828 (SAR_RS09140)	0,00	2,79	2,51	0,00	2,79	2,51	0,00	2,79	2,51
>1450	ald2	SAUR1830 (SAR_RS09150)	0,00	0,54	0,00	0,00	0,54	0,00	0,09	0,63	0,09
>1451	yxiE	SAUR1831 (SAR_RS09155)	0,00	0,60	0,20	0,00	0,60	0,20	0,00	0,60	0,20
>1452	ackA	SAUR1832 (SAR_RS09160)	0,00	0,75	0,33	0,00	0,75	0,33	0,08	0,83	0,42
>1453	ytxK	SAUR1833 (SAR_RS09165)	0,11	0,74	>=5,00	0,11	0,74	>=5,00	0,21	0,84	>=5,00
>1454	tpx	SAUR1834 (SAR_RS09170)	0,00	1,21	0,00	0,00	1,21	0,00	0,00	1,21	0,00
>1455	ytnM	SAUR1835 (SAR_RS09175)	0,13	1,82	0,00	0,13	1,82	0,00	0,13	1,82	0,00
>1456	thiI	SAUR1836 (SAR_RS09180)	0,00	3,35	2,37	0,00	3,35	2,37	0,00	3,35	2,37
>1457	csd2=icsS	SAUR1837 (SAR_RS09185)	0,00	>=5,00	2,46	0,00	>=5,00	2,46	0,00	>=5,00	2,46
>1458	ezrA	SAUR1839 (SAR_RS09195)	0,00	0,88	0,12	0,00	0,88	0,12	0,06	0,94	0,18
>1459	ytsP=msrC	SAUR1840 (SAR_RS09200)	0,00	>=5,00	0,86	0,00	>=5,00	0,86	0,00	>=5,00	0,86
>1460	rpsD	SAUR1841 (SAR_RS09205)	0,00	0,17	0,17	0,17	0,33	0,33	0,17	0,33	0,33
>1461	ugpQ2	SAUR1843 (SAR_RS09215)	0,00	1,08	0,54	0,00	1,08	0,54	0,00	1,08	0,54
>1462	osmC	SAUR1844 (SAR_RS09220)	0,22	1,34	0,45	0,00	1,12	0,22	0,00	1,12	0,22
>1463	pucG	(N/A)	0,09	1,21	0,69	0,00	1,12	0,60	0,00	1,12	0,60
>1464	serA	SAUR1846 (SAR_RS09230)	0,06	0,87	0,62	0,00	0,81	0,56	0,00	0,81	0,56
>1465	Q5HF49	SAUR1848 (SAR_RS09240)	0,09	0,62	0,27	0,09	0,62	0,27	0,09	0,62	0,27
>1466	nagE	SAUR1849 (SAR_RS09245)	0,07	1,50	0,75	0,00	1,43	0,68	0,07	1,50	0,75
>1467	plsC	SAUR1850 (SAR_RS09250)	0,00	0,32	0,97	0,00	0,32	0,97	0,00	0,32	0,97
>1468	htrC	SAUR1851 (SAR_RS09255)	0,00	1,02	0,55	0,00	1,02	0,55	0,00	1,02	0,55
>1469	tyrS	SAUR1852 (SAR_RS09260)	0,08	1,35	1,50	0,08	1,35	1,50	0,08	1,35	1,50
>1470	sgtA	SAUR1853 (SAR_RS09265)	0,00	0,66	0,11	0,00	0,66	0,11	0,11	0,77	0,22
>1471	isdH=harA	SAUR1855 (SAR_RS09275)	0,00	>=5,00	2,37	0,07	>=5,00	2,45	0,04	>=5,00	2,41
>1472	fhs	SAUR1857 (SAR_RS09285)	0,00	3,06	0,48	0,12	3,06	0,60	0,06	3,00	0,54
>1473	acsA1	SAUR2999 (acs)	0,00	0,82	0,35	>=5,00	>=5,00	>=5,00	>=5,00	>=5,00	>=5,00
>1474	acuA	SAUR1859 (SAR_RS09295)	0,00	1,26	0,32	0,00	1,26	0,32	0,00	1,26	0,32
>1475	acuC	SAUR1860 (SAR_RS09300)	0,09	0,77	0,43	0,09	0,77	0,43	0,17	0,85	0,51
>1476	ccpA	SAUR1861 (SAR_RS09305)	0,00	1,41	0,51	0,00	1,41	0,51	0,00	1,41	0,51
>1477	aroA2	SAUR1863 (SAR_RS09315)	0,00	3,75	0,55	0,09	3,85	0,64	0,09	3,85	0,64
>1478	Q99TC6	(N/A)	>=5,00	4,90	1,98	>=5,00	4,90	1,98	>=5,00	4,98	2,05
>1479	yoxC	SAUR1866 (SAR_RS09330)	0,00	0,81	0,20	0,00	0,81	0,20	0,00	0,81	0,20
>1480	murC	SAUR1867 (SAR_RS09335)	0,00	0,30	>=5,00	0,00	0,30	>=5,00	0,00	0,30	>=5,00
>1481	Q5HF33	SAUR1868 (SAR_RS09340)	0,03	1,52	0,68	0,03	1,52	0,68	0,03	1,52	0,68
>1482	pheT2	SAUR1869 (SAR_RS09345)	0,00	0,34	0,84	0,00	0,34	0,84	0,00	0,34	0,84
>1483	Q5HF31=DUF1444	SAUR1870 (SAR_RS09350)	0,12	0,47	0,58	0,12	0,47	0,58	0,12	0,47	0,58
>1484	ytpP	SAUR1871 (SAR_RS09355)	0,00	0,64	0,64	0,00	0,64	0,64	0,00	0,64	0,64
>1485	pepA1	SAUR1872 (SAR_RS09360)	0,09	1,39	0,19	0,09	1,39	0,19	0,09	1,39	0,19
>1486	ytzB	(N/A)	0,00	0,00	0,00	0,00	0,00	0,00	0,32	0,32	0,32
>1487	ytnP	SAUR1874 (SAR_RS09370)	0,00	1,42	0,71	0,12	1,54	0,83	0,12	1,54	0,83
>1488	trmB	SAUR1881 (SAR_RS09405)	0,16	0,78	0,16	0,00	0,62	0,31	0,00	0,62	0,31
>1489	ytmP	SAUR1882 (SAR_RS09410)	0,13	1,77	0,51	0,00	1,77	0,38	0,00	1,77	0,38
>1490	daaA=dat	SAUR1883 (SAR_RS09415)	0,00	0,82	0,47	0,00	0,82	0,47	0,00	0,82	0,47
>1491	ytjP=sapep	SAUR1884 (SAR_RS09420)	0,00	0,71	0,99	0,00	0,71	0,99	0,00	0,71	0,99
>1492	Q5HF22	SAUR1885 (SAR_RS09425)	0,00	0,71	0,24	0,00	0,71	0,24	0,24	0,94	0,47
>1493	rsuA	SAUR1886 (SAR_RS09430)	0,00	0,57	0,43	0,14	0,72	0,57	0,14	0,72	0,57
>1494	ytgP	SAUR1887 (SAR_RS09435)	0,00	1,08	0,24	0,00	1,08	0,24	0,12	1,20	0,36
>1495	Q1Y9Y5	SAUR1888 (SAR_RS09440)	0,00	1,89	0,55	0,00	1,89	0,55	0,00	1,89	0,55
>1496	sasC	SAUR1889 (SAR_RS09445)	0,03	1,94	0,67	0,02	1,92	0,66	0,02	1,92	0,66
>1497	ytwF=moeB	SAUR1890 (SAR_RS09450)	0,00	>=5,00	0,00	0,00	>=5,00	0,00	0,00	>=5,00	0,00
>1498	leuS	SAUR1891 (SAR_RS09455)	0,00	1,95	1,04	0,00	1,95	1,04	0,00	1,95	1,04
>1499	yttB=mdtH	SAUR1892 (SAR_RS09460)	0,00	1,44	1,35	0,00	1,44	1,35	0,08	1,52	1,44
>1500	ytqA	SAUR1893 (SAR_RS09465)	0,00	0,63	0,31	0,10	0,73	0,42	0,10	0,73	0,42
>1501	ytqB	SAUR1894 (SAR_RS09470)	0,00	1,06	0,53	0,00	1,06	0,53	0,00	1,06	0,53
>1502	rot	SAUR1895 (SAR_RS09475)	0,00	0,25	1,49	0,00	0,25	1,49	0,00	0,25	1,49
>1503	pldB1	SAUR1896 (SAR_RS09480)	0,00	0,60	0,12	0,12	0,72	0,24	0,00	0,60	0,12
>1504	putA	SAUR1898 (SAR_RS09490)	0,00	0,80	0,60	0,00	0,80	0,60	0,00	0,80	0,60
>1505	ribH=ribE	SAUR1899 (SAR_RS09495)	0,00	1,08	0,22	0,00	1,08	0,22	0,00	1,08	0,22
>1506	ribA	SAUR1900 (SAR_RS09500)	0,08	0,68	0,08	0,17	0,76	0,17	0,17	0,76	0,17
>1507	ribB	SAUR1901 (SAR_RS09505)	>=5,00	>=5,00	>=5,00	0,00	0,79	0,63	0,00	0,79	0,63
>1508	ribD	SAUR1902 (SAR_RS09510)	0,10	1,15	0,77	0,10	1,15	0,77	0,10	1,15	0,77
>1509	Q5HF04	SAUR1903 (SAR_RS09515)	0,07	1,80	1,33	0,00	1,73	1,26	0,00	1,73	1,26
>1510	arsR	SAUR1904 (SAR_RS09520)	0,00	1,90	0,32	0,00	1,90	0,32	0,00	1,90	0,32
>1511	arsB	SAUR1905 (SAR_RS09525)	0,00	2,40	1,47	0,00	2,40	1,47	0,00	2,40	1,47
>1512	arsC	(N/A)	0,00	>=5,00	0,00	0,00	>=5,00	0,00	0,00	>=5,00	0,00
>1513	sagB=gad	SAUR1906 (SAR_RS09530)	0,12	0,35	0,47	0,23	0,47	0,58	0,23	0,47	0,58
>1514	Q5HEZ9	SAUR1907 (SAR_RS09535)	0,00	1,33	0,89	0,44	1,78	1,33	0,44	1,78	1,33
>1515	sigS	SAUR1908 (SAR_RS09540)	0,00	1,91	1,91	0,00	1,91	1,91	0,85	2,76	2,76
>1516	Q5HEZ7=comK	(N/A)	0,00	1,35	>=5,00	0,00	1,35	>=5,00	0,00	1,35	>=5,00
>1517	Q5HEZ6	SAUR1910 (SAR_RS09550)	0,00	0,68	>=5,00	0,00	0,68	>=5,00	0,00	0,68	>=5,00

Number/ Position in the figures	Gene ID	ID in PubMLST cg scheme	ED133 vs. swan isolate 15V8707	X22 vs. swan isolate 15V8707	CC522 goat isolate 17CS1042 vs. swan isolate 15V8707	ED133 vs. duck isolate V315	X22 vs. duck isolate V315	CC522 goat isolate 17CS1042 vs. duck isolate V315	ED133 vs. duck isolate V482	X22 vs. duck isolate V482	CC522 goat isolate 17CS1042 vs. duck isolate V482
>1518	Q5HEZ5=sdpA_core	(N/A)	0,00	1,10	1,89	0,00	1,10	1,89	0,00	1,10	1,89
>1519	tal	SAUR1913 (SAR_RS09565)	0,00	0,56	0,70	0,00	0,56	0,70	0,14	0,70	0,84
>1520	Q7A500	SAUR1914 (SAR_RS09570)	0,33	2,31	1,65	0,00	1,98	1,32	0,00	1,98	1,32
>1521	crcB1	SAUR1915 (SAR_RS09575)	0,00	1,13	0,45	0,00	1,13	0,45	0,00	1,13	0,45
>1522	crcB2	SAUR1916 (SAR_RS09580)	0,00	2,82	2,26	0,00	2,82	2,26	0,00	2,82	2,26
>1523	ytbE=yvgN	SAUR1917 (SAR_RS09585)	0,00	0,84	1,44	0,00	0,84	1,44	0,00	0,84	1,44
>1524	nrd	SAUR1918 (SAR_RS09590)	0,00	0,99	0,77	0,00	0,99	0,77	0,11	1,10	0,88
>1525	metK	SAUR1919 (SAR_RS09595)	0,00	0,84	0,17	0,00	0,84	0,17	0,00	0,84	0,17
>1526	pckA	SAUR1920 (SAR_RS09600)	0,00	0,69	0,75	0,00	0,69	0,75	0,06	0,75	0,82
>1527	ytmA	SAUR1921 (SAR_RS09605)	0,00	3,63	3,50	0,00	3,63	3,50	0,00	3,63	3,50
>1528	rppH=nudH=ytkD	SAUR1922 (SAR_RS09610)	0,00	0,42	0,42	0,00	0,42	0,42	0,00	0,42	0,42
>1529	ytjA=yidD	SAUR1923 (SAR_RS09615)	0,00	0,39	0,39	0,00	0,39	0,39	0,00	0,39	0,39
>1530	menC	SAUR1924 (SAR_RS09620)	0,00	2,50	2,79	0,00	2,50	2,79	0,00	2,50	2,79
>1531	menE	SAUR1925 (SAR_RS09625)	0,00	2,57	1,42	0,00	2,57	1,42	0,00	2,57	1,42
>1532	Q5HEY1=DUF4909	SAUR1926 (SAR_RS09630)	0,00	1,24	2,48	0,00	1,24	2,48	0,00	1,24	2,48
>1533	Q5HEY0=yokF	SAUR1927 (SAR_RS09635)	0,15	4,61	0,31	0,00	4,45	0,15	0,00	4,45	0,15
>1534	DUF4352	SAUR1928 (SAR_RS09640)	0,00	3,31	1,20	0,10	3,41	1,31	0,00	3,31	1,20
>1535	Q5HEX8	(N/A)	0,00	>=5,00	3,03	0,00	>=5,00	3,03	0,00	>=5,00	3,03
>1536	Q5HEX7=DUF3969	(N/A)	0,00	>=5,00	1,16	0,00	>=5,00	1,16	0,00	>=5,00	1,16
>1537	A5ITW8	(N/A)	0,00	>=5,00	>=5,00	0,00	>=5,00	>=5,00	0,00	>=5,00	>=5,00
>1539	hsdS-spl	(N/A)	0,00	>=5,00	>=5,00	0,00	>=5,00	>=5,00	0,00	>=5,00	>=5,00
>1540	hsdM-spl	(N/A)	1,28	4,69	4,56	0,26	4,05	4,11	0,19	4,11	4,05
>1541	splF	(N/A)	0,00	4,58	2,36	0,00	4,58	2,36	0,00	4,58	2,36
>1543	splD2	(N/A)	0,00	0,00	>=5,00	0,00	0,00	>=5,00	0,00	0,00	>=5,00
>1545	splC	(N/A)	0,00	>=5,00	>=5,00	0,00	>=5,00	>=5,00	0,00	>=5,00	>=5,00
>1546	splB	(N/A)	0,14	>=5,00	2,35	0,00	>=5,00	2,35	0,14	>=5,00	2,49
>1547	splA	(N/A)	0,00	2,54	0,42	0,00	2,54	0,42	0,00	2,54	0,42
>1548	Q2FXC0=ear2	(N/A)	0,00	>=5,00	>=5,00	0,00	>=5,00	>=5,00	0,00	>=5,00	>=5,00
>1549	epiG=bsaG	(N/A)	0,00	2,29	>=5,00	0,00	2,29	>=5,00	0,00	2,29	>=5,00
>1550	epiE	(N/A)	0,00	>=5,00	>=5,00	0,00	>=5,00	>=5,00	0,00	>=5,00	>=5,00
>1551	epiF	(N/A)	0,00	4,62	>=5,00	0,00	4,62	>=5,00	0,00	4,62	>=5,00
>1552	epiP	(N/A)	0,00	4,22	>=5,00	0,00	4,22	>=5,00	0,00	4,22	>=5,00
>1553	epiD	(N/A)	0,00	>=5,00	>=5,00	0,00	>=5,00	>=5,00	0,00	>=5,00	>=5,00
>1554	epiC	(N/A)	0,08	>=5,00	>=5,00	0,08	>=5,00	>=5,00	0,08	>=5,00	>=5,00
>1555	epiB	(N/A)	0,10	3,74	>=5,00	0,07	3,70	>=5,00	0,10	3,74	>=5,00
>1556	epiA=bsA1	(N/A)	0,00	0,00	>=5,00	0,00	0,00	>=5,00	0,00	0,00	>=5,00
>1557	bsaX	(N/A)	0,00	>=5,00	>=5,00	0,00	>=5,00	>=5,00	0,00	>=5,00	>=5,00
>1558	lukD	(N/A)	0,00	1,93	1,73	0,00	1,93	1,73	0,00	1,93	1,73
>1559	lukE	(N/A)	0,00	2,35	>=5,00	1,71	4,06	>=5,00	1,71	4,06	>=5,00
>1560	A6U2S7	(N/A)	0,00	0,84	0,42	0,00	0,84	0,42	0,00	0,84	0,42
>1561	Q7A4X2=DUF1828	(N/A)	0,00	1,54	0,13	0,00	1,54	0,13	0,00	1,54	0,13
>1568	ydeN	SAUR1973 (SAR_RS09865)	0,00	1,62	0,90	0,00	1,62	0,90	0,00	1,62	0,90
>1569	hemY=hemG	SAUR1975 (SAR_RS09875)	>=5,00	2,86	0,79	>=5,00	2,78	0,71	>=5,00	2,86	0,79
>1570	hemH	SAUR1976 (SAR_RS09880)	0,00	1,41	0,65	0,00	1,41	0,65	0,00	1,41	0,65
>1571	hemE	SAUR1977 (SAR_RS09885)	0,00	0,77	0,19	0,10	0,87	0,29	0,10	0,87	0,29
>1572	traP	SAUR1979 (SAR_RS09895)	0,00	>=5,00	0,60	0,00	>=5,00	0,60	0,00	>=5,00	0,60
>1573	ecsB	SAUR1980 (SAR_RS09900)	0,08	2,21	1,14	0,08	2,21	1,14	0,08	2,21	1,14
>1574	ecsA	SAUR1981 (SAR_RS09905)	0,13	1,21	0,27	0,13	1,21	0,27	0,27	1,35	0,40
>1575	hit	SAUR1982 (SAR_RS09910)	0,00	0,24	0,00	0,00	0,24	0,00	0,00	0,24	0,00
>1576	gvpP	SAUR1983 (SAR_RS09915)	0,00	0,27	1,09	0,00	0,27	1,09	0,00	0,27	1,09
>1577	yhaJ	SAUR1984 (SAR_RS09920)	0,00	0,54	1,08	0,00	0,54	1,08	0,00	0,54	1,08
>1578	prsA2	SAUR1985 (SAR_RS09925)	0,00	0,93	0,00	0,00	0,93	0,00	0,00	0,93	0,00
>1579	cbf=yhaM	SAUR1986 (SAR_RS09930)	0,11	0,74	0,64	0,00	0,64	0,53	0,00	0,64	0,53
>1580	Q5HET2	SAUR1987 (SAR_RS09935)	0,00	0,65	0,34	0,00	0,65	0,34	0,03	0,68	0,37
>1581	Q5HET1=yhaO	SAUR1988 (SAR_RS09940)	0,08	0,58	0,25	0,00	0,50	0,17	0,00	0,50	0,17
>1582	UPF0342	SAUR1990 (SAR_RS09950)	0,00	0,29	0,00	0,00	0,29	0,00	0,00	0,29	0,00
>1583	UPF0754=DUF0445	SAUR1991 (SAR_RS09955)	0,00	0,98	0,98	0,09	1,07	1,07	0,09	1,07	1,07
>1584	xdrA	SAUR1992 (SAR_RS09960)	0,00	0,22	0,00	0,00	0,22	0,00	0,00	0,22	0,00
>1585	airR=vraR=yhcR	SAUR1993 (SAR_RS09965)	0,16	0,16	0,16	0,16	0,16	0,16	0,32	0,32	0,32
>1586	airS=vraS=yhcS	SAUR1994 (SAR_RS09970)	0,00	0,81	0,54	0,00	0,81	0,54	0,00	0,81	0,54
>1587	rluA2	SAUR1995 (SAR_RS09975)	0,00	0,49	1,46	0,00	0,49	1,46	0,00	0,49	1,46
>1588	fumC=citG	SAUR1996 (SAR_RS09980)	0,00	0,79	0,29	0,00	0,79	0,29	0,00	0,79	0,29
>1589	Q5HES3	SAUR1997 (SAR_RS09985)	0,00	0,25	0,00	0,00	0,25	0,00	0,00	0,25	0,00
>1590	Q5HES1	SAUR1999 (SAR_RS09995)	0,00	1,31	0,65	0,00	1,31	0,65	0,00	1,31	0,65
>1591	Q5HES0	SAUR2000 (SAR_RS10000)	0,00	0,83	0,17	0,00	0,83	0,17	0,00	0,83	0,17
>1592	cspR=trmL	SAUR2001 (SAR_RS10005)	0,00	0,42	0,21	0,00	0,42	0,21	0,00	0,42	0,21
>1593	queG	SAUR2002 (SAR_RS10010)	0,00	0,98	0,27	0,00	0,98	0,27	0,00	0,98	0,27
>1594	artR=glnQ	SAUR2003 (SAR_RS10015)	0,00	0,69	0,14	0,00	0,69	0,14	0,00	0,69	0,14
>1595	artQ	SAUR2004 (SAR_RS10020)	0,00	0,69	0,34	0,00	0,69	0,34	0,00	0,69	0,34
>1596	Q1Y9N4	SAUR2005 (SAR_RS10025)	0,00	>=5,00	0,47	0,00	>=5,00	0,47	0,09	>=5,00	0,56
>1597	perR	SAUR2036 (SAR_RS10180)	0,00	0,22	0,22	0,22	0,45	0,45	0,22	0,45	0,45
>1598	Q5HER2=ghrB2	SAUR2037 (SAR_RS10185)	0,00	2,00	1,26	0,00	2,00	1,26	0,00	2,00	1,26
>1599	bcp	SAUR2038 (SAR_RS10190)	0,00	0,66	>=5,00	0,00	0,66	>=5,00	0,00	0,66	>=5,00
>1600	gsaB=hemL	SAUR2039 (SAR_RS10195)	0,00	1,24	0,47	0,00	1,24	0,47	0,00	1,24	0,47
>1601	DUF0939-L1	SAUR2040 (SAR_RS10200)	0,00	0,37	0,09	0,00	0,37	0,09	0,00	0,37	0,09
>1602	msbA2	SAUR2042 (SAR_RS10210)	0,06	1,04	0,35	0,12	1,09	0,40	0,06	1,04	0,35
>1603	DUF0402	SAUR2043 (SAR_RS10215)	0,00	0,18	0,00	0,00	0,18	0,00	0,00	0,18	0,00
>1604	mutY	SAUR2044 (SAR_RS10220)	0,10	1,64	0,87	0,10	1,64	0,87	0,10	1,64	0,87
>1605	DUF0457	SAUR2045 (SAR_RS10225)	0,00	0,10	0,00	0,20	0,31	0,20	0,20	0,31	0,20
>1606	tagG	SAUR2047 (SAR_RS10235)	0,24	0,72	0,12	0,24	0,72	0,12	0,24	0,72	0,12
>1607	tagH	SAUR2048 (SAR_RS10240)	0,07	0,53	0,46	0,00	0,59	0,40	0,00	0,59	0,40
>1608	yfhH	SAUR2049 (SAR_RS10245)	0,00	0,32	0,00	0,00	0,32	0,00	0,00	0,32	0,00
>1609	recX	SAUR2050 (SAR_RS10250)	0,00	>=5,00	>=5,00	0,00	>=5,00	>=5,00	0,00	>=5,00	>=5,00
>1610	sgtB	SAUR2051 (SAR_RS10255)	0,12	0,74	0,12	0,00	0,62	0,00	0,12	0,74	0,12
>1611	pfpI	SAUR2052 (SAR_RS10260)	0,00	0,00	0,19	0,00	0,00	0,19	0,00	0,00	0,19
>1612	Q5HEP8	SAUR2053 (SAR_RS10265)	0,00	1,85	0,00	0,00	1,85	0,00	0,00	1,85	0,00
>1613	A5IU43=yfkAB	SAUR2054 (SAR_RS10270)	1,99	0,78	>=5,00	1,99	0,78	>=5,00	2,08	0,87	>=5,00
>1614	A5IU44	SAUR2055 (SAR_RS10275)	0,00	0,94	0,94	0,00	0,94	0,94	0,00	0,94	0,94
>1615	ampS=pepS	SAUR2056 (SAR_RS10280)	0,00	1,04	1,44	0,00	1,04	1,44	0,00	1,04	1,44
>1616	Q5HEP4=DUF1128	SAUR2057 (SAR_RS10285)	0,00	0,97	0,97	0,00	0,97	0,97	0,00	0,97	0,97
>1617	ptpA	SAUR2058 (SAR_RS10290)	0,00	0,22	0,22	0,00	0,22	0,22	0,00	0,22	0,22
>1618	Q5HEP2	SAUR2059 (SAR_RS10295)	0,00	0,36	0,00	0,00	0,36	0,00	0,00	0,36	0,00
>1619	rbn	SAUR2060 (SAR_RS10300)	0,00	0,90	1,23	0,00	0,90	1,23	0,00	0,90	1,23
>1620	vraR	SAUR2061 (SAR_RS10305)	0,00	0,32	0,00	0,00	0,32	0,00	0,00	0,32	0,00
>1621	vraS	SAUR2062 (SAR_RS10310)	0,00	0,29	0,57	0,00	0,29	0,57	0,10	0,38	0,67
>1622	vraT=liaF	SAUR2063 (SAR_RS10315)	0,00	0,43	0,57	0,00	0,43	0,57	0,00	0,43	0,57
>1623	vraU	SAUR2064 (SAR_RS10320)	0,00	0,78	0,52	0,00	0,78	0,52	0,00	0,78	0,52
>1624	ampM	SAUR2065 (SAR_RS10325)	0,00	1,05	0,13	0,00	1,05	0,13	0,00	1,05	0,13
>1625	DUF0939-L2	(N/A)	0,00	1,72	0,81	0,00	1,72	0,81	0,10	1,82	0,91
>1626	Q5HEN4	(N/A)	0,00	3,31	>=5,00	0,00	3,31	>=5,00	0,22	3,53	>=5,00
>1627	gatD	SAUR2069 (SAR_RS10345)	0,00	1,23	0,14	0,00	1,23	0,14	0,00	1,23	0,14
>1628	murT	SAUR2070 (SAR_RS10350)	0,00	0,76	0,61	0,00	0,76	0,61	0,00	0,76	0,61
>1629	ftnA	SAUR2071 (SAR_RS10355)	0,00	0,60	0,40	0,00	0,60	0,40	0,00	0,60	0,40
>1630	dinG2	SAUR2073 (SAR_RS10365)	0,18	0,36	0,36	0,00	0,18	0,18	0,00	0,18	0,18
>1631	dinB	SAUR2074 (SAR_RS10370)	0,00	0,84	0,56	0,00	0,84	0,56	0,00	0,84	0,56
>1632	Q5HEM6=DUF3267	SAUR2075 (SAR_RS10375)	0,00	0,38	0,38	>=5,00	>=5,00	>=5,00	0,00	0,38	0,38
>1633	rlmCD	SAUR2076 (SAR_RS10380)	0,00	0,37	0,00	0,00	0,37	0,00	0,00	0,37	0,00
>1634	dagK	SAUR2077 (SAR_RS10385)	0,00	0,53	0,00	0,00	0,53	0,00	0,00	0,53	0,00
>1635	gatB	SAUR2079 (SAR_RS10395)	0,00	0,56	0,21	0,00	0,56	0,21	0,00	0,56	0,21
>1636	gatA	SAUR2080 (SAR_RS10400)	0,00	0,41	0,34	0,00	0,41	0,34	0,00	0,41	0,34
>1637	gatC	(N/A)	0,00	0,33	0,33	0,00	0,33	0,33	0,00	0,33	0,33
>1638	putP	SAUR2082 (SAR_RS10410)	0,00	0,58	0,26	0,06	0,65	0,32	0,00	0,58	0,26
>1639	camS	SAUR2083 (SAR_RS10415)	0,00	0,99	0,41	0,00	0,99	0,41	0,08	1,08	0,50
>1640	ligA	SAUR2084 (SAR_RS10420)	0,05	>=5,00	0,50	0,00	>=5,00	0,45	0,00	>=5,00	0,45
>1641	pcrA	SAUR2085 (SAR_RS10425)	0,00	0,46	0,41	0,05	0,50	0,46	0,05	0,50	0,46
>1642	pcrB	SAUR2086 (SAR_RS10430)	0,00	0,14	0,14	0,00	0,14	0,14	0,00	0,14	0,14
>1643	yerC	SAUR2087 (SAR_RS10435)	0,00	0,00	0,00	0,00	0,00	0,00	0,00	0,00	0,00
>1644	purB	SAUR2088 (SAR_RS10440)	0,00	0,93	0,15	0,00	0,93	0,15	0,00	0,93	0,15
>1645	sspP=scpA	SAUR2089 (SAR_RS10445)	0,00	0,77	1,80	0,00	0,77	1,80	0,09	0,86	1,89
>1646	sspS	SAUR2090 (SAR_RS10450)	0,00	1,22	1,53	0,00	1,22	1,53	0,00	1,22	1,53
>1647	Q5HEL1	SAUR2091 (SAR_RS10455)	0,00	1,15	1,72	0,00	1,15	1,72	0,00	1,15	1,72
>1648	UPF0316=DUF2179	SAUR2092 (SAR_RS10460)	0,00	0,17	0,00	0,00	0,17	0,00	0,00	0,17	0,00
>1649	nadE	SAUR2093 (SAR_RS10465)	0,00	1,09	0,12	0,00	1,09	0,12	0,00	1,09	0,12
>1650	nadC	SAUR2094 (SAR_RS10470)	0,07	0,82	0,27	0,00	0,75	0,20	0,00	0,75	0,20
>1651	nos	SAUR2095 (SAR_RS10475)	0,00	0,19	0,09	0,00	0,19	0,09	0,00	0,19	0,09
>1652	pdt	SAUR2096 (SAR_RS10480)	0,00	0,88	0,50	0,00	0,88	0,50	0,00	0,88	0,50
>1653	sdcS	SAUR2098 (SAR_RS10490)	0,00	1,41	0,45	0,00	1,41	0,45	0,00	1,41	0,45
>1654	Q5HEK3=pfbA	SAUR2099 (SAR_RS10495)	0,00	1,73	0,36	0,09	1,82	0,46	0,09	1,82	0,46
>1655	pncA	SAUR2100 (SAR_RS10500)	0,00	0,00	0,36	0,00	0,00	0,36	0,00	0,00	0,36
>1656	ppaC	SAUR2101 (SAR_RS10505)	0,00	0,65	0,22	0,00	0,65	0,22	0,11	0,75	0,32
>1657	aldH	SAUR2103 (SAR_RS10515)	0,00	0,80	0,80	0,00	0,80	0,80	0,00	0,80	0,80
>1658	Q5HEJ8	SAUR2104 (SAR_RS10520)	0,00	0,58	0,00	0,00	0,58	0,00	0,00	0,58	0,00
>1659	Q5HEJ7	SAUR2105 (SAR_RS10525)	0,00	0,50	0,00	0,25	0,75	0,25	0,25	0,75	0,25
>1660	A6U329	SAUR2106 (SAR_RS10530)	0,00	0,00	0,00	0,00	0,00	0,00	0,00	0,00	0,00
>1661	A6U330	SAUR2107 (SAR_RS10535)	0,09	0,81	0,09	0,09	0,81	0,09	0,09	0,81	0,09
>1662	Q5HEJ4=DUF4097	SAUR2108 (SAR_RS10540)	0,00	0,95	0,00	0,00	0,95	0,00	0,00	0,95	0,00
>1663	DUF1700	SAUR2109 (SAR_RS10545)	0,00	0,36	0,36	0,00	0,36	0,36	0,00	0,36	0,36
>1664	Q5HEJ2	SAUR2110 (SAR_RS10550)	0,00	0,76	0,95	0,00	0,76	0,95	0,00	0,76	0,95
>1665	Q5HEJ1	SAUR2111 (SAR_RS10555)	0,00	0,35	0,18	0,00	0,35	0,18	0,00	0,35	0,18
>1666	pmtD	SAUR2112 (SAR_RS10560)	0,00	0,67	0,13	0,00	0,67	0,13	0,00	0,67	0,13
>1667	pmtC	SAUR2113 (SAR_RS10565)	0,00	0,34	0,34	0,00	0,34	0,34	0,00	0,34	0,34
>1668	pmtB	SAUR2114 (SAR_RS10570)	0,00	0,73	0,15	0,00	0,73	0,15	0,00	0,73	0,15
>1669	pmtA	SAUR2115 (SAR_RS10575)	0,11	1,00	0,11	0,11	1,00	0,11	0,11	1,00	0,11
>1670	pmtR	SAUR2116 (SAR_RS10580)	0,00	1,84	0,00	0,00	1,84	0,00	0,00	1,84	0,00
>1671	A6U340	SAUR2117 (SAR_RS10585)	0,00	>=5,00	>=5,00	0,00	>=5,00	>=5,00	0,00	>=5,00	>=5,00
>1672	Q5HEI3=aspC	SAUR2119 (SAR_RS10595)	0,00	0,93	0,23	0,00	0,93	0,23	0,08	1,01	0,31
>1673	map=eap	(N/A)	0,17	>=5,00	0,80	0,00	>=5,00	0,63	0,11	>=5,00	0,75
>1674	hlb	SAUR2190 (SAR_RS10950)	0,00	0,91	0,91	0,00	0,91	0,91	0,10	1,01	1,01

Number/ Position in the figures	Gene ID	ID in PubMLST cg scheme	ED133 vs. swan isolate 15V8707	X22 vs. swan isolate 15V8707	CC522 goat isolate 17CS1042 vs. swan isolate 15V8707	ED133 vs. duck isolate V315	X22 vs. duck isolate V315	CC522 goat isolate 17CS1042 vs. duck isolate V315	ED133 vs. duck isolate V482	X22 vs. duck isolate V482	CC522 goat isolate 17CS1042 vs. duck isolate V482
>1675	lukX=lukG	SAUR2191 (SAR_RS10955)	0,10	4,33	0,49	0,00	4,23	0,39	0,00	4,23	0,39
>1676	lukY=lukH	SAUR2192 (SAR_RS10960)	0,00	1,70	0,38	0,00	1,70	0,38	0,09	1,79	0,47
>1677	dapE	SAUR2193 (SAR_RS10965)	0,00	0,65	0,25	0,00	0,65	0,25	0,00	0,65	0,25
>1678	ktrB	SAUR2194 (SAR_RS10970)	0,00	1,07	0,23	0,00	1,07	0,23	0,00	1,07	0,23
>1679	groL=groEL	SAUR2199 (SAR_RS10995)	0,00	3,28	1,48	0,00	3,28	1,48	0,00	3,28	1,48
>1680	groS	SAUR2200 (SAR_RS11000)	0,00	1,40	0,70	0,00	1,40	0,70	0,00	1,40	0,70
>1681	ydiL=mroQ	SAUR2201 (SAR_RS11005)	0,00	>=5,00	1,21	0,00	>=5,00	1,21	0,13	>=5,00	1,34
>1682	sdrH	SAUR2202 (SAR_RS11010)	0,08	>=5,00	4,80	3,12	4,40	>=5,00	3,20	4,48	>=5,00
>1683	Q5HEG8	SAUR3001	0,16	2,71	2,55	0,16	2,71	2,55	0,00	2,55	2,39
>1684	mtnU=ramA	SAUR2204 (SAR_RS11020)	0,00	0,64	0,51	0,00	0,64	0,51	0,13	0,76	0,64
>1685	hld	SAUR3002 (hld)	0,00	>=5,00	0,74	0,00	>=5,00	0,74	0,00	>=5,00	0,74
>1686	agrB-I	SAUR2953 (agrB)	0,00	2,63	0,00	0,00	2,63	0,00	0,00	2,63	0,00
>1687	agrD-I	SAUR2956 (agrD3)	0,00	0,00	0,00	0,00	0,00	0,00	0,00	0,00	0,00
>1688	agrC-I	SAUR2958 (agrC)	0,00	>=5,00	0,08	0,00	>=5,00	0,08	0,00	>=5,00	0,08
>1689	agrA	SAUR2959 (agrA)	0,00	0,84	0,00	0,00	0,84	0,00	0,00	0,84	0,00
>1690	scrK=frk	SAUR2210 (SAR_RS11050)	0,00	1,15	0,42	0,00	1,15	0,42	0,10	1,25	0,52
>1691	scrB	SAUR2211 (SAR_RS11055)	0,00	1,28	0,67	0,00	1,28	0,67	0,07	1,35	0,74
>1692	scrR	SAUR2212 (SAR_RS11060)	0,11	1,47	0,53	0,00	1,37	0,42	0,00	1,37	0,42
>1693	nrgA	SAUR2213 (SAR_RS11065)	0,08	3,60	0,96	0,00	3,52	0,88	0,16	3,68	1,04
>1694	Q5HEF7=tusA	SAUR2214 (SAR_RS11070)	0,00	>=5,00	0,40	0,00	>=5,00	0,40	0,00	>=5,00	0,40
>1695	yeeE=DUF395	SAUR2215 (SAR_RS11075)	0,00	1,40	0,47	0,00	1,40	0,47	0,00	1,40	0,47
>1696	rex	SAUR2216 (SAR_RS11080)	0,00	0,16	0,47	0,00	0,16	0,47	0,00	0,16	0,47
>1697	yheS	SAUR2217 (SAR_RS11085)	0,00	0,78	0,26	0,00	0,78	0,26	0,05	0,83	0,31
>1698	mutS3	SAUR3003	0,00	1,74	0,87	0,06	1,80	0,93	0,06	1,80	0,93
>1699	tsaD=gcp	SAUR2220 (SAR_RS11100)	0,10	>=5,00	0,68	0,10	>=5,00	0,68	0,10	>=5,00	0,68
>1700	rimI	SAUR2221 (SAR_RS11105)	0,00	0,86	0,43	0,00	0,86	0,43	0,00	0,86	0,43
>1701	tsaB	SAUR2222 (SAR_RS11110)	0,00	1,06	0,15	0,00	1,06	0,15	0,30	1,06	0,45
>1702	tsaE=yjeE	(N/A)	0,00	1,15	0,00	0,23	1,38	0,23	0,23	1,38	0,23
>1703	ilvD	SAUR2225 (SAR_RS11125)	0,06	1,12	0,30	0,12	1,18	0,36	0,12	1,18	0,36
>1704	ilvB	SAUR2226 (SAR_RS11130)	0,06	0,73	0,73	0,06	0,73	0,73	0,06	0,73	0,73
>1705	ilvH	SAUR2227 (SAR_RS11135)	0,39	2,35	0,39	0,39	2,35	0,39	0,39	2,35	0,39
>1706	ilvC	SAUR2228 (SAR_RS11140)	0,00	0,50	0,20	0,10	0,50	0,20	0,00	0,50	0,20
>1707	leuA1	SAUR2229 (SAR_RS11145)	0,00	0,46	>=5,00	0,00	0,46	>=5,00	0,00	0,46	>=5,00
>1708	leuB	SAUR2230 (SAR_RS11150)	0,10	1,05	0,19	0,10	1,05	0,19	0,10	1,05	0,19
>1709	leuC	SAUR2231 (SAR_RS11155)	0,00	1,24	0,22	0,00	1,24	0,22	0,00	1,24	0,22
>1710	leuD	SAUR2232 (SAR_RS11160)	0,00	>=5,00	0,70	0,00	>=5,00	0,70	0,00	>=5,00	0,70
>1711	ilvA2	SAUR2233 (SAR_RS11165)	0,00	2,99	0,47	0,00	2,99	0,47	0,00	2,99	0,47
>1712	sprL	SAUR2240 (SAR_RS11200)	0,00	1,54	1,10	0,00	1,54	1,10	0,00	1,54	1,10
>1713	yhgF	SAUR2241 (SAR_RS11205)	0,00	1,44	0,46	0,00	1,44	0,46	0,00	1,44	0,46
>1714	sigB	SAUR2242 (SAR_RS11210)	0,00	1,56	0,13	0,00	1,56	0,13	0,00	1,56	0,13
>1715	rsbW	SAUR2243 (SAR_RS11215)	0,00	0,63	0,21	0,00	0,63	0,21	0,21	0,63	0,42
>1716	rsbV	SAUR2244 (SAR_RS11220)	0,00	0,31	0,31	0,00	0,31	0,31	0,00	0,31	0,31
>1717	rsbU	SAUR2245 (SAR_RS11225)	0,00	0,10	0,00	0,00	0,10	0,00	0,00	0,10	0,00
>1718	mazF	SAUR2247 (SAR_RS11235)	0,00	0,28	0,28	0,00	0,28	0,28	0,00	0,28	0,28
>1719	mazE=ydcD	SAUR2248 (SAR_RS11240)	0,00	0,00	0,00	0,00	0,00	0,00	0,00	0,00	0,00
>1720	alr1	SAUR2249 (SAR_RS11245)	0,00	1,48	0,87	0,00	1,48	0,87	0,00	1,48	0,87
>1721	acpS	SAUR2250 (SAR_RS11250)	0,00	1,39	0,56	0,00	1,39	0,56	0,00	1,39	0,56
>1722	A5IUL8	SAUR2251 (SAR_RS11255)	0,00	2,24	0,81	0,00	2,24	0,81	0,00	2,24	0,81
>1723	DUF0304	SAUR2252 (SAR_RS11260)	0,00	2,78	0,57	0,00	2,78	0,57	0,00	2,78	0,57
>1724	Q9ZAH9	SAUR2253 (SAR_RS11265)	0,00	2,71	0,63	0,00	2,71	0,63	0,00	2,71	0,63
>1725	kdpC-chr	SAUR2254 (SAR_RS11270)	0,00	1,07	1,25	0,00	1,07	1,25	0,00	1,07	1,25
>1726	kdpB-chr	SAUR2255 (SAR_RS11275)	0,00	1,48	0,49	0,00	1,48	0,49	0,05	1,52	0,54
>1727	kdpA-chr	SAUR2256 (SAR_RS11280)	0,00	1,60	0,59	1,78	3,26	2,37	1,78	3,26	2,37
>1728	kdpF-chr	SAUR2257 (SAR_RS11285)	0,00	2,70	2,70	0,00	2,70	2,70	0,00	2,70	2,70
>1729	kdpD-chr	SAUR2258 (SAR_RS11290)	0,00	1,13	0,30	0,00	1,13	0,30	0,08	1,20	0,38
>1730	kdpE-chr	SAUR2259 (SAR_RS11295)	0,14	1,44	1,01	0,14	1,44	1,01	0,14	1,44	1,01
>1731	cshA	SAUR2260 (SAR_RS11300)	0,00	0,99	0,33	0,00	0,99	0,33	0,00	0,99	0,33
>1732	murF	SAUR2261 (SAR_RS11305)	0,00	0,88	0,00	0,00	0,88	0,00	0,00	0,88	0,00
>1733	ddlA	SAUR2262 (SAR_RS11310)	0,00	0,37	0,75	0,09	0,47	0,84	0,09	0,47	0,84
>1734	ftsW1=rodA	SAUR2263 (SAR_RS11315)	0,00	0,91	0,58	0,00	0,91	0,58	0,00	0,91	0,58
>1735	Q5HEB5	(N/A)	0,00	0,00	0,72	0,00	0,00	0,72	0,00	0,00	0,72
>1736	csoZ	SAUR2266 (SAR_RS11330)	0,00	0,95	1,43	0,00	0,95	1,43	0,00	0,95	1,43
>1737	csoR	SAUR2267 (SAR_RS11335)	0,00	0,68	1,02	0,34	1,02	1,36	0,00	0,68	1,02
>1738	cls2	SAUR2268 (SAR_RS11340)	0,13	0,61	0,20	0,07	0,54	0,13	0,07	0,54	0,13
>1739	yedJ	SAUR2269 (SAR_RS11345)	0,15	1,70	1,08	0,15	1,70	1,08	0,15	1,70	1,08
>1740	yidC=oxaA	SAUR2271 (SAR_RS11355)	0,11	0,34	0,23	0,11	0,34	0,23	0,11	0,34	0,23
>1741	thiE	SAUR2272 (SAR_RS11360)	0,00	>=5,00	0,31	0,00	>=5,00	0,31	0,00	>=5,00	0,31
>1742	thiM	SAUR2273 (SAR_RS11365)	0,13	1,01	0,13	0,00	0,88	0,00	0,00	0,88	0,00
>1743	thiD2	SAUR2274 (SAR_RS11370)	0,00	1,32	0,24	0,00	1,32	0,24	0,12	1,44	0,36
>1744	tenA	SAUR2275 (SAR_RS11375)	0,14	0,58	0,14	0,00	0,43	0,00	0,00	0,43	0,00
>1745	sceD	SAUR2277 (SAR_RS11385)	0,00	0,71	1,28	0,00	0,71	1,28	0,00	0,71	1,28
>1746	Q5HEA3	SAUR2278 (SAR_RS11390)	0,00	0,00	0,00	0,00	0,00	0,00	0,00	0,00	0,00
>1747	ywpF	SAUR2279 (SAR_RS11395)	0,00	0,23	0,00	0,00	0,23	0,00	0,00	0,23	0,00
>1748	fabZ	SAUR2280 (SAR_RS11400)	0,23	1,13	0,45	0,23	1,13	0,45	0,23	1,13	0,45
>1749	murA	SAUR2281 (SAR_RS11405)	0,00	4,34	0,32	0,00	4,34	0,32	0,00	4,34	0,32
>1750	ywzB	SAUR2282 (SAR_RS11410)	0,00	0,00	0,00	0,00	0,00	0,00	0,00	0,00	0,00
>1751	atpE	SAUR2284 (SAR_RS11420)	0,00	0,25	0,25	0,25	0,49	0,49	0,25	0,49	0,49
>1752	atpD	SAUR2285 (SAR_RS11425)	0,00	0,42	0,42	0,00	0,42	0,42	0,00	0,42	0,42
>1753	atpG	SAUR2286 (SAR_RS11430)	0,00	0,23	0,00	0,12	0,35	0,12	0,00	0,23	0,00
>1754	atpA	SAUR2287 (SAR_RS11435)	0,00	0,07	0,07	0,00	0,07	0,07	0,00	0,07	0,07
>1755	atpH	SAUR2288 (SAR_RS11440)	0,00	0,56	0,19	0,00	0,56	0,19	0,00	0,56	0,19
>1756	atpF	SAUR2289 (SAR_RS11445)	0,00	0,19	0,00	0,38	0,57	0,38	0,19	0,38	0,19
>1757	atpL=atpE	(N/A)	0,00	0,00	0,00	0,00	0,00	0,00	0,00	0,00	0,00
>1758	atpB	SAUR2291 (SAR_RS11455)	0,00	0,27	0,00	0,00	0,27	0,00	0,00	0,27	0,00
>1759	atpI	SAUR2292 (SAR_RS11460)	0,00	1,13	3,67	0,28	1,41	3,95	0,28	1,41	3,95
>1760	mnaA=wecB	SAUR2293 (SAR_RS11465)	0,00	0,53	0,53	0,00	0,53	0,53	0,00	0,53	0,53
>1761	upp	SAUR2294 (SAR_RS11470)	0,00	0,63	0,16	0,00	0,63	0,16	0,00	0,63	0,16
>1762	glyA	SAUR2295 (SAR_RS11475)	0,08	0,32	0,48	0,16	0,40	0,56	0,08	0,32	0,48
>1763	ywlG	SAUR2296 (SAR_RS11480)	0,19	0,95	0,95	0,00	0,76	0,76	0,00	0,76	0,76
>1764	ptpB	SAUR2297 (SAR_RS11485)	0,00	0,24	1,19	0,00	0,24	1,19	0,00	0,24	1,19
>1765	tsaC=rimN	SAUR2298 (SAR_RS11490)	0,00	0,85	1,33	0,00	0,85	1,33	0,00	0,85	1,33
>1766	prmC=hemK	SAUR2299 (SAR_RS11495)	0,00	0,36	0,48	0,00	0,36	0,48	0,00	0,36	0,48
>1767	prfA	SAUR2300 (SAR_RS11500)	0,00	1,02	0,46	0,00	1,02	0,46	0,00	1,02	0,46
>1768	tdk	SAUR2301 (SAR_RS11505)	0,00	0,83	0,67	0,00	0,83	0,67	0,00	0,83	0,67
>1769	rpmE	(N/A)	0,00	0,00	0,00	0,00	0,00	0,00	0,00	0,00	0,00
>1770	rho	SAUR2304 (SAR_RS11520)	0,00	0,76	0,38	0,00	0,76	0,38	0,00	0,76	0,38
>1771	aldA3	SAUR2305 (SAR_RS11525)	>=5,00	>=5,00	0,00	>=5,00	>=5,00	0,00	>=5,00	>=5,00	0,00
>1772	arxR	SAUR2306 (SAR_RS11530)	0,00	0,89	1,19	0,00	0,89	1,19	0,00	0,89	1,19
>1773	murZ	SAUR2307 (SAR_RS11535)	0,00	0,79	0,48	0,00	0,79	0,48	0,00	0,79	0,48
>1774	fbaA	SAUR2308 (SAR_RS11540)	0,00	0,35	0,35	0,00	0,35	0,35	0,00	0,35	0,35
>1775	DUF2529	SAUR2309 (SAR_RS11545)	0,00	0,77	0,96	0,00	0,77	0,96	0,00	0,77	0,96
>1776	pyrG=ctrA	SAUR2310 (SAR_RS11550)	0,00	1,43	0,25	0,00	1,43	0,25	0,06	1,49	0,31
>1777	rpoE	SAUR2311 (SAR_RS11555)	0,19	>=5,00	0,00	0,19	>=5,00	0,00	0,19	>=5,00	0,00
>1778	Q5HE71	SAUR2312 (SAR_RS11560)	0,00	1,05	0,58	0,00	1,05	0,58	0,00	1,05	0,58
>1779	coaW	SAUR2313 (SAR_RS11565)	0,00	0,87	0,50	0,00	0,87	0,50	0,00	0,87	0,50
>1780	DUF2750	SAUR2314 (SAR_RS11570)	0,00	0,30	>=5,00	0,00	0,30	>=5,00	0,00	0,30	>=5,00
>1781	A5IUS7	SAUR2315 (SAR_RS11575)	0,00	1,34	0,67	0,00	1,34	0,67	0,00	1,34	0,67
>1782	hmrA	SAUR2316 (SAR_RS11580)	0,08	0,51	>=5,00	0,08	0,51	>=5,00	0,25	0,67	>=5,00
>1783	luxS	SAUR2317 (SAR_RS11585)	0,00	1,06	0,21	0,00	1,06	0,21	0,00	1,06	0,21
>1784	Q5HE65	SAUR2318 (SAR_RS11590)	0,00	0,85	0,85	0,00	0,85	0,85	0,00	0,85	0,85
>1785	pdp	SAUR2319 (SAR_RS11595)	0,00	1,31	0,54	0,00	1,31	0,54	0,00	1,31	0,54
>1786	deoC-L2	SAUR1837 (SAR_RS09185)	0,00	1,66	1,81	0,00	1,66	1,81	0,00	1,66	1,81
>1787	deoD-L2	SAUR2322 (SAR_RS11610)	0,00	0,42	0,42	0,00	0,42	0,42	0,00	0,42	0,42
>1788	dpsA	SAUR2323 (SAR_RS11615)	0,00	1,80	0,00	0,00	1,80	0,00	0,00	1,80	0,00
>1789	DUF0393	SAUR2324 (SAR_RS11620)	0,00	0,48	0,24	0,00	0,48	0,24	0,00	0,48	0,24
>1790	Q5HE59	SAUR2325 (SAR_RS11625)	0,00	0,58	0,66	0,07	0,66	0,73	0,07	0,66	0,73
>1791	Q7A0B3	SAUR2326 (SAR_RS11630)	0,00	0,43	0,00	>=5,00	>=5,00	>=5,00	0,00	0,43	0,00
>1792	manA1=gmuF	SAUR2327 (SAR_RS11635)	0,00	0,53	0,21	0,21	0,75	0,43	>=5,00	>=5,00	>=5,00
>1793	yhfK1	SAUR2328 (SAR_RS11640)	0,00	1,05	0,90	0,15	1,05	1,05	0,00	1,05	0,90
>1794	czrA=zntR	SAUR2329 (SAR_RS11645)	0,00	0,62	0,00	0,00	0,62	0,00	0,00	0,62	0,00
>1795	czrB=zntA	SAUR2330 (SAR_RS11650)	0,00	1,83	0,41	0,00	1,83	0,41	0,00	1,83	0,41
>1796	Q9ZB00	SAUR2331 (SAR_RS11655)	0,00	1,28	0,00	0,00	1,28	0,00	0,00	1,28	0,00
>1797	ylmA	SAUR2335 (SAR_RS11675)	0,00	>=5,00	>=5,00	0,00	>=5,00	>=5,00	0,00	>=5,00	>=5,00
>1798	glmS	SAUR2336 (SAR_RS11680)	0,00	2,58	0,71	0,00	2,58	0,71	0,00	2,58	0,71
>1799	mtlA	SAUR2337 (SAR_RS11685)	0,00	>=5,00	>=5,00	0,06	>=5,00	>=5,00	0,00	>=5,00	>=5,00
>1800	mtlR	SAUR2338 (SAR_RS11690)	0,00	1,08	0,66	0,00	1,08	0,66	0,05	1,13	0,70
>1801	mtlF	SAUR2339 (SAR_RS11695)	0,00	0,69	0,23	0,23	0,92	0,46	0,46	1,15	0,69
>1802	mtlD	SAUR2340 (SAR_RS11700)	0,00	0,90	0,45	0,00	0,90	0,45	0,00	0,90	0,45
>1803	sasB=fmtB	(N/A)	>=5,00	2,70	>=5,00	>=5,00	2,70	>=5,00	>=5,00	2,78	>=5,00
>1804	glmM	SAUR2343 (SAR_RS11715)	0,07	0,37	0,37	0,15	0,44	0,44	0,15	0,44	0,44
>1805	ybbR=cdaR	SAUR2344 (SAR_RS11720)	0,11	0,54	0,21	0,11	0,54	0,21	0,11	0,54	0,21
>1806	cdaA=dacA	SAUR2345 (SAR_RS11725)	0,12	0,49	0,25	0,12	0,49	0,25	0,00	0,37	0,12
>1807	argI=rocF	SAUR2346 (SAR_RS11730)	0,00	0,44	0,44	0,00	0,44	0,44	0,22	0,66	0,66
>1808	salA	SAUR2357 (SAR_RS11785)	0,09	2,91	0,94	0,00	2,82	0,85	>=5,00	>=5,00	>=5,00
>1809	yncB=lmrS	SAUR2358 (SAR_RS11790)	0,00	3,33	2,36	0,07	3,40	2,43	0,00	3,33	2,36
>1810	sepA	SAUR2359 (SAR_RS11795)	0,00	0,64	0,00	0,00	0,64	0,00	0,00	0,64	0,00
>1811	sdrM	(N/A)	0,00	1,71	0,67	0,00	1,71	0,67	0,00	1,71	0,67
>1812	hlIII=yqfA	SAUR2361 (SAR_RS11805)	0,00	>=5,00	1,02	0,00	>=5,00	1,02	0,00	>=5,00	1,02
>1813	Q5HE34=urtF	SAUR2362 (SAR_RS11810)	0,00	1,43	0,51	0,08	1,52	0,59	0,08	1,52	0,59
>1814	yvsG=ydjM	SAUR2363 (SAR_RS11815)	0,19	1,74	0,58	0,00	1,55	0,39	0,00	1,55	0,39
>1815	ynzG	SAUR2364 (SAR_RS11820)	0,00	1,53	0,38	0,00	1,53	0,38	0,00	1,53	0,38
>1816	Q5HE31	SAUR2365 (SAR_RS11825)	0,00	1,39	0,22	0,00	1,39	0,22	0,15	1,53	0,36
>1817	htsC=fecD	SAUR2366 (SAR_RS11830)	0,00	0,93	0,83	0,00	0,93	0,83	0,10	1,04	0,93
>1818	htsB	SAUR2367 (SAR_RS11835)	0,19	0,87	0,58	0,10	0,78	0,48	0,10	0,78	0,48
>1819	htsA	SAUR2368 (SAR_RS11840)	0,00	2,44	0,00	0,00	2,44	0,00	0,00	2,44	0,00
>1820	Q5HE27=sfnaC	SAUR2370 (SAR_RS11850)	0,00	1,12	1,21	0,09	1,21	1,31	0,00	1,12	1,21
>1821	rhbC1=sfaB	SAUR2371 (SAR_RS11855)	0,00	1,88	1,37	0,00	1,88	1,37	0,06	1,93	1,42
>1822	Q5HE25=sfaA	SAUR2372 (SAR_RS11860)	0,00	0,84	0,59	0,00	0,84	0,59	>=5,00	>=5,00	>=5,00

Number/ Position in the figures	Gene ID	ID in PubMLST cg scheme	ED133 vs. swan isolate 15V8707	X22 vs. swan isolate 15V8707	CC522 goat isolate 17CS1042 vs. swan isolate 15V8707	ED133 vs. duck isolate V315	X22 vs. duck isolate V315	CC522 goat isolate 17CS1042 vs. duck isolate V315	ED133 vs. duck isolate V482	X22 vs. duck isolate V482	CC522 goat isolate 17CS1042 vs. duck isolate V482
>1823	rhbC2=sfaD	SAUR2373 (SAR_RS11865)	0,05	0,91	0,35	0,05	0,91	0,35	0,05	0,91	0,35
>1824	asp23	SAUR2374 (SAR_RS11870)	0,00	2,16	0,20	0,00	2,16	0,20	0,00	2,16	0,20
>1825	DUF2273	SAUR2375 (SAR_RS11875)	0,00	1,67	0,42	0,00	1,67	0,42	0,00	1,67	0,42
>1826	Q5HE21=amaP	SAUR2376 (SAR_RS11880)	0,00	0,36	1,46	0,00	0,36	1,46	>=5,00	>=5,00	>=5,00
>1827	opuD2	SAUR2377 (SAR_RS11885)	0,13	1,60	0,96	0,13	1,60	0,96	0,13	1,60	0,96
>1828	Q5HE19	SAUR2378 (SAR_RS11890)	0,00	1,49	0,79	0,00	1,49	0,79	0,00	1,49	0,79
>1829	qorA=curA	SAUR2379 (SAR_RS11895)	0,10	1,59	1,29	0,30	1,79	1,49	0,10	1,59	1,29
>1830	DUF0915	(N/A)	0,00	0,80	1,49	0,00	0,80	1,49	0,00	0,80	1,49
>1831	lacG	SAUR2381 (SAR_RS11905)	0,00	1,34	0,85	0,00	1,34	0,85	0,00	1,34	0,85
>1832	lacE	SAUR2382 (SAR_RS11910)	0,58	1,28	1,05	0,58	1,28	1,05	0,58	1,28	1,05
>1833	lacF	SAUR2383 (SAR_RS11915)	0,00	1,28	1,28	0,00	1,28	1,28	0,00	1,28	1,28
>1834	lacD	SAUR2384 (SAR_RS11920)	0,00	0,82	0,41	0,00	0,82	0,41	0,00	0,82	0,41
>1835	lacC	SAUR2385 (SAR_RS11925)	0,00	2,47	0,64	0,00	2,47	0,64	0,00	2,47	0,64
>1836	lacB	SAUR2386 (SAR_RS11930)	0,00	0,78	0,00	0,00	0,78	0,00	0,00	0,78	0,00
>1837	lacA	SAUR2387 (SAR_RS11935)	0,23	0,47	0,47	0,00	0,23	0,23	0,00	0,23	0,23
>1838	lacR	SAUR2388 (SAR_RS11940)	0,26	2,65	0,00	0,26	2,65	0,00	0,26	2,65	0,00
>1839	cobB	SAUR2389 (SAR_RS11945)	0,00	>=5,00	>=5,00	0,00	>=5,00	>=5,00	>=5,00	2,46	1,50
>1840	Q5HE05	(N/A)	0,00	>=5,00	>=5,00	0,00	>=5,00	>=5,00	0,00	>=5,00	>=5,00
>1841	yvgN2	SAUR2394 (SAR_RS11970)	0,00	1,06	1,06	0,00	1,06	1,06	0,00	1,06	1,06
>1842	adhR	SAUR2395 (SAR_RS11975)	0,00	0,96	0,96	0,00	0,96	0,96	0,00	0,96	0,96
>1843	hysA-L1.8	(N/A)	0,04	>=5,00	>=5,00	0,04	>=5,00	>=5,00	0,08	>=5,00	>=5,00
>1844	eap-L2=eapH-1	SAUR2400 (SAR_RS12000)	0,00	3,29	0,47	0,00	3,29	0,47	0,00	3,29	0,47
>1845	alsD-L1=budA	SAUR2401 (SAR_RS12005)	0,14	2,55	1,13	0,14	2,55	1,13	0,14	2,55	1,13
>1846	alsS	SAUR2402 (SAR_RS12010)	0,00	3,24	0,54	0,00	3,24	0,54	0,00	3,24	0,54
>1847	rpsI	SAUR2406 (SAR_RS12030)	0,00	0,25	0,00	0,00	0,25	0,00	0,00	0,25	0,00
>1848	rplM	SAUR2407 (SAR_RS12035)	0,00	0,46	0,46	0,00	0,46	0,46	0,00	0,46	0,46
>1849	truA	SAUR2408 (SAR_RS12040)	0,00	2,99	2,24	0,00	2,99	2,24	0,00	2,99	2,24
>1850	ecfT	SAUR2409 (SAR_RS12045)	0,00	1,49	0,99	0,00	1,49	0,99	0,00	1,49	0,99
>1851	ecfA1	SAUR2410 (SAR_RS12050)	0,00	>=5,00	0,93	0,00	>=5,00	0,93	>=5,00	0,00	>=5,00
>1852	ecfA2	SAUR2411 (SAR_RS12055)	0,12	1,11	0,49	0,12	1,11	0,49	0,12	1,11	0,49
>1853	rplQ	(N/A)	0,00	0,00	0,00	0,00	0,00	0,00	0,00	0,00	0,00
>1854	rpoA	SAUR2415 (SAR_RS12075)	0,00	0,11	0,00	0,00	0,11	0,00	0,00	0,11	0,00
>1855	rpsK	SAUR2416 (SAR_RS12080)	0,00	0,00	0,00	0,00	0,00	0,00	0,00	0,00	0,00
>1856	rpsM	(N/A)	0,00	0,27	0,55	0,00	0,27	0,55	0,00	0,27	0,55
>1857	rpmJ	(N/A)	0,00	0,00	0,00	0,00	0,00	0,00	0,00	0,00	0,00
>1858	infA	(N/A)	0,00	0,00	0,00	0,00	0,00	0,00	0,00	0,00	0,00
>1859	adk	SAUR2420 (SAR_RS12100)	0,00	0,15	0,15	0,00	0,15	0,15	0,00	0,15	0,15
>1860	secY1	SAUR2421 (SAR_RS12105)	0,00	0,15	0,08	0,00	0,15	0,08	0,08	0,23	0,15
>1861	rplO	SAUR2422 (SAR_RS12110)	0,00	0,00	0,00	0,00	0,00	0,00	0,00	0,00	0,00
>1862	rpmD	(N/A)	0,00	0,00	0,00	0,00	0,00	0,00	0,00	0,00	0,00
>1863	rpsE	(N/A)	0,00	0,00	0,00	0,00	0,00	0,00	0,00	0,00	0,00
>1864	rplR	SAUR2425 (SAR_RS12125)	0,00	0,28	0,00	0,28	0,56	0,28	0,00	0,28	0,00
>1865	rplF	SAUR2426 (SAR_RS12130)	0,00	0,19	0,19	0,00	0,19	0,19	0,00	0,19	0,19
>1866	rpsH	SAUR2427 (SAR_RS12135)	0,00	0,25	0,25	0,00	0,25	0,25	0,25	0,50	0,50
>1867	rpsZ	(N/A)	0,00	0,00	0,00	0,00	0,00	0,00	0,00	0,00	0,00
>1868	rplE	(N/A)	0,00	0,19	0,37	0,00	0,19	0,37	0,00	0,19	0,37
>1869	rplX	SAUR2430 (SAR_RS12150)	0,00	0,31	0,00	0,00	0,31	0,00	0,00	0,31	0,00
>1870	rplN	SAUR2431 (SAR_RS12155)	0,00	0,54	0,54	0,00	0,54	0,54	0,00	0,54	0,54
>1871	rpsQ	SAUR2432 (SAR_RS12160)	0,00	0,00	0,38	0,00	0,00	0,38	0,00	0,00	0,38
>1872	rpmC	(N/A)	0,00	0,00	0,00	0,00	0,00	0,00	0,00	0,00	0,00
>1873	rplP	SAUR2434 (SAR_RS12170)	0,00	0,23	0,00	0,00	0,23	0,00	0,00	0,23	0,00
>1874	rpsC	SAUR2435 (SAR_RS12175)	0,15	0,31	0,46	0,00	0,15	0,31	0,00	0,15	0,31
>1875	rplV	(N/A)	2,82	0,00	0,00	2,82	0,00	0,00	2,82	0,00	0,00
>1876	rpsS	(N/A)	0,00	0,00	0,00	0,00	0,00	0,00	0,00	0,00	0,00
>1877	rplB	SAUR2438 (SAR_RS12190)	0,00	0,24	0,24	0,12	0,36	0,36	0,00	0,24	0,24
>1878	rplW	SAUR2439 (SAR_RS12195)	0,00	0,72	0,72	0,00	0,72	0,72	0,00	0,72	0,72
>1879	rplD	SAUR2440 (SAR_RS12200)	0,00	0,16	0,00	0,00	0,16	0,00	0,00	0,16	0,00
>1880	rplC	SAUR2441 (SAR_RS12205)	0,00	0,00	0,30	0,00	0,00	0,30	0,00	0,00	0,30
>1881	rpsJ	SAUR2442 (SAR_RS12210)	0,00	0,65	0,00	0,00	0,65	0,00	0,00	0,65	0,00
>1882	Q5HDV6	SAUR2443 (SAR_RS12215)	0,00	1,03	0,51	0,00	1,03	0,51	0,00	1,03	0,51
>1883	pbuG	SAUR2444 (SAR_RS12220)	0,00	0,67	0,45	0,00	0,67	0,45	0,07	0,75	0,52
>1884	topB	SAUR2445 (SAR_RS12225)	0,05	0,89	0,70	0,14	0,98	0,80	0,09	0,94	0,75
>1885	Q5HDV3	SAUR2447 (SAR_RS12235)	0,00	1,46	2,03	0,00	1,46	2,03	0,00	1,46	2,03
>1886	glcU	SAUR2448 (SAR_RS12240)	0,00	0,58	0,23	0,12	0,69	0,35	0,00	0,58	0,23
>1887	Q1Y7Y7	SAUR2449 (SAR_RS12245)	0,00	1,21	>=5,00	0,00	1,21	>=5,00	0,00	1,21	>=5,00
>1888	Q5HDU8=mspA	SAUR2451 (SAR_RS12255)	0,00	0,63	0,63	0,00	0,63	0,63	0,00	0,63	0,63
>1889	acrB	SAUR2452 (SAR_RS12260)	0,03	>=5,00	0,66	0,00	>=5,00	0,63	0,00	>=5,00	0,63
>1890	fmhB=femX	SAUR2453 (SAR_RS12265)	0,00	0,87	0,39	0,08	0,95	0,47	0,00	0,87	0,39
>1891	Q5HDU5	SAUR2455 (SAR_RS12275)	0,00	0,92	0,78	0,00	0,92	0,78	0,00	0,92	0,78
>1892	Q5HDU4	SAUR2456 (SAR_RS12280)	0,00	0,91	0,68	0,00	0,91	0,68	0,00	0,91	0,68
>1893	ybfD	SAUR2457 (SAR_RS12285)	0,00	0,33	0,41	0,00	0,33	0,41	0,00	0,33	0,41
>1894	sarV	SAUR2458 (SAR_RS12290)	0,00	0,00	0,00	0,00	0,00	0,00	0,00	0,00	0,00
>1895	moaA	SAUR2461 (SAR_RS12305)	0,00	0,78	0,29	0,00	0,78	0,29	0,00	0,78	0,29
>1896	mobA	SAUR2462 (SAR_RS12310)	0,00	0,67	1,00	0,00	0,67	1,00	0,00	0,67	1,00
>1897	moaD	SAUR2463 (SAR_RS12315)	0,00	1,71	>=5,00	0,00	1,71	>=5,00	0,00	1,71	>=5,00
>1898	moaE	SAUR2464 (SAR_RS12320)	0,00	0,89	0,45	0,00	0,89	0,45	0,00	0,89	0,45
>1899	mobB	SAUR2465 (SAR_RS12325)	0,00	>=5,00	0,82	0,00	>=5,00	0,82	0,00	>=5,00	0,82
>1900	moeA	SAUR2466 (SAR_RS12330)	0,00	1,43	0,40	0,00	1,43	0,40	0,00	1,43	0,40
>1901	moaC	SAUR2467 (SAR_RS12335)	0,00	0,40	1,21	0,20	0,61	1,41	0,00	0,40	1,21
>1902	moaB	SAUR2468 (SAR_RS12340)	0,00	1,18	0,59	0,00	1,18	0,59	0,00	1,18	0,59
>1903	moeB	SAUR2469 (SAR_RS12345)	>=5,00	1,19	0,40	0,00	>=5,00	>=5,00	0,00	>=5,00	>=5,00
>1904	modC	SAUR2470 (SAR_RS12350)	0,00	0,83	0,50	0,00	0,83	0,50	0,00	0,83	0,50
>1905	modB	SAUR2471 (SAR_RS12355)	0,15	0,15	0,60	0,15	0,15	0,60	0,15	0,15	0,60
>1906	modA	SAUR2472 (SAR_RS12360)	0,00	2,04	0,26	0,00	2,04	0,26	0,00	2,04	0,26
>1907	fdhD=narQ	SAUR2473 (SAR_RS12365)	0,00	1,50	0,88	0,13	1,63	1,00	0,00	1,50	0,88
>1908	Q5HDS6	SAUR2474 (SAR_RS12370)	0,00	0,78	0,13	0,00	0,78	0,13	0,00	0,78	0,13
>1909	bioY	SAUR2475 (SAR_RS12375)	0,00	0,72	0,54	0,00	0,72	0,54	0,00	0,72	0,54
>1910	rihB	SAUR2476 (SAR_RS12380)	>=5,00	1,70	1,17	0,00	>=5,00	>=5,00	0,00	>=5,00	>=5,00
>1911	fhuD2	SAUR2477 (SAR_RS12385)	0,00	0,55	0,44	0,00	0,55	0,44	0,00	0,55	0,44
>1912	caiA	SAUR2478 (SAR_RS12390)	0,00	0,95	0,52	0,00	0,95	0,52	0,00	0,95	0,52
>1913	utp-yut	SAUR2479 (SAR_RS12395)	0,00	4,19	0,77	0,00	4,19	0,77	0,00	4,19	0,77
>1914	ureA	(N/A)	0,33	0,66	0,66	0,33	0,66	0,66	0,33	0,66	0,66
>1915	ureB	SAUR2481 (SAR_RS12405)	0,00	0,73	0,24	0,00	0,73	0,24	0,00	0,73	0,24
>1916	ureC	SAUR2482 (SAR_RS12410)	0,00	1,22	0,35	0,00	1,22	0,35	0,06	1,28	0,41
>1917	ureE	SAUR2483 (SAR_RS12415)	0,00	1,32	1,10	0,00	1,32	1,10	0,00	1,32	1,10
>1918	ureF	SAUR2484 (SAR_RS12420)	0,00	1,01	1,01	0,00	1,01	1,01	0,00	1,01	1,01
>1919	ureG	SAUR2485 (SAR_RS12425)	0,00	0,98	0,65	0,00	0,98	0,65	0,16	1,14	0,81
>1920	ureD	SAUR2486 (SAR_RS12430)	0,00	0,60	0,48	0,00	0,60	0,48	0,00	0,60	0,48
>1921	sarR	(N/A)	0,00	0,00	0,00	0,00	0,00	0,00	0,00	0,00	0,00
>1922	rsr	SAUR2489 (SAR_RS12445)	0,00	0,00	0,00	0,00	0,00	0,00	0,00	0,00	0,00
>1923	iraD	SAUR2490 (SAR_RS12450)	0,00	0,94	0,00	0,00	0,94	0,00	0,00	0,94	0,00
>1924	iraC	SAUR2491 (SAR_RS12455)	>=5,00	>=5,00	>=5,00	0,00	1,48	0,20	0,61	1,99	0,82
>1925	ssaA2	SAUR2492 (SAR_RS12460)	0,00	1,24	0,12	0,00	1,24	0,12	0,00	1,24	0,12
>1926	nhaC	SAUR2494 (SAR_RS12470)	0,00	0,72	0,36	0,00	0,72	0,36	0,00	0,72	0,36
>1927	Q5HDQ7=odh	SAUR2495 (SAR_RS12475)	0,00	0,65	0,09	0,00	0,65	0,09	0,00	0,65	0,09
>1928	Q2FEJ1=DUF4870	SAUR2496 (SAR_RS12480)	2,31	4,63	4,17	0,00	2,31	1,85	0,00	2,31	1,85
>1929	ssaA3	SAUR2497 (SAR_RS12485)	0,00	2,79	0,20	0,00	2,79	0,20	0,20	2,99	0,40
>1930	yvcT	SAUR2499 (SAR_RS12495)	0,10	1,15	0,42	0,10	1,15	0,42	0,21	1,26	0,52
>1931	Q8NV79=nagX	SAUR2500 (SAR_RS12500)	0,09	0,71	>=5,00	0,00	0,62	>=5,00	0,00	0,62	>=5,00
>1932	sagA=lytA=atlE	SAUR2502 (SAR_RS12510)	0,00	0,77	0,00	0,13	0,90	0,13	0,13	0,90	0,13
>1933	Q5HDQ1	SAUR2503 (SAR_RS12515)	0,00	0,00	0,00	0,00	0,00	0,00	0,00	0,00	0,00
>1934	yrhD=DUF1641	SAUR2504 (SAR_RS12520)	0,00	>=5,00	1,90	0,00	>=5,00	1,90	0,00	>=5,00	1,90
>1935	fdhL=fdhF	SAUR2505 (SAR_RS12525)	0,03	>=5,00	0,51	0,03	>=5,00	0,51	0,03	>=5,00	0,51
>1936	lytR2=lcpC	SAUR2506 (SAR_RS12530)	0,00	0,52	0,31	0,00	0,52	0,31	0,00	0,52	0,31
>1937	suhB2	SAUR2507 (SAR_RS12535)	0,00	1,38	0,00	0,00	1,38	0,00	0,00	1,38	0,00
>1938	Q5HDP6	SAUR2508 (SAR_RS12540)	0,14	1,15	0,14	0,00	1,01	0,00	0,00	1,01	0,00
>1939	Q5HDP4=sdpB	SAUR2509 (SAR_RS12545)	0,00	1,49	1,36	0,00	1,49	1,36	0,14	1,63	1,49
>1940	Q5HDP3	SAUR2510 (SAR_RS12550)	0,00	1,21	0,30	0,00	1,21	0,30	0,00	1,21	0,30
>1941	rpiRC	SAUR2511 (SAR_RS12555)	0,11	0,46	0,23	0,11	0,46	0,23	0,00	0,34	0,11
>1942	proY	SAUR2512 (SAR_RS12560)	0,00	0,72	0,51	0,00	0,72	0,51	0,07	0,80	0,58
>1943	Q5HDP0	SAUR2513 (SAR_RS12565)	0,00	0,56	>=5,00	0,00	0,56	>=5,00	0,00	0,56	>=5,00
>1944	Q5HDN9	SAUR2514 (SAR_RS12570)	0,00	0,00	0,00	0,00	0,00	0,00	0,00	0,00	0,00
>1945	Q5HDN8	SAUR2515 (SAR_RS12575)	0,00	1,06	0,53	0,00	1,06	0,53	0,00	1,06	0,53
>1946	glpW	SAUR2516 (SAR_RS12580)	0,00	0,79	0,31	0,16	0,94	0,47	0,00	0,79	0,31
>1947	yocS	SAUR2517 (SAR_RS12585)	0,00	0,87	0,65	0,11	0,98	0,76	0,11	0,98	0,76
>1948	Q5HDN5	SAUR2518 (SAR_RS12590)	0,00	0,75	0,00	0,38	1,13	0,38	0,00	0,75	0,00
>1949	glvC	SAUR2519 (SAR_RS12595)	>=5,00	>=5,00	0,00	>=5,00	>=5,00	0,00	>=5,00	>=5,00	0,00
>1950	glvR	SAUR2520 (SAR_RS12600)	0,00	>=5,00	0,26	0,00	>=5,00	0,26	0,00	>=5,00	0,26
>1951	Q5HDN2	SAUR2521 (SAR_RS12605)	0,00	0,20	0,39	0,00	0,20	0,39	0,00	0,20	0,39
>1952	nhaC=metT=mleN	(N/A)	0,14	0,72	1,09	0,14	0,72	1,09	0,22	0,65	1,01
>1953	Q5HDN0	SAUR2523 (SAR_RS12615)	0,00	>=5,00	>=5,00	0,00	>=5,00	>=5,00	0,00	>=5,00	>=5,00
>1954	yhxD=yghA	SAUR2524 (SAR_RS12620)	0,00	0,68	0,00	0,00	0,68	0,00	0,00	0,68	0,00
>1955	Q5HDM8=yxeP	SAUR2525 (SAR_RS12625)	0,18	2,14	2,85	0,36	2,32	3,03	0,18	2,14	2,85
>1956	hutI	SAUR2527 (SAR_RS12635)	0,00	1,21	0,81	0,00	1,21	0,81	0,08	1,29	0,73
>1957	hutU	SAUR2528 (SAR_RS12640)	0,12	0,72	0,48	0,24	0,84	0,60	0,18	0,78	0,54
>1958	hutR	SAUR2529 (SAR_RS12645)	0,11	2,02	0,45	0,34	2,25	0,67	0,11	2,02	0,45
>1959	fosB	(N/A)	0,24	1,43	>=5,00	0,24	1,43	>=5,00	>=5,00	>=5,00	0,00
>1960	hutG	SAUR2531 (SAR_RS12655)	0,00	0,85	0,64	0,00	0,85	0,64	0,00	0,85	0,64
>1961	lyrA=sdpC	SAUR2532 (SAR_RS12660)	0,00	1,27	1,11	0,00	1,27	1,11	0,00	1,27	1,11
>1962	rpiA	SAUR2533 (SAR_RS12665)	0,00	1,02	0,58	0,00	1,02	0,58	0,29	1,31	0,87
>1963	yflK=yiiM	SAUR2534 (SAR_RS12670)	0,00	1,07	0,15	0,00	1,07	0,15	0,15	1,22	0,31
>1964	galM	SAUR2536 (SAR_RS12680)	0,00	0,59	0,10	>=5,00	>=5,00	>=5,00	0,00	0,59	0,10
>1965	ynfA	SAUR2537 (SAR_RS12685)	0,00	0,00	0,00	0,00	0,00	0,00	0,00	0,00	0,00
>1966	yhaP	SAUR2538 (SAR_RS12690)	0,00	0,65	0,16	0,00	0,65	0,16	0,00	0,65	0,16
>1967	yhaQ	SAUR2539 (SAR_RS12695)	0,11	1,22	0,33	0,44	1,56	0,67	0,11	1,22	0,33
>1968	Q5HDL3=yhaI	SAUR2540 (SAR_RS12700)	0,00	>=5,00	>=5,00	0,15	>=5,00	>=5,00	0,00	>=5,00	>=5,00
>1969	yxlJ	SAUR2541 (SAR_RS12705)	0,00	0,66	0,49	0,00	0,66	0,49	0,00	0,66	0,49
>1970	gltS	SAUR2542 (SAR_RS12710)	0,08	1,41	0,83	0,08	1,41	0,83	0,17	1,49	0,91

Number/ Position in the figures	Gene ID	ID in PubMLST cg scheme	ED133 vs. swan isolate 15V8707	X22 vs. swan isolate 15V8707	CC522 goat isolate 17CS1042 vs. swan isolate 15V8707	ED133 vs. duck isolate V315	X22 vs. duck isolate V315	CC522 goat isolate 17CS1042 vs. duck isolate V315	ED133 vs. duck isolate V482	X22 vs. duck isolate V482	CC522 goat isolate 17CS1042 vs. duck isolate V482
>1971	fni=idi2	SAUR2543 (SAR_RS12715)	0,00	0,95	0,38	0,00	0,95	0,38	0,00	0,95	0,38
>1972	corA	SAUR2544 (SAR_RS12720)	0,00	0,74	0,63	0,00	0,74	0,63	0,00	0,74	0,63
>1973	Q5HDK8	SAUR2545 (SAR_RS12725)	0,20	0,39	0,39	0,20	0,39	0,39	0,20	0,39	0,39
>1974	Q5HDK7	SAUR2546 (SAR_RS12730)	0,00	1,19	>=5,00	0,00	1,19	>=5,00	0,00	1,19	>=5,00
>1975	mlhB	(N/A)	0,00	0,66	0,33	0,00	0,66	0,33	0,00	0,66	0,33
>1976	Q5HDK5	SAUR2548 (SAR_RS12740)	0,16	1,10	0,78	0,16	1,10	0,78	0,16	1,10	0,78
>1977	semB	SAUR2549 (SAR_RS12745)	0,00	>=5,00	0,16	0,00	>=5,00	0,16	0,00	>=5,00	0,16
>1978	semA	SAUR2550 (SAR_RS12750)	0,00	>=5,00	0,31	0,00	>=5,00	0,31	0,00	>=5,00	0,31
>1979	Q5HDK2	SAUR2551 (SAR_RS12755)	0,00	1,26	0,00	0,00	1,26	0,00	0,00	1,26	0,00
>1980	tcaB	SAUR2553 (SAR_RS12765)	0,08	1,32	0,66	0,00	1,24	0,58	0,00	1,24	0,58
>1981	tcaA	SAUR2555 (SAR_RS12775)	0,22	0,65	0,22	0,29	0,72	0,29	0,36	0,80	0,36
>1982	tcaR	SAUR2557 (SAR_RS12785)	0,00	0,22	0,22	0,00	0,22	0,22	0,00	0,22	0,22
>1983	Q5HDJ7	SAUR2558 (SAR_RS12790)	0,00	0,72	0,00	0,00	0,72	0,00	0,00	0,72	0,00
>1984	hrtA	SAUR2559 (SAR_RS12795)	0,15	1,05	>=5,00	0,15	1,05	>=5,00	0,15	1,05	>=5,00
>1985	hrtB	SAUR2560 (SAR_RS12800)	0,00	1,23	0,47	0,19	1,42	0,66	0,00	1,23	0,47
>1986	hssR	SAUR2561 (SAR_RS12805)	0,00	0,89	0,59	0,00	0,89	0,59	0,00	0,89	0,59
>1987	hssS	SAUR2562 (SAR_RS12810)	0,07	1,24	0,87	0,07	1,24	0,87	0,07	1,24	0,87
>1988	lytT	SAUR2563 (SAR_RS12815)	0,00	1,59	0,23	0,00	1,59	0,23	0,23	1,81	0,45
>1989	Q5HDJ1=DUF3021	SAUR2564 (SAR_RS12820)	0,00	4,61	0,88	0,00	4,61	0,88	0,00	4,61	0,88
>1990	mqo	SAUR2568 (SAR_RS12840)	0,00	1,08	0,14	0,00	1,08	0,14	0,00	1,08	0,14
>1991	lctP-locus2	SAUR2569 (SAR_RS12845)	0,00	0,63	0,00	0,00	0,63	0,00	0,00	0,63	0,00
>1992	tagF	(N/A)	0,00	0,72	0,66	0,00	0,72	0,66	0,00	0,72	0,66
>1993	Q5HDI7	SAUR2571 (SAR_RS12855)	0,00	0,63	0,32	0,16	0,79	0,48	0,00	0,63	0,32
>1994	paiA	SAUR2572 (SAR_RS12860)	0,19	1,16	0,78	0,00	0,97	0,58	>=5,00	>=5,00	>=5,00
>1995	yhfP=A1KWX9	(N/A)	0,00	0,70	0,20	0,00	0,70	0,20	0,00	0,70	0,20
>1996	Q5HDI4	SAUR2574 (SAR_RS12870)	0,00	>=5,00	1,99	0,25	>=5,00	2,24	0,00	>=5,00	1,99
>1997	iruO	SAUR2575 (SAR_RS12875)	0,19	1,06	0,58	0,10	0,97	0,48	0,10	0,97	0,48
>1998	Q5HDI2	SAUR2577 (SAR_RS12885)	0,00	0,00	0,00	0,00	0,00	0,00	0,00	0,00	0,00
>1999	pip	SAUR2579 (SAR_RS12895)	0,00	0,94	0,23	0,00	0,94	0,23	0,00	0,94	0,23
>2000	Q5HDH9=tetR21	SAUR2580 (SAR_RS12900)	0,00	0,64	0,48	0,00	0,64	0,48	0,00	0,64	0,48
>2001	cobI	SAUR2581 (SAR_RS12905)	0,00	0,42	0,00	0,00	0,42	0,00	0,00	0,42	0,00
>2002	scrA	SAUR2582 (SAR_RS12910)	0,00	1,04	0,35	0,00	1,04	0,35	0,07	1,11	0,42
>2003	DUF1722	SAUR2583 (SAR_RS12915)	0,26	1,04	0,52	0,26	1,04	0,52	0,52	1,30	0,78
>2004	rsp=araC	SAUR2584 (SAR_RS12920)	0,05	1,28	0,14	0,00	1,23	0,09	0,05	1,28	0,14
>2005	ydaG	SAUR2585 (SAR_RS12925)	0,00	>=5,00	0,47	0,00	>=5,00	0,47	0,00	>=5,00	0,47
>2006	Q5HDH2=DUF4889	(N/A)	0,00	>=5,00	0,00	0,00	>=5,00	0,00	0,00	>=5,00	0,00
>2007	gltT	SAUR2588 (SAR_RS12940)	0,00	0,39	0,39	0,00	0,39	0,39	0,00	0,39	0,39
>2008	DUF3139	SAUR2589 (SAR_RS12945)	0,27	1,37	0,82	0,00	1,09	0,55	0,27	1,37	0,82
>2009	sarZ	SAUR2590 (SAR_RS12950)	0,00	0,22	0,00	0,22	0,45	0,22	0,00	0,22	0,00
>2010	hsp20	(N/A)	0,23	1,40	0,93	0,00	1,17	0,70	0,00	1,17	0,70
>2011	narK	SAUR2592 (SAR_RS12960)	0,00	1,71	0,17	0,00	1,71	0,17	0,00	1,71	0,17
>2012	nreC	SAUR2595 (SAR_RS12975)	0,00	0,31	0,00	0,00	0,31	0,00	0,00	0,31	0,00
>2013	nreB	SAUR2596 (SAR_RS12980)	0,00	0,48	0,00	0,00	0,48	0,00	0,00	0,48	0,00
>2014	nreA	SAUR2597 (SAR_RS12985)	0,00	0,44	0,22	0,00	0,44	0,22	0,00	0,44	0,22
>2015	narI	SAUR2598 (SAR_RS12990)	0,00	1,18	0,29	0,00	1,18	0,29	0,00	1,18	0,29
>2016	narJ	SAUR2599 (SAR_RS12995)	0,00	0,17	0,00	0,00	0,17	0,00	0,00	0,17	0,00
>2017	narH	SAUR2600 (SAR_RS13000)	0,00	0,96	>=5,00	0,00	0,96	>=5,00	0,06	1,02	>=5,00
>2018	narG	SAUR2601 (SAR_RS13005)	0,00	0,89	1,00	0,03	0,92	1,03	0,00	0,89	1,00
>2019	nasF=cobA	SAUR2602 (SAR_RS13010)	0,00	1,64	0,92	0,00	1,64	0,92	0,00	1,64	0,92
>2020	nasE=nirD	SAUR2603 (SAR_RS13015)	0,00	0,63	0,32	0,00	0,63	0,32	0,00	0,63	0,32
>2021	nasD=nirB	SAUR2604 (SAR_RS13020)	0,00	0,96	0,21	0,00	0,96	0,21	0,00	0,96	0,21
>2022	nirR=nasR=cbiX	SAUR2605 (SAR_RS13025)	0,00	>=5,00	>=5,00	0,00	>=5,00	>=5,00	0,00	>=5,00	>=5,00
>2023	Q5HDF4=ytml	SAUR2606 (SAR_RS13030)	0,00	>=5,00	1,47	0,00	>=5,00	1,47	0,00	>=5,00	1,47
>2024	focA-L2=nirC	SAUR2607 (SAR_RS13035)	0,00	>=5,00	0,48	0,00	>=5,00	0,48	0,00	>=5,00	0,48
>2025	Q8NV18	(N/A)	0,00	1,09	0,00	0,00	1,09	0,00	0,00	1,09	0,00
>2026	Q5HDF2	SAUR2609 (SAR_RS13045)	0,00	1,71	0,64	0,00	1,71	0,64	0,00	1,71	0,64
>2027	zinT=adcA	SAUR2610 (SAR_RS13050)	0,26	0,90	0,58	0,19	0,90	0,51	0,19	0,90	0,51
>2028	yoeB1	(N/A)	0,00	1,87	2,25	0,37	2,25	2,62	0,00	1,87	2,25
>2029	yefM1	(N/A)	0,00	0,40	1,59	0,00	0,40	1,59	0,00	0,40	1,59
>2030	dsbA	SAUR2614 (SAR_RS13070)	0,17	1,00	0,83	0,00	0,83	0,67	0,17	1,00	0,83
>2031	DUF4467	SAUR2615 (SAR_RS13075)	>=5,00	>=5,00	>=5,00	0,26	0,26	0,26	0,00	0,00	0,00
>2032	fmhA	SAUR2616 (SAR_RS13080)	0,00	1,60	0,40	0,08	1,68	0,48	0,00	1,60	0,40
>2033	tcyC	SAUR2617 (SAR_RS13085)	0,00	0,27	0,14	0,00	0,27	0,14	0,00	0,27	0,14
>2034	tcyB	SAUR2618 (SAR_RS13090)	0,14	0,27	0,41	0,00	0,14	0,27	0,00	0,14	0,27
>2035	tcyA	SAUR2619 (SAR_RS13095)	0,51	1,15	1,28	0,00	0,64	0,77	0,00	0,64	0,77
>2036	mdeA	SAUR2620 (SAR_RS13100)	0,00	1,32	0,90	0,00	1,32	0,90	0,07	1,39	0,97
>2037	gpmA2	SAUR2622 (SAR_RS13110)	0,00	0,73	0,29	0,00	0,73	0,29	0,00	0,73	0,29
>2038	cdf	SAUR2623 (SAR_RS13115)	0,00	0,58	0,46	0,12	0,69	0,58	0,00	0,58	0,46
>2039	sbi	SAUR2625 (SAR_RS13125)	0,00	>=5,00	>=5,00	3,38	3,24	3,53	0,00	>=5,00	>=5,00
>2040	hlgA	SAUR2627 (SAR_RS13135)	0,11	0,54	0,11	0,11	0,54	0,11	0,11	0,54	0,11
>2041	lukS=hlgC	SAUR2628 (SAR_RS13140)	0,00	0,52	0,21	0,00	0,52	0,21	1,15	1,67	1,36
>2042	lukF=hlgB	SAUR2629 (SAR_RS13145)	0,00	0,82	0,10	0,00	0,82	0,10	0,00	0,82	0,10
>2043	bioX	SAUR2630 (SAR_RS13150)	0,00	1,28	0,64	0,00	1,28	0,64	0,00	1,28	0,64
>2044	bioW	SAUR2631 (SAR_RS13155)	0,00	1,30	1,15	0,00	1,30	1,15	0,00	1,30	1,15
>2045	bioF	SAUR2632 (SAR_RS13160)	0,00	1,17	1,17	0,00	1,17	1,17	0,00	1,17	1,17
>2046	bioB	SAUR2633 (SAR_RS13165)	0,00	1,09	0,99	0,00	1,09	0,99	0,00	1,09	0,99
>2047	bioA	SAUR2634 (SAR_RS13170)	0,00	1,69	1,47	0,00	1,69	1,47	0,00	1,69	1,47
>2048	bioD	SAUR2635 (SAR_RS13175)	0,00	3,97	1,42	0,00	3,97	1,42	0,00	3,97	1,42
>2049	Q5HDC6-L1	SAUR2637 (SAR_RS13185)	0,00	>=5,00	0,00	0,00	>=5,00	0,00	0,00	>=5,00	0,00
>2050	msbA3	(N/A)	0,00	2,48	>=5,00	0,00	2,48	>=5,00	0,00	2,48	>=5,00
>2051	msbA4	(N/A)	0,06	0,74	>=5,00	0,00	0,68	>=5,00	0,00	0,68	>=5,00
>2052	Q5HDC6-L2	SAUR2640 (SAR_RS13200)	0,00	0,93	0,00	0,00	0,93	0,00	0,00	0,93	0,00
>2053	ldr_fst_L2	(N/A)	0,00	0,00	0,00	0,00	0,00	0,00	0,00	0,00	0,00
>2054	gtrA=gtxA	SAUR2642 (SAR_RS13210)	0,26	2,84	0,26	0,52	3,10	0,52	0,26	2,84	0,26
>2055	glxK1	SAUR2643 (SAR_RS13215)	0,00	0,52	0,96	0,09	0,61	1,05	0,00	0,52	0,96
>2056	Q5HDC0	SAUR2644 (SAR_RS13220)	0,00	1,97	0,91	0,15	2,12	1,06	0,00	1,97	0,91
>2057	bcr	SAUR2645 (SAR_RS13225)	0,00	1,32	0,41	0,00	1,32	0,41	0,00	1,32	0,41
>2058	aarP	SAUR2646 (SAR_RS13230)	0,00	>=5,00	0,21	0,00	>=5,00	0,21	0,00	>=5,00	0,21
>2059	Q5HDB7	SAUR2647 (SAR_RS13235)	0,15	1,07	0,46	0,30	1,22	0,61	0,30	1,22	0,61
>2060	Q5HDB6	SAUR2648 (SAR_RS13240)	0,00	>=5,00	0,25	0,00	>=5,00	0,25	0,00	>=5,00	0,25
>2061	aapA3=cycA	SAUR2649 (SAR_RS13245)	0,00	1,06	0,57	0,07	1,13	0,64	0,00	1,06	0,57
>2062	nhaK2	SAUR2650 (SAR_RS13250)	0,00	1,25	0,43	0,00	1,25	0,43	0,05	1,30	0,48
>2063	ydaO	SAUR2652 (SAR_RS13260)	0,00	0,98	0,16	0,00	0,98	0,16	0,00	0,98	0,16
>2064	flp=fmtA2	SAUR2654 (SAR_RS13270)	0,00	4,00	2,00	0,00	4,00	2,00	0,00	4,00	2,00
>2065	rfbD	SAUR2655 (SAR_RS13275)	0,00	0,59	0,70	0,00	0,59	0,70	0,00	0,59	0,70
>2066	panE1	SAUR2657 (SAR_RS13285)	0,00	1,39	0,11	0,00	1,39	0,11	0,00	1,39	0,11
>2067	mmr=norB	SAUR2658 (SAR_RS13290)	0,07	1,14	0,21	0,00	1,07	0,14	0,00	1,07	0,14
>2068	opuCD	SAUR2659 (SAR_RS13295)	0,00	1,44	0,57	0,14	1,58	0,72	0,00	1,44	0,57
>2069	opuCC	SAUR2660 (SAR_RS13300)	0,00	1,17	0,42	0,00	1,17	0,42	0,00	1,17	0,42
>2070	opuCB	SAUR2661 (SAR_RS13305)	0,00	1,42	0,16	0,00	1,42	0,16	0,00	1,42	0,16
>2071	opuCA	SAUR2662 (SAR_RS13310)	0,24	1,21	0,24	0,00	0,97	0,00	0,00	0,97	0,00
>2072	ydeI=opuCB=groEL	SAUR3004	0,00	1,33	0,00	0,00	1,33	0,00	0,00	1,33	0,00
>2073	ybeC=yveA	SAUR3005	0,06	1,16	0,61	0,00	1,10	0,55	0,00	1,10	0,55
>2074	pnbA	SAUR2667 (SAR_RS13335)	0,00	0,81	0,22	0,00	0,81	0,22	0,07	0,89	0,30
>2075	pbuE	SAUR2668 (SAR_RS13340)	0,00	0,84	0,67	0,00	0,84	0,67	0,00	0,84	0,67
>2076	yjkA=ybbM=fetB	SAUR2669 (SAR_RS13345)	0,00	>=5,00	0,13	0,00	>=5,00	0,13	0,00	>=5,00	0,13
>2077	yjkB	SAUR2670 (SAR_RS13350)	0,00	>=5,00	>=5,00	0,00	>=5,00	>=5,00	0,00	>=5,00	>=5,00
>2078	pepA2	SAUR2671 (SAR_RS13355)	0,19	2,97	0,56	0,28	3,06	0,65	0,09	2,88	0,46
>2079	Q5HD92	SAUR2672 (SAR_RS13360)	2,99	0,43	0,43	2,78	0,21	0,21	2,78	0,21	0,21
>2080	yerD	SAUR2673 (SAR_RS13365)	0,13	1,33	0,38	0,13	1,33	0,38	0,32	1,39	0,57
>2081	Q5HD90	SAUR2674 (SAR_RS13370)	0,13	3,20	0,40	0,53	3,60	0,80	0,27	3,33	0,53
>2082	norD=yhhS	SAUR2675 (SAR_RS13375)	0,00	0,75	0,67	0,00	0,75	0,67	0,00	0,75	0,67
>2083	cntF	SAUR2676 (SAR_RS13380)	0,00	1,20	0,13	0,00	1,20	0,13	0,00	1,20	0,13
>2084	cntD	SAUR2677 (SAR_RS13385)	0,25	4,17	1,10	0,00	3,92	0,86	0,00	3,92	0,86
>2085	cntC	SAUR2678 (SAR_RS13390)	0,00	0,69	0,34	>=5,00	>=5,00	>=5,00	0,00	0,69	0,34
>2086	cntB	SAUR2679 (SAR_RS13395)	0,00	0,53	0,00	0,00	0,53	0,00	0,00	0,53	0,00
>2087	cntA	SAUR2680 (SAR_RS13400)	0,06	1,19	0,13	0,00	1,13	0,06	0,00	1,13	0,06
>2088	DUF2338=cntM	(N/A)	0,00	1,63	>=5,00	0,00	1,63	>=5,00	0,00	1,63	>=5,00
>2089	fmrO=cntL	SAUR2682 (SAR_RS13410)	0,12	0,48	0,36	0,12	0,48	0,36	0,73	1,09	0,97
>2090	dapF=cntK	SAUR2683 (SAR_RS13415)	0,12	0,97	0,12	0,24	1,09	0,24	0,12	0,97	0,12
>2091	yxbG	SAUR2685 (SAR_RS13425)	0,00	>=5,00	2,69	0,00	>=5,00	2,69	>=5,00	>=5,00	>=5,00
>2092	Q5HD78=abgT	SAUR2686 (SAR_RS13430)	0,00	2,08	0,97	0,06	2,14	1,04	>=5,00	>=5,00	>=5,00
>2093	aphD	SAUR2687 (SAR_RS13435)	0,00	0,95	0,00	0,00	0,95	0,00	0,00	0,95	0,00
>2094	bdhA	SAUR2694 (SAR_RS13470)	0,14	1,72	0,43	0,14	1,72	0,43	0,14	1,72	0,43
>2095	iraB=DUF1413	SAUR2695 (SAR_RS13475)	0,00	>=5,00	0,35	0,00	>=5,00	0,35	0,00	>=5,00	0,35
>2096	Q5HD71	SAUR2697 (SAR_RS13485)	0,00	0,00	1,56	0,00	0,00	1,56	0,00	0,00	1,56
>2097	pgcA	SAUR2704 (SAR_RS13520)	0,00	3,24	1,40	0,00	3,24	1,40	0,00	3,24	1,40
>2098	Q5HD60	SAUR2705 (SAR_RS13525)	0,00	0,00	0,75	1,87	1,87	2,62	0,37	0,37	1,12
>2099	Q5HD59	SAUR2706 (SAR_RS13530)	0,00	3,86	>=5,00	0,00	3,86	>=5,00	0,00	3,86	>=5,00
>2101	sarT	(N/A)	0,00	0,00	>=5,00	0,00	0,00	>=5,00	0,00	0,00	>=5,00
>2102	sarU	(N/A)	0,13	0,13	>=5,00	0,00	0,00	>=5,00	0,00	0,00	>=5,00
>2103	gtaB=galU	SAUR2707 (SAR_RS13535)	0,00	1,85	1,73	0,00	1,85	1,73	0,00	1,85	1,73
>2104	fnbB	(N/A)	0,00	>=5,00	>=5,00	>=5,00	>=5,00	0,00	0,00	>=5,00	>=5,00
>2105	Q5HD52	(N/A)	0,81	>=5,00	>=5,00	>=5,00	>=5,00	0,00	1,63	>=5,00	>=5,00
>2106	fnbA	(N/A)	1,39	>=5,00	>=5,00	>=5,00	>=5,00	>=5,00	1,39	>=5,00	>=5,00
>2107	Q2G207	(N/A)	0,50	0,50	1,00	0,50	0,50	1,00	0,00	0,00	0,50
>2108	gntP	SAUR3006 (gntP)	0,00	1,32	0,88	0,00	1,32	0,88	0,07	1,40	0,96
>2109	gntK	SAUR3007 (gntK)	0,06	2,06	1,54	0,00	1,99	1,48	0,00	1,99	1,48
>2110	gntR=ydfH	(N/A)	0,00	0,88	1,03	0,00	0,88	1,03	0,00	0,88	1,03
>2111	merR3=bmrR=zntR	SAUR3009	0,13	3,53	>=5,00	0,00	3,40	>=5,00	0,00	3,40	>=5,00
>2112	relP	SAUR3010	0,29	1,01	0,87	0,14	0,87	0,72	0,14	0,87	0,72
>2113	DUF2188	SAUR3011	>=5,00	0,00	2,66	>=5,00	0,00	2,66	>=5,00	0,20	2,86
>2114	yccS	(N/A)	0,00	1,95	1,80	0,00	1,95	1,80	0,11	2,06	1,90
>2115	ycbE=garP	SAUR3012	0,00	0,55	0,94	0,00	0,55	0,94	0,00	0,55	0,94
>2116	dedA	SAUR2719 (SAR_RS13595)	0,00	1,63	0,65	0,00	1,63	0,65	0,00	1,63	0,65
>2117	stp	SAUR2723 (SAR_RS13615)	0,00	0,91	>=5,00	0,13	1,04	>=5,00	0,00	0,91	>=5,00
>2118	smp	SAUR2724 (SAR_RS13620)	0,00	1,43	0,13	0,13	1,56	0,26	0,13	1,56	0,26
>2119	fbp	SAUR2725 (SAR_RS13625)	0,00	>=5,00	1,02	0,05	>=5,00	1,07	0,05	>=5,00	1,07

Number/ Position in the figures	Gene ID	ID in PubMLST cg scheme	ED133 vs. swan isolate 15V8707	X22 vs. swan isolate 15V8707	CC522 goat isolate 17CS1042 vs. swan isolate 15V8707	ED133 vs. duck isolate V315	X22 vs. duck isolate V315	CC522 goat isolate 17CS1042 vs. duck isolate V315	ED133 vs. duck isolate V482	X22 vs. duck isolate V482	CC522 goat isolate 17CS1042 vs. duck isolate V482
>2120	Q1Y5K2	SAUR2726 (SAR_RS13630)	0,00	1,85	0,74	0,00	1,85	0,74	0,09	1,94	0,83
>2121	Q1Y5K1	SAUR2727 (SAR_RS13635)	0,00	2,53	1,01	0,17	2,69	1,18	0,00	2,53	1,01
>2122	mhqA-2	SAUR2728 (SAR_RS13640)	0,10	2,27	1,44	0,00	2,17	1,34	0,10	2,27	1,44
>2123	mhqR	SAUR2729 (SAR_RS13645)	0,23	0,46	0,23	0,23	0,46	0,23	0,23	0,46	0,23
>2124	Q5HD32	SAUR2730 (SAR_RS13650)	0,00	2,81	0,00	0,00	2,81	0,00	0,00	2,81	0,00
>2125	catE	SAUR2731 (SAR_RS13655)	0,25	1,49	1,49	0,00	1,24	1,24	0,00	1,24	1,24
>2126	frp	SAUR2732 (SAR_RS13660)	0,15	1,19	1,04	0,15	1,19	1,04	0,15	1,19	1,04
>2127	ldhD=ddh_L1	SAUR2733 (SAR_RS13665)	0,10	0,60	0,60	0,10	0,60	0,60	0,10	0,60	0,60
>2128	ywtE	SAUR2736 (SAR_RS13680)	0,00	3,00	1,62	0,00	3,00	1,62	0,00	3,00	1,62
>2129	srtA	SAUR2737 (SAR_RS13685)	0,00	0,48	0,00	0,00	0,48	0,00	0,00	0,48	0,00
>2130	ywnH=yncA	SAUR2738 (SAR_RS13690)	0,20	1,63	0,61	0,00	1,42	0,41	0,00	1,42	0,41
>2131	sdaA	SAUR2741 (SAR_RS13705)	0,00	0,78	0,56	0,00	0,78	0,56	0,00	0,78	0,56
>2132	sdaB	SAUR2742 (SAR_RS13710)	0,00	0,44	0,00	0,00	0,44	0,00	0,00	0,44	0,00
>2133	ptsEIIC	SAUR2743 (SAR_RS13715)	0,00	0,29	0,67	0,00	0,29	0,67	0,00	0,29	0,67
>2134	Q5HD18	SAUR2744 (SAR_RS13720)	0,00	0,49	0,00	0,00	0,49	0,00	0,00	0,49	0,00
>2135	ydeD=yicL	(N/A)	0,00	0,86	1,40	0,22	1,08	1,62	0,00	0,86	1,40
>2136	aes	SAUR2746 (SAR_RS13730)	0,00	1,55	0,52	0,10	1,65	0,62	0,00	1,55	0,52
>2137	Q5HD15	SAUR2747 (SAR_RS13735)	0,00	0,28	4,76	0,00	0,28	4,76	0,00	0,28	4,76
>2138	Q5HD14	SAUR2748 (SAR_RS13740)	0,00	1,46	0,49	0,00	1,46	0,49	0,00	1,46	0,49
>2139	glcB=ptsG	SAUR2749 (SAR_RS13745)	0,00	1,16	0,87	0,15	1,31	1,02	0,00	1,16	0,87
>2140	cidC=pox	SAUR2750 (SAR_RS13750)	0,06	4,37	0,40	0,11	4,43	0,46	0,06	4,37	0,40
>2141	cidB	SAUR2751 (SAR_RS13755)	0,00	0,72	4,06	0,00	0,72	4,06	0,00	0,72	4,06
>2142	cidA	SAUR2752 (SAR_RS13760)	0,00	0,76	>=5,00	0,00	0,76	>=5,00	0,00	0,76	>=5,00
>2143	cidR	SAUR2753 (SAR_RS13765)	0,00	0,46	0,11	0,00	0,46	0,11	0,11	0,57	0,23
>2144	Q2FV82	(N/A)	0,00	0,00	0,00	0,00	0,00	0,00	0,00	0,00	0,00
>2145	ssaA4	SAUR2755 (SAR_RS13775)	0,23	0,23	0,23	0,23	0,23	0,23	0,23	0,23	0,23
>2146	mvaA	SAUR2756 (SAR_RS13780)	0,00	4,76	0,70	0,00	4,76	0,70	0,08	4,84	0,78
>2147	mvaS	SAUR2757 (SAR_RS13785)	0,09	2,74	0,00	0,09	2,74	0,00	0,09	2,74	0,00
>2148	adaB	SAUR2758 (SAR_RS13790)	0,00	2,49	1,34	0,00	2,49	1,34	0,00	2,49	1,34
>2149	clpL	SAUR2759 (SAR_RS13795)	0,09	>=5,00	1,66	0,05	>=5,00	1,61	0,05	>=5,00	1,61
>2150	A8Z3E9	(N/A)	0,00	4,07	1,74	0,00	4,07	1,74	0,00	4,07	1,74
>2151	feoB	(N/A)	0,00	1,15	0,35	0,00	1,15	0,35	0,00	1,15	0,35
>2152	feoA	SAUR3013	0,00	0,88	0,88	0,00	0,88	0,88	0,44	1,32	1,32
>2153	mmpL=farE	SAUR3014	0,00	>=5,00	>=5,00	0,04	>=5,00	>=5,00	0,00	>=5,00	>=5,00
>2154	Q8NUR3=farR	SAUR2764 (SAR_RS13820)	0,00	>=5,00	0,73	0,00	>=5,00	0,73	0,00	>=5,00	0,73
>2155	Q5HCZ7	SAUR2765 (SAR_RS13825)	0,00	1,19	2,38	0,00	1,19	2,38	>=5,00	>=5,00	>=5,00
>2156	rocA=pruA	SAUR2766 (SAR_RS13830)	0,00	1,29	0,71	0,00	1,29	0,71	0,00	1,29	0,71
>2157	maa	SAUR2767 (SAR_RS13835)	0,00	>=5,00	2,00	0,00	>=5,00	2,00	0,00	>=5,00	2,00
>2158	cwrA	SAUR2768 (SAR_RS13840)	0,00	3,13	0,00	0,00	3,13	0,00	0,00	3,13	0,00
>2159	copA	SAUR2769 (SAR_RS13845)	0,08	0,87	0,46	0,08	0,87	0,46	0,12	0,91	0,50
>2160	copZ	SAUR2770 (SAR_RS13850)	0,00	0,97	0,48	0,00	0,97	0,48	0,00	0,97	0,48
>2161	ldhD=ddh_L2	SAUR2771 (SAR_RS13855)	>=5,00	>=5,00	>=5,00	0,10	0,60	0,70	>=5,00	>=5,00	>=5,00
>2162	ywfG	SAUR2772 (SAR_RS13860)	0,00	1,99	0,61	0,00	1,99	0,61	0,00	1,99	0,61
>2163	crtN	SAUR2773 (SAR_RS13865)	0,00	1,72	0,66	0,00	1,72	0,66	0,00	1,72	0,66
>2164	crtM	SAUR2774 (SAR_RS13870)	0,12	0,93	0,46	0,00	0,81	0,35	0,00	0,81	0,35
>2165	crtQ	SAUR2775 (SAR_RS13875)	0,00	1,06	0,35	0,00	1,06	0,35	0,00	1,06	0,35
>2166	crtP	SAUR2776 (SAR_RS13880)	0,00	1,14	0,33	0,00	1,14	0,33	0,07	1,20	0,40
>2167	crtO	SAUR2777 (SAR_RS13885)	0,00	2,61	1,00	0,00	2,61	1,00	0,00	2,61	1,00
>2168	ssaA1	SAUR2778 (SAR_RS13890)	0,00	1,69	0,52	0,00	1,69	0,52	0,00	1,69	0,52
>2169	oatA	SAUR2779 (SAR_RS13895)	0,00	1,88	0,94	0,00	1,88	0,94	0,00	1,88	0,94
>2170	isaA	SAUR2780 (SAR_RS13900)	0,00	0,71	0,00	0,14	0,85	0,14	0,00	0,71	0,00
>2171	Q5HCY0	SAUR2781 (SAR_RS13905)	0,00	1,14	0,09	0,00	1,14	0,09	0,00	1,14	0,09
>2172	acrA	(N/A)	0,00	1,23	0,53	0,18	1,40	0,70	0,00	1,23	0,53
>2173	Q5HCX8	SAUR2784 (SAR_RS13920)	0,00	3,79	1,00	0,00	3,79	1,00	0,00	3,79	1,00
>2174	ynzC	SAUR2785 (SAR_RS13925)	0,00	0,87	1,30	0,43	1,30	1,73	0,00	0,87	1,30
>2175	Q5HCX6	SAUR2786 (SAR_RS13930)	0,00	0,79	0,26	0,00	0,79	0,26	0,00	0,79	0,26
>2176	nmrA	SAUR2787 (SAR_RS13935)	0,00	0,85	0,24	0,00	0,85	0,24	0,00	0,85	0,24
>2177	Q5HCX4=DUF2316	SAUR2788 (SAR_RS13940)	0,00	2,00	0,33	0,00	2,00	0,33	0,00	2,00	0,33
>2178	gbaA	SAUR2789 (SAR_RS13945)	0,00	>=5,00	0,18	0,00	>=5,00	0,18	0,00	>=5,00	0,18
>2179	gbaB	SAUR2790 (SAR_RS13950)	0,14	2,27	3,55	0,00	2,13	3,40	0,00	2,13	3,40
>2180	Q5HCX0	SAUR2791 (SAR_RS13955)	0,00	1,19	1,78	0,00	1,19	1,78	0,00	1,19	1,78
>2181	yraK	SAUR2792 (SAR_RS13960)	>=5,00	>=5,00	>=5,00	0,00	1,20	0,36	0,00	1,20	0,36
>2182	cobW3	SAUR2793 (SAR_RS13965)	0,00	2,69	0,79	0,11	2,81	0,90	0,67	2,92	1,23
>2183	Q6GDL8=feoB	SAUR2794 (SAR_RS13970)	0,15	>=5,00	>=5,00	0,15	>=5,00	>=5,00	>=5,00	1,41	0,59
>2184	Q5HCW6	SAUR2795 (SAR_RS13975)	0,00	1,17	0,90	0,09	1,26	0,99	0,81	0,72	0,09
>2185	DUF4176=esaC	SAUR2796 (SAR_RS13980)	0,00	0,34	1,01	0,00	0,34	1,01	1,01	1,35	0,00
>2186	Q8NUP1	SAUR2797 (SAR_RS13985)	0,00	>=5,00	>=5,00	0,00	>=5,00	>=5,00	>=5,00	0,00	0,00
>2187	Q8NUP0	SAUR2798 (SAR_RS13990)	0,00	4,35	0,36	0,00	4,35	0,36	0,36	3,99	0,00
>2188	Q8NUN9	SAUR2799 (SAR_RS13995)	0,00	2,54	1,04	0,12	2,65	1,15	1,04	1,96	0,00
>2189	pyrD	SAUR2800 (SAR_RS14000)	0,00	0,75	0,09	0,00	0,75	0,09	0,19	0,75	0,09
>2190	Q5HCW0	SAUR2801 (SAR_RS14005)	0,00	0,72	0,72	0,00	0,72	0,72	0,72	0,00	0,00
>2191	DUF0208=queH	SAUR2802 (SAR_RS14010)	0,00	0,14	0,00	0,14	0,28	0,14	0,00	0,14	0,00
>2192	Q5HCV8	SAUR2803 (SAR_RS14015)	0,00	1,33	0,44	0,00	1,33	0,44	0,44	0,89	0,00
>2193	pepX	SAUR2806 (SAR_RS14030)	0,00	1,07	0,59	0,00	1,07	0,59	0,59	1,19	0,12
>2194	panD	SAUR2807 (SAR_RS14035)	0,00	0,52	0,00	0,00	0,52	0,00	0,00	0,52	0,00
>2195	panC	SAUR2808 (SAR_RS14040)	0,00	0,35	0,12	0,12	0,47	0,23	0,35	0,70	0,23
>2196	panB	SAUR2809 (SAR_RS14045)	0,00	4,15	3,41	0,00	4,15	3,41	0,00	4,15	3,41
>2197	panE2	SAUR2810 (SAR_RS14050)	0,00	1,28	0,00	0,00	1,28	0,00	0,00	1,28	0,00
>2198	alsD-L2=budA	SAUR2811 (SAR_RS14055)	0,14	0,14	0,28	0,14	0,14	0,28	0,43	0,43	0,28
>2199	ldh2	SAUR2812 (SAR_RS14060)	0,00	0,42	0,21	0,00	0,42	0,21	0,21	0,63	0,21
>2200	yfnA1=pheP	SAUR2814 (SAR_RS14070)	0,07	0,69	0,35	0,07	0,69	0,35	0,48	0,83	0,07
>2201	yhxA=gabT	SAUR2815 (SAR_RS14075)	0,00	1,49	0,67	0,00	1,49	0,67	0,67	1,12	0,00
>2202	Q5HCU7	SAUR2816 (SAR_RS14080)	0,00	1,44	2,16	>=5,00	>=5,00	>=5,00	2,16	1,68	0,00
>2203	fda	SAUR2817 (SAR_RS14085)	0,11	2,92	>=5,00	0,11	2,92	>=5,00	>=5,00	>=5,00	0,00
>2204	lqo	SAUR2818 (SAR_RS14090)	0,07	0,60	0,47	0,07	0,60	0,47	0,53	0,27	0,00
>2205	acsA2=bclA	SAUR2820 (SAR_RS14100)	0,00	0,94	0,56	0,00	0,94	0,56	0,56	1,25	0,00
>2206	Q5HCU3	SAUR2821 (SAR_RS14105)	0,00	2,70	2,03	0,00	2,70	2,03	2,03	1,58	0,00
>2207	Q5HCU2	SAUR2822 (SAR_RS14110)	0,00	0,47	0,47	0,00	0,47	0,47	0,47	0,00	0,00
>2208	betA	SAUR2823 (SAR_RS14115)	0,00	0,53	0,29	0,00	0,53	0,29	>=5,00	>=5,00	>=5,00
>2209	betB	SAUR2824 (SAR_RS14120)	0,07	0,60	0,27	0,07	0,60	0,27	0,27	0,67	0,07
>2210	A8Z5A6	SAUR2825 (SAR_RS14125)	0,00	0,00	0,00	0,00	0,00	0,00	0,00	0,00	0,00
>2211	Q79ZX2=cudC	SAUR2826 (SAR_RS14130)	0,00	0,18	0,53	0,00	0,18	0,53	0,53	0,71	0,00
>2212	cudT	SAUR2827 (SAR_RS14135)	0,00	1,48	1,17	1,23	0,49	0,18	0,00	1,48	1,17
>2213	nrdG	SAUR2830 (SAR_RS14150)	0,00	0,56	0,00	0,00	0,56	0,00	0,00	0,56	0,00
>2214	nrdD	SAUR2831 (SAR_RS14155)	0,05	1,08	0,32	0,00	1,03	0,27	0,27	1,08	0,11
>2215	citM	SAUR2832 (SAR_RS14160)	0,00	1,97	1,02	0,00	1,97	1,02	1,02	2,26	0,00
>2216	cysG=sirC	SAUR2834 (SAR_RS14170)	0,00	>=5,00	0,83	0,00	>=5,00	0,83	0,83	>=5,00	0,00
>2217	cysJ	SAUR2835 (SAR_RS14175)	0,05	0,90	0,58	0,00	0,85	0,53	0,48	0,96	0,05
>2218	gpxA-L2	SAUR2837 (SAR_RS14185)	0,00	0,60	0,00	0,00	0,60	0,00	0,00	0,60	0,00
>2219	A6QKB0	SAUR2838 (SAR_RS14190)	0,40	1,59	0,79	0,00	1,19	0,40	0,40	0,79	0,00
>2220	nsaB	SAUR2841 (SAR_RS14205)	0,00	2,25	1,05	0,10	2,35	1,15	1,20	1,85	0,15
>2221	nsaA=lolD	SAUR2842 (SAR_RS14210)	0,00	2,48	1,70	0,00	2,48	1,70	1,57	1,31	0,13
>2222	nsaS	SAUR2843 (SAR_RS14215)	0,00	0,90	0,23	0,11	1,01	0,11	0,11	0,79	0,11
>2223	nsaR	SAUR2844 (SAR_RS14220)	0,00	0,90	0,15	0,00	0,90	0,15	0,15	0,75	0,00
>2224	Q5HCS2	SAUR2845 (SAR_RS14225)	0,00	0,50	0,00	0,00	0,50	0,00	0,00	0,50	0,00
>2225	phoB	(N/A)	0,00	1,33	0,49	0,00	1,33	0,49	0,28	1,33	0,35
>2226	DUF2648	(N/A)	0,00	0,00	0,98	0,00	0,00	0,98	0,00	0,00	0,98
>2227	slyA	SAUR2849 (SAR_RS14245)	0,00	0,00	0,44	0,22	0,22	0,66	0,66	0,66	0,22
>2228	estA	SAUR2850 (SAR_RS14250)	0,13	1,05	0,79	0,00	0,92	0,66	0,79	0,66	0,13
>2229	clfB	SAUR2851 (SAR_RS14255)	3,14	>=5,00	>=5,00	>=5,00	>=5,00	>=5,00	>=5,00	>=5,00	2,38
>2230	arcR	SAUR2852 (SAR_RS14260)	0,00	0,43	0,43	0,00	0,43	0,43	0,43	0,57	0,00
>2231	arcC-L2	SAUR2853 (SAR_RS14265)	2,89	0,83	0,31	0,00	3,51	2,99	2,99	0,72	0,00
>2232	arcD-L2	SAUR2854 (SAR_RS14270)	0,00	0,70	0,14	0,07	0,77	0,21	0,07	0,77	0,07
>2233	arcB-L2	SAUR2855 (SAR_RS14275)	0,00	0,30	0,20	0,00	0,30	0,20	0,40	0,69	0,20
>2234	arcA-L2	SAUR2856 (SAR_RS14280)	0,08	0,97	0,24	0,08	0,97	0,24	0,32	0,89	0,16
>2235	argR-L2	SAUR2858 (SAR_RS14290)	0,00	0,89	0,67	0,00	0,89	0,67	0,67	0,67	0,00
>2236	aur	SAUR2859 (SAR_RS14295)	0,00	0,59	0,20	0,00	0,59	0,20	0,20	0,65	0,13
>2237	isaB	(N/A)	0,00	0,57	0,19	0,00	0,57	0,19	0,00	0,57	0,19
>2238	Q6G635	SAUR2861 (SAR_RS14305)	0,00	1,96	0,00	0,00	1,96	0,00	0,00	1,96	0,00
>2239	manR	SAUR2862 (SAR_RS14310)	0,05	0,96	0,27	0,00	0,91	0,21	0,27	0,85	0,16
>2240	manP	SAUR2863 (SAR_RS14315)	0,00	0,92	0,67	0,05	0,97	0,72	0,61	1,13	0,15
>2241	manA2=yvyl	SAUR2864 (SAR_RS14320)	0,00	0,96	0,43	0,11	1,06	0,53	0,53	0,64	0,11
>2242	yhgE	SAUR2865 (SAR_RS14325)	0,00	1,68	0,27	0,00	1,68	0,27	0,44	1,64	0,17
>2243	scaH=lytZ	SAUR2866 (SAR_RS14330)	0,00	0,48	0,27	0,05	0,54	0,32	0,22	0,48	0,05
>2244	ywoC1	SAUR2867 (SAR_RS14335)	0,00	0,36	0,36	0,00	0,36	0,36	0,36	0,36	0,00
>2245	sasF	SAUR2868 (SAR_RS14340)	0,21	2,72	1,99	0,16	2,67	1,94	2,15	2,10	0,05
>2246	gtfB	SAUR2869 (SAR_RS14345)	0,00	1,03	0,07	0,00	1,03	0,07	0,07	1,10	0,15
>2247	gtfA=tagE	SAUR2870 (SAR_RS14350)	0,00	1,59	0,07	0,07	1,66	0,13	0,13	1,72	0,20
>2248	secA2	SAUR2871 (SAR_RS14355)	0,00	0,79	0,33	0,04	0,75	0,38	0,33	0,88	0,17
>2249	asp3	(N/A)	0,00	1,67	0,31	0,00	1,67	0,31	0,52	2,19	0,21
>2250	asp2	SAUR2873 (SAR_RS14365)	0,00	1,15	0,57	0,06	1,21	0,64	0,51	1,27	0,06
>2251	asp1	(N/A)	0,00	2,32	0,13	0,06	2,38	0,19	0,19	2,38	0,06
>2252	secY2	SAUR2875 (SAR_RS14375)	0,00	3,63	1,90	0,08	3,71	1,98	1,90	3,88	0,00
>2253	sasA	SAUR2876 (SAR_RS14380)	4,23	>=5,00	>=5,00	3,99	>=5,00	>=5,00	>=5,00	0,00	>=5,00
>2254	A6U540	SAUR2877 (SAR_RS14385)	0,14	>=5,00	0,29	0,14	>=5,00	0,29	0,43	>=5,00	0,00
>2255	Q5HCP0	SAUR2880 (SAR_RS14400)	0,33	>=5,00	>=5,00	0,66	>=5,00	>=5,00	>=5,00	>=5,00	0,00
>2256	Q5HCN9	SAUR2881 (SAR_RS14405)	0,00	2,50	2,22	0,00	2,50	2,22	2,22	3,89	0,00
>2257	ywrF	SAUR2882 (SAR_RS14410)	0,00	1,63	0,49	0,33	1,96	0,16	0,49	2,12	0,00
>2258	msrA3	SAUR2883 (SAR_RS14415)	0,00	3,56	2,73	2,31	3,35	0,42	>=5,00	>=5,00	>=5,00
>2259	Q5HCN6	SAUR2884 (SAR_RS14420)	0,00	1,40	0,20	0,20	1,60	0,00	0,20	1,60	0,00
>2260	capC-L2	SAUR2885 (SAR_RS14425)	0,13	1,69	0,65	0,39	1,69	0,13	0,52	1,82	0,26
>2261	capB-L2	SAUR2886 (SAR_RS14430)	0,14	1,30	1,30	1,01	1,88	0,43	1,01	1,88	0,43
>2262	capA-L2	SAUR2887 (SAR_RS14435)	0,00	2,26	1,21	1,21	2,41	0,00	1,21	2,41	0,00
>2263	icaR	SAUR2888 (SAR_RS14440)	0,00	0,89	0,18	0,18	0,71	0,00	0,18	0,71	0,00
>2264	icaA	SAUR2889 (SAR_RS14445)	0,00	0,73	>=5,00	0,16	0,73	>=5,00	0,24	0,81	>=5,00
>2265	icaD	SAUR2890 (SAR_RS14450)	0,00	>=5,00	>=5,00	0,00	>=5,00	>=5,00	0,00	>=5,00	>=5,00
>2266	icaB	SAUR2891 (SAR_RS14455)	0,00	0,57	>=5,00	>=5,00	>=5,00	0,00	>=5,00	>=5,00	0,00
>2267	icaC	SAUR2892 (SAR_RS14460)	0,00	1,52	0,47	0,28	1,61	0,19	0,28	1,61	0,19

Number/ Position in the figures	Gene ID	ID in PubMLST cg scheme	ED133 vs. swan isolate 15V8707	X22 vs. swan isolate 15V8707	CC522 goat isolate 17CS1042 vs. swan isolate 15V8707	ED133 vs. duck isolate V315	X22 vs. duck isolate V315	CC522 goat isolate 17CS1042 vs. duck isolate V315	ED133 vs. duck isolate V482	X22 vs. duck isolate V482	CC522 goat isolate 17CS1042 vs. duck isolate V482
>2268	lip1	SAUR2893 (SAR_RS14465)	0,10	1,27	0,59	0,64	1,13	0,15	0,69	1,17	0,20
>2269	hisI	SAUR2895 (SAR_RS14475)	0,00	0,95	1,26	1,26	0,95	0,00	1,26	0,95	0,00
>2270	hisF	SAUR2896 (SAR_RS14480)	0,13	2,64	1,58	1,71	2,64	0,00	1,84	2,77	0,13
>2271	hisA	SAUR2897 (SAR_RS14485)	0,00	2,98	1,13	1,13	2,13	0,00	1,13	2,13	0,00
>2272	hisH	SAUR2898 (SAR_RS14490)	0,00	4,32	0,00	0,35	4,66	0,35	0,00	4,32	0,00
>2273	hisB	SAUR2899 (SAR_RS14495)	1,21	3,28	>=5,00	1,38	3,11	>=5,00	1,38	3,11	>=5,00
>2274	hisC1	SAUR2900 (SAR_RS14500)	2,17	2,96	0,00	2,17	2,96	0,00	2,17	2,96	0,00
>2275	hisD	SAUR2901 (SAR_RS14505)	1,52	1,76	0,16	1,52	1,76	0,16	1,44	1,68	0,08
>2276	hisG	SAUR2902 (SAR_RS14510)	0,81	>=5,00	0,16	0,81	>=5,00	0,16	0,81	>=5,00	0,16
>2277	hisZ	SAUR2903 (SAR_RS14515)	0,85	1,22	0,00	0,85	1,22	0,00	0,98	1,34	0,12
>2278	Q5HCL6	SAUR2904 (SAR_RS14520)	0,53	1,16	0,09	0,53	1,16	0,09	0,53	1,16	0,09
>2279	Q5HCL5	(N/A)	>=5,00	1,81	0,00	>=5,00	1,81	0,00	>=5,00	1,81	0,00
>2280	yceI	SAUR2910 (SAR_RS14550)	1,16	>=5,00	0,00	1,16	>=5,00	0,00	1,16	>=5,00	0,00
>2281	drp35	(N/A)	2,57	4,63	0,10	2,57	4,63	0,10	2,88	4,94	0,41
>2282	Q5HCK8	SAUR2912 (SAR_RS14560)	1,15	2,30	0,00	1,15	2,30	0,00	1,15	2,30	0,00
>2283	pcp	SAUR2913 (SAR_RS14565)	0,63	>=5,00	0,31	0,47	>=5,00	0,16	0,47	>=5,00	0,16
>2286	Q2FUS3=bstA	SAUR2914 (SAR_RS14570)	1,51	2,37	0,00	1,51	2,37	0,00	1,51	2,37	0,00
>2287	cna	(N/A)	>=5,00	>=5,00	>=5,00	>=5,00	>=5,00	0,43	>=5,00	>=5,00	>=5,00
>2288	Q7WS08=citT	SAUR2916 (SAR_RS14580)	1,27	>=5,00	0,00	1,27	>=5,00	0,00	1,27	>=5,00	0,00
>2289	rarD	SAUR2917 (SAR_RS14585)	1,21	1,76	0,11	1,10	1,65	0,00	1,10	1,76	0,11
>2290	Q5HCK1	SAUR2918 (SAR_RS14590)	2,60	1,04	0,17	2,60	1,04	0,17	2,60	1,04	0,17
>2291	nixA	(N/A)	0,29	1,57	0,39	0,29	1,57	0,39	0,29	1,57	0,39
>2292	Q5HCJ9=nhoA	SAUR2920 (SAR_RS14600)	3,66	>=5,00	0,12	3,54	>=5,00	0,00	3,54	>=5,00	0,00
>2293	hdeD	SAUR2921 (SAR_RS14605)	0,39	>=5,00	0,00	0,39	>=5,00	0,00	0,39	>=5,00	0,00
>2294	vraD	SAUR2922 (SAR_RS14610)	0,92	0,79	0,00	0,92	0,79	0,00	0,92	0,79	0,00
>2295	vraE	SAUR2923 (SAR_RS14615)	1,38	2,82	0,05	1,38	2,82	0,05	1,38	2,82	0,05
>2296	Q5HCJ5=vraH	(N/A)	2,60	>=5,00	0,00	2,60	>=5,00	0,00	2,60	>=5,00	0,00
>2297	cspC-L3=cspB	(N/A)	0,00	0,00	0,00	0,00	0,00	0,00	0,00	0,00	0,00
>2298	Q5HCI9=immR	SAUR2930 (SAR_RS14650)	1,23	>=5,00	0,00	1,23	>=5,00	0,00	1,23	>=5,00	0,00
>2299	DUF3147_L1	(N/A)	>=5,00	>=5,00	>=5,00	>=5,00	>=5,00	>=5,00	>=5,00	>=5,00	>=5,00
>2300	DUF3147_L2	SAUR2932 (SAR_RS14660)	0,85	3,67	0,56	0,56	3,39	0,28	0,56	3,39	0,28
>2301	noc=parB2	SAUR2934 (SAR_RS14670)	0,36	0,95	0,12	0,48	1,07	0,24	0,36	0,95	0,12
>2302	rsmG=gidB	SAUR2935 (SAR_RS14675)	0,00	0,00	0,00	0,14	0,14	0,14	0,14	0,14	0,14
>2303	mnmG=gidA	SAUR2936 (SAR_RS14680)	0,00	0,00	0,00	0,00	0,00	0,00	0,00	0,00	0,00
>2304	mnmE=trmE	SAUR2937 (SAR_RS14685)	0,22	0,58	0,07	0,29	0,65	0,14	0,22	0,58	0,07
>2305	rnpA	SAUR2938 (SAR_RS14690)	0,00	0,00	0,00	0,29	0,29	0,29	0,29	0,29	0,29
>2306	rpmH	SAUR2939 (SAR_RS14695)	0,00	0,00	0,00	0,00	0,00	0,00	0,00	0,00	0,00
