# Supplementary material for: Molecular Characterization of Chimeric Staphylococcus aureus Strains from Waterfowl
Source: Microorganisms. 2024 Jan 3;12(1):96. doi: 10.3390/microorganisms12010096 (PMC10821479; doi:10.3390/microorganisms12010096)
Supplement: Supplementary file 1 [file microorganisms-12-00096-s001.zip › Supplemental file 4c_Mapping of the avian prophages in study isolates and reference sequences.pdf]

Supplemental file 4b: Mapping of the avian prophages in study isolates and reference sequences. The numbers refer to the position in the prophage genome, and for the sake of comparability, genomes are shown with the integrase gene first, and counting starts with the start codon of the integrase genes. The actual positions in the *S. aureus* genome sequences are CC5-MSSA-ED98, CP001781.1 (2,041,199.. 2,087,963; rc); CC5-MSSA-SA01, CP053075.1 (2,081,890 to 2,128,654; rc); CC692-MSSA-X22, CP042650.1 (2,101,683..2,152,905; rc); CC692-MRSA-CKMM-401M, CP129360.1 (2,088,250..2,140,329; rc); CC692-MSSA-NCTC9546, UHAK01000002.1 (2,069,330 to 2,115,442; rc); 15V8707 (2,003,179.. 2,065,164; rc); 511525-22, Contigs 135 (from pos. 300 on), 150rc, 026, 165, 037; V315 (1,987,006.. 2,031,814; rc); IMT40427, Contigs 259 (from pos. 20 on), 011, 184rc, 222rc, 203rc, 069.

Gene ID	Function / description	Locus-tags	CC5-MSSA ED98	CC5-MSSA SA01	CC692-MSSA X22	CC692-MRSA CKMM-401M	CC692-MSSA NCTC9546	15V8707	511525-22	V315	IMT40427
int	integrase, lysogeny module	SAAV_2070	1.. 1038	1.. 1038	1.. 1038	1.. 1038	1.. 1038	1.. 1038	1.. 1038	1.. 1038	1.. 1038
-	conserved hypothetical protein	SAAV_2069	1200.. 1526	1200.. 1526	1200.. 1526	1200.. 1526	1200.. 1526	1200.. 1526	1200.. 1526	1231.. 1557	1231.. 1557
-	conserved hypothetical protein	SAAV_2068	1539.. 1904	1539.. 1904	-	-	-	-	-	-	-
-	conserved hypothetical protein	SAAV_2067	2244.. 2411	2244.. 2411	2245.. 2412	2244.. 2411	2245.. 2412	2245.. 2412	2245.. 2412	2275.. 2442	2275.. 2442
-	conserved hypothetical protein	SAAV_2066	2510.. 2863	2510.. 2863	-	-	-	-	-	-	-
-	conserved hypothetical protein	SAAV_2065	3184.. 4068	3184.. 4068	-	-	-	-	-	-	-
-	conserved hypothetical protein	SAAV_2064	4137.. 4583	4137.. 4583	-	-	-	-	-	-	-
DUF0955	putative metallo-protease, lysogeny module	SAAV_2063, KMD47_gp05	4600.. 5061	4600.. 5061	3053.. 3514	3052.. 3513	3053.. 3514	3053.. 3514	3053.. 3514	-	-
rep	XRE family regulatory protein, lysogeny module	SAAV_2062	5074.. 5397	5074.. 5397	-	-	-	-	-	-	-
*repC*_SAS0897	helix-turn-helix family protein, putative phage regulatory protein	SAS0897	-	-	3527.. 3856	3526.. 3855	3527.. 3856	3527.. 3856	3527.. 3856	-	-
*repC*_SAAV_2061	putative Cro-like repressor, /helix-turn-helix family protein	SAAV_2061	5561.. 5809	5561.. 5809	-	-	-	-	-	-	-
ant	anti-repressor Ant	SAAV_2060, KMD47_gp08	5822.. 6265	5822.. 6265	-	-	-	-	-	-	-
*rinB*_SAAV_2059	transcriptional activator RinB, replication module	SAAV_2059	6280.. 6429	6280.. 6429	-	-	-	-	-	-	-
-	conserved hypothetical protein	SAAV_2058	6470.. 6682	6470.. 6682	-	-	-	-	-	-	-
-	conserved hypothetical protein	SAOV_0282	-	-	4162.. 4392	4161.. 4391	4469.. 4699	4469.. 4699	4469.. 4699	-	-
Q931J5	putative protein, phi PVL ORF 35-like protein, lysogeny module	-	6751.. 6948	6751.. 6948	4447.. 4644	4446.. 4643	4754.. 4951	4754.. 4951	4754.. 4951	5081.. 5278	5081.. 5278
rep	XRE family regulatory protein, lysogeny module	-	-	-	-	-	4008.. 4208	4008.. 4208	4008.. 4208	-	-
-	putative protein	-	-	-	3989.. 4165	3988.. 4164	4296.. 4472	4296.. 4472	4296.. 4472	6886.. 7062	6886.. 7062
-	putative protein	-	-	-	4162.. 4380	4161.. 4379	4469.. 4687	4469.. 4687	4469.. 4687	7059.. 7277	7059.. 7277
-	conserved hypothetical protein	SAAV_2057	7224.. 7367	7224.. 7367	5078.. 5221	5077.. 5220	5385.. 5528	5385.. 5528	5385.. 5528	5712.. 5855	5712.. 5855
-	conserved hypothetical protein	SAAV_2056, MW1928	7518.. 7838	7518.. 7838	5372.. 5692	5371.. 5691	5679.. 5999	5679.. 5999	5679.. 5999	7348.. 7668	7348.. 7668

Gene ID	Function / description	Locus-tags	CC5-MSSA ED98	CC5-MSSA SA01	CC692-MSSA X22	CC692-MRSA CKMM-401M	CC692-MSSA NCTC9546	15V8707	511525-22	V315	IMT40427
DUF1270	DUF1270 domain-containing protein, replication or lysogeny module	SAAV_2055, KMD47_gp15	7835.. 7996	7835.. 7996	5689.. 5850	5688.. 5849	5996.. 6157	5996.. 6157	5996.. 6157	7665.. 7826	7665.. 7826
DUF1108	conserved hypothetical protein	SAAV_2054	8095.. 8355	8095.. 8355	5949.. 6209	5948.. 6208	6256.. 6516	6256.. 6516	6256.. 6516	8143.. 8403	8143.. 8403
segr=gam	Chromosome segregation protein/host-nuclease inhibitor, Gam family protein, replication module	-	-	-	-	-	-	-	-	8416.. 8952	8416.. 8952
-	hypothetical protein	SAAV_2053	8363.. 8599	8363.. 8599	6217.. 6453	6216.. 6452	6524.. 6760	6524.. 6760	6524.. 6760	-	-
-	conserved hypothetical protein	SAAV_2052	8622.. 9233	8622.. 9233	6476.. 7087	6475.. 7086	6783.. 7394	6783.. 7394	6783.. 7394	-	-
-	conserved hypothetical protein	SAAV_2051	9246.. 9569	9246.. 9569	7100.. 7423	7099.. 7422	7407.. 7730	7407.. 7730	7407.. 7730	-	-
DUF1351_SAAV_2050	hypothetical protein	SAAV_2050	9580.. 10338	9580.. 10338	7434.. 8192	7433.. 8191	7741.. 8499	7741.. 8499	7741.. 8499	-	-
ssbP	single-stranded DNA-binding protein, replication module	-	-	-	-	-	-	-	-	9600.. 10043	9600.. 10043
DUF0968	putative HNHc nuclease	-	-	-	-	-	-	-	-	10055.. 10750	10055.. 10750
-	hypothetical protein	SAAV_2049	10361.. 10996	10361.. 10996	8215.. 8850	8214.. 8849	8522.. 9157	8522.. 9157	8522.. 9157	-	-
*pol*_SAAV_2048	putative phage DNA polymerase	SAAV_2048	11016.. 12749	11016.. 12749	-	-	-	-	-	-	-
primase_SAAV_2047	putative phage primase	SAAV_2047	12771.. 14627	12771.. 14627	-	-	-	-	-	-	-
-	conserved hypothetical protein	SAAV_2046	14624.. 14824	14624.. 14824	-	-	-	-	-	-	-
-	hypothetical protein	SAAV_2045	14890.. 15216	14890.. 15216	-	-	-	-	-	-	-
dnaC	DNA-replication/ATP-binding protein	-	-	-	-	-	-	-	-	12239.. 13024	12239.. 13024
sri	staphylococcal replication inhibitor, replication module	-	-	-	-	-	-	-	-	13021.. 13179	13021.. 13179
DUF3269	DUF3269 family protein, SACOL0345, replication module	-	-	-	-	-	-	-	-	13192.. 13413	13192.. 13413
rusA	RusA family crossover junction endodeoxyribonuclease, replication module	-	-	-	-	-	-	-	-	13423.. 13831	13423.. 13831
DUF3113	DUF3113 family protein, replication module	SAAV_2044	15685.. 15870	15685.. 15870	13523.. 13708	13522.. 13707	13828.. 14013	13830.. 14015	13830.. 14015	13831.. 14016	13831.. 14016
rep	XRE family regulatory protein/helix-turn-helix transcriptional repressor, lysogeny module	-	-	-	-	-	14014.. 14271	14016.. 14273	14016.. 14273	-	-
-	hypothet protein	-	15871.. 16034	15871.. 16034	13709.. 13872	13708.. 13871	14283.. 14446	14285.. 14448	14285.. 14448	-	-
dbp	phi PVL ORF 50-like protein, replication module	SAAV_2043, MW1917	15871.. 16230	15871.. 16230	13709.. 14068	13708.. 14067	14283.. 14642	14285.. 14644	14285.. 14644	-	-
DUF1270	DUF1270 domain-containing protein, replication module	SAAV_2042, KMD47_gp15	16315.. 16479	16315.. 16479	14069.. 14317	14068.. 14316	14643.. 14891	14645.. 14893	14645.. 14893	14717.. 14881	14717.. 14881
DUF1024	hypothetical protein	SAAV_2041	16494.. 16745	16494.. 16745	14332.. 14583	14331.. 14582	14906.. 15157	14908.. 15159	14908.. 15159	14896.. 15147	14896.. 15147

Gene ID	Function / description	Locus-tags	CC5-MSSA ED98	CC5-MSSA SA01	CC692-MSSA X22	CC692-MRSA CKMM-401M	CC692-MSSA NCTC9546	15V8707	511525-22	V315	IMT40427
-	conserved hypothetical protein	SAAV_2040	16735.. 16977	16735.. 16977	14573.. 14815	14572.. 14814	15147.. 15389	15149.. 15391	15149.. 15391	15137.. 15379	15137.. 15379
dut	dUTP pyrophosphatase, replication module	SAAV_2039	16970.. 17506	16970.. 17506	-	-	-	-	-	-	-
*rinB*_SaO11_00291	transcriptional activator RinB, replication module	SaO11_00291	-	-	15056.. 15169	15055.. 15168	15630.. 15743	15632.. 15745	15632.. 15745	15620.. 15733	15620.. 15733
-	hypothet protein	-	-	-	15195.. 15296	15194.. 15295	15769.. 15870	15771.. 15872	15771.. 15872	15759.. 15860	15759.. 15860
DUF1381	DUF1381 domain-containing protein, replication module	SAAV_2038, KMD47_gp30	17543.. 17749	-	-	-	-	-	-	-	-
-	conserved hypothetical protein	SAAV_2037	17746.. 17988	17746.. 17988	-	-	-	-	-	-	-
Q4ZCN3=DUF1514	DUF1514 family protein/conserved putative protein	SAAV_2036	17988.. 18188	17988.. 18188	15238.. 15438	15237.. 15437	15812.. 16012	15814.. 16014	15814.. 16014	15802.. 16002	15802.. 16002
-	hypothetical protein	KMD47_gp40	-	-	17020.. 17145	17019.. 17144	17594.. 17719	17596.. 17721	17596.. 17721	17584.. 17709	17584.. 17709
rinA	phage transcriptional regulator, RinA family protein	SAAV_2035	18216.. 18632	18216.. 18632	-	-	-	-	-	-	-
nuc-HNH	HNH endonuclease family protein	SAAV_2034	18787.. 19101	18787.. 19101	17132.. 17446	17131.. 17445	17706.. 18020	17708.. 18022	17708.. 18022	17696.. 18010	17696.. 18010
*terS*_MW1402	phage terminase small subunit	SAAV_2033	19228.. 19533	19228.. 19533	17567.. 17872	17566.. 17871	18141.. 18446	18143.. 18448	18143.. 18448	18131.. 18436	18131.. 18436
*terL*_MW1401	putative phage terminase, large subunit	SAAV_2032	19523.. 21214	19523.. 21214	17862.. 19553	17861.. 19552	18436.. 20127	18438.. 20129	18438.. 20129	18426.. 20117	18426.. 20117
*port*_SACOL0368	phage portal protein, HK97 family), packaging module	SAAV_2031, SACOL0368	21411.. 22457	21411.. 22457	19750.. 20796	19749.. 20795	20324.. 21370	20326.. 21372	20326.. 21372	20314.. 21360	20314.. 21360
clpP=Q5HIZ6	Clp-protease/head maturation protein, packaging module/phage Clp protease	SAAV_2030	22441.. 23214	22441.. 23214	20780.. 21553	20779.. 21552	21354.. 22127	21356.. 22129	21356.. 22129	21344.. 22117	21344.. 22117
macp	major capsid protein, head module	SAAV_2029	23226.. 24389	23226.. 24389	21565.. 22728	21564.. 22727	22139.. 23302	22141.. 23304	22141.. 23304	22129.. 23292	22129.. 23292
-	hypothet protein	-	24337.. 24447	24337.. 24447	22676.. 22786	22675.. 22785	23250.. 23360	23252.. 23362	23252.. 23362	23240.. 23350	23240.. 23350
htcp	head-tail adaptor Ad1	SAAV_2028, KMD47_gp47	24458.. 24736	24458.. 24736	22797.. 23075	22796.. 23074	23371.. 23649	23373.. 23651	23373.. 23651	23361.. 23639	23361.. 23639
-	hypothetical protein	SAAV_2027, KMD47_gp48, MW1396	24748.. 25080	24748.. 25080	23087.. 23419	23086.. 23418	23661.. 23993	23663.. 23995	23663.. 23995	23651.. 23983	23651.. 23983
tnp-Q6GH59	*IS*3 family transposase	-	-	-	-	-	-	24260.. 25827	-	-	-
-	hypothetical protein	SAAV_2026	25077.. 25478	25077.. 25478	23416.. 23817	23415.. 23816	23990.. 24391	-	-	23980.. 24381	23980.. 24381
DUF3168	DUF3168 domain-containing protein, head module	SAAV_2025, MW1394, KMD47_gp49	25479.. 25874	25479.. 25874	23818.. 24213	23817.. 24212	24392.. 24787	26057.. 26452	25007.. 25402	24382.. 24777	24382.. 24777
*matp*1	phage major tail protein	SAAV_2024	25909.. 26550	25909.. 26550	24248.. 24891	24247.. 24888	24822.. 25463	26487.. 27128	25437.. 26078	24812.. 25453	24812.. 25453
*matp*2	phage major tail protein	SAAV_2023	26642.. 27097	26642.. 27097	24983.. 25438	24980.. 25435	25555.. 26010	27220.. 27675	26170.. 26625	25545.. 26000	25545.. 26000
-	putative protein, tail module	SAAV_2022, KMD47_gp52	27155.. 27505	27155.. 27505	25496.. 25846	25493.. 25843	26068.. 26418	27733.. 28083	26683.. 27033	26058.. 26408	26058.. 26408
-	putative protein	SAAV_2021, KMD47_gp53	27547.. 27705	27547.. 27705	25888.. 26046	25885.. 26043	26460.. 26618	28125.. 28283	27075.. 27233	26450.. 26608	26450.. 26608

Gene ID	Function / description	Locus-tags	CC5-MSSA ED98	CC5-MSSA SA01	CC692-MSSA X22	CC692-MRSA CKMM-401M	CC692-MSSA NCTC9546	15V8707	511525-22	V315	IMT40427
*tmpM3*_SAAV_2020	tail tape measure protein, tail module (un-truncated)	SAAV_2020	27719.. 33895	27719.. 33895	26060.. 32236	26057.. 32234	26632.. 32808	-	-	26622.. 32799	26622.. 32799
*tmpM3*_SAAV_2020, first fragment	tail tape measure protein, tail module (truncated by insertion, first fragment)	SAAV_2020, partial	-	-	-	-	-	28297.. 28619	27247.. 27569	-	-
Q6GHB2	putative protein	-	-	-	-	-	-	28667.. 28960	27617.. 27910	-	-
-	transposase	KI244_10150	-	-	-	-	-	29117.. 29422	28067.. 28372	-	-
-	YdcP family protein	KI244_10155	-	-	-	-	-	29462.. 29797	28412.. 28747	-	-
-	putative protein	KI244_11250	-	-	-	-	-	29801.. 30103	28751.. 29053	-	-
rstA	phage replication initiation factor	-	-	-	-	-	-	30283.. 31359	29233.. 30309	-	-
-	conjugal transfer protein	KI244_10170	-	-	-	-	-	31520.. 32595	30470.. 31546	-	-
-	putative protein	SAPIG1861	-	-	-	-	-	32600.. 32860	31551.. 31811	-	-
*yddD*_ED98	conjugal transfer protein	-	-	-	-	-	-	32866.. 33255	31817.. 32206	-	-
*yddE*_ED98	ATP-binding protein	-	-	-	-	-	-	33290.. 35785	32241.. 34736	-	-
-	putative protein	SAPIG1858	-	-	-	-	-	35797.. 36015	34748.. 34966	-	-
-	putative protein	SAPIG1857	-	-	-	-	-	36022.. 36249	34973.. 35200	-	-
-	putative protein	SAPIG1856	-	-	-	-	-	36246.. 36579	35197.. 35529	-	-
Q6GHC0	cell division protein FtsK	-	-	-	-	-	-	36584.. 37945	35534.. 36895	-	-
-	putative protein	KI244_10210	-	-	-	-	-	37949.. 38116	36899.. 37066	-	-
-	putative protein	KI244_10215	-	-	-	-	-	38135.. 38368	37085.. 37318	-	-
A8Z131	putative membrane protein	-	-	-	-	-	-	38391.. 40322	37341.. 39272	-	-
traG	putative transposase/transfer complex protein G	-	-	-	-	-	-	40312.. 41340	39262.. 40290	-	-
A8Z129	putative exported protein	-	-	-	-	-	-	41346.. 41939	40296.. 40889	-	-
DUF4467	DUF4467 domain-containing (lipo-/cystatin-like) protein	-	-	-	-	-	-	41986.. 42348	40936.. 41298	-	-
-	helix-turn-helix family protein	KI244_10240	-	-	-	-	-	42459.. 42971	41409.. 41921	-	-
*tmpM3*_SAAV_2020, second fragment	tail tape measure protein, tail module (truncated by insertion, second fragment)	SAAV_2020, partial	-	-	-	-	-	43696.. 49549	42646.. 48499	-	-
-	hypothet protein	-	31603.. 32109	31603.. 32109	29944.. 30450	29942.. 30448	30516.. 31022	47257.. 47763	46207.. 46713	30507.. 31013	30507.. 31013
-	hypothet protein	-	32757.. 32900	32757.. 32900	31098.. 31241	31096.. 31239	31670.. 31813	48411.. 48554	47361.. 47504	31661.. 31804	31661.. 31804
Q4ZCS6	putative bacteriophagal tail protein/holin	SAAV_2019	33895.. 34719	33895.. 34719	-	-	32808.. 33641	49549.. 50373	48499.. 49323	32799.. 33623	32799.. 33623
P18179_Tn552	ATP-binding protein for transposon Tn552	-	-	-	32949.. 33764	32947.. 33762	-	-	-	-	-
*tnp*_Tn552-1	transposase for transposon Tn552	-	-	-	33757.. 35201	33755.. 35197	-	-	-	-	-

Gene ID	Function / description	Locus-tags	CC5-MSSA ED98	CC5-MSSA SA01	CC692-MSSA X22	CC692-MRSA CKMM-401M	CC692-MSSA NCTC9546	15V8707	511525-22	V315	IMT40427
res	plasmid resolvase	-	-	-	35173.. 35766	35169.. 35762	-	-	-	-	-
blaI	beta-lactamase repressor inhibitor	-	-	-	36029.. 36409	36026.. 36406	-	-	-	-	-
blaR	beta-lactam sensor/signal transducer BlaR	-	-	-	36399.. 38153	36396.. 38153	-	-	-	-	-
blaZ	penicillin-hydrolyzing class A beta-lactamase BlaZ	-	-	-	38260.. 39105	38260.. 39105	-	-	-	-	-
*sitp*_Q8SDP1	siphon tail protein/peptidase, tail module	SAAV_2018	34728.. 36311	34728.. 36311	39618.. 41201	39618.. 41201	33650.. 35233	50382.. 51965	49332.. 50915	33632.. 35215	33632.. 35215
-	putative protein, tail module	SAAV_2017, MW1387, KMD47_gp57	36311.. 36601	36311.. 36601	41201.. 41491	41201.. 41491	35233.. 35523	51965.. 52255	50915.. 51205	35215.. 35505	35215.. 35505
mitp1	minor structure protein, tail module/hypothetical protein	SAAV_2016, KMD47_gp58	36617.. 38527	36617.. 38527	41507.. 43417	41507.. 43417	35539.. 37449	52271.. 54181	51221.. 53131	35521.. 37431	35521.. 37431
bppU=DUF2479	BppU family baseplate upper protein	SAAV_2015, KMD47_gp59	38527.. 39993	38527.. 39993	43417.. 44883	43417.. 44883	37449.. 38915	54181.. 55647	53131.. 54597	37431.. 38897	37431.. 38897
mitp2=DUF2977	DUF2977 domain-containing protein/minor tail protein, tail module	SAAV_2014, KMD47_gp60	39993.. 40382	39993.. 40382	44883.. 45272	44883.. 45272	38915.. 39304	55647.. 56036	54597.. 54986	38897.. 39286	38897.. 39286
*xldX*_SACOL0386	XLdX family protein/putative protein TIGR01669	SAAV_2013, SACOL0386, KMD47_gp61	40375.. 40539	40375.. 40539	45265.. 45429	45265.. 45429	39297.. 39461	56029.. 56193	54979.. 55143	39279.. 39443	39279.. 39443
Q9MBN6=DUF2951	DUF2951 domain-containing protein, tail module	SAAV_2012	40585.. 40884	40585.. 40884	-	-	-	-	-	39489.. 39788	39489.. 39788
txpA	type I toxin-antitoxin system, in which the antitoxin is a RNA, from *hlb*-converting phages	-	41118.. 41225	41118.. 41225	46437.. 46544	46437.. 46544	40469.. 40576	57200.. 57307	56151.. 56258	40022.. 40129	40022.. 40129
holA	holin	SAAV_2011	41423.. 41725	41423.. 41725	46742.. 47044	46742.. 47044	40774.. 41076	57505.. 57807	56456.. 56758	40327.. 40629	40327.. 40629
amidase	putative amidase	SAAV_2010	41736.. 43190	41736.. 43190	47055.. 48508	47055.. 48508	41087.. 42540	57818.. 59271	56769.. 58222	40640.. 42094	40640.. 42094
D0K6J9-bird	novel bird associated caax amino protease family protein, putative membrane protein	SAAV_2009	43535.. 44275	43535.. 44275	48853.. 49593	48853.. 49593	42885.. 43625	59616.. 60356	58536.. 59276	42439.. 43179	42439.. 43179
D0K6J8-bird	novel bird associated ornithine cyclodeaminase	SAAV_2008	44952.. 45905	44952.. 45905	50269.. 51222	50269.. 51222	44301.. 45254	61032.. 61985	59905.. 60858	43855.. 44808	43855.. 44808
